# Synthesis and structure–activity relationship of peptide nucleic acid probes with improved interstrand-crosslinking abilities: application to biotin-mediated RNA-pulldown†

**DOI:** 10.1039/d2cb00095d

**Published:** 2022-07-21

**Authors:** Enrico Cadoni, Francesca Pennati, Penthip Muangkaew, Joke Elskens, Annemieke Madder, Alex Manicardi

**Affiliations:** Organic and Biomimetic Chemistry Research Group, Department of Organic and Macromolecular Chemistry, Ghent University Krijgslaan 281-7 9000 Gent Belgium; Organic Synthesis Research Unit, Department of Chemistry, Faculty of Science, Chulalongkorn University Phayathai Road Patumwan 10330 Bangkok Thailand

## Abstract

The development of interstrand-crosslinking (ICL) probes for the covalent targeting of DNA and RNA sequences of interest has been extensively reported in the past decade. However, most of the reactions reported so far induce the formation of a stable adduct that cannot be reverted, thus rendering these chemistries less useful in applications where the reversibility of the reaction is needed for further downstream processing of the targeted and isolated sequences, such as enzymatic amplification steps. In this work, we report on the reversibility of the furan-mediated ICL reaction. ICL formation can be conveniently triggered by either chemical (*N*-bromo succinimide, NBS) or luminous stimuli (visible light irradiation in presence of a photosensitizer) and quantitative reversion can be achieved by heating the crosslinked sample at 95 °C, while maintaining the structure of the DNA/RNA targets intact. As a proof-of-concept and showing the benefits of the ICL reversibility, we apply furan-mediated ICL to the pulldown of a target RNA strand from cell lysate.

## Abstract 2

After optimization of interstrand crosslink reaction between furan-containing peptide nucleic acids and target oligonucleotides, the reversibility of the formed product is exploited for the pull-down of a sequence of interest from cell lysates.

## Graphical abstract

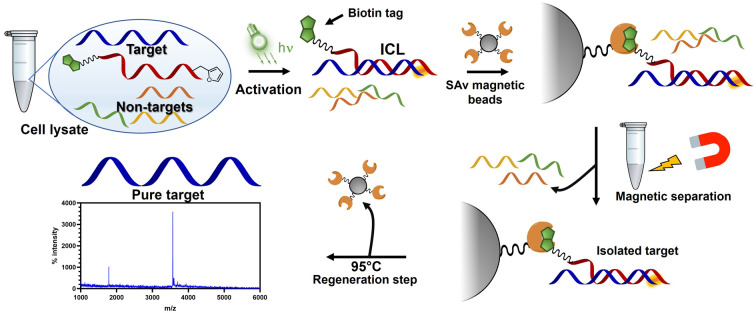

## Introduction

Over the past decades, the use of the unique base-pair recognition of DNA nucleobases has been extensively exploited in different fields, ranging from nanotechnology, diagnostics, and target identification to therapeutic developments, to mention just some of the main applications.^1–7^

Most of the interactions involved in these approaches are of non-covalent nature, featuring the mere formation of stable DNA:DNA or DNA:RNA duplexes. This is often a desired feature, as the exact nature and identity of the target is maintained due to the reversibility of the non-covalent hydrogen bonding. However, this can also represent a limiting factor, as premature dissociation of the complex under stringent conditions might interfere with the envisaged isolation and identification of an unknown target. A possible solution is the use of synthetic oligonucleotide derivatives featuring enhanced duplex stability, such as peptide nucleic acids (PNAs),^8–10^ which have been extensively exploited in the context of DNA and RNA detection, bio-nanotechnology, and therapeutics.^11–14^ Nevertheless, even if higher duplex stabilities can be achieved, the reversibility of the interaction remains.

This limitation can be overcome by introducing reactive functional groups that can specifically and covalently capture the target oligonucleotide. Among all chemistries exploited to date, those functions able to form such covalent interstrand-crosslinks (ICLs) by on-demand activation, showed superior selectivity through accurate spatiotemporal control of system reactivity. Several oligonucleotides and analogues thereof carrying inducible reactive groups such as 3-cyanovinylcarbazole, diazirine, and phenyl selenide have been reported to allow stable covalent bond formation with nucleic acid targets.^15–18^

On the other hand, the formation of a stable ICL could limit the application of these approaches in those cases where system reversibility is needed. Pull-down assays, used for nucleic acid target identification, constitute such an example. These assays mostly rely on the hybridization of an artificial probe, often decorated with a tag, to a complementary target, followed by target isolation through the specific recognition of the tag. As an example, the specific recognition of a biotin tag through its interaction with streptavidin is often used in the context of RNA pull-down for the identification of RNA-binding proteins.^19–21^ Indeed, in this case, the introduction of a covalent modification can hamper the processes (*e.g.* enzymatic amplification) that are needed for processing and identification of the nucleic acid target. The exploitation of covalent modifications that could be reverted in presence of a stimulus or trigger can be extremely useful in this context, as they can ensure complex stability when using harsh washing conditions while allowing at the same time the recovery of the oligonucleotide target after reversal of the formed ICL.

In the last decade, our group has reported on the use of furan modification of oligonucleotides for the realization of DNA–D(R)NA and PNA–DNA ICL and templated ligation systems.^22–26^ In this context, furan was used as a pro-reactive moiety, activated *in situ* by the addition of *N*-bromosuccinimide (NBS) or by singlet oxygen (^1^O_2_)-mediated photo-activation, in presence of a photosensitizer (PS). The resulting keto–enal, generated after furan oxidation, subsequently reacts with a closely located nucleophile, leading to the formation of a stable product, which, under all the conditions tested in previous applications, proved to be irreversibly linked to the target probe.

**Fig. 1:**
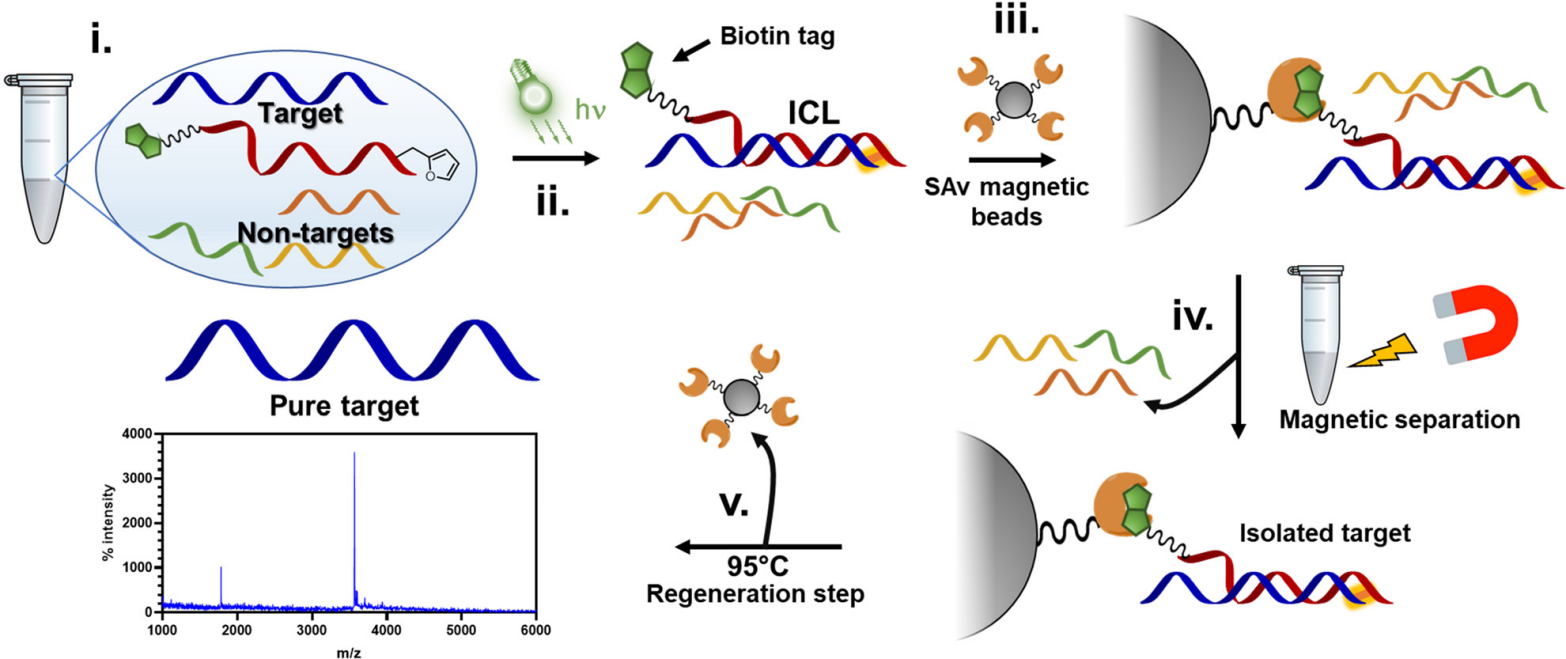
Schematic representation of the experiment proposed in this work. Furan-containing PNA probes equipped with a biotin tag are designed to be complementary to an RNA target in cell-lysate (i). Upon hybridization and subsequent light-irradiation in presence of a photosensitizer, the probe can covalently crosslink to the target (ii). To this solution, streptavidin (SAv)-containing magnetic beads are added, allowing the biotin probes to bind to SAv on the beads (iii). The beads are separated with a magnet and washed multiple times to remove non-crosslinked probes (iv). Finally, the beads are heated at 95 °C for 1 h, allowing for the release of the probe from the beads and simultaneous reversal of the ICL reaction (v).

In this work, we show that the ICL product resulting from the reaction between the activated furan in a PNA strand and the nucleophilic nucleobases present in DNA and RNA oligonucleotides is reversible when heated at 90 °C under physiological pH conditions (PBS buffer, pH 7.4). First, the optimal conditions to maximize the crosslink yield were screened on a model sequence, in which we determined the ideal positioning of the pro-reactive furan moiety and the best-suited type of furan-modified PNA monomer. We then applied this chemistry in a proof-of-concept pull-down approach, exploiting the reversibility of the crosslink reaction to pull down a specific sequence of interest from spiked cell lysates (Fig. 1).

## Results and discussion

In order to design suitable target pull-down probes, we set out to test a series of furan-containing PNA building blocks to identify the most suited furan-monomer for the purpose and to define its optimal position within the PNA probe.

Hereto, various furan-containing monomers were synthesized, the effect of their positioning within the PNA strand evaluated (*i.e.* internal and terminal), and conditions for maximizing the ICL reaction yield screened. In a first stage, all monomers were evaluated using simple NBS addition to activate the system, which allowed fast screening of all possible permutations and combinations. From the results obtained, we identified the most optimal probes, subsequently testing their activation using ^1^O_2_ (generated through irradiation of the reacting solution in presence of a suitable photosensitizer) rather than NBS, to ensure more biology-friendly conditions.

Finally, the best-performing probe was selected and tested for ICL reactivity towards RNA and in cell-lysate, in view of the final pull-down experiment. For the latter, a biotin tag was introduced at the C-terminus of the PNA-strand to enable Streptavidin (SAv) recognition.

### PNA monomer and probe design

In previous work, we reported that the crosslinking ability of furan-containing PNA probes towards target oligonucleotides was negatively affected by nucleobase pairing.^22,27,28^ To understand whether that behaviour was connected to the reduced nucleophilicity of the target nucleophile or to an unfortunate placement of the pro-reactive units on the PNA strand, we decided to explore the possibility to improve the ICL yield by expanding the toolbox of furan-containing monomers. Next to furan introduction through the universal nucleobase building block M1, we explored the possibility to append the small aromatic ring on the backbone of a PNA building block, as well as mounted on a modified nucleobase, designing the monomers M2 and M3 respectively.

**Fig. 2:**
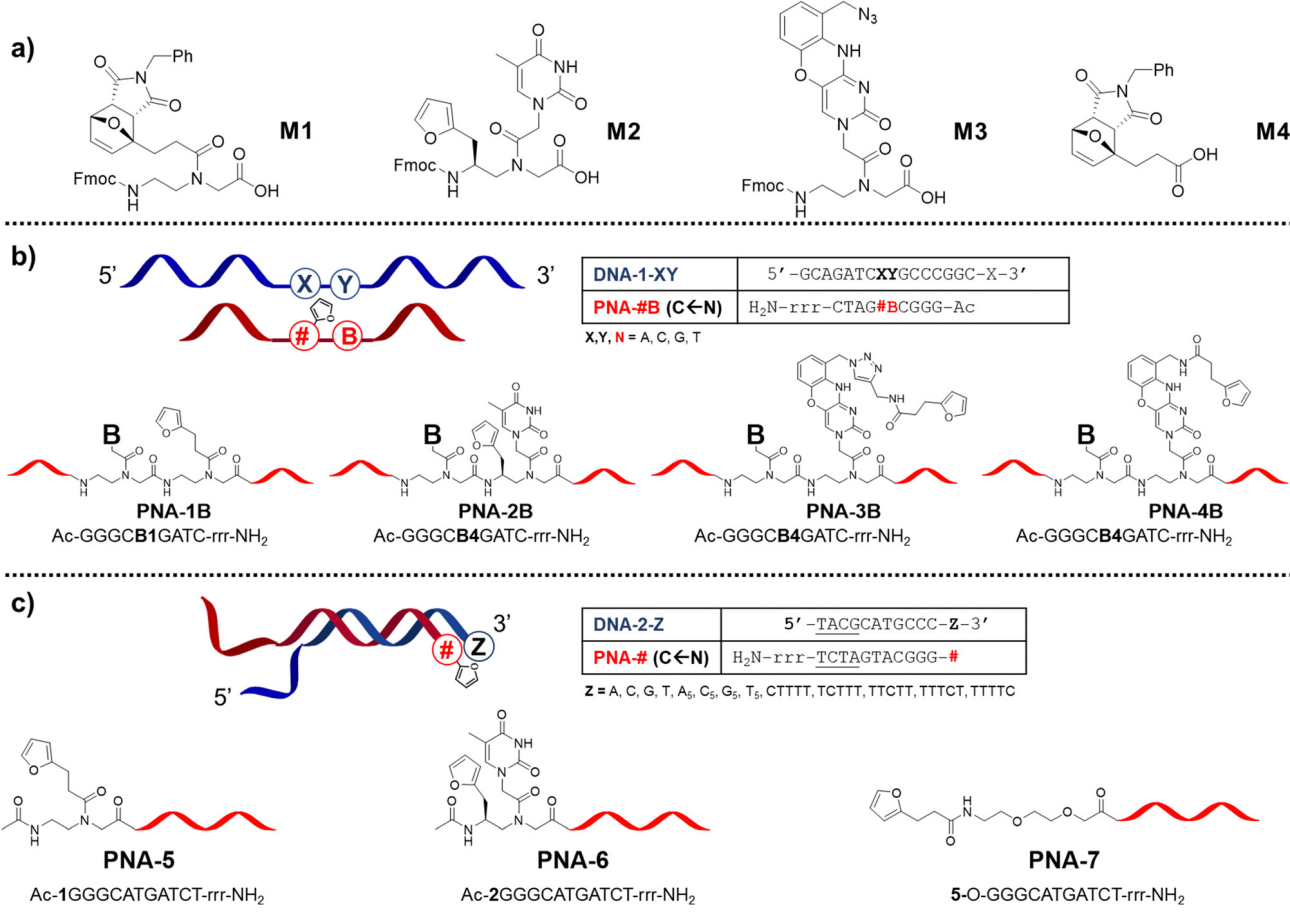
Structures and sequences of probes and monomers used in this study. (a) Monomer structures; (b) design and sequences of the probes for internal furan modification studies; (c) design and sequences of the probes for terminal furan modifications studies. r: l-arginine; O: 2-aminoethoxy(2-ethoxy)acetyl spacer; underlined nucleobases indicate mismatches.

Monomer M2 was designed to maintain base-discrimination abilities, bearing a thymine nucleobase and a furan moiety in position C5. This backbone modification, displaying a furan residue connected to an *S*-configured backbone carbon, was foreseen to allow the formation of PNA:DNA complexes without affecting their stabilities^29^ while orienting the furan ring towards the base at the *N* + 1 position in the target strand (*i.e.* next position toward 3′-end), thus increasing the chances of ICL with the nearby nucleobase. In an alternative approach to maintain the discrimination ability of the monomer and orient the pro-reactive functionality toward potential reactive nucleophiles, we were inspired by the so-called G-clamps.^30^ In this case the furan ring was installed on a phenoxazine unit to allow its positioning in the major groove of the PNA:DNA duplex, thus ensuring easy access to either the facing nucleobase or the one located in the *N* + 1 position. The monomer bearing this modified nucleobase, M3, was designed to display an azide function in its structure that can be exploited as a handle for the later introduction of the furan ring, either during solid-phase synthesis or exploiting an off-resin approach, such as a post-synthesis Huisgen cycloaddition (Fig. 2a).

Given the orientation of the furan ring in these new building blocks, with a directionality toward the *N* + 1 position in the target strand, we set out to evaluate the influence of the nucleobase in the *N* − 1 position in the PNA strand (*i.e.* next position toward the N-terminus, Fig. 2b). Next, in analogy to what was recently reported by Vilaivan's group,^28^ we additionally evaluated the effect of furan-introduction by incorporating M1 or M2 as a terminal modification, next to introduction of a simple furan unit, introduced *via* the building block M4 (Fig. 2c).

### PNA monomer synthesis

**Scheme 1:**
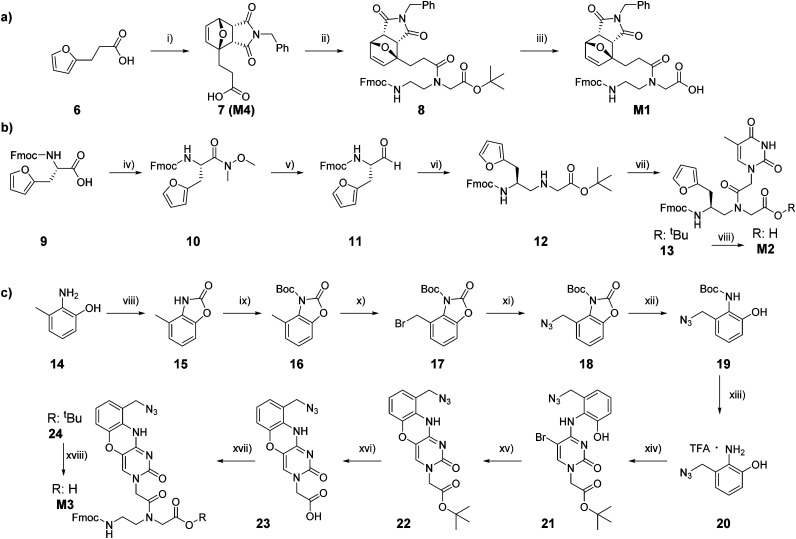
Synthetic pathways for the synthesis of M1 (a), M2 (b), and M3 (c). (i) 1-Benzyl-1*H*-pyrrole-2,5-dione, AcOEt, 75 °C, o.n., 75%; (ii) *tert*-butyl *N*-(2-Fmoc-aminoethyl)glycinate hydrochloride, DhBtOH, EDC·HCl, DIPEA, DMF, 0 °C to r.t., 2 h; 74%; (iii) TFA, DCM, 0 °C to r.t., 6 h, quantitative; (iv) HBTU, DIPEA, *N*,*O*-dimethylhydroxylamine, DMF, 0 °C to r.t., 45′, 71%; (v) LiAlH_4_, THF, 0 °C to r.t., 20′, 86%; (vi) glycine *tert*-butyl ester hydrochloride, DIPEA, NaBH_3_CN, AcOH, MeOH, r.t., 5 h, 81%; (vii) 2-(thymin-1-yl) acetic acid, DhBtOH, EDC·HCl, DIPEA, DMF, 0 °C to r.t., 2 h, 65%; (viii) TFA, CHCl_3_, 0 °C, 4 h, 97%; (viii) CDI, THF, reflux, on, 91%; (ix) Boc_2_O, TEA, DMAP, THF, 0 °C to r.t., on, 92%; (x) NBS, AIBN, CCl_4_, reflux, on, 67%; (xi) NaN_3_, DMF, 0 °C to r.t., 2 h, 67%; (xii) K_2_CO_3_, MeOH, r.t., 98%; (xiii) TFA, CHCl_3_, 0 °C to r.t., quantitative yield; (xiv) *tert*-butyl 2-(5-bromo-2-oxo-4-(1*H*-1,2,4-triazol-1-yl)pyrimidin-1(2*H*)-yl)acetate, DBU, MeCN, r.t., 95%; (xv) KF, EtOH, reflux, 3 days, 50%; (xvi) TFA, CHCl_3_, r.t., 3 h, quantitative yield; (xvii) *tert*-butyl *N*-(2-Fmoc-aminoethyl)glycinate hydrochloride, DhBtOH, EDC·HCl, DIPEA, DMF, 0 °C to r.t., 5 h, 71%; (xviii) TFA, CHCl_3_, r.t., 4 h, quantitative yield.

Temporary protection of the furan ring to avoid alkylation of the C5 position during PNA cleavage from the solid support was earlier demonstrated to minimize the formation of by-product, thus increasing the final yield of synthesis.^27^ In a previously reported approach, temporary protection of the furan ring was performed directly on the solid support, prior to probe cleavage. This approach, despite the yield increase, was not optimal as the conditions for the on-resin Diels–Alder (DA) reaction, exploited for the furan protection, led to the formation of possible undesired maleimide-nucleobase adducts. Therefore, we decided to synthesize a PNA monomer bearing the furan moiety already protected in the form of its DA-adduct. As previously reported, this adduct can be straightforwardly obtained by heating 3-(furan-2-yl)propanoic acid in presence of an excess of *N*-benzylmaleimide.^31^ The lower solubility of the thermodynamic endo-product caused its precipitation, thus driving the reaction towards quantitative conversion and avoiding the need for any purification step. The resulting carboxylic acid 7, which corresponds to monomer M4, was then coupled to the protected Fmoc-PNA backbone, and final deprotection of the *tert*-butyl ester 8 under acidic conditions provided the desired M1 in quantitative yield (Scheme 1a).

Monomer M2 can be easily obtained following previous literature reports, starting from commercially available Fmoc-l-furylalanine.^27,32^ Briefly, the starting carboxylic acid 9 was converted to the corresponding aldehyde 11 by LiAlH_4_ reduction of the Weinreb amide intermediate 10. In turn, 11 was then submitted to reductive amination in presence of glycine *tert*-butyl ester, to obtain the corresponding modified backbone building block 12, to which carboxymethylthymine was coupled before the final *tert*-butyl ester deprotection under acidic conditions to provide the desired M2 (Scheme 1b). The possibility to exploit the aforementioned protection strategy based on the synthesis of a DA-protected monomer was also investigated. DA reaction in presence of benzylmaleimide was tested on different intermediates, but, in all cases, failed to provide the desired enantiopure product in satisfactory yield, therefore it was decided to incorporate the furan-unprotected monomer M2 in the growing polymer.

The synthesis of M3 started from the commercially available 2-amino-3-methylphenol 14, which was converted to the corresponding oxazole 15. An additional protection step with *tert*-butyl dicarbonate was performed to obtain the fully protected derivative 16. This second protection step is required to remove the slightly acidic proton that can interfere with the subsequent radical bromination in presence of 1.1 equivalents of NBS and AIBN to afford 17.^33–35^ Bromine substitution in presence of sodium azide and subsequent stepwise complete deprotection (first oxazole removal under basic conditions and then Boc deprotection under acidic conditions)^36^ provided the azide-bearing 20 as trifluoroacetic (TFA) salt, that can be converted to the desired monomer following previously reported protocols.^37–39^ In particular, it can be exploited in nucleophilic aromatic substitution of *tert*-butyl 2-(5-bromo-2-oxo-4-(1*H*-1,2,4-triazol-1-yl)pyrimidin-1(2*H*)-yl)acetate to obtain the cytosine analogue 21. Cyclization in presence of KF in absolute ethanol provided the desired phenoxazine 22, which, after ester hydrolysis, can be installed on the Fmoc-protected PNA backbone to obtain the desired M3 (Scheme 1c).

### PNA synthesis

All synthesized monomers can be readily incorporated into the growing PNA chain following standard Fmoc-based solid-phase peptide synthesis protocols but monomer-specific manipulations are required to produce the desired PNA probes.

**Scheme 2:**
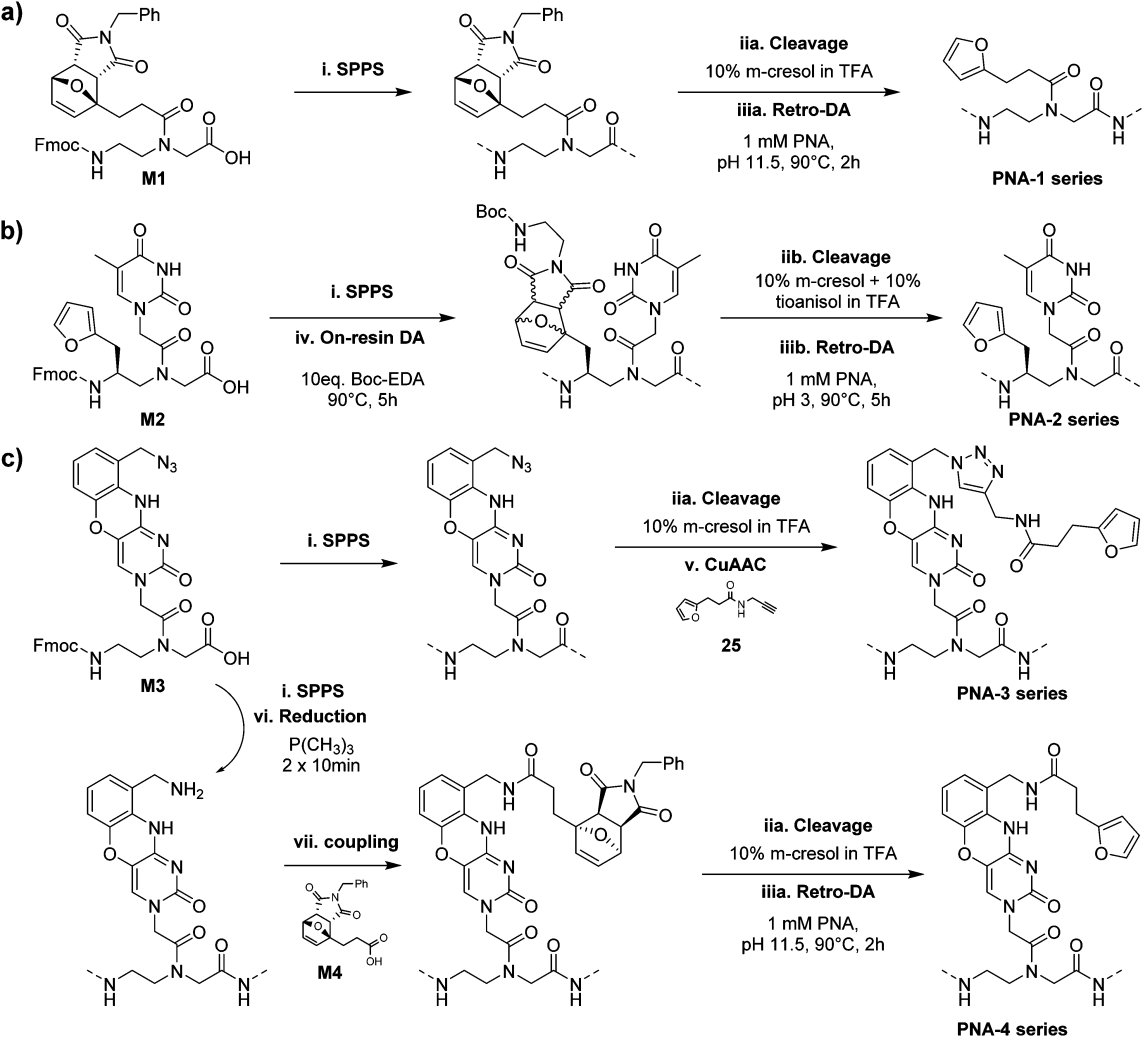
Introduction of the furan modified monomers in the final PNA sequences. (a) The maleimide-protected furan-containing monomer M1 was included in the PNAs on solid support (i). After cleavage (10% *m*-cresol in TFA, iia), the obtained PNA probes were submitted to retro Diels–Alder (DA) conditions to obtain PNA-1 series (pH 11.5, 90 °C, 2 h, iiia). (b) The unprotected building-block M2 was included through SPPS on the PNA probe (i). DA protection was subsequently performed directly on the solid support (iv), prior sequence cleavage (10% *m*-cresol, 10% thioanisol in TFA, iib). The PNA-2 series was obtained after post-cleavage retro-DA under acidic conditions (pH 3, 90 °C for 5 h, iiib). (c) Azide-containing monomer M3 was included on solid support through standard SPPS procedure (i). For PNA-3 series, an unprotected furan-containing alkyne 3-(furan-2-yl)-*N*-(prop-2-yn-1-yl)propanamide was included post-cleavage (10% *m*-cresol in TFA, iia), through CuAAC chemistry (v). For the PNA-4 series, the azide moiety of M3 was reduced (P(Me)_3_, 2 × 10′, vi) prior the on-resin coupling of 2. Subsequent cleavage (10% *m*-cresol in TFA, iia) and retro-DA (pH 11.5, 90 °C for 2 h, iiia) delivered the desired probes.

The PNA-1 series and PNA-5, synthesized using M1 next to regular monomers, were easily obtained by cleaving the probes from the solid support using a classical cleavage cocktail (10% *m*-cresol in TFA) and submitting the resulting crude to retro-Diels–Alder (rDA) furan deprotection under basic conditions (Scheme 2a).^31^ For the PNA-2 series and PNA-6, an additional step was required. As M2 contains a regular 2-alkylfuran, it needed to be protected from alkylation at the C5 position by the formation of a DA-adduct. This was conveniently done directly on the solid support, exploiting the on-resin DA approach.^27^ Once cleaved using a cleavage cocktail with higher scavenger content (10% *m*-cresol and 10% thioanisole in TFA), the crude PNA was subsequently submitted to rDA furan deprotection under acidic conditions to avoid possible epimerisation of the stereogenic center (Scheme 2b). Probes containing monomer M3 were exploited for the preparation of two different series of PNAs. In the PNA-3 series, the furan moiety was inserted through a post-cleavage copper-catalyzed 1,3-dipolar cycloaddition in presence of 3-(furan-2-yl)-*N*-(prop-2-yn-1-yl)propenamide 25. On the other hand, the PNA-4 series was obtained by on-resin Staudinger reduction^40^ of the azide function and coupling of the resulting amine with 7 before cleavage and furan deprotection under basic rDA conditions (Scheme 2c). Finally, for PNA-7 a similar approach as the one exploited for the PNA-1 series was followed, using M4 as building block for the insertion of the protected furan moiety on the solid support.

### Preliminary interstrand crosslink (ICL) experiments

#### Insertion of furan as internal modification

At first, the ICL abilities of the PNA-1, PNA-2, PNA-3, and PNA-4 series were tested, exploiting NBS-activation for fast screening. For each series of PNA, we tested the ICL activity towards the nucleobase facing the monomer (named X), as well as the nucleobase in *N* − 1 position (named Y). All the PNAs were tested with all 16 possible permutations (X, Y = A, C, G, T). The reactions were evaluated at 5 μM strand concentration, in buffered aqueous solution (PBS, pH 7.4). Each reaction was analyzed with HPLC-UV and PAGE experiments. Additionally, the putative ICL adducts were isolated *via* HPLC and characterized by MALDI-TOF to confirm the formation of the desired adduct (please refer to examples in ESI,† Section 5). Given the large number of experiments performed, we here report the results referring to the general code #B-XY, where # is the monomer included in the PNA probe (1–4), B is the nucleobase next to the modified monomer, and X and Y are the DNA nucleobases facing # and B respectively. For the same reason, “good yielding” systems were defined as those delivering an ICL% higher than 60%, as determined by HPLC-UV analysis. DNA:PNA complex formation was confirmed on representative samples (containing one or two mismatches) *via* UV-melting experiments (data not shown).

**Fig. 3:**
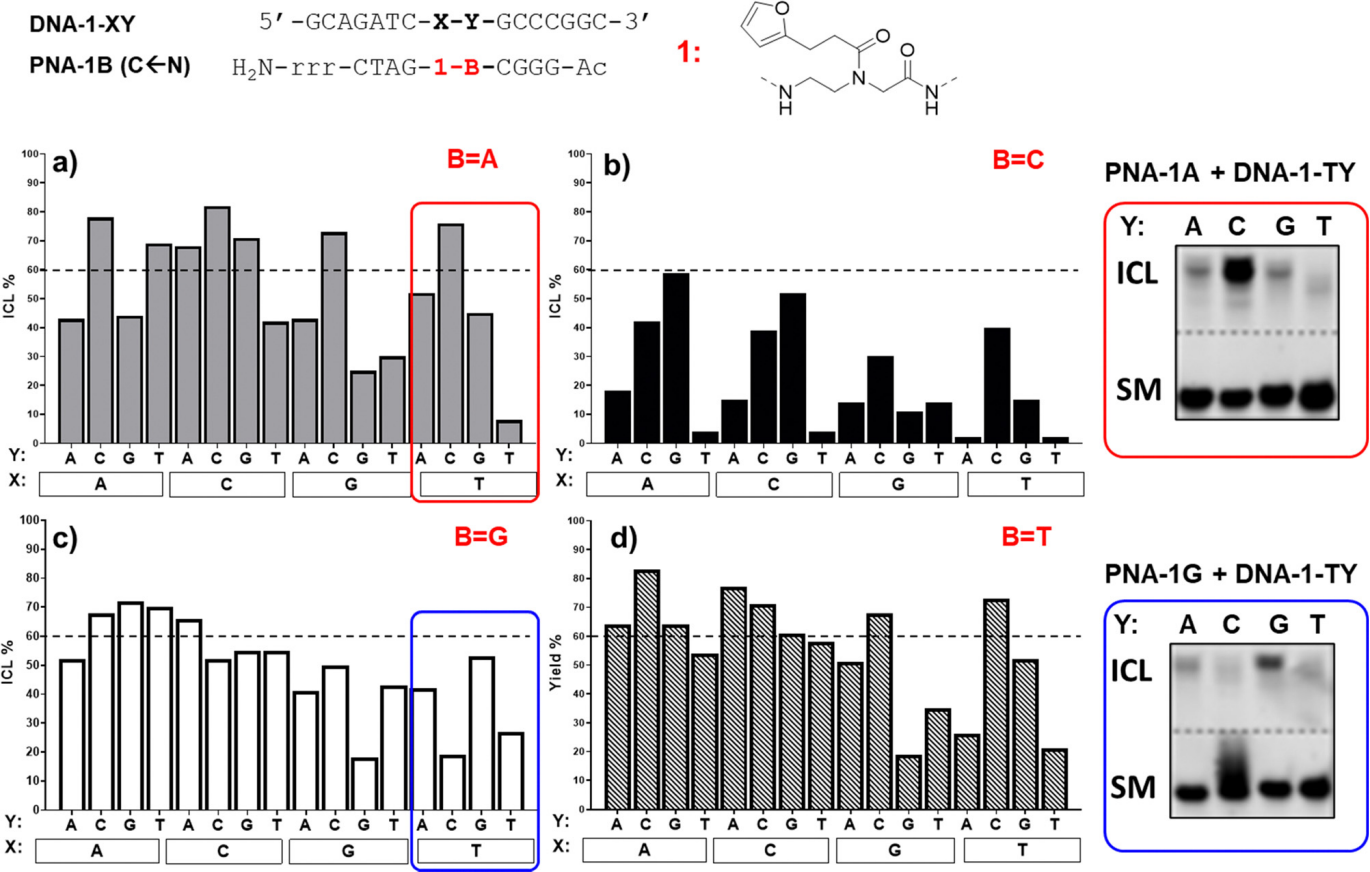
ICL yield (%) of PNA-1 series equipped with 1, towards DNA-1-XY (X = nucleobase facing 1; Y = nucleobase facing B), and representative PAGE analysis examples. (a) ICL% of PNA-1A. (b) ICL% of PNA-1C. (c) ICL% of PNA-1G. (d) ICL% of PNA-1T. The dashed line indicates the 60% yield threshold. Experiment performed in PBS buffer, pH 7.4 (100 mM NaCl, 10 mM phosphate), at 5 μM strand concentration, upon addition of 4.0 equivalents of NBS. Experiments were performed as single replicates.

**Fig. 4:**
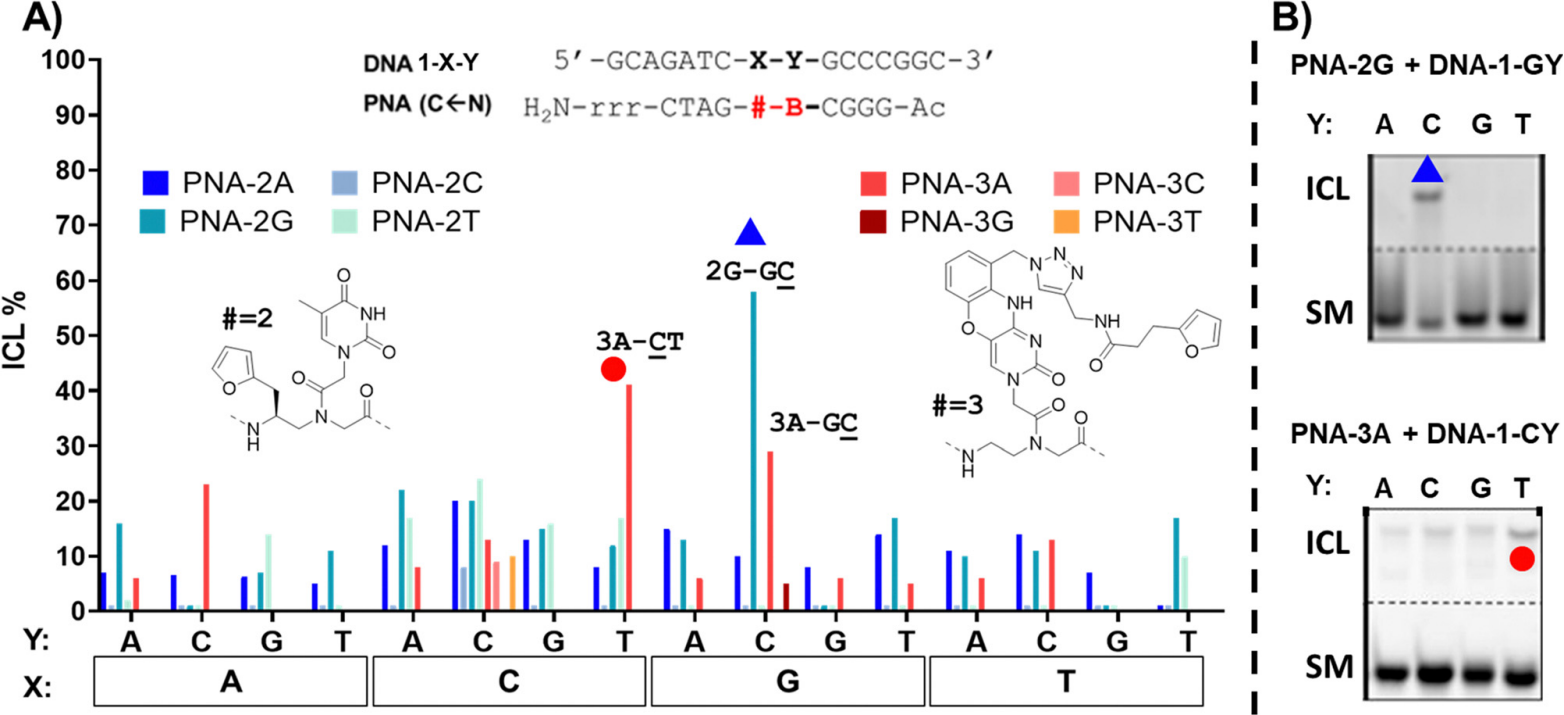
(A) ICL% of PNA-2 (a) and PNA-3 (b) series towards DNA-1-XY. Experiment performed in PBS buffer, pH 7.4 (100 mM NaCl, 10 mM phosphate), at 5 μM strand concentration, upon addition of 4.0 equivalents of NBS. The experiment leading to a confirmed ICL formation are indicated in the graph (2G-GC̲, 3A-C̲T, 3A-GC̲). (B) Examples of PAGE analysis for PNA-2G + DNA-1G-Y and PNA-3A + DNA-1Y. The reacting nucleobases are underlined. Experiments were performed as single replicates.

As can be seen in Fig. 3, the ICL% is highly variable and largely dependent on the monomer used. As a general trend, the PNA-1 series display maximal yields (>60% in 20 permutation cases out of 64 combinations) as compared to PNA-2, PNA-3 (Fig. 4) and PNA-4 series. These results are in line with the behaviour of the monomer as a universal nucleobase, which does not discriminate the facing nucleobase. The ability of these PNAs to induce ICL depends on the ability of 1 to react with the facing nucleobase, owing to the reduced rigidity induced by the lack of base-pair recognition.

More in detail for the PNA-1 series, as a general trend, higher yields are found when 1 is facing either adenine, cytosine, or guanine (X = A, C or G), and Y (the nucleobase in *N* + 1 position in the target strand) and B (the PNA nucleobase facing Y) are not matching. In contrast, the lowest yields are found when the modified nucleobase is facing a thymine and/or Y and B are paired. This can be explained by the need for a proximate nucleophilic moiety (A, C, and G exocyclic amines), available for engaging in reaction with the activated furan probe. This is not possible for thymine or when the exocyclic amines are involved in hydrogen bonding. The results obtained highlight the tendency of 1 to crosslink to a cytosine in the *N* + 1 position (Y = C, as in 1A-TC and 1A-GC), when the two bases Y and B are not matching. In contrast, when Y and B are paired, the system shows a higher preference for crosslinking towards a facing adenine rather than cytosine (*e.g.*1A-A̲T*vs.*1A-C̲T, 1C-A̲G*vs.*1C-C̲G). These results are in line with our previous report,^22^ where the ability of the monomer to crosslink to its target was only tested for permutations 1A-XT (1A-AT, 1A-CT, 1A-GT and 1A-TT). As can be seen in Fig. 3b, PNA-1C displayed general lower activity as compared to the other probes. This could be connected to the possible formation of an intrastrand crosslink with the neighbouring C when it is not paired with the facing Y. Indeed, when Y = G the reactivity of the system is restored and the activated furan can then react with the facing nucleobase X (1C-A̲G, 1C-C̲G). The results were confirmed by PAGE (please refer to Fig. S4, ESI†), and the formation of an ICL confirmed by MALDI analysis of the isolated products (see ESI,† Section 4 for representative examples).

In contrast to 1 and its behaviour as a universal nucleobase, for the other monomers ICL was only observed in a few cases. This is connected to the ability of these monomers to hybridize to the complementary base, given the presence of a (modified) nucleobase. In the case of the PNA-2 series, synthesized using monomer M2, containing a thymine able to recognize a facing adenine, the first evidence of the lowered system reactivity was delivered through PAGE electrophoresis. In this case, only a limited number of slower-running bands, less intense than in the former case, appeared (see Fig. S5, ESI†). In addition, when fully-matching probes were tested (*i.e.*2A-AT, 2A-CG, 2G-AC and 2T-AA), the additional band was related to the formation of a very stable PNA:DNA duplex that could not be melted under denaturing PAGE conditions (7 M urea) and not to a genuine ICL product. This was additionally supported by the perfect overlap of the DNA peak in HPLC, regardless of furan activation. The only clear indication which supports the formation of an ICL product is the experiment 2G-GC. The results pointed towards the formation of an adduct compatible with a crosslink between 2 and the facing G. This result can be explained by the induced system-rigidity due to the G:C pairing, and the formation of a 2(T):G wobble base-pairing that orients the free, exocyclic amine of the guanine towards the furan residue. Although the general ICL yield that resulted was lower than in the case of the universal nucleobase 1, the additional recognition element provides an increased crosslink selectivity, allowing for better discrimination of the target. Judging from the results obtained with 2 as internal modification, its inclusion can be useful for the further development of targeting probes where sequence-selectivity for a specific point mutation is needed (see Fig. 4).

In case of the PNA-3 series (Fig. 4), equipped with a modified phenoxazine able to recognize guanine, PAGE analysis revealed the formation of additional bands corresponding to stable PNA:DNA duplexes when fully matching probes were used (3A-GT, 3C-GG, 3G-GC and 3T-GA, see Fig. S6, ESI†). The formation of bands with lower electrophoretic mobility and compatibility with the formation of ICL was found in presence of permutations featuring an unpaired cytosine in *N* + 1 position (Y = C). This tendency was confirmed by HPLC experiments, highlighting a reduction of the starting material peak in case of 3A-TC, 3A-AC and 3A-GC, with the latter being the highest yielding of the three. This can be explained by the selective clamping of the facing guanine, which orients the reactive furan moiety towards the exocyclic amine of the adjacent cytosine. PAGE and HPLC data confirmed the highest yield in the 3A-CT case, which can also in this case be explained by the complementarity of B (= A) and Y (= T) that conveniently orients the furan-monomer to the facing cytosine (X = C). However, MALDI analysis did not produce satisfactory data supporting the formation of any ICL product, revealing the presence of signals with a mass shifted by 1000 Da from the expected product MW (3700). This was attributed to the instability of the phenoxazine core under oxidizing conditions (NBS) and towards MALDI ionization conditions.

The analysis of ICL experiments performed with the PNA-4 series revealed to be more challenging as compared to the PNA-3 series. While PAGE analysis supports the formation of a stable PNA:DNA duplex band for the fully matched case 4C-GG (therefore, a putative ICL formation towards an *N* − 1 guanine residue, see Fig. S7 for PAGE analysis, ESI†), the broadening of the DNA peaks in HPLC chromatograms upon NBS activation made the ICL% calculation challenging. In addition, no new peaks in the chromatogram or MALDI signals with values compatible to the formation of a product, were found. In summary, although a reaction with the DNA is happening (supported by the appearance of new bands in PAGE and by the radical change of the HPLC-UV chromatograms upon NBS addition, see ESI,† Fig. S7), the different linker introduced in 4 could lead to a different interaction with the DNA target that we were not able to fully characterize.

#### Insertion of furan as a terminal modification

From the studies performed in the previous section it became clear that the universal nucleobase performs better in terms of ICL activity, when compared to the monomers bearing a nucleobase. Furthermore, M3 failed to deliver clear evidence of ICL product, presumably due to the degradation of the phenoxazine core under the experimental conditions. Therefore, we decided to exclude the latter from the ICL-evaluation studies with furan monomers in terminal position and to focus on PNA-5, obtained using the universal monomer M1, and PNA-6, containing the thymine-modified monomer M2. In addition, we decided to evaluate the behaviour of PNA-7, obtained using the furan-containing monomer M4, bearing a more flexible aliphatic chain and verifying the influence on the alkylation outcome.

DNA target sequences were designed to be complementary to the PNA probes. The terminal nucleobase Z was either a single base residue (Z = A, C, G or T) for evaluation of reactivity towards a single extra base, or a 5-bases overhang (Z = A5, C5, G5 or T5). In addition, we performed a C-scan experiment, in which a C_1_T_4_ overhang was designed to bear a cytosine residue at different position of the T-stretch (Z = CTTTT, TCTTT, TTCTT, TTTCT or TTTTC). This last experiment was designed for better identification of the preferred position for cytosine to react with the facing PNA probe.

Preliminary experiments were performed using fully matching 11-mer DNA probes. In this geometry, the high thermal stability of the PNA:DNA duplex resulted in only partial melting under the analytical conditions used (7 M urea), making the ICL analysis more challenging. Therefore, it was decided to reduce the complementarity between target DNA and PNA probes from 11 to 7mers, to allow correct and unambiguous visualisation of ICL product formation and to clearly distinguish it from the formation of a thermally stable PNA:DNA duplex.

**Fig. 5:**
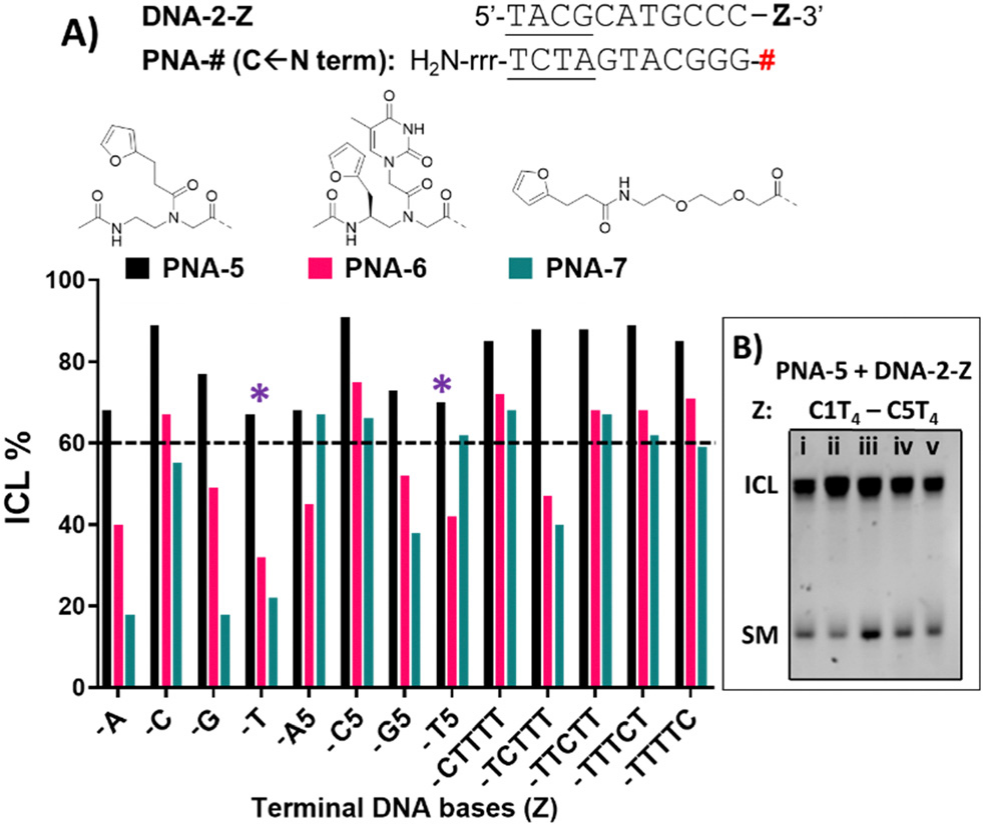
(A) ICL% for PNA-5 (black), PNA-6 (pink) and PNA-7 (green), in presence of DNA-2-Z (Z = a single base, a 5-base overhang or a C-scan experiment). Z is indicated on the X-axis of the graph. The dashed line indicates a 60% ICL threshold. (B) Example of PAGE analysis of C-scan experiment in presence of PNA-5 and DNA-2-C1T4 (i), DNA-2-C2T_4_ (ii), DNA-2-C3T_4_ (iii), DNA-2-C4T_4_ (iv), DNA-2-C1T_4_ (v). Experiment performed in PBS buffer, pH 7.4 (100 mM NaCl, 10 mM phosphate), at 5 μM strand concentration, upon addition of 4.0 equivalents of NBS. * = ICL to cytosine at *N* − 1 position. Experiments were performed as single replicates.

If compared to internally modified probes, the ICL yield for the terminally-modified PNAs resulted to be higher, and was more evident when adopting HPLC-UV analysis (Fig. 5) as compared to PAGE analysis (Please refer to Fig. S8 and S9, ESI†). However, dependence on the monomer included in the probe is observed, and yield was maximal in the PNA-5 case, which showed in all cases an ICL yield above 60% (Fig. 5). Maximal ICL activity was found when Z is represented by a cytosine residue or multiple cytosine residues, but reactivity is shown even in presence of other nucleobases, including thymine, albeit to a lower extent. The observed ICL formation in presence of Z = T can be attributed to the breathing of the duplex and the consequent exposure of the stretch of cytosines located at the 3′-end of the target sequence. This is even more evident when a T_5_ tail is introduced on the targeted DNA. The crosslink formation with the adjacent cytosine was confirmed by MALDI-TOF analysis, where a mass compatible with a PNA–cytosine adduct was found (refer to ESI,† Fig. S14).

Next to PNA-5, PNA-6 equipped with 2 displayed overall good ICL yields. In contrast to internal modification, 2 in terminal position resulted in higher ICL%, with yields above 60% in presence of a facing cytosine or a cytosine stretch. Finally, PNA-7 obtained using monomer M4 displayed the lowest activity, although the general preference for a facing cytosine or cytosine stretch was maintained. Also in these cases, the retained reactivity of the system when facing a thymine or a thymine stretch, could indicate the possibility for the monomers to react with the cytosine- in position *N* − 1 of the target DNA strand and was supported by the C-scan experiment (*vide supra*). Surprisingly, while the C-scan experiment in PNA-5 did not show reaction dependence from the cytosine position, in both PNA-6 and PNA-7 a drop in ICL yield was observed when cytosine was included in the second position of the stretch. This could be connected to a reduced accessibility of the cytosine in the *N* + 2 position.^41^ This result was confirmed by PAGE analysis (see Fig. 5B and Fig. S9 in ESI†).

In order to exclude/minimize the contribution of the C stretch at the 3′-end of the target DNA, we decided to investigate the reactivity of the system in presence of a different terminal nucleobase (*i.e.* adenine) on the target. Thus, we synthesized furan-containing probes bearing an extra thymine residue on the N-termini of each sequence (PNA-5T and PNA-7T). The thymine-containing PNA-6 was used as such, with no further modifications of the sequence.

**Fig. 6:**
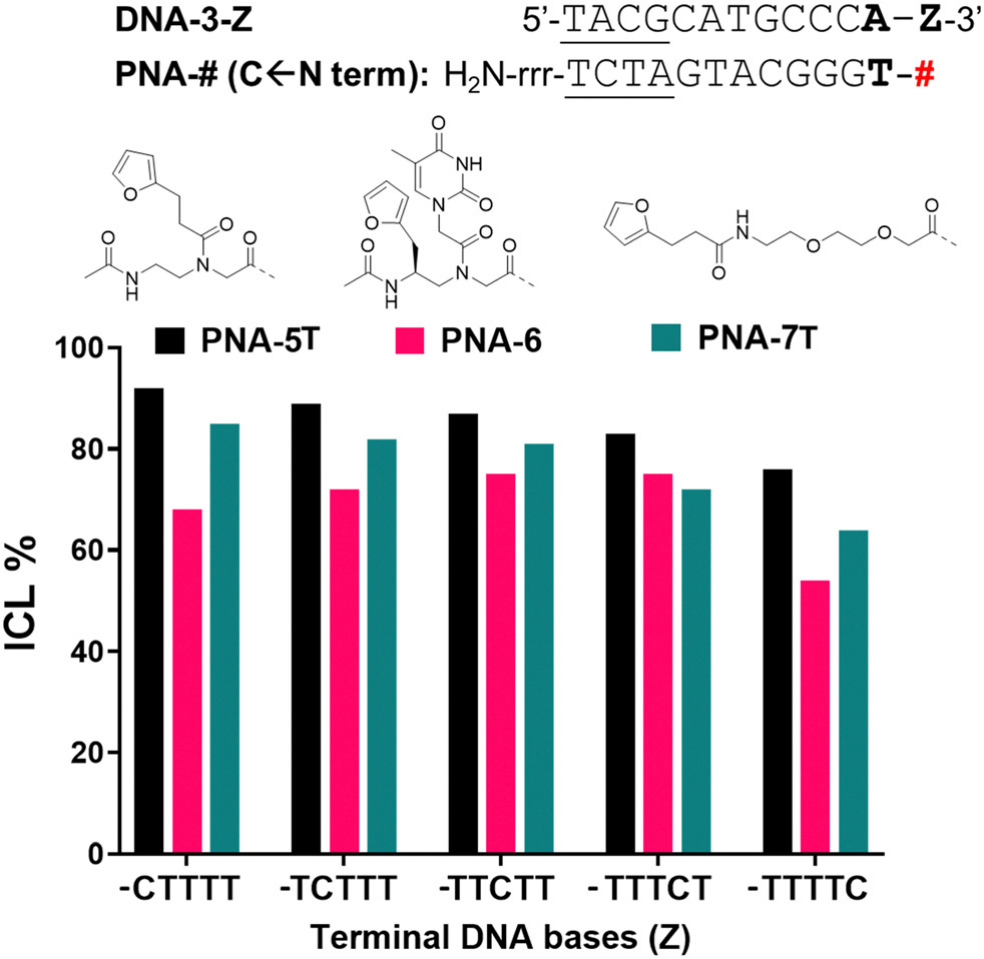
ICL% for the C-scan experiment of PNA-5T (black), PNA-6 (pink) and PNA-7T (green), in presence of DNA-2-Z (Z = C-scan experiment). Z is indicated on the *X*-axis of the graph. Experiments were performed in PBS buffer, pH 7.4 (100 mM NaCl, 10 mM phosphate), at 5 μM strand concentration, upon addition of 4.0 equivalents of NBS. Experiments were performed as single replicates.

When inserting an extra base-pair in the sequence (and replacing a terminal C with an A), the behaviour of the C-Scan experiment followed a more predictable trend (Fig. 6), indeed reactivity of PNA-5T and -7T decreased with the increasing distance of the target C. This was not the case for PNA-6, which preserved its ICL yield towards the target up to position 4 (Z = TTTCT), which only drops starting from position 5 (Z = TTTTC).

#### From NBS to singlet oxygen-mediated furan activation

Next, we decided to evaluate the influence of a different method for furan activation on the ICL yield, exploiting the generation of singlet oxygen obtained by light irradiation in presence of methylene blue (MB) as the photosensitizer (PS). The initial choice for MB was driven by the wavelength required to trigger the PS activation (*λ*_max_ = 665 nm, red light), thus guaranteeing at the same time higher biological applicability due to lower background absorption by cell lysate components and the deeper light penetration,^42^ as well as fast activation of the system due to the high ^1^O_2_ quantum yield.^43^ In addition, as demonstrated in previous studies, under these irradiations conditions no damage of the DNA probes was observed.^24,44^

**Fig. 7:**
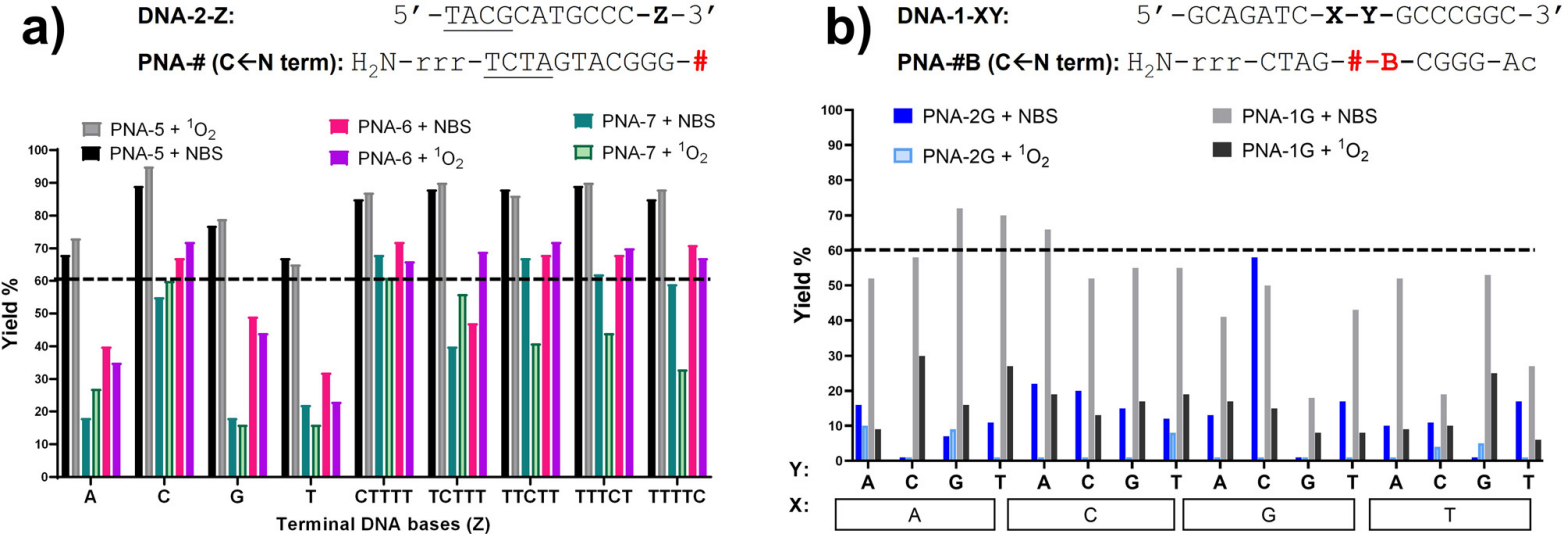
Comparison of ICL% under NBS and ^1^O_2_-mediated furan activation. (a) Comparison of terminal furan modifications included in PNA-5, PNA-6 and PNA-7, upon NBS and ^1^O_2_-mediated activation; (b) comparison of the internal modification included in PNA-1G and PNA-2G upon NBS and ^1^O_2_-mediated activation. The dashed line indicates 60% ICL% threshold. Experiment performed in PBS buffer, pH 7.4 (100 mM NaCl, 10 mM phosphate), at 5 μM strand concentration, upon addition of 4.0 equivalents of NBS or in presence of 2 μM MB concentration for the light-triggered setup (20′ light irradiation). Experiments were performed as single replicates.

The variation of the activation protocol had a different effect depending on the position of the furan moiety. Indeed, the terminal-furan probes PNA-5, PNA-6, and PNA-7 showed similar yields regardless of the activation protocol (Fig. 7a for the single base experiments and C-scan experiments, Fig. S3 for the five-bases overhang, ESI†). This was not the case for the probes bearing internal modifications, which showed a lower alkylation yield in most of the cases, more evident when PNA-1G and PNA-2G probes were employed (Fig. 7b). The different mechanism of activation, in particular the different intermediates that are formed before leading to the final keto-enal species, might have an impact on the structure of the double strand, negatively affecting the alkylation outcome when ^1^O_2_ activation is performed. In addition, we cannot exclude a possible mechanism in which the highly reactive brominated intermediates, generated by the NBS-mediated activation, are directly attacked by the exocyclic amines of the DNA target. The lower solvent exposure of the internal positions can avoid the water quenching, while this phenomenon is reduced in the case of the more exposed terminal position.

Summarizing the results obtained in the preliminary ICL experiments, to maximize the ICL yield, alkylating probes based on terminal incorporation of universal monomers M1 and M4 should be used. These probes showed high reactivity and good tolerance to different activation protocols. The possibility to use these modifications to target all reactive nucleobases placed from *N* − 5 to *N* − 1 position, with a preference for cytosine (where ICL% was higher than 90%), allows great flexibility in the design of pull-down probes. Higher sequence selectivity was obtained when the reactive furan was placed in a central position of the PNA probe, especially using M2 and M3. Unfortunately, the low tolerance of these modifications to singlet oxygen discourages their use in applications where NBS activation is not allowed. Finally, the incorporation of M1 in a central position showed intermediate properties.

### Towards a methodology for PNA-mediated RNA pulldown: crosslinking to RNA, reactivity in cell-lysate, and crosslink reversibility

**Fig. 8:**
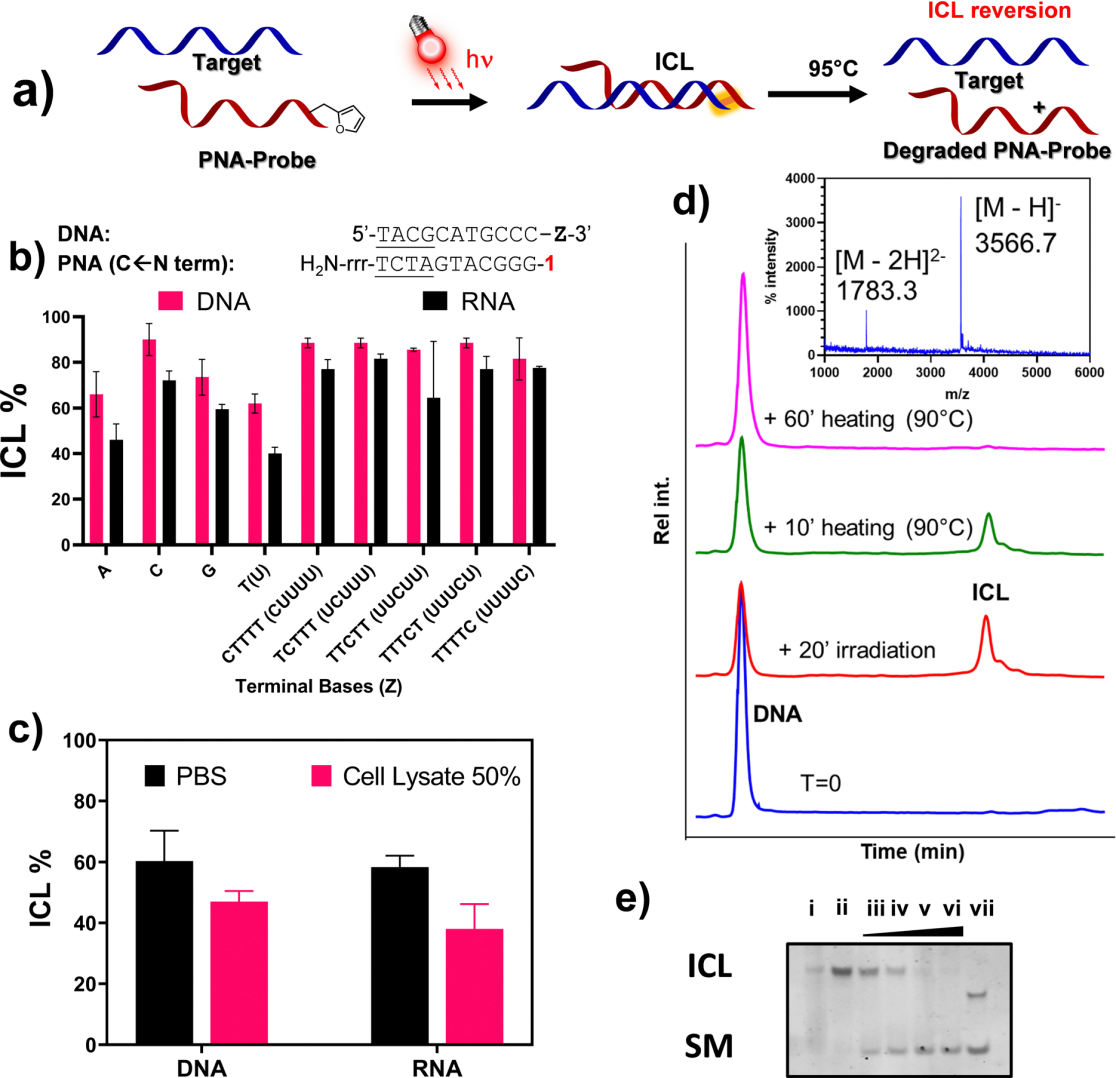
Expansion of the ICL reaction towards RNA targets and crosslink reversibility studies. (a) Representation of the ICL reaction and its reversion; (b) comparison between the ICL reaction of PNA-5 in presence of RNA-Z and DNA-2-Z. Experiment performed in PBS buffer, pH 7.4 (100 mM NaCl, 10 mM phosphate), at 5 μM strand concentration, in presence of 2 μM MB (20′ light irradiation). (c) Comparison between the ICL% of PNA-5 in PBS pH 7.4 and Cell lysate (55 million cells per mL) towards DNA-2-A and RNA-2-A. Experiment performed in PBS buffer, pH 7.4 (100 mM NaCl, 10 mM phosphate), at 5 μM strand concentration, in presence of 2 μM MB, eventually supplemented with cell lysate. (d) ICL reversion between PNA-5 and DNA-2-A. Chromatograms indicate the reaction before irradiation (blue trace), 20 minutes light irradiation (red line) and after heating at 95 °C for 10 (green) and 60 minutes (purple). Experiment performed in PBS buffer, pH 7.4 (100 mM NaCl, 10 mM phosphate), at 5 μM strand concentration, in presence of 2 μM MB (20′ irradiation). The insert shows the MALDI-TOF analysis of the purified DNA peak after ICL reversion, upon heating the sample at 95 °C for 1 hour; (e) PAGE analysis of the ICL reaction of PNA-5 in presence of RNA-C, and ICL reversion upon heating at 95 °C. Lane i: RNA:PNA duplex; lane ii: ICL reaction after 20′ irradiation in presence of MB 2 μM; lane iii: 5′ heating at 95 °C; lane iv: 10′ heating at 95 °C; lane v: 30′ heating at 95 °C; lane vi: 60′ heating at 95 °C; lane vii: RNA starting material. All experiments shown in the figures were performed in triplicate.

Based on the outcome of the previous preliminary studies and given the preference for using ^1^O_2_ over NBS to ensure the activation of furan in cell lysate for the final pull-down application, we decided to focus our attention on the use of M1 in terminal position, which displayed higher ICL% as compared to other monomers and type of modifications. We therefore decided to extend the ICL study from DNA to RNA targets, only focusing on the PNA-5 probe, and evaluate the possibility to revert the formation of the ICL (Fig. 8a for a schematic representation of the approach).

At first, we decided to validate the crosslinking towards a complete series of RNA targets. As shown in Fig. 8b, despite an overall reduction of the ICL yields, where variation is in most cases lower than 10%, the general crosslinking trends do not significantly differ when moving from DNA to homologous RNA targets (DNA-2-A and RNA-A). Crosslinking was additionally studied in presence of a high concentration of various ions (Ca^2+^, Mg^2+^, K^+^), as well as high molecular crowding conditions to simulate the physiological concentration of species in living cells, using PEG6000 (30% w/v). In none of these cases, the outcome of the reaction appeared to be influenced (please refer to ESI,† Fig. S2). Moving from buffered solution to cell lysate, the ICL% was reduced for both DNA and RNA targets (Fig. 8c). The general reduction of the observed ICL% under these conditions can be explained by the presence of surfactants (*e.g.* SDS), free thiols and other stabilizers that can interfere with the formation of the PNA:RNA duplex and/or quench the furan once activated into its keto-enal form. Nevertheless, the light induced, furan-based, ICL formation with target RNA strand in cell lysate still showed satisfactory yield.

Finally, we tested the possibility to revert the ICL by heating the solution at 95 °C, thus allowing regeneration of the target DNA. The idea of a possible reversion of the furan-mediated ICL, was originally hypothesized to explain abnormal behaviours observed when recording the melting temperature of some crosslinked samples (data not shown). The possibility of reverting the ICL reaction with a simple heating step proved very convenient for the final envisaged application, as heating of the streptavidin beads is a generally accepted methodology to release the probe-target complexes in common pull-down applications. The analysis of the ICL reversion was performed in PBS buffer, irradiating the furan-containing probe PNA-5 for 20′ in presence of the complementary target DNA-2-A, to induce the formation of the ICL-adduct. The crosslinked sample was then heated at 95 °C, sampled at different time points, and analysed through HPLC-UV. As shown in Fig. 8d, the area of the ICL peak gradually decreased over time, with complete disappearance after 60 minutes heating. Simultaneously, the area of the DNA peak, reduced by more than 50% after reaction with the PNA probe, gradually increased until becoming superimposable with the starting material peak, thus indicating a full reversal of the ICL. MALDI analysis of the purified DNA peak revealed an adduct with the same MW as the starting DNA, hence excluding DNA depurination as a consequence of crosslink reversal. Similar results were obtained using RNA probes, where the reversion of the reaction was complete within a similar time span (see Fig. S19, ESI†). In addition, we were not able to find any traces of degradation product attributable to the prolonged heating of the system (please refer to Fig. S20, ESI†). The reaction outcome was furthermore confirmed by PAGE analysis (Fig. 8e), where no additional bands appeared when heating up the system for a prolonged period of time.

### Proof-of-concept pull-down experiments

**Fig. 9:**
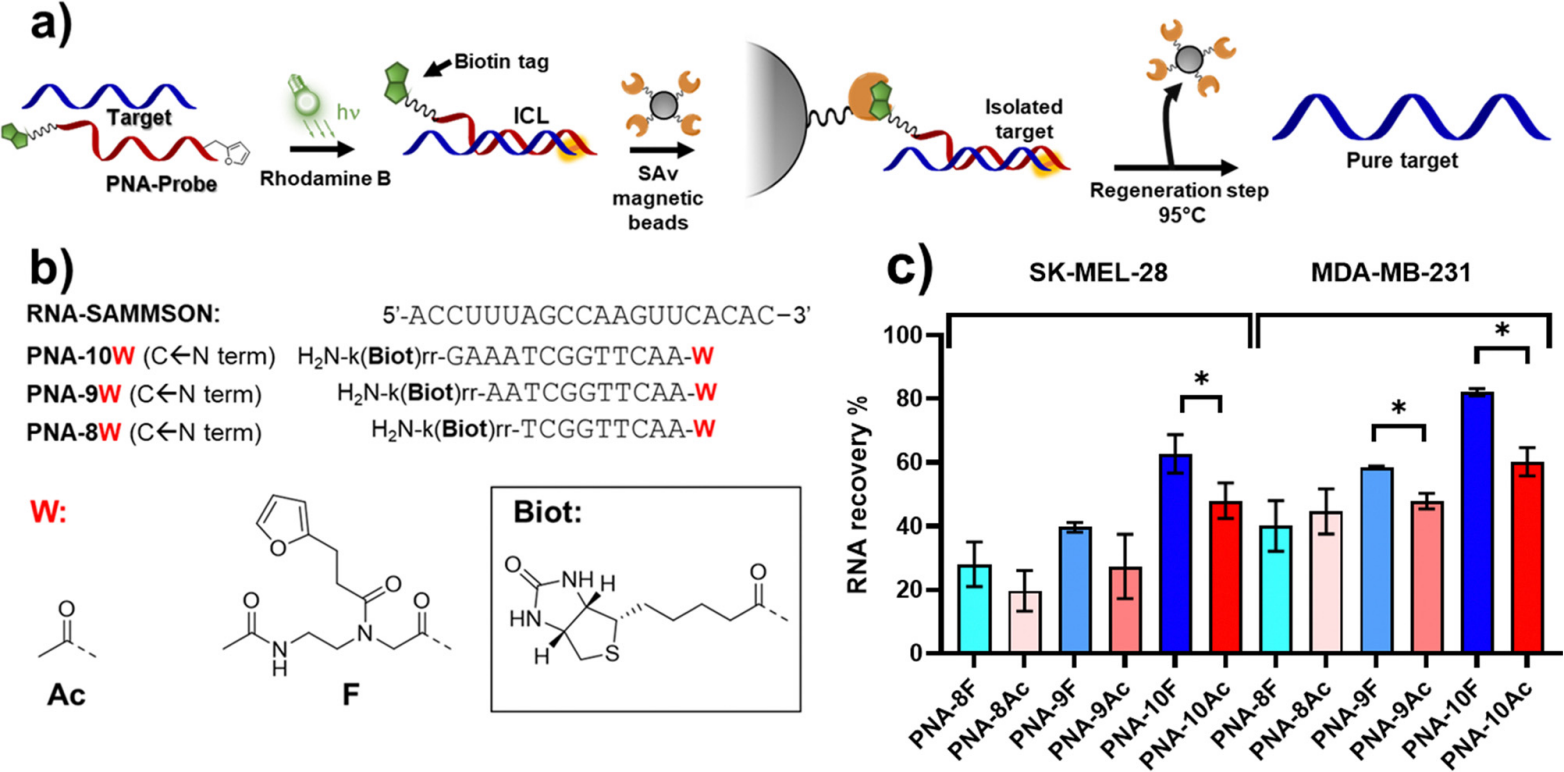
Biotin-mediated RNA pull-down. (a) Schematic representation of the pull-down experiment proposed in this work. (b) Design of the sequences used in the pool-down approach. (c) Overview of the pull-down experiment results, performed in PBS buffer (pH 7.4) supplemented with cell lysate (SK-MEL-28 or MDA-MB-231 cell lines), at a final strands concentration of 5 μM. * indicates *p* < 0.05. For the furan-containing probes, the experiments were performed at 5 μM RhoB concentration. The sequence and probe design are reported in the left panel of the figure. *k* = l-lysine; r = l-arginine. All experiments shown in the figures were performed in triplicate.

Finally, we set out to apply the reaction to a proof-of-concept pull-down experiment (Fig. 9a), employing PNA probes equipped with a biotin tag to enable streptavidin-mediated recovery of the RNA target. The use of PNA as compared to regular DNA derivatives is advantageous given the higher stability of PNA:DNA duplexes of similar length, which are less influenced by the sample composition (*e.g.* ionic strength, salts). In addition, PNA probes feature higher resistance toward enzymatic and chemical degradation and, therefore, are more stable in the biological environment in which the assay is performed.^9,45^

As a target, we focus our attention on the long non-coding RNA SAMMSON (Survival Associated Mitochondrial Melanoma Specific Oncogene RNA), found overexpressed in more than 90% of melanoma cells, and associated with increased mitochondrial localization of p32 protein levels.^46^ Secondary structure prediction of SAMMSON using the RNAfold webserver highlighted a single-stranded region featuring a cytosine-adenine repeat toward the 3′ end of the target, which is desirable in view of the high ICL% that is obtained when either cytosine or adenine residues were present on the targeted strand (Fig. 9b for sequences of probes and target).

Since a decrease in ICL% was observed when moving from PBS to cell-lysate, in order to ensure PNA:RNA complex stability in the final setup, PNA probes of different length (13, 11, and 9mer, see Fig. 9b or probes sequences) were synthesized, to understand the influence of the duplex stability on RNA pull-down. The sequences were equipped on their C-terminus with a biotin tag through the functionalization of a lysine side chain, to enable streptavidin pull-down. M1 was appended to the N-terminus, maintaining a similar geometry as in PNA-5. Homologous probes containing an acetylated N-terminus were also synthesized as control, illustrating the benefits of the ICL reaction.

After ICL formation, samples were mixed and equilibrated for 30 minutes with an equal volume of a solution containing the streptavidin-coated magnetic beads (10 mg mL^−1^), suspended in 2× Binding and Washing buffer (Tris–HCl 5 mM, pH 7.5, 1 M NaCl, 0.5 mM EDTA), followed by a magnetic decantation step (isolation), a washing step (with 1× Binding and Washing buffer) and a final heating step (95 °C for 1 h) to induce the release of the probes from the beads and revert the ICL.

Initially, we performed the ICL reaction using MB as a photosensitizer to activate the furan probes, under the same conditions as the previous optimization experiments. In this setup, the furan-containing probes performed worse as compared to pull-down performed with acetylated probes in absence of the light irradiation step. This was attributed to the ^1^O_2_-induced oxidation of the biotin included on the PNA probes (please refer to Fig. S21 in ESI†). The possible generation of biotin sulfoxide species is a known phenomenon that can lower the binding to streptavidin, thus hampering the final RNA recovery.^47^

To reduce oxidative biotin damage of the PNA probes, we changed PS from MB to Rhodamine B (RhoB), whose lower ^1^O_2_ production enables activation of the furan moiety with minor oxidation of biotin, as previously demonstrated.^25^ To ensure quantitative activation of the furan moiety, the PS concentration was increased to 5 μM, and the irradiation time was prolonged to 1 h (data not shown). The results obtained revealed that the RNA recovery is dependent on the probe length (Fig. 9c), and thus related to the ability of the PNA probe to form a stable complex with the target at the moment of furan activation. The RNA recovery was higher in presence of the longer 13-mer probes, PNA-10F and PNA-10Ac. Here, the presence of the alkylating furan warhead showed a statistically significant enhanced RNA recovery as compared to the acetylated probe in both SK-MEL-28 and MDA-MB-231 cell lines (Fig. 9c). Interestingly, in the case of MDA-MB cell lysate, a significantly enhanced recovery can also be observed when using shorter 11-mer probes.

## Conclusions

In conclusion, we have presented a structure–activity relationship study on the crosslink reactivity of furan-containing monomers inserted in both terminal and internal positions of PNA-probes. The results obtained demonstrate how probes equipped with an internal modification potentially allow reaching high levels of selectivity when maintaining the ability to hybridize to a complementary nucleobase (more specifically in the case of monomer M2), narrowing the number of ICL reactions to just a few permutations (1 or 3 out of 64 possible permutations). On the other hand, the use of universal nucleobases (M1 and M4) allows increasing the ICL yield for a larger number of permutations, desirable when a higher crosslinking activity of the probes is required. The ICL reaction was demonstrated to work in biologically relevant conditions, such as in a cell lysate, towards both DNA and RNA oligonucleotide targets, thus rendering the procedure suitable for the sequence-specific alkylation of intracellular targets. Moreover, we here showed for the first time how the furan-mediated ICL reaction can be reverted by simply heating the crosslinked product under conditions commonly used for *in vitro* assays, such as the biotin-mediated pulldown of targets. The pull-down experiment, performed on a SAMMSON-derived RNA target, showed an increased recovery of the target, as compared to probes of similar length not equipped with a furan warhead. As for pull-down approaches, the use of longer probes appears beneficial to ensure a higher recovery, as the higher stability of the PNA:RNA interaction ensures the formation of the duplex under more challenging conditions. In addition, the application of a reversible ICL reaction in this study illustrates the beneficial effect that crosslinking probes can have on target pull-down approaches, and can be extended to other types of stimuli-triggered reversible interactions, such as 3-cyanovinylcarbazole (CNV) chemistry.^48,49^ Indeed, CNV chemistry was recently applied to the target identification of miR-29b targets, but the possibility of reverting the formed crosslink after pull-down was not explored.^50^ Moreover, CNV is reported to selectively crosslink to thymine, and, secondly, to cytosine placed at the *N* + 1 position on the target strand.^51^ We here reported an approach that has a greater tolerance, given its ability to target both cytosine and adenine in good yields and guanine to a minor extent, placed at the 3′-flanking region of the RNA target. Thanks to the exact structure nature of M1, the furan warhead could alternatively be included at the C-terminus of the PNA probe for crosslinking to nucleobases placed in the 5′-flanking sequence, thus further expanding the range of possible sequences that could be targeted with this approach. Further studies are however required to evaluate the reactivity of the system in that position.

## Experimental procedures

### Crosslink experiments, general protocol for NBS activation

In a 1.5 mL Eppendorf tube, a 100 μL solution containing both PNA and DNA probes at 5 μM in PBS buffer (100 mM NaCl, 10 mM phosphates) pH 7.4 was prepared. This solution was allowed to equilibrate for 15 minutes at 25 °C before the start of the experiment. 2 μL of freshly prepared 250 μM NBS solution in mQ (1 eq.) were added. This was repeated every 15 minutes until 4 equivalents of NBS were added. Reaction outcome was monitored *via* PAGE and HPLC.

### Crosslink experiments, general protocol for ^1^O_2_ activation

100 μM working solution of PS was freshly prepared from a 1 mM stock solution. In a 1.5 mL Eppendorf tube, a 100 μL solution containing both PNA and DNA probes at 5 μM in PBS buffer (100 mM NaCl, 10 mM phosphates) pH 7.4 was prepared. This solution was allowed to equilibrate for 15 minutes at 25 °C before the addition of the PS, at a final concentration of 2 μM (MB) or 5 μM (RhoB). In case of cell-lysate experiments, the samples were additionally supplemented with cell lysate of SK-MEL-28 or MDA-MB-231 cells. The final cell concentration was 55 million cells per mL for SK-MEL-28 and 4 million cells per mL for MDA-MB-231 cells. The lamp (100 W halogen lamp LE.5210 Euromex EK-1 illuminator, equipped with Euromex LE.5214 dual arm light conductor) was placed on top of the Eppendorf tube for the entire duration of the experiment (20 minutes). Reaction outcome was monitored *via* PAGE and HPLC. The intensity of the lamps was set before starting the irradiation, using a TES 1335 luxmeter equipped with a custom fitting for the lamp bulbs to maintain the light intensity constant and similar for each irradiated sample.

### ICL% calculation

For each chromatogram, the peak corresponding to the DNA starting material and the DNA–PNA adduct were integrated, and the ICL% was calculated following the formula:1
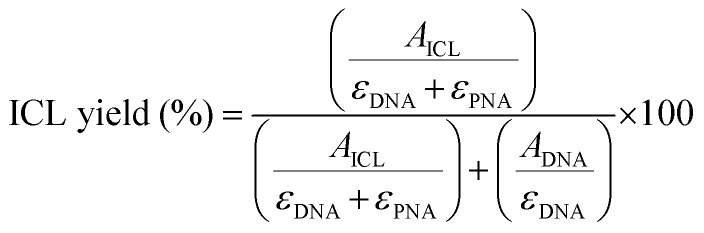
where *A*_ICL_ is the integral of the ICL product, *ε*_DNA+PNA_ is the calculated molar extinction coefficient of the ICL product, *A*_DNA_ indicates the integral of the DNA residual peak, and *ε*_DNA_ is the molar extinction coefficient of the DNA target.

### SAMMSON RNA pull-down

Prior to each experiment, Invitrogen^TM^ Dynabeads^TM^ MyOne^TM^ C1 magnetic beads (10 mg mL^−1^) were vortexed for 30 seconds and transferred to a 1.5 mL Eppendorf tube. Following the manufacturer's protocol, beads were washed 3 times with 1× Binding and Washing buffer (Tris–HCl 5 mM, pH 7.5, 1 M NaCl, 0.5 mM EDTA) and resuspended in 30 μL of 2× Binding and Washing buffer. In a typical pull-down experiment, 30 μL of crosslinked or hybridized PNA:RNA sample, at 5 μM strand concentration in cell lysate (SK-MEL-28, 55 million cells per mL), were mixed with an equal volume of resuspended beads (5 mg mL^−1^). The beads were equilibrated with the sample for 30 minutes, after which they were washed 5 times with 1 mL of Binding and Washing buffer and resuspended in 30 μL of Milli-Q water. The release of the RNA was achieved by incubating the beads at 95 °C for 1 hour in an Eppendorf Thermomixer. The obtained solution was analyzed by HPLC-UV.

## Author contributions

Al. M. conceived the presented idea and planned the experiments. F. P. performed the synthesis of the PNA monomers. F. P., E. C., and P. M. performed the PNA synthesis, the crosslink experiments and processed the data. E. C. and J. E. performed the pulldown experiments. Al. M. and E. C. wrote the manuscript. An. M and Al. M. supervised the project and provided financial support. All authors discussed the results and commented on the manuscript.

## Conflicts of interest

There are no conflicts to declare.

## Supplementary Material

CB-003-D2CB00095D-s001
