# Supplementary material for: Synthesis and structure–activity relationship of peptide nucleic acid probes with improved interstrand-crosslinking abilities: application to biotin-mediated RNA-pulldown
Source: RSC Chem Biol. 2022 Jul 21;3(9):1129–43. doi: 10.1039/d2cb00095d (PMC9428673; doi:10.1039/d2cb00095d)
Supplement: CB-003-D2CB00095D-s001 [file CB-003-D2CB00095D-s001.pdf]

Electronic Supplementary Material (ESI) for RSC Chemical Biology.
This journal is © The Royal Society of Chemistry 2022

Synthesis and structure-activity relationship of Peptide Nucleic Acid Probes with improved Interstrand-Crosslinking abilities: Application to Biotin-mediated RNA-Pulldown

Enrico Cadoni,[1‡] Francesca Pennati,[1‡] Penthip Muangkaew,[1] Joke Elskens,[1] Annemieke Madder,[1] and Alex Manicardi[1,2*]

[1] Organic and Biomimetic Chemistry Research Group, Department of Organic and Macromolecular Chemistry, Ghent University, Krijgslaan 281-7, 9000 Gent, Belgium

[2] Current address: Department of Chemistry, Life Sciences and Environmental Sustainability, University of Parma, Parco area delle Scienze, 17/A, 43124 Parma, Italy

1. General 2
2. Monomers synthesis 3
Calculation of the molar extinction coefficient for S14. 11
3. PNA synthesis 12
4. ICL experiments 15
PAGE experiments 18
5. Representative MALDI spectra 23
6. ICL reversibility 27
7. Pull-down experiments 29
8. Supporting References 33
9. NMR spectra 34
10. HPLC-MS chromatograms of pure PNAs 52
11. Representative HPLC traces of crosslink experiments 79

1. General

All reagents were purchased from Sigma-Aldrich, Fluka, Merck, TCI Europe, Fluorochem and used without further purification. Dry DMF was stored over 4 Å molecular sieves. TLCs were run on Merck silica 60 on aluminum sheets. Column chromatography was performed as flash chromatography on Grace silica 60 (0.060-0.200 mm). DNA sequences were purchased from IDT (Leuven, Belgium).

3-(2-benzyl-1,3-dioxo-1,2,3,3a,7,7a-hexahydro-4H-4,7-epoxyisoindol-4-yl)propanoic acid[1] and tert-butyl 2-(5-bromo-2-oxo-4-(1H-1,2,4-triazol-1-yl)pyrimidin-1(2H)-yl)acetate were synthesized according to a procedure previously reported.[2]

NMR spectra were recorded on a Bruker Avance 300 or 400. δ values are expressed in ppm relatively either to $CDCl_3$ (7.29 ppm for proton and 76.9 ppm for carbon) or DMSO-d^6 (2.50 ppm for proton and 39.5 ppm for carbon). The following abbreviations are used to explain the multiplicities: s=singlet, d=doublet, t=triplet, q=quartet, m=multiplet, and br=broad.

HPLC-MS data were collected on an Agilent 1100 Series instrument equipped with a Phenomenex Kinetex C18 100 Å column (150 x 4.6 mm, 5 µm at 35 °C) connected to an ESMSD type VL mass detector (quadrupole ion trap mass spectrometer) with a flow rate of 1.5 mL/min was used with the following solvent systems: (A): 0.1% HCOOH in H_2O and (B) MeCN. Gradient: 100% A for 2 min, then a gradient from 0 to 100% B over 6 min was used, followed by 2 min of flushing with 100% B (further referred to as HPLC1 conditions) or 100% A for 0.5 min, a gradient from 0 to 10% B over 0.1 min and then from 10 % to 30 % B over 7.7 minutes was used, followed by 2 min of flushing with 100% B (further referred to as HPLC2 conditions).

HPLC-UV data were collected on an Agilent 1100 Series instrument equipped with a Waters XTERRA RP18 5µm column (250 x 2.1 mm at 40°C or 50°C) connected to a DAD using a flow rate of 0.35 mL/min with the following solvent systems: (A): 0.1% TFA in H_2O and (B) 0.1% TFA in MeCN. Gradient: 100% A for 1 min, then a gradient from 0 to 10% B in 1 min, then to 30% B in 10 min, and finally to 100% B in 1 min, followed by 3.5 min of flushing with 100% B (further referred to as HPLC3 conditions). PNA oligomers were purified using a Luna C18(2) (5 µm, 100 Å, 250x10 mm) (further referred to as HPLC4 conditions: 100% A for 5 min, then a gradient from 0 to 50% B over 30 min at a flow rate of 4.0 mL/min).

UV-VIS spectra were recorded using a Trinean DropSense96 UV/VIS droplet reader.

MALDI-TOF analysis was performed using an Applied Biosystems - 4800 Plus MALDI TOF/TOF™ Analyzer. As matrix, 100mg/mL 2,5-Dihydroxybenzoic acid (DHB) in mQ water : MeCN (1:2) + 0.1% TFA was used.

PAGE analysis was performed using CBS Scientific QNC-700 Quadra Mini-Vertical Combo System, connected to a Consort 202 EV power supply. Gels were prepared into Novex 8x8 cm Empty Cassettes (1.0 mm). Temperature control was ensured by a JULABO-F12 refrigerated circulator connected to the QNC.

Crosslinking experiments were performed using Eppendorf Thermomixer Comfort for temperature control and Euromex Illuminator EK-1 lamps (100W halogen lamp LE.5210), equipped with Euromex LE.5214 dual arm light conductor. Power of the lamps was measured using a TES 1335 light meter equipped with a custom fitting for the lamp bulbs.

3-(2-benzyl-1,3-dioxo-1,2,3,3a,7,7a-hexahydro-4H-4,7-epoxyisoindol-4-yl)propanoic acid[1] and tert-butyl 2-(5-bromo-2-oxo-4-(1H-1,2,4-triazol-1-yl)pyrimidin-1(2H)-yl)acetate[2] were prepared according to previous literature reports.

2. Monomers synthesis

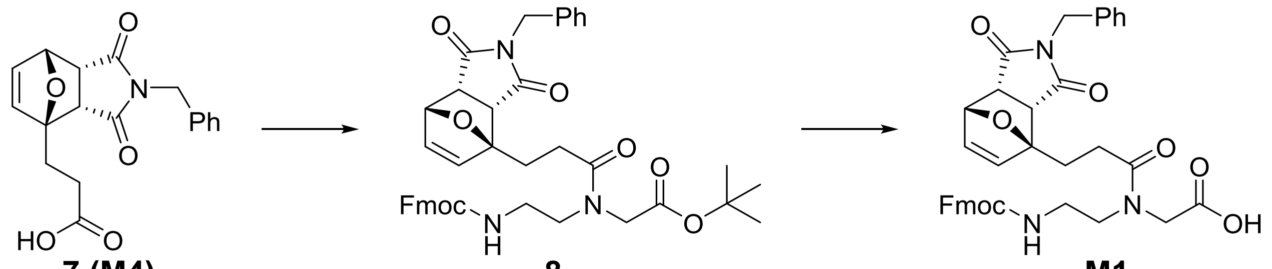

Scheme S1: overview of **M1** synthetic pathway.

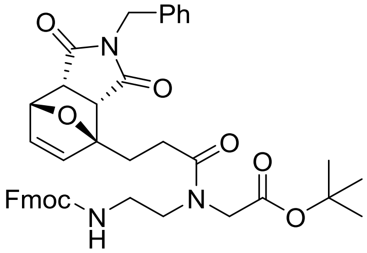

Synthesis of Fmoc-PNA-F(DA)-OtBu [8]: under Ar atmosphere 3-(2-benzyl-1,3-dioxo-1,2,3,3a,7,7a-hexahydro-4H-4,7-epoxyisoindol-4-yl)propanoic acid (293.72 mg, 0.897 mmol, 1.4 eq.), EDC HCl (171.965 mg, 0.597 mmol, 1.4 eq.) and DhBtOH (146.328 mg, 0.597 mmol, 1.4 eq.) are dissolved in 3 mL of dry DMF at 0°C. Then, DIPEA (0.254 mL, 1.538 mmol, 2.4 eq.) is added to the mixture and allowed to react 30' at 0°C before the addiction of the N-Fmoc-tert-butyl(2-aminoethyl)glycinate (277.52 mg, 0.641 mmol, 1 eq.). After 2 hours the mixture is diluted with 100 mL of AcOEt and washed with saturated $KHSO_4$ (2x 100 mL), saturated $NaHCO_3$ (2x 100 mL) and brine (100 mL). The organic phase is dried over Na_2SO_4, filtered, and the solvent is removed under reduced pressure. The crude is purified through flash chromatographic (from AcOEt/Hexane 3:7 to AcOEt) to obtain **8** as a white foamy solid (335.7 mg, 74.2%). TLC (AcOEt): Rf: 0.32; 1H-NMR (400 MHz, DMSO-d6) δ (ppm, major rotamer): 7.88 (d, J = 7.4 Hz, 2H), 7.66 (t, J = 7.8 Hz, 2H), 7.41 (t, J = 7.1 Hz, 2H), 7.36 – 7.16 (m, 8H), 6.55 (dd, J= 5.7 Hz, 1.8 Hz, 1H), 6.46 (d, J = 5.7 Hz, 1H), 5.08 (dd, J = 3.4, 1.8 Hz, 1H), 4.53 (s, 2H), 4.29 (d, J = 5.9 Hz, 2H), 4.23 – 4.16 (m, 1H), 3.89 (s, 2H), 3.35 (d, J = 5.6 Hz, 2H), 3.2 - 3.05 (m, 3H), 2.96 – 2.89 (m, 1H), 2.46 – 2.05 (m, 4H), 1.39 (d, J = 6.1 Hz, 9H); **^{13}C-NMR** (101 MHz, DMSO-d_6) δ(ppm): 176.1, 174.8, 171.8, 168.5, 156.2, 143.9, 140.7, 138.3, 137.1, 135.9, 128.4, 127.6, 127.2, 127.0, 126.8, 125.0, 120.1, 90.6, 80.6, 80.0, 65.4, 65.3, 62.6, 59.7, 50.3, 50.3, 48.8, 48.4, 46.7, 41.2, 27.7; **LC-MS** (MeOH, HPLC2) Rt: 2.1 min; *m/z* calcd for $C_{41}H_{43}N_3O_8$ [M]: 705,30502, found: 706.2 $[M+H]^+$, 650.1 $[M\text{-}tBu+H]^+$; **HR-MS** (ESI, MeOH) m/z found 706.3122 $[C_{41}H_{44}N_3O_8]^+$.

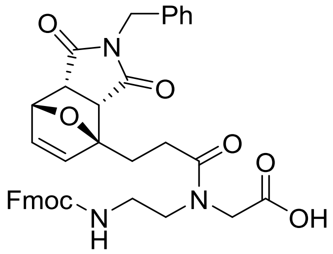

Synthesis of Fmoc-PNA-F(DA)-OH [M1]: in a 25 mL round bottom flask **8** (318.4 mg, 0.451 mmol, 1 eq.) is solubilized in 3 mL of DCM and the temperature is lowered to 0°C before the addiction of 1 mL of TFA. The temperature is kept at 0°C for 30', then raised to r.t. for 6h. The reaction is then diluted with MeOH and the solvent is removed under reduced pressure. Traces of TFA are removed by co-evaporation with $CHCl_3$ to give **M1** as a pink foamy solid (317.9 mg, quantitative yield). **TLC** (AcOEt): Rf: 0.27; **^{1}H-NMR** (400 MHz,

DMSO-d_6) δ (ppm, major rotamer): 12.88 (s, 1H), 7.88 (d, *J* = 7.4 Hz, 2H), 7.66 (t, *J* = 7.4 Hz, 2H), 7.41 (t, *J* = 7.4 Hz, 2H), 7.39 – 7.15 (m, 8H), 6.54 (dd, *J* = 5.7, 1.8 Hz, 1H), 6.46 (d, *J* = 5.7 Hz, 1H), 5.07 (d, *J* = 1.8 Hz, 1H), 4.53 (s, 2H), 4.27 (d, *J* = 2.3 Hz, 2H), 4.22 - 4.17 (m, 1H), 3.93 (d, *J* = 3.2 Hz, 2H), 3.44 – 3.28 (m, 2H), 3.16 (d, *J* = 5.9 Hz, 2H), 3.10 (d, *J* = 1.34 Hz, 1H), 2.93 (s, 1H), 2.70 – 2.28 (m, 2H), 2.23-2.01 (m, 2H); **^{13}C-NMR** (75 MHz, DMSO-d_6) δ(ppm): 184.4, 176.1, 174.7, 172.2, 171.7, 171.2, 170.8, 156.2, 143.8, 140.7, 138.3, 137.1, 135.9, 128.4, 127.6, 127.2, 127.0, 126.8, 125.0, 120.0, 90.6, 80.0, 65.3, 63.8, 62.5, 56.7, 50.2, 48.7, 47.5, 46.7, 46.4, 41.2; **LC-MS** (MeOH, HPLC2) Rt: 4.98 min; *m/z* calcd for $C_{37}H_{35}N_3O_8$ [M]: 649,24242, found: 648.1 [M-H]$^-$, 427.1 [M-Fmoc]$^-$, 426.1 [M-Fmoc-H]$^-$; **HR-MS** (ESI, MeOH) m/z found 650.2484 [$C_{37}H_{36}N_3O_8$]$^+$.

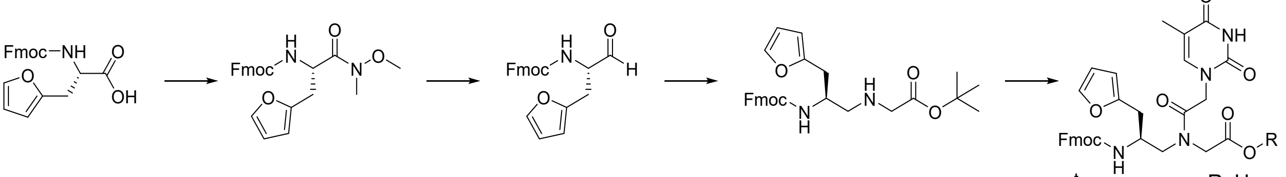

9 10 11 12 13 → M2

Scheme S2: overview of **M1** synthetic pathway.

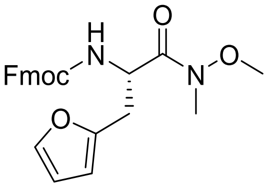

Synthesis of (S)-N-Fmoc-3-(furan-2-yl)alanin(N-methoxy-N-methyl)amide [10]: in a round bottom flask (S)-N-Fmoc-3-(furan-2-yl)alanine (1.026 g, 2.72 mmol, 1 eq.) is dissolved in 5 mL dry DMF. Then, the temperature is lowered to 0°C before the addiction of HBTU (1.134 g, 2.992 mmol, 1.1 eq) and DIPEA (1.17 mL, 7.072 mmol, 2.6 eq). The mixture is left to reacts for 15' at 0°C, the temperature is then raised to r.t. and left to react for further 15'. The mixture is cooled back to 0°C before the addition of N,O-dimethylhydroxylamine hydrochloride (398 mg, 4.08 mmol, 1.5 eq.) and left react for 5' before removing the ice bath and allowed to react at room temperature. After 2h the solvent is removed under reduced pressure, the crude is taken up with AcOEt (100 mL) and washed with saturated $KHSO_4$ (2x 100 mL), saturated $NaHCO_3$ (2x 100 mL), and brine (100 mL). The organic phase is then dried over Na_2SO_4 and the solvent is removed under reduced pressure to give **10** as a yellowish foam (815,4 mg, 71.4%). **TLC** (AcOEt/AcOH 100:1) Rf: 0.67; **^{1}H-NMR** (300 MHz, CDCl3) δ(ppm): 7.76 (d, *J* = 7.6 Hz, 2H), 7.59 (dd, *J* = 6.4, 4.4 Hz, 2H), 7.40 (t, *J* = 7.5 Hz, 2H), 7.34 – 7.28 (m, 3H), 6.29 (dd, *J* = 3.2, 1.9 Hz, 1H), 6.11 (d, *J* = 3.2 Hz, 1H), 5.63 (d, *J* = 8.9 Hz, 1H), 5.02 (d, *J* = 8.0 Hz, 1H), 4.39 (dd, *J* = 10.3, 7.3 Hz, 1H), 4.33 - 4.18 (m, 2H), 3.75 (s, 3H), 3.22 (s, 3H), 3.14 (dd, *J* = 15.0, 5.6 Hz, 1H), 3.05 (dd, *J* = 15.0, 6.8 Hz, 1H); **^{13}C-NMR** (75 MHz, CDCl3) δ(ppm): 155.8, 150.6, 144.0, 144.0, 142.2, 141.4, 127.8, 127.2, 125.30, 120.1, 110.5, 107.9, 67.2, 61.9, 50.6, 47.3, 32.4, 31.3; **LC-MS (MeOH,** HPLC2) Rt: 6.6 min; *m/z* calcd for $C_{24}H_{24}N_2O_5$ [M]: 420.16852, found: 421.1 [M+H]$^+$, 199.1 [M-Fmoc+H]$^+$, 183.1 [M-Fmoc-NH_3+H]; **HR-MS (ESI, MeOH)** m/z found 421.1765 [$C_{24}H_{25}N_2O_5$]$^+$.

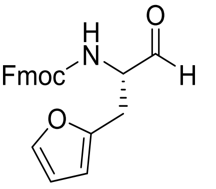

Synthesis of (S)-N-Fmoc-3-(furan-2-yl)alanal [11]: in a 2-neck flask flushed with Ar, **10** (757.8 mg, 1.804 mmol, 1eq) is dissolved in 5 mL of dry THF and cooled down to 0°C. In a separate round bottom flask $LiAlH_4$ (75.308 mg, 1.9844 mmol, 1.1 eq) is dissolved in 4 mL THF and dropwise via cannulation to the first flask.

After 20' the reaction is quenched with 4 mL of saturated $KHSO_4$ and the volatile part is removed under reduced pressure. The resulting oil is partitioned between 50 mL of AcOEt and 50 mL of saturated $KHSO_4$. The organic phase is then washed with saturated $KHSO_4$ (50 mL) and brine (50 mL). The organic phase is dried over Na_2SO_4 and the solvent is removed under reduced pressure to give **11** as a yellowish powder (564,5 mg, 86.6%). **TLC** ($CHCl_3$/MeOH 98:2) Rf: 0.4; **^{1}H-NMR** (300 MHz, $CDCl_3$) δ(ppm): 9.67 (s, 1H), 7.77 (d, *J* = 7.5 Hz, 2H), 7.58 (d, *J* = 7.9 Hz, 2H), 7.45 – 7.37 (m, 2H), 7.33 (t, *J* = 7.5, 1.2 Hz, 2H), 7.29 (d *J* = 1.3 Hz, 1H), 6.29 (d, *J* = 2.6 Hz, 1H), 6.11 (d, *J* = 3.2 Hz, 1H), 5.46 (d, *J* = 7.0 Hz, 1H), 4.52 (t, *J* = 6.3 Hz, 1H), 4.43 (t, *J* = 7.2 Hz, 2H), 4.23 (t, *J* = 6.9 Hz, 1H), 3.25 (d, *J* = 5.9 Hz, 2H); **^{13}C-NMR** (75 MHz, $CDCl_3$) δ(ppm): 156.0, 149.9, 143.8, 142.4, 141.6, 141.5, 127.9, 127.2, 125.2, 120.2, 110.7, 108.5, 67.3, 59.4, 47.3, 28.1.

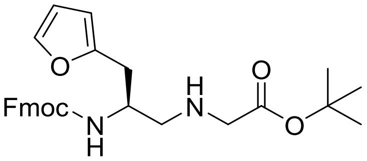

Synthesis of tert-butyl (S)-(2-(Fmoc-amino)-3-(furan-2-yl)propyl)glycinate [12]: in a round bottom flask **11** (127.6 mg, 0.353 mmol, 1 eq.) is dissolved in 10 mL of MeOH, then glycine t-butyl ester hydrochloride (118.4 mg, 706 µmol, 2 eq.) and DIPEA (0.123 mL, 706 µmol, 2 eq.) are added. After 15' $NaBH_3CN$ (44.3 mg, 706 µmol, 2 eq.) and AcOH (242 µL, 4.236 mol, 6 eq.) are added to the mixture. The pH of the reaction mixture is eventually adjusted to 4.5. After 80' the solvent is removed and the crude is taken up with 50 mL AcOEt and washed with saturated $NaHCO_3$ (3x 50 mL), and brine (50 mL). The organic phase is dried over Na_2SO_4 and the solvent is removed under reduced pressure. The crude is purified by flash chromatography (from DCM to DCM/MeOH 98:2) to give **12** as a yellowish sticky oil (137 mg, 81.4%). **TLC** ($CHCl_3$/MeOH 98:2) Rf: 0.1; **^{1}H-NMR** (300 MHz, $CDCl_3$) δ(ppm): 7.76 (d, *J* = 7.7 Hz, 2H), 7.60 (d, *J* = 8.4 Hz, 2H), 7.40 (d, *J* = 7.0 Hz, 2H), 7.32 (dd, *J* = 7.4, 1.3 Hz, 2H), 7.29 (d, *J* = 1.2 Hz, 1H), 6.29 (dd, *J* = 3.2, 1.9 Hz, 1H), 6.08 (s, 1H), 4.43 (dd, *J* = 7.1, 10.5 Hz, 2H), 4.22 (t, J= 4.22 Hz, 1H), 3.97 (s, 1H), 3.20 (m, 2H), 3.02 - 2.82 (m, 2H), 2.68 (s, 2H), 1.47 (s, 9H); **^{13}C-NMR** (75 MHz, $CDCl_3$) δ(ppm): 156.5, 152.5, 144.5, 144.5, 144.4, 142.1, 141.8, 141.8, 128.1, 128.1, 127.5, 127.5, 125.6, 125.6, 120.4, 120.4, 110.8, 107.8, 81.8, 67.1, 52.2, 52.1, 50.9, 47.8, 31.5, 28.8, 28.8, 28.8; **LC-MS** (MeOH, HPLC2) Rt: 7.159 min; *m/z* calcd for $C_{28}H_{32}N_2O_5$ [M]: 476.23112, found: 477.2 $[M+H]^+$, 421.1 $[M-^tBu+H]^+$; **HR-MS** (ESI, MeOH) m/z found 477.2400 $[C_{28}H_{33}N_2O_5]^+$.

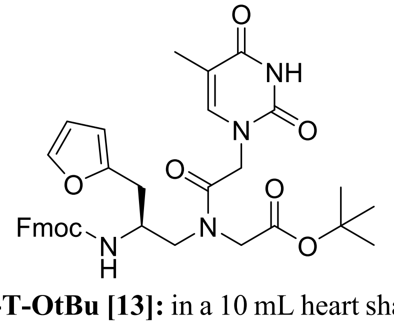

Synthesis of Fmoc-5L-FurAla-PNA-T-OtBu [13]: in a 10 mL heart shaped flask flushed with Ar, 2-(thymin-1-yl)acetic acid (536.669 mg, 2.86 mmol, 2 eq.) is solubilized in 3 mL of dry DMF. Then, DhBtOH (480.88 mg, 2.86 mmol, 2 eq) is added to the mixture and the temperature is lowered to 0°C with an ice bath before the addition of EDC·HCl (548.26 mg, 2.86 mmol, 2 eq.) and DIPEA (499 µL, 2.86 mmol, 2 eq.). The reaction is left at 0°C for 5' then further 5' at r.t. before the addiction of **12**. After 2h DMF is removed under reduced pressure. The crude is taken up with 50 mL of AcOEt and washed with saturated $KHSO_4$ (2x 50 mL), saturated $NaHCO_3$ (2x 50 mL), and brine (50 mL). The organic phase is dried over Na_2SO_4 and the solvent is removed under reduced pressure. The crude is purified by flash chromatography (from AcOEt/Hexane 6:4 to AcOEt/Hexane 8:2) to give **13** as a white solid (595,1 mg, 64.8%). **TLC** (AcOEt/Hexane 8:2) Rf: 0.36; (AcOEt/Hexane 7:3) Rf: 0.15; (AcOEt/Hexane 6:4) Rf: 0.1. **^{1}H-NMR** (300 MHz, $CDCl_3$) δ (ppm, major rotamer): 8.39 (s, 1H), 7.76 (d, *J* = 6.7 Hz, 2H), 7.56 (d, *J* = 7.4 Hz, 2H), 7.39 (t, *J* = 7.6 Hz, 2H), 7.34 – 7.27 (m, 2H), 7.26 (s, 1H), 6.94 (q, *J* = 1.2 Hz, 1H), 6.29 (s, 1H), 6.11 (s, 1H), 5.24 (d, *J* = 7.6 Hz, 1H), 4.68 – 4.44

(m, 2H), 4.43 – 4.24 (m, 1H), 4.19 (t, J = 7.0 Hz, 1H), 4.14 – 4.01 (m, 3H), 3.93 – 3.28 (m, 2H), 3.05 – 2.84 (m, 2H), 1.87 (dd, J = 5.9, 1.2 Hz, 3H), 1.49 (s, 9H).**^{13}C-NMR** (75 MHz, $CDCl_3$) δ (ppm, major rotamer): 168.6, 168.2, 164.0, 156.3, 151.0, 144.2, 144.0, 144.0, 142.0, 141.5, 141.4, 141.0, 127.9, 127.8, 127.3, 127.2, 125.3, 125.3, 120.2, 120.1, 110.8, 110.6, 107.8, 83.7, 66.8, 51.3, 50.8, 49.5, 47.7, 47.4, 30.6, 28.2, 28.2, 28,2; **LC-MS** (MeOH, HPLC2) Rt 6.51 min; *m/z* calcd for $C_{35}H_{38}N_4O_8$ [M]: 642,26896, found: 643.2 $[M+H]^+$, 365.1 $[M\text{-Fmoc-tBut}+H]^+$; **HR-MS** (ESI, MeOH) m/z found 665.2582 $[C_{35}H_{39}N_4O_8+Na]^+$.

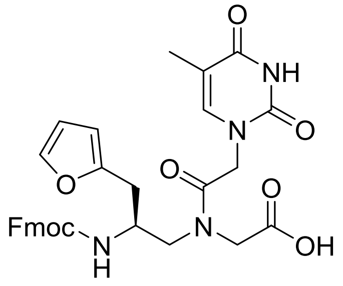

Synthesis of Fmoc-5L-FurAla-PNA-T-OH [M2]: in a 25 mL round bottom flask, **13** is solubilized in 4 mL $CHCl_3$ and the temperature is lowered to 0°C before the addition of 1 mL TFA. The temperature is maintained at 0°C for 30’, then raised to r.t. Additional 2 aliquots of 1 mL TFA were added every 2h. The colour of the reaction turns dark (olive green) over time. After 4h the reaction is diluted with 5 mL of MeOH and the solvent is removed under reduced pressure. Traces of TFA are removed by co-evaporation with $CHCl_3$. **M2** is obtained as a white foamy solid (519,2 mg, 97.3 %). **TLC** (AcOEt/Hexane 8:2) Rf: 0.24; **^{1}H-NMR** (300 MHz, DMSO-d_6) δ (ppm, major rotamer): 13.03 (s, 1H), 11.27 (d, J = 5.0 Hz, 1H), 7.88 (d, J = 7.5 Hz, 2H), 7.64 (t, J = 6.3 Hz, 2H), 7.49 (d, J = 9.0, 1.0 Hz, 2H), 7.41 (t, J = 7.5 Hz, 2H), 7.31 (t, J = 7.5 Hz, 1H), 7.23 (d, J = 1.3 Hz, 1H), 6.33 (ddd, J = 9.5, 3.2, 1.8 Hz, 1H), 6.10 (dd, J = 15.5, 3.5 Hz, 1H), 4.77 – 4.41 (m, 2H), 4.4 - 4.22 (m, 3H), 4.22 – 4.16 (m, 2H), 4.12 – 3.88 (m, 2H), 3.73 – 2.59 (m, 4H), 1.72 (d, J = 7.6, 1.1 Hz, 3H); **^{13}C-NMR** (75 MHz, DMSO-d_6) δ (ppm, major rotamer): 170.1, 167.5, 164.4, 155.7, 152.2, 151.0, 143.8 (two signals), 141.9, 141.7, 140.7, 127.6, 127.0, 125.2, 125.1, 120.1 (two signals), 110.4, 108.1, 106.7, 65.4, 50.7, 49.1, 47.8, 47.6, 46.7, 30.4, 11.9; **LC-MS** (MeOH, HPLC2) Rt: 6.01 min; m/z calcd for $C_{31}H_{30}N_4O_8$ [M]: 586,20636, found: 587.1 $[M+H]^+$, 365.1 $[M\text{-Fmoc}+H]^+$; **HR-MS** (ESI, MeOH) m/z found 587.2136 $[C_{31}H_{31}N_4O_8]^+$.

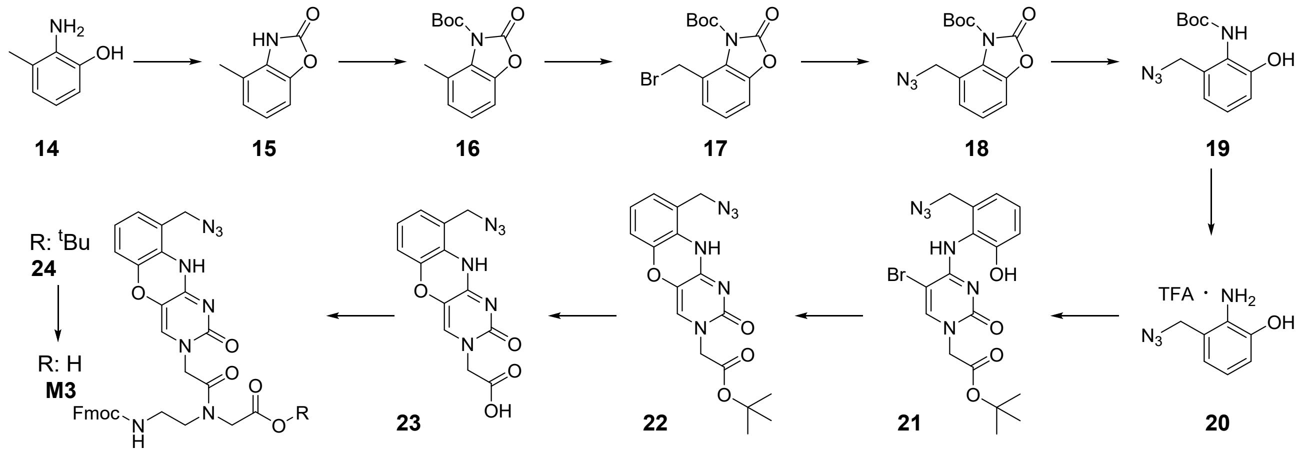

Scheme S3: overview of **M3** synthetic pathway.

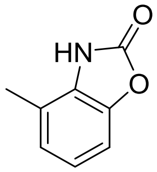

Synthesis of 4-methylbenzo[d]oxazol-2(3H)-one [15]: in a round bottom flask 2-amino-3-methylphenol (1 g, 8.12 mmol, 1 eq.) is solubilized in 10 mL of dry THF. CDI (1.98 g, 12.18 mmol, 1.5 eq.) is then added and the mixture is heated at reflux overnight. In the morning the reaction is diluted with AcOEt (40 mL) and washed with saturated $NaHCO_3$ (2x 50 mL), saturated $KHSO_4$ (2x 50 mL), and brine (50 mL). The organic phase is dried over Na_2SO_4, filtered and the solvent removed under reduced pressure to give **15** as yellowish solid (1,10 g, 91.1%). **TLC** (Hexane/AcOEt, 1:1): R_f: 0.49; **^{1}H-NMR** (300 MHz, DMSO-d_6) δ(ppm): 11.66 (s, 1H), 7.08 (dd, *J* = 6.3, 2.7 Hz, 1H), 6.97 (t, *J* = 7.8, 1H), 6.95 (dd, *J* = 7.8, 0.7 Hz, 1H), 2.29 (s, 3H); **^{13}C-NMR** (101 MHz, DMSO-d_6) δ(ppm): 154.6, 143.0, 129.3, 124.8, 121.6, 119.8, 106.8, 16.0; **LC-MS** (MeOH, HPLC2) Rt: 4.82 min; m/z calcd for $C_8H_7NO_2$ [M]: 149,04768, found: 150.1 $[M+H]^+$; **HR-MS** (ESI, MeOH) m/z found 150.0545 $[C_8H_8NO_2]^+$.

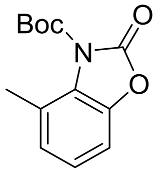

Synthesis of tert-butyl 4-methyl-2-oxobenzo[d]oxazole-3(2H)-carboxylate [16]: in a round bottom flask **15** (1.085 g, 7.279 mmol, 1 eq.) is solubilized in 10 mL dry THF. The temperature is lowered to 0°C before the addiction of TEA (1.015 mL, 7.279 mmol, 1 eq.). Di-*tert*-butyl dicarbonate (3.177 g, 14.559 mmol, 2 eq.) and DMAP (0.1779 g, 1.456 mmol, 0.2 eq.) are added and left stirring at r.t. overnight. In the morning the mixture is diluted with AcOEt (40 mL) and then washed with saturated $NaHCO_3$ (2x 50 mL), saturated $KHSO_4$ (2x 50 mL) and brine (50 mL). The organic phase is then dried over Na_2SO_4, filtered and the solvent removed under reduced pressure to give **16** as a pale orange oil (1,66 g, 91.5%). **TLC** (Hexane/AcOEt, 1:1): R_f: 0.62; **^{1}H-NMR** (300 MHz, DMSO-d_6) δ (ppm): 7.21 (m, 1H), 7.18 (t, *J* = 7.3 Hz, 1H), 7.09 (dd, *J* = 7.3, 0.9 Hz, 1H), 2.33 (s, 3H), 1.58 (s, 9H); **^{13}C-NMR** (101 MHz, DMSO-d_6) δ(ppm): 147.0, 142.2, 127.2, 126.0, 124.6, 123.9, 107.6, 85.7, 27.3, 19.2; **LC-MS** (MeOH, HPLC2) Rt: 6.71 min; m/z calcd for $C_{13}H_{15}NO_4$ [M]: 249,10011, found: 194.1 $[M-{}^tBu+H]^+$, 150.1 $[M-Boc+H]^+$.

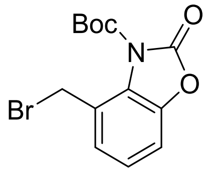

Synthesis of tert-butyl 4-(bromomethyl)-2-oxobenzo[d]oxazole-3(2H)-carboxylate [17]: in a round bottom flask **16** (800.0 mg, 3.212 mmol, 1 eq.) is dissolved in 15 mL of CCl_4. NBS (628.75 mg, 3.533 mmol, 1.1 eq.) and AIBN (52.74 mg, 0.321 mmol, 0.1 eq.) are then added and allowed to reflux overnight. In the morning the reaction is cooled to rt, diluted with CCl_4 and filtered under vacuum. The solvent is then removed, the residue is diluted with AcOEt (100 mL) and washed with saturated $NaHCO_3$ (2x 100 mL) and brine (100 mL). The organic phase is dried over Na_2SO_4, filtered, and the solvent removed under reduced pressure to give **17** as yellowish solid (1,02 mg, 97.2%). **TLC** (Hexane/AcOEt 1:1) Rf**:** 0.51; **^{1}H-NMR** (300 MHz, DMSO-d_6) δ(ppm): 7.41 (dd, *J* = 7.8, 1.5 Hz, 1H), 7.35 (dd, 1H), 7.27 (t, *J* = 7.9 Hz, 1H), 4.94 (s, 2H), 1.61 (s, 9H); **^{13}C-NMR** (75 MHz, DMSO-d_6) δ(ppm): 149.3, 147.2, 142.6, 126.4, 125.4, 124.9, 123.8, 110.7, 86.1, 59.7, 32.5, 27.4, 22.0; **LC-MS** (MeOH, HPLC2) Rt: 6.84 min; m/z calcd for $C_{13}H_{14}BrNO_4$ [M]: 327,01062, found: 271.9 $[M-{}^tBu+H]^+$, 227.9 $[M-Boc+H]^+$, 150.1 $[M-Br-Boc+H]^+$.

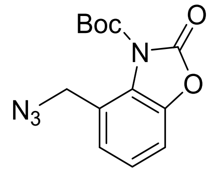

Synthesis of tert-butyl 4-(azidomethyl)-2-oxobenzo[d]oxazole-3(2H)-carboxylate [18]: in a round bottom flask **17** (1.021 mg, 3.123 mmol, 1 eq.) is dissolved in 10 mL of dry DMF. NaN_3 (223.32 mg, 3.435 mmol, 1.1 eq.) is added and the mixture left to react for 1h at r.t.. The reaction is diluted with AcOEt (90 mL), washed with water (2x 100 mL) and brine (100 mL). The organic phase is dried over Na_2SO_4, filtered and the solvent removed under reduced pressure to give an orangish oil (795.3 mg, 87.5%). The crude is purified through flash chromatography (from AcOEt/Hexane 1:9 to AcOEt/Hexane 3:7) to end up with **18** as a white solid (604.7 mg, 66.8%). **TLC** (Hexane/AcOEt 1:1) Rf: 0.67; **^{1}H-NMR** (300 MHz, DMSO-d_6) δ (ppm): 7.40 (dd, *J* = 7.1, 2.3 Hz, 1H), 7.31 (t, J = 7.8, 1H), 7.28 (dd, J = 7.8, 0.8 Hz, 1H), 4.73 (s, 2H), 1.59 (s, 9H); **^{13}C-NMR** (101 MHz, DMSO-d_6) δ(ppm): 149.5, 147.2, 142.5, 125.9, 125.4, 124.8, 122.0, 110.1, 85.9, 52.0, 27.4; **LC-MS** (MeOH, HPLC2) Rt: 5.01 min; m/z calcd for $C_{13}H_{14}N_4O_4$ [M]: 290,10150, found: 312.0 $[M+Na]^-$, 311.0 $[M+Na-H]^-$.

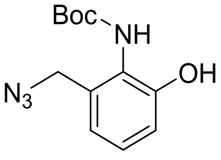

Synthesis of tert-butyl (2-hydroxy-6-methylphenyl)carbamate [19]: in a round bottom flask **18** (583.9 mg, 0.201 mmol, 1 eq.) is solubilized in 10 mL of MeOH. Then, K_2CO_3 (417.24 mg, 0.302 mmol, 1.5 eq.) is added and left to react for 2h at r.t.. The mixture is diluted with 100 mL of AcOEt, washed with saturated NH_4Cl (2x 100 mL) and brine (100 mL). The organic phase is dried over Na_2SO_4, filtered and the solvent removed to obtain **19** as a yellowish foamy solid (523.4 mg, 98.4%). **TLC** (Hexane/AcOEt 1:1) Rf: 0.53; **^{1}H-NMR** (400 MHz, DMSO-d_6) δ(ppm): 9.47 (s, 1H), 8.08 (s, 1H), 7.08 (t, *J* = 7.8 Hz, 1H), 6.84 (dd, *J* = 8.2, 1.5 Hz, 1H), 6.80 (dd, *J* = 7.6, 1.6 Hz, 1H), 4.32 (s, 2H), 1.42 (s, 9H); **^{13}C-NMR** (75 MHz, DMSO-d_6) δ(ppm): 154.1, 153.7, 134.3, 127.0, 123.8, 119.3, 115.8, 78.3, 50.5, 28.1; **LC-MS** (MeOH, HPLC2) Rt: 5.84 min; m/z calcd for $C_{12}H_{16}N_4O_3$ [M]: 264,12224, found: 181.1 $[M-^tBu+H]^+$, 137.1 $[M-Boc+H]^+$.

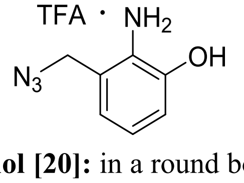

Synthesis of 2-amino-3-(azidomethyl)phenol [20]: in a round bottom flask **19** (500 mg, 1.893 mmol, 1 eq.) is solubilized in 6 mL $CHCl_3$, then TFA (2 mL) is added and the mixture is left react at r.t. for 4 hours with a colour change from yellow to salmon pink. The reaction is then diluted with 10 mL of MeOH and the solvent is removed. The remaining TFA is then co-evaporated with MeOH/$CHCl_3$ to obtain **20** as a reddish sticky solid (quantitative yield). **TLC** (Hexane/AcOEt 1:1) Rf: 0.48; **^{1}H-NMR** (400 MHz, DMSO-d_6) δ(ppm): 9.56 (s, 1H), 6.74 (dd, *J* = 7.8, 1.5 Hz, 1H), 6.66 (dd, *J* = 7.7, 1.6 Hz, 1H), 6.55 (t, *J* = 7.7 Hz, 1H), 4.38 (s, 2H); **^{13}C-NMR** (75 MHz, DMSO-d_6) δ(ppm): 145.0, 133.3, 121.0, 120.1, 117.2, 114.5, 50.6; **LC-MS** (MeOH, HPLC2) Rt: 5.73 min; m/z calcd for $C_7H_8N_4O$ [M]: 164,06981, found: 165.2 $[M+H]^+$.

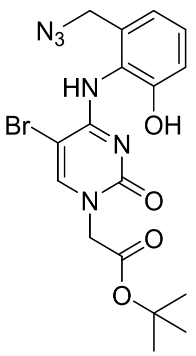

Synthesis of tert-butyl 2-(4-((2-(azidomethyl)-6-hydroxyphenyl)amino)-5-bromo-2-oxopyrimidin-1(2H)-yl)acetate [21]: in a round bottom flask **20** (98.68 mg, 0.277 mmol, 1 eq.) is solubilized in 2 mL of dry MeCN. Then, tert-butyl 2-(5-bromo-2-oxo-4-(1H-1,2,4-triazol-1-yl)pyrimidin-1(2H)-yl)acetate (50 mg, 0.305 mmol, 1.1 eq) is added to the mixture followed by DBU (45.53 µL, 0.305 mmol, 1.1 eq.). After the DBU addition the solution turns from orange to brown over time. After 6h the solvent is removed and the crude is taken up with 50 mL AcOEt, washed with saturated $KHSO_4$ (2x 50 mL) and brine (50 mL). The first aliquot of aqueous phase is reextracted with AcOEt (20 mL x3), washed with 60 mL of brine and then the merged organic phases are dried over Na_2SO_4 and the solvent is removed under reduced pressure to give **21** as an orange solid (116.9 mg, 93.5%). **TLC** (Hexane/AcOEt 1:1) Rf: 0.33; **^{1}H-NMR** (300 MHz, DMSO-d_6) δ (ppm): 9.63 (s, 1H), 8.44 (s, 1H), 8.16 (s, 1H), 7.19 (t, *J* = 7.8 Hz, 1H), 6.91 (dd, *J* = 8.2, 1.6 Hz, 1H), 6.88 (dd, *J* = 8.4, 1.6 Hz, 1H), 4.36 (s, 2H), 4.30 (s, 2H), 1.40 (s, 9H); **^{13}C-NMR** (75 MHz, DMSO-d_6) δ(ppm): 167.2, 166.4, 166.2, 153.9, 153.7, 147.0, 134.8, 128.0, 119.0, 116.1, 81.8, 81.6, 50.8, 50.3, 27.6; **LC-MS** (MeOH, HPLC2) Rt: 5.65 min; m/z calcd for $C_{17}H_{19}BrN_6O_4$ [M]: 450,06512, found: 453.0 $[M(^{81}Br)+H]^+$, 451.0 $[M+H]^+$; **HR-MS** (ESI, MeOH) m/z found 451.0722 $[C_{17}H_{20}BrN_6O_4]^+$ and 453.0703 $[C_{17}H_{20}{}^{81}BrN_6O_4]^+$.

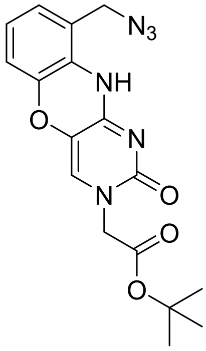

Synthesis of tert-butyl 2-(9-(azidomethyl)-2-oxo-2,10-dihydro-3H-benzo[b]pyrimido[4,5-e][1,4]oxazin-3-yl)acetate [22]: in a round bottom flask **21** (52.35 mg, 0.116 mmol, 1 eq.) is solubilized in 5 mL of absolute EtOH. Then potassium fluoride (67.396 mg, 1.16 mmol, 10 eq.) is added to the mixture and left to react over-weekend under reflux. Then the solvent is removed and the reddish crude is diluted with 50 mL AcOEt and washed with saturated $KHSO_4$ (2x 50 mL), saturated $NaHCO_3$ (2x 50 mL), and brine (50 mL). The organic phase is dried over Na_2SO_4, the solution is filtered, and the solvent is removed. The crude is purified by flash chromatography (from Hexane/AcOEt 6:4 to AcOEt) to give **22** as a yellowish powder (21.7 mg, 50.5%). **TLC** (Hexane/AcOEt 1:1) Rf: 0.44; **^{1}H-NMR** (300 MHz, $CDCl_3$) δ (ppm): 6.88 (t, *J* = 7.6 Hz, 1H), 6.83 (dd, *J* = 7.7, 2.1 Hz, 1H), 6.68 (dd, *J* = 7.4, 2.1 Hz, 1H), 6.37 (s, 1H), 4.37 (s, 2H), 4.23 (s, 2H), 1.49 (s, 9H); **^{13}C-NMR** (75 MHz, $CDCl_3$) δ (ppm): 166.8, 150.8, 150.5, 143.9, 129.6, 129.0, 127.9, 125.0, 124.5, 119.7, 115.2, 83.2, 50.3, 49.7, 29.7, 28.0; **LC-MS** (MeOH, HPLC2) Rt: 5.94 min; m/z calcd for $C_{17}H_{20}N_6O_4$ [M]: 370,13895, found: 371.11 $[M+H]^+$, 315.1 $[M-tBu+H]^+$; **HR-MS** (ESI, MeOH) m/z found 371.1459 $[C_{17}H_{21}N_6O_4]^+$.

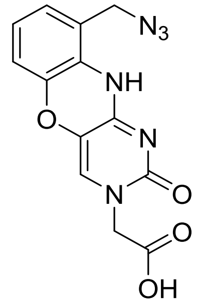

Synthesis of 2-(9-(azidomethyl)-2-oxo-2,10-dihydro-3H-benzo[b]pyrimido[4,5-e][1,4]oxazin-3-yl)acetic acid [23]: in a round bottom flask **22** (14.1 mg, 0.038 mmol, 1 eq.) is solubilized in 1 mL $CHCl_3$, then TFA (0.5 mL) is added and the mixture is left react at r.t. for 3 hours with a colour change from yellow to orange. The reaction is then diluted with 3 mL of MeOH and the solvent is removed. The remaining TFA is then co-evaporated with MeOH/$CHCl_3$ to give **23** as a yellow solid (quantitative yield). **ε**(H_2O, 378 nm): 6580 $M^{-1}cm^{-1}$; **ε**(H_2O, 260 nm): 6350 $M^{-1}cm^{-1}$; **^{1}H-NMR** (300 MHz, DMSO-d_6) δ (ppm): 7.07 (s, 1H), 6.91 (dd, J = 7.8, 5.3 Hz, 1H), 6.89 (t, J = 7.6 Hz, 1H), 6.75 (dd, J = 7.1, 2.4 Hz, 1H), 4.46 (s, 2H), 4.25 (s, 2H); **^{13}C-NMR** (101 MHz, DMSO-d_6) δ(ppm) 169.7, 143.5, 143.5, 143.5, 143.5, 127.2, 124.8, 124.3, 115.2, 114.8, 63.9, 48.7, 48.5; **LC-MS** (MeOH, HPLC2) Rt: 3.88 min; m/z calcd for $C_{13}H_{10}N_6O_4$ [M]: 314,07635, found: 313.0 $[M-H]^-$; **HR-MS** (ESI, MeOH) m/z found 313.0703 $[C_{13}H_9N_6O_4]^-$.

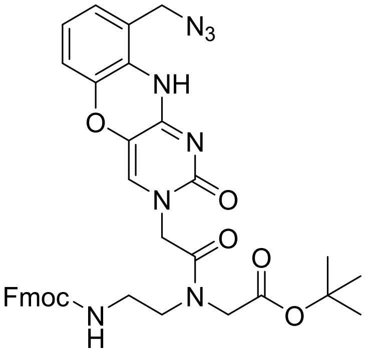

Synthesis of Fmoc-PNA-phenoxazine-N3-OtBu [24]: under Ar atmosphere **23** (100 mg, 0.424 mmol, 2 eq.), EDC·HCl (81.33 mg, 0.424 mmol, 2 eq.) and DhBtOH (69.18 mg, 0.424 mmol, 2 eq.) are dissolved in 1.5 mL of dry DMF at 0°C. Then, DIPEA (0.105 mL, 0.636 mmol, 3 eq.) is added to the mixture and allowed to react 10’ at 0°C before the addiction of the N-Fmoc-*tert*-butyl(2-aminoethyl)glycinate (91.84 mg, 0.212 mmol, 1 eq.). After 5 hours the solvent is removed and the crude is partitioned between 50 mL AcOEt and 50 mL saturated $KHSO_4$. The organic phase is washed with saturated $KHSO_4$ (50 mL), saturated $NaHCO_3$ (2x 50 mL), and brine (50 mL), the organic phase is then dried over Na_2SO_4 and the solvent is removed under reduce pressure. The crude is purified by flash chromatography (from AcOEt/Hexane 7:3 to AcOEt) to give **24** as a yellowish solid (104.4 mg, 71.1%). **TLC** (AcOEt) Rf: 0.38; **^{1}H-NMR** (400 MHz, $CDCl_3$) δ(ppm, major rotamer): 7.76 (t, J = 7.7 Hz, 2H), 7.61 (t, J = 5.7 Hz, 2H), 7.39 (q, J = 7.6 Hz, 2H), 7.35 – 7.28 (m, 2H), 6.86 (t, J=7.6 Hz, 1H), 6.83 – 6.78 (m, 1H), 6.66 (dd, J = 9.0 Hz, 1H), 6.39 (s, 1H), 6.01 (s, 1H), 4.46 (d, J = 6.8 Hz, 2H), 4.39 – 4.28 (m, 4H), 4.23 (d, J = 6.8 Hz, 1H), 3.95 (s, 2H), 3.60 – 3.48 (m, 2H), 3.39 (s, 2H), 1.50 (s, J = 9.5 Hz, 9H); **^{13}C-NMR** (75 MHz, $CDCl_3$) δ(ppm, major rotamer) 168.8, 168.7, 167.4, 156.7, 151.4, 151.2, 144.0, 143.8, 141.3, 128.6, 127.8, 127.1, 125.2, 124.7, 124.4, 120.0, 115.3, 83.6, 66.8, 50.4, 49.9, 49.0, 48.3, 47.2, 39.1, 28.0; **LC-MS** (MeOH, HPLC2) Rt: 6.91 min**;** m/z calcd for $C_{36}H_{36}N_8O_7$ [M]: 692,27070, found: 693.2 $[M+H]^+$. 694 **HR-MS** (ESI, MeOH) m/z found 693.2784 $[C_{36}H_{37}N_8O_7]^+$.

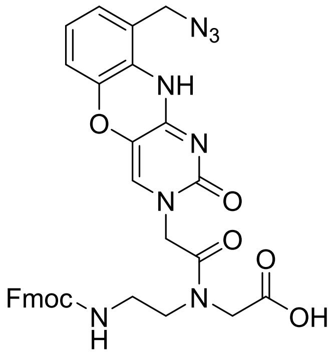

Synthesis of Fmoc-PNA-phenoxazine-N3-OH [M3]: in a 25 mL round bottom flask, **24** (97.5 mg, 0.14 mmol, 1 eq.) is solubilized in 2 mL $CHCl_3$ and the temperature is lowered to 0°C before the addition of 1 mL TFA. The temperature is then raised to r.t. and allowed to react for 4h. The colour of the reaction turns into orange/pink over time. As soon as the reaction is complete, it is diluted with 5 mL of MeOH and the solvent is removed under reduced pressure. Traces of TFA are removed by co-evaporation with $CHCl_3$. **M3** is obtained as a yellow solid (88.9 mg, quantitative yield). **^{1}H-NMR** (300 MHz, DMSO-d_6) δ(ppm, major rotamer): 11.85 (s, 1H), 7.93 – 7.83 (m, 2H), 7.73 – 7.63 (m, 2H), 7.41 (dt, J = 7.5, 1.8 Hz, 2H), 7.33 (dt, J = 7.3, 1.2 Hz, 2H), 6.96 – 6.81 (m, 2H), 6.70 (dd, J = 7.5, 2.1 Hz, 1H), 4.53 (s, 2H), 4.45 (d, J = 1.9 Hz, 2H), 4.39 – 4.15 (m, 4H), 3.99 (s, 2H), 3.44 – 3.29 (m, 2H), 3.25 (d, J = 6.0 Hz, 2H); **^{13}C-NMR** (75 MHz, DMSO-d_6) δ(ppm): 170.7, 170.4, 167.7, 167.3, 156.3, 143.8, 143.5, 140.7, 127.6, 127.0, 125.1, 124.8, 124.21, 120.1, 115.1, 114.6, 65.5, 48.5, 48.0, 46.7, 38.9; **LC-MS** (MeOH, HPLC2) Rt: 4.92 min**;** m/z calcd for $C_{32}H_{28}N_8O_7$ [M]: 636,20810, found: 637.1 $[M+H]^+$; **HR-MS** (ESI, MeOH) m/z found 637.2149 $[C_{32}H_{29}N_8O_7]^+$.

Calculation of the molar extinction coefficient for S14.

A stock solution of **23** was prepared in H_2O (0.99 mg in 1.0mL, 3.18 mM). From this solution an intermediate 1:10 dilution was prepared, which was then used to prepare the final solutions with a 1.5:100, 2:100, 2.5:100, 3:100, 3.5:100 dilution. Each solution was prepared in triplicate and the absorbance of the solutions were evaluated within the rangeof 200–600 nm at a scan speed of 600 nm/min.

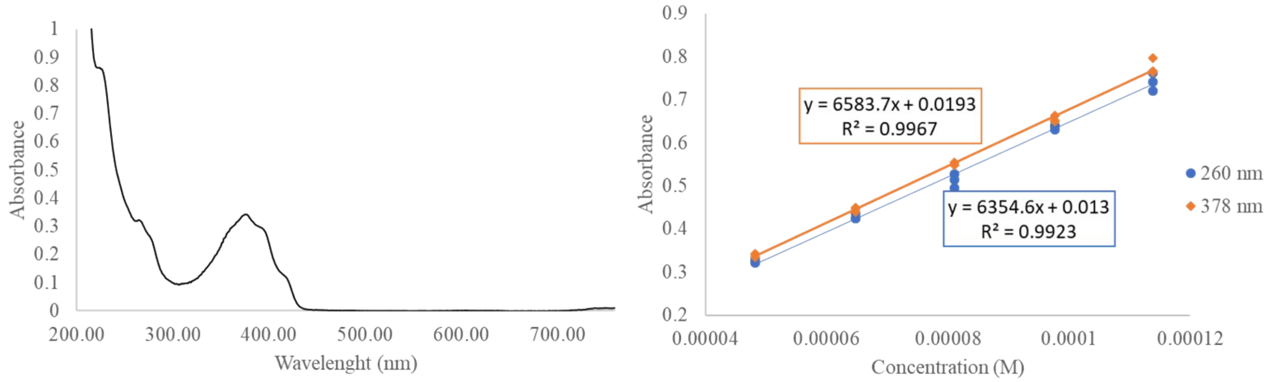

Figure S1: Representative UV spectrum of S14 (left) and linear regression for the evaluation of the extinction coefficient at 260 nm and 378 nm

3. PNA synthesis

The synthesis of the PNA probes was performed with standard manual Fmoc-based solid-phase synthesis using HBTU/DIPEA as coupling mixture and commercially available Fmoc-PNA-OH monomers (Biosearch Technologies, Scotland). Rinkamide-ChemMatrix resin was first loaded with Fmoc-Arg$_{(Pbf)}$-OH as first monomer (0.2 mmol/g). After monomer-specific manipulations (Table S1), the PNA probes were purified via RP-HPLC (HPLC4 conditions, *vide supra* general). The purity and identity of the PNAs were evaluated by LC-MS (HPLC1 conditions).

Table S1: list of monomer specific manipulations for the synthesis of the different PNA series. SPPS: solid phase peptide synthesis, DA: Diels-Alder; rDA: retro-Diels-Alder.

PNA	Protocol
**PNA-1 series**<br>**PNA-5**	1- SPPS with **M1**<br>2- Cleavage: 10% m-cresol in TFA<br>3- rDA (basic)
**PNA-2 series**<br>**PNA-6**	1- SPPS<br>2- Insertion of **M2**<br>3- On-resin DA<br>4- SPPS<br>5- Cleavage: 10% m-cresol, 10% thioanisole in TFA<br>6- rDA (acid)
**PNA-3 series**	1- SPPS with **M3**<br>2- Cleavage: 10% m-cresol in TFA<br>3- CuAAC reaction
**PNA-4 series**	1- SPPS with **M3**<br>2- Staudinger reduction<br>3- Coupling of **2**<br>4- Cleavage: 10% m-cresol in TFA<br>5- rDA (basic)
**PNA-7**	1- SPPS<br>2- Insertion of **M4**<br>3- Cleavage: 10% m-cresol in TFA<br>4- rDA (basic)

Retro Diels-Alder protocols: the crude PNA obtained after cleavage was solubilized in mQ water at a final PNA concentration of 1 mM (based on synthesis scale). If necessary, the pH of the solution can be increased from ~3 to 11.5 with carbonate buffer (add 10 µL of saturate sodium carbonate solution for each µmol of crude, then adjust with 1M NaOH). The solution was then aliquoted in 1.5 mL Eppendorf and submitted to the retro Diels-Alder temperature ramp: (A) pH ~3: from 25°C to 90°C in 30 minutes, then 90°C for 4.5h and finally to 15°C in 20 minutes; (B) pH ~11.5: from 25°C to 90°C in 30 minutes, then 90°C for 90 minutes and finally to 15°C in 20 minutes. In case of presence of PEG-based spacers, the retro-Diels-Alder has to be carried out in basic conditions to avoid ether bond hydrolysis.

On-resin Diels-Alder protocol: for a 5 µmol scale of resin-loaded PNA in a 500 µL Eppendorf tube, 200 µL of a 0.25 M solution of N-(N-Boc-2-amminoethyl)maleimide in DMF (10 eq.) were added and the mixture was heated at 90 °C for 5 h. The resin beads were then transferred to and empty SPE tube equipped with PE frit, and washed carefully with DMF and DCM. The resin was then submitted to the cleavage step.[3]

On-resin Staudinger reduction: for a 5 µmol scale, 10 minutes reductions with 1M $P(CH_3)_3$ in THF/THF/H_2O 1:2:3 solution, twice.

Click reaction protocol: different solutions were prepared: 200 mM solution of 2 in MeOH, 200 mM solution of copper sulfate in H_2O, 200 mM solution of sodium ascorbate in H_2O. Reaction was carried out with a final PNA concentration (from crude PNA) of 2 mM or 5 mM using a molar ratio alkyne/ascorbate/Cu(II) of 2:4:2. The mixture was then left to react for 2h before the purification.[4]

PNA-1A (Ac-GGGC-A1-GATC-rrr-NH_2): 9.2%; Rt = 3.09 min; ε = 96000 $M^{-1}cm^{-1}$; ESI-MS: calcd MW 3234.3 [M]: m/z found: 1078.9 [M+3H]$^{3+}$, 809.4 [M+4H]$^{4+}$, 647.8 [M+5H]$^{5+}$, 540.0 [M+6H]$^{6+}$, 463.0 [M+7H]$^{7+}$. **PNA-1C** (Ac-GGGC-C1-GATC-rrr-NH_2): 4.6%; Rt= 3.08 min; ε = 88900 $M^{-1}cm^{-1}$; ESI-MS: calcd MW 3210.2 [M]: m/z found: 1070.8 [M+3H]$^{3+}$, 803.4 [M+4H]$^{4+}$, 642.9 [M+5H]$^{5+}$, 536.0 [M+6H]$^{6+}$, 459.6 [M+7H]$^{7+}$. **PNA-1G** (Ac-GGGC-G1-GATC-rrr-NH_2): 5.1%; Rt = 3.1 min; ε = 94000 $M^{-1}cm^{-1}$; ESI-MS: calcd MW 3249.9 [M]: m/z found: 1084.2 [M+3H]$^{3+}$, 813.5 [M+4H]$^{4+}$, 651.0 [M+5H]$^{5+}$, 542.7 [M+6H]$^{6+}$, 465.3 [M+7H]$^{7+}$. **PNA-1T** (Ac-GGGC-T1-GATC-rrr-NH_2): 5.1%; Rt = 3.12 min; ε = 90900 $M^{-1}cm^{-1}$; ESI-MS: calcd MW 3224.9 [M]: m/z found: 1075.8 [M+3H]$^{3+}$, 807.2 [M+4H]$^{4+}$, 645.9 [M+5H]$^{5+}$, 538.5 [M+6H]$^{6+}$, 461.7 [M+7H]$^{7+}$. **PNA-2A** (Ac-GGGC-A2-GATC-rrr-NH_2): 0.6%; Rt = 3.12 min; ε = 104600 $M^{-1}cm^{-1}$; ESI-MS: calcd MW 3358.4 [M]: m/z found: 1120.1 [M+3H]$^{3+}$, 840.3 [M+4H]$^{4+}$, 672.5 [M+5H]$^{5+}$, 560.7 [M+6H]$^{6+}$, 480.7 [M+7H]$^{7+}$. **PNA-2C** (Ac-GGGC-C2-GATC-rrr-NH_2): 1.1%; HPLC2 Rt = 3.11 min; ε = 97500 $M^{-1}cm^{-1}$; ESI-MS: calcd MW 3374,4 [M]: m/z found: 1125.4 [M+3H]$^{3+}$, 844.3 [M+4H]^{4}+, 675.6 [M+5H]$^{5+}$, 563.3 [M+6H]$^{6+}$, 483.0 [M+7H]$^{7+}$. **PNA-2G** (Ac-GGGC-G2-GATC-rrr-NH_2): 0.7%; Rt = 3.1 min; ε = 102600 $M^{-1}cm^{-1}$; ESI-MS: calcd MW 3374.4 [M]: m/z found: 1125.4 [M+3H]$^{3+}$, 844.3 [M+4H]$^{4+}$, 675.6 [M+5H]$^{5+}$, 563.3 [M+6H]$^{6+}$, 483.0 [M+7H]$^{7+}$. **PNA-2T** (Ac-GGGC-T2-GATC-rrr-NH_2): 1.2%; Rt = 3.12 min; ε = 99500 $M^{-1}cm^{-1}$; ESI-MS: calcd MW 3349,3 [M]: m/z found: 1117.1 [M+3H]$^{3+}$, 838.0 [M+4H]$^{4+}$, 670.7; [M+5H]$^{5+}$, 559.0 [M+6H]$^{6+}$, 479.4 [M+7H]$^{7+}$. **PNA-3A** (Ac-GGGC-A3-GATC-rrr-NH_2): 2.2%; Rt = 3.22 min; ε = 102350 $M^{-1}cm^{-1}$; ESI-MS: calcd MW 3585.6 [M]: m/z found: 1195.8 [M+3H]$^{3+}$, 897.0 [M+4H]^{4}+, 717.9 [M+5H]$^{5+}$, 598.5 [M+6H]$^{6+}$, 513.2 [M+7H]$^{7+}$. **PNA-3C** (Ac-GGGC-C3-GATC-rrr-NH_2): 2.8%; Rt = 3.17 min; ε = 95250 $M^{-1}cm^{-1}$; ESI-MS: calcd MW 3561.6 [M]: m/z found: 1187.8 [M+3H]$^{3+}$, 891.2 [M+4H]$^{4+}$, 713.1 [M+5H]$^{5+}$, 594.5 [M+6H]$^{6+}$. **PNA-3G** (Ac-GGGC-G3-GATC-rrr-NH_2): 2.8%; Rt = 3.23 min; ε = 100350 $M^{-1}cm^{-1}$; ESI-MS: calcd MW 3601.6 [M]: m/z found: 1201.1 [M+3H]$^{3+}$, 901.0 [M+4H]$^{4+}$, 721.0 [M+5H]$^{5+}$, 601.0 [M+6H]$^{6+}$, 515.5 [M+7H]$^{7+}$. **PNA-3T** (Ac-GGGC-T3-GATC-rrr-NH_2): 1.7%; Rt = 3.32 min; ε = 97250 $M^{-1}cm^{-1}$; ESI-MS: calcd MW 3576.6 [M]: m/z found: 1192.7 [M+3H]$^{3+}$, 894.7 [M+4H]$^{4+}$, 716.1 [M+5H]$^{5+}$, 597.0 [M+6H]$^{6+}$, 511.8 [M+7H]$^{7+}$. **PNA-4A** (Ac-GGGC-A4-GATC-rrr-NH_2): 0.72%; Rt = 3.13 min; ε = 102350 $M^{-1}cm^{-1}$; ESI-MS: calcd MW 3502.5 [M]: m/z found: 1168.8 [M+3H]$^{3+}$, 876.9 [M+4H]$^{4+}$, 701.7 [M+5H]$^{5+}$, 585.0 [M+6H]$^{6+}$, 501.6 [M+7H]$^{7+}$. **PNA-4C** (Ac-GGGC-C4-GATC-rrr-NH_2): 0.10%; Rt = 3.09 min; ε = 95250 $M^{-1}cm^{-1}$; ESI-MS: calcd MW 3478.5 [M]: m/z found: 1160.7 [M+3H]$^{3+}$, 870.9 [M+4H]$^{4+}$, 696.9 [M+5H]$^{5+}$, 581.0 [M+6H]$^{6+}$. **PNA-4G** (Ac-GGGC-G4-GATC-rrr-NH_2): 0.79%; Rt = 3.16 min; ε = 100350 $M^{-1}cm^{-1}$; ESI-MS: calcd MW 3518.5 [M]: m/z found: 1174.1 [M+3H]$^{3+}$, 880.8 [M+4H]$^{4+}$, 704.8 [M+5H]$^{5+}$, 587.5 [M+6H]$^{6+}$, 503.9 [M+7H]$^{7+}$. **PNA-4T** (Ac-GGGC-T4-GATC-rrr-NH_2): 0.93%; Rt = 3.22 min; ε = 97250 $M^{-1}cm^{-1}$; ESI-MS: calcd MW 3493.5 [M]: m/z found: 1165.7 [M+3H]$^{3+}$, 874.5 [M+4H]$^{4+}$, 699.9 [M+5H]$^{5+}$, 583.3 [M+6H]$^{6+}$, 500.3 [M+7H]$^{7+}$. **PNA-5** (Ac-1-GGGCATGATCT-rrr-NH_2): 9.03 %; Rt = 2.91 min; ε = 113200 $M^{-1}cm^{-1}$; ESI-MS: calcd MW 3766.2 [M]: m/z found: 1256.4 [M+3H]$^{3+}$, 942.5 [M+4H]$^{4+}$, 754.2 [M+5H]$^{5+}$, 628.7 [M+6H]$^{6+}$, 539.1 [M+7H]$^{7+}$. **PNA-5T** (Ac-1-TGGGCATGATCT-rrr-NH_2): 5.01 %; Rt = 3.14min; ε = 121800 $M^{-1}cm^{-1}$; ESI-MS: calcd MW 4033.0 [M]: m/z found: 1344.9 [M+3H]$^{3+}$, 1008.9 [M+4H]$^{4+}$, 807.5 [M+5H]$^{5+}$, 673.0 [M+6H]$^{6+}$, 577.0 [M+7H]$^{7+}$. **PNA-6** (Ac-2-GGGCATGATCT-rrr-NH_2): 6.55%; Rt = 2.96 min; ε = 121800 $M^{-1}cm^{-1}$; ESI-MS: calcd MW 3889.2 [M]: m/z found: 1297.7[M+3H]$^{3+}$, 973.5[M+4H]$^{4+}$, 779.0 [M+5H]$^{5+}$, 649.3[M+6H]$^{6+}$, 556.7 [M+7H]$^{7+}$. **PNA-7** (5-O-GGGCATGATCT-rrr-NH_2): 11.21 %; Rt = 3.11, 3.25 min; ε = 113200 $M^{-1}cm^{-1}$; ESI-MS: calcd MW 3768.2 [M]: m/z found: 1257.4 [M+3H]$^{3+}$, 943.3 [M+4H]$^{4+}$, 755.0 [M+5H]$^{5+}$, 629.2 [M+6H]$^{6+}$, 539.5 [M+7H]$^{7+}$. **PNA-7T** (5-O-TGGGCATGATCT-rrr-NH_2): 9.82 %; Rt = 3.13; ε = 121800 $M^{-1}cm^{-1}$; ESI-MS: calcd MW 3890.9 [M]: m/z found: 1297.7 [M+3H]$^{3+}$, 973.5 [M+4H]$^{4+}$, 779.0[M+5H]$^{5+}$, 649.3[M+6H]$^{6+}$, 556.8[M+7H]$^{7+}$. **PNA-8F (**Ac-**1**-AACTTGGCT-rrk(Biot)-NH_2): 21.5 %; Rt = 3.11, 3.25 min; ε = 89800 $M^{-1}cm^{-1}$; ESI-MS: calcd MW 3768.2 [M]: m/z found: 1257.4 [M+3H]$^{3+}$, 943.3

$[M+4H]^{4+}$, 755.0 $[M+5H]^{5+}$, 629.2 $[M+6H]^{6+}$, 539.5 $[M+7H]^{7+}$. **PNA-8Ac (**Ac-AACTTGGCT-rrk(Biot)-NH_2): 23.0%; Rt = 3.11, 3.25 min; ε = 89800 $M^{-1}cm^{-1}$; ESI-MS: calcd MW 3768.2 [M]: m/z found: 1257.4 $[M+3H]^{3+}$, 943.3 $[M+4H]^{4+}$, 755.0 $[M+5H]^{5+}$, 629.2 $[M+6H]^{6+}$, 539.5 $[M+7H]^{7+}$. **PNA-9F (**Ac-**1**-AACTTGGCTAA-rrk(Biot)-NH_2): 20.8 %; Rt = 3.15 min; ε = 117200$M^{-1}cm^{-1}$; ESI-MS: calcd MW 3949.1 [M]: m/z found: 1257.4 $[M+3H]^{3+}$, 943.3 $[M+4H]^{4+}$, 755.0 $[M+5H]^{5+}$, 629.2 $[M+6H]^{6+}$, 539.5 $[M+7H]^{7+}$. **PNA-9Ac (**Ac-AACTTGGCTAA-rrk(Biot)-NH_2): 11.21 %; Rt = 3.14 min; ε = 117200$M^{-1}cm^{-1}$; ESI-MS: calcd MW 3710.8 [M]: m/z found: 1237.3 $[M+3H]^{3+}$, 928.2 $[M+4H]^{4+}$, 742.8 $[M+5H]^{5+}$, 619.2 $[M+6H]^{6+}$, 530.8 $[M+7H]^{7+}$. **PNA-10F (**Ac-**1**-AACTTGGCTAAAG-rrk(Biot)-NH_2): 15.7% %; Rt = 3.26, min; ε = 142600 $M^{-1}cm^{-1}$; ESI-MS: calcd MW 4499.6 [M]: m/z found: 1500.3$[M+3H]^{3+}$, 1125.4 $[M+4H]^{4+}$, 900.6 $[M+5H]^{5+}$, 750.6 $[M+6H]^{6+}$, 643.5 $[M+7H]^{7+}$. **PNA-10Ac (**Ac-AACTTGGCTAAAG-rrk(Biot)-NH_2): 18.3 %; Rt = 3.16 min; ε = 142600 $M^{-1}cm^{-1}$; ESI-MS: calcd MW 4277.4 [M]: m/z found: 1426.2 $[M+3H]^{3+}$, 1069.9 $[M+4H]^{4+}$, 856.2 $[M+5H]^{5+}$, 713.5 $[M+6H]^{6+}$, 611.7 $[M+7H]^{7+}$.

4. ICL experiments

Table S2. Sequences of PNA probes and DNA and RNA targets used in this study.

Probe	Sequence	Probe	Sequence
PNA-1A	Ac-GGGCA1GATC-rrr-NH_2	DNA-1-TA	5'-GCAGATCTAGCCCGGC-3'
PNA-1C	Ac-GGGCC1GATC-rrr-NH_2	DNA-1-TC	5'-GCAGATCTCGCCCGGC-3'
PNA-1G	Ac-GGGCG1GATC-rrr-NH_2	DNA-1-TG	5'-GCAGATCTGGCCCGGC-3'
PNA-1T	Ac-GGGCT1GATC-rrr-NH_2	DNA-1-TT	5'-GCAGATCTTGCCCGGC-3'
PNA-2A	Ac-GGGCA2GATC-rrr-NH_2	DNA-2-A	5'-TACGCATGCCC-A-3'
PNA-2C	Ac-GGGCC2GATC-rrr-NH_2	DNA-2-C	5'-TACGCATGCCC-C-3'
PNA-2G	Ac-GGGCG2GATC-rrr-NH_2	DNA-2-G	5'-TACGCATGCCC-G-3'
PNA-2T	Ac-GGGCT2GATC-rrr-NH_2	DNA-2-T	5'-TACGCATGCCC-T-3'
PNA-3A	Ac-GGGCA3GATC-rrr-NH_2	DNA-2-A_5	5'-TACGCATGCCC-AAAAA-3'
PNA-3C	Ac-GGGCC3GATC-rrr-NH_2	DNA-2-C_5	5'-TACGCATGCCC-CCCCC-3'
PNA-3G	Ac-GGGCG3GATC-rrr-NH_2	DNA-2-G_5	5'-TACGCATGCCC-GGGGG-3'
PNA-3T	Ac-GGGCT3GATC-rrr-NH_2	DNA-2-T_5	5'-TACGCATGCCC-TTTTT-3'
PNA-4A	Ac-GGGCA4GATC-rrr-NH_2	DNA-2-C1T_4	5'-TACGCATGCCC-CTTTT-3'
PNA-4C	Ac-GGGCC4GATC-rrr-NH_2	DNA-2-C2T_4	5'-TACGCATGCCC-TCTTT-3'
PNA-4G	Ac-GGGCG4GATC-rrr-NH_2	DNA-2-C3T_4	5'-TACGCATGCCC-TTCTT-3'
PNA-4T	Ac-GGGCT4GATC-rrr-NH_2	DNA-2-C4T_4	5'-TACGCATGCCC-TTTCT-3'
PNA-5	Ac-1-GGGCATGATCT-rrr-NH_2	DNA-2-C5T_4	5'-TACGCATGCCC-TTTTC-3'
PNA-5T	Ac-1-TGGGCATGATCT-rrr-NH_2	DNA-3-A	5'-TACGCATGCCCA-A-3'
PNA-6	Ac-2-GGGCATGATCT-rrr-NH_2	DNA-3-C	5'-TACGCATGCCCA-C-3'
PNA-7	5-O-GGGCATGATCT-rrr-NH_2	DNA-3-G	5'-TACGCATGCCCA-G-3'
PNA-7T	5-O-TGGGCATGATCT-rrr-NH_2	DNA-3-T	5'-TACGCATGCCCA-T-3'
PNA-8Ac	Ac-AACTTGGCTAAAG-rrk(Biot)-NH_2	DNA-3-A_5	5'-TACGCATGCCCA-AAAAA-3'
PNA-8F	Ac-**1**-AACTTGGCTAAAG-rrk(Biot)-NH_2	DNA-3-C_5	5'-TACGCATGCCCA-CCCCC-3'
PNA-9Ac	Ac-AACTTGGCTAA- rrk(Biot)-NH_2	DNA-3-G_5	5'-TACGCATGCCCA-GGGGG-3'
PNA-9F	Ac-**1**-AACTTGGCTAA- rrk(Biot)-NH_2	DNA-3-T_5	5'-TACGCATGCCCA-TTTTT-3'
PNA-10Ac	Ac-AACTTGGCT- rrk(Biot)-NH_2	DNA-3-C1T_4	5'-TACGCATGCCCA-CTTTT-3'
PNA-10F	Ac-**1**-AACTTGGCT- rrk(Biot)-NH_2	DNA-3-C2T_4	5'-TACGCATGCCCA-TCTTT-3'
DNA-1-AA	5'-GCAGATCAAGCCCGGC-3'	DNA-3-C3T_4	5'-TACGCATGCCCA-TTCTT-3'
DNA-1-AC	5'-GCAGATCACGCCCGGC-3'	DNA-3-C4T_4	5'-TACGCATGCCCA-TTTCT-3'
DNA-1-AG	5'-GCAGATCAGGCCCGGC-3'	DNA-3-C5T_4	5'-TACGCATGCCCA-TTTTC-3'
DNA-1-AT	5'-GCAGATCATGCCCGGC-3'	RNA-A	5'-UACGCAUGCCC-A-3'
DNA-1-CA	5'-GCAGATCCAGCCCGGC-3'	RNA-C	5'-UACGCAUGCCC-C-3'
DNA-1-CC	5'-GCAGATCCCGCCCGGC-3'	RNA-G	5'-UACGCAUGCCC-G-3'
DNA-1-CG	5'-GCAGATCCGGCCCGGC-3'	RNA-U	5'-UACGCAUGCCC-U-3'
DNA-1-CT	5'-GCAGATCCTGCCCGGC-3'	RNA-C1U_4	5'-UACGCAUGCCC-CUUUU-3'
DNA-1-GA	5'-GCAGATCGAGCCCGGC-3'	RNA-C2U_4	5'-UACGCAUGCCC-UCUUU-3'
DNA-1-GC	5'-GCAGATCGCGCCCGGC-3'	RNA-C3U_4	5'-UACGCAUGCCC-UUCUU-3'
DNA-1-GG	5'-GCAGATCGGGCCCGGC-3'	RNA-C4U_4	5'-UACGCAUGCCC-UUUCU-3'
DNA-1-GT	5'-GCAGATCGTGCCCGGC-3'	RNA-C5U_4	5'-UACGCAUGCCC-UUUUC-3'
RNA-SAMMSON	5'-ACCUUUAGCCAAGUUCACAC-3'		

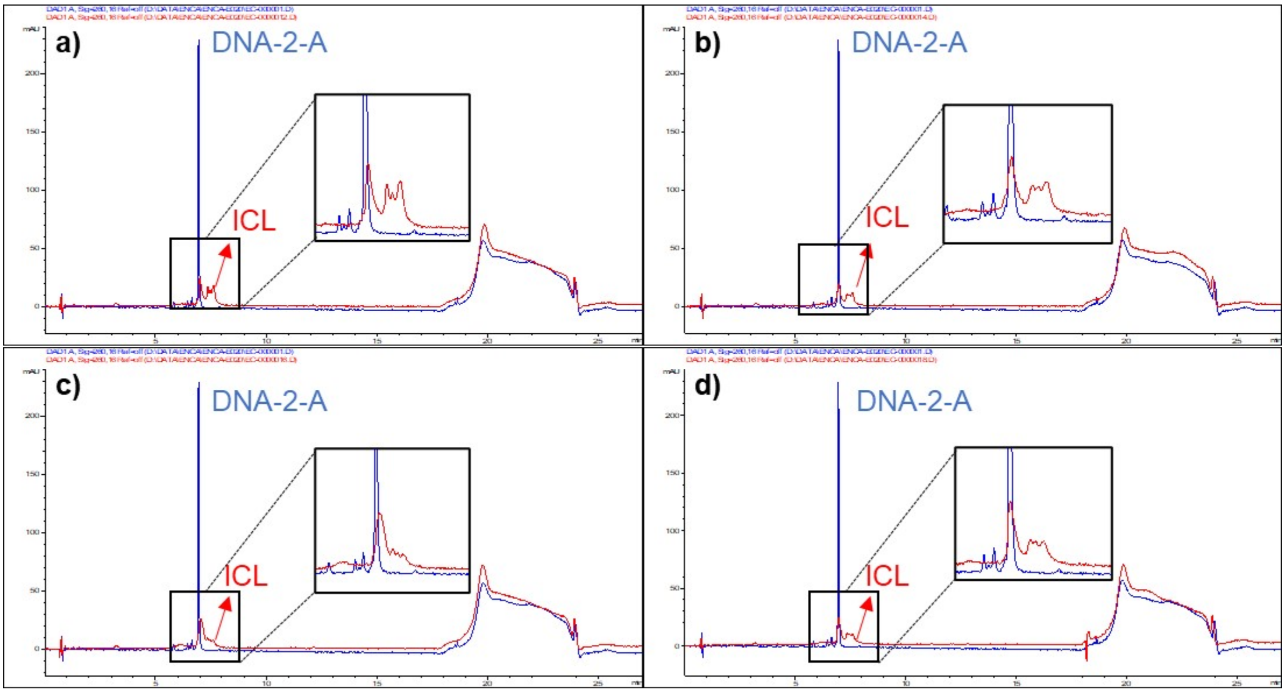

Figure S2. ICL reaction performed in presence of **DNA-2A** and **PNA-5**, using MB as photosensitizer at a final concentration of 2 µM, varying solution composition. Experiments performed at 5 µM final strand concentration, in PBS buffer pH 7.4 supplemented with 200 mM Ca^{2+} (a), 200 mM Mg^{2+} (b), 200 mM K^{+} (c) or in a 30% PEG6000 solution supplemented with 150 mM K^{+}, 10 mM Na^{+} and 0.5 mM Mg^{2+}(d).

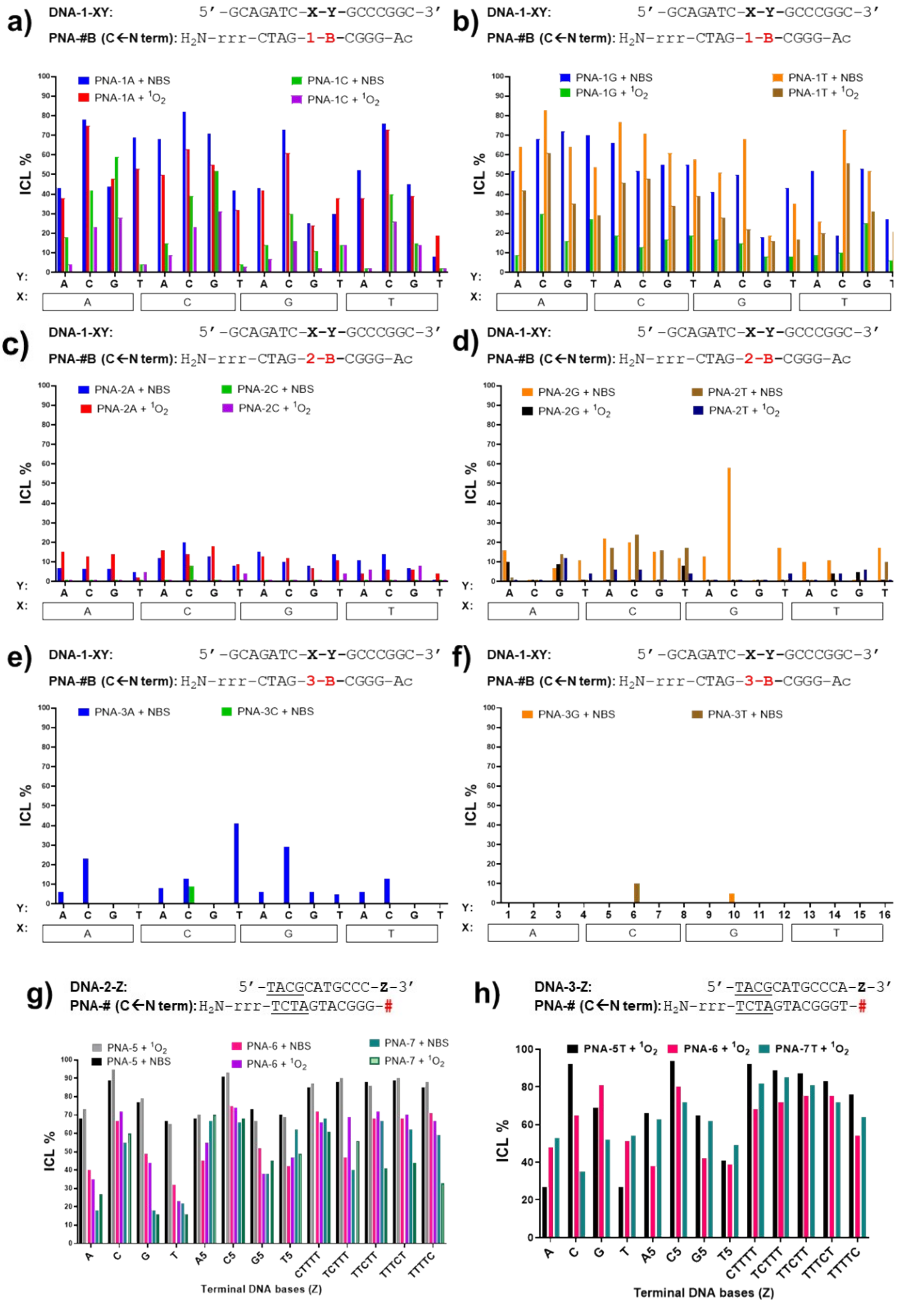

Figure S3. ICL% for each PNA used in this study, towards their target DNAs, in presence of NBS (4 eq) or upon 20’ irradiation in presence of MB (2 µM). a-f) **PNA-1B** to **PNA-3B** in presence of target **DNA-1-XY**; g-h) **PNA-5** to **PNA-7T**, in presence of **DNA-2-Z** and **DNA-3-Z**. Experiments performed at 5 µM probe concentration in PBS.

PAGE experiments

Crosslink samples were analysed on a 20% polyacrylamide gel (acrylamide:bisacrylamide 19:1) prepared in 1× TBE buffer containing 7 M urea. 10 µL of a 1:4 sample/formamide solution are loaded in each pit. The power supply used for gel electrophoresis was a consort EV202 and a constant voltage of 230 V was used to run the gels. Gels were stained with SYBR gold (Thermo Fisher Scientific, Life Technologies, Merelbeke, Belgium) and pictures were taken with an Autochemi imaging system (UVP).

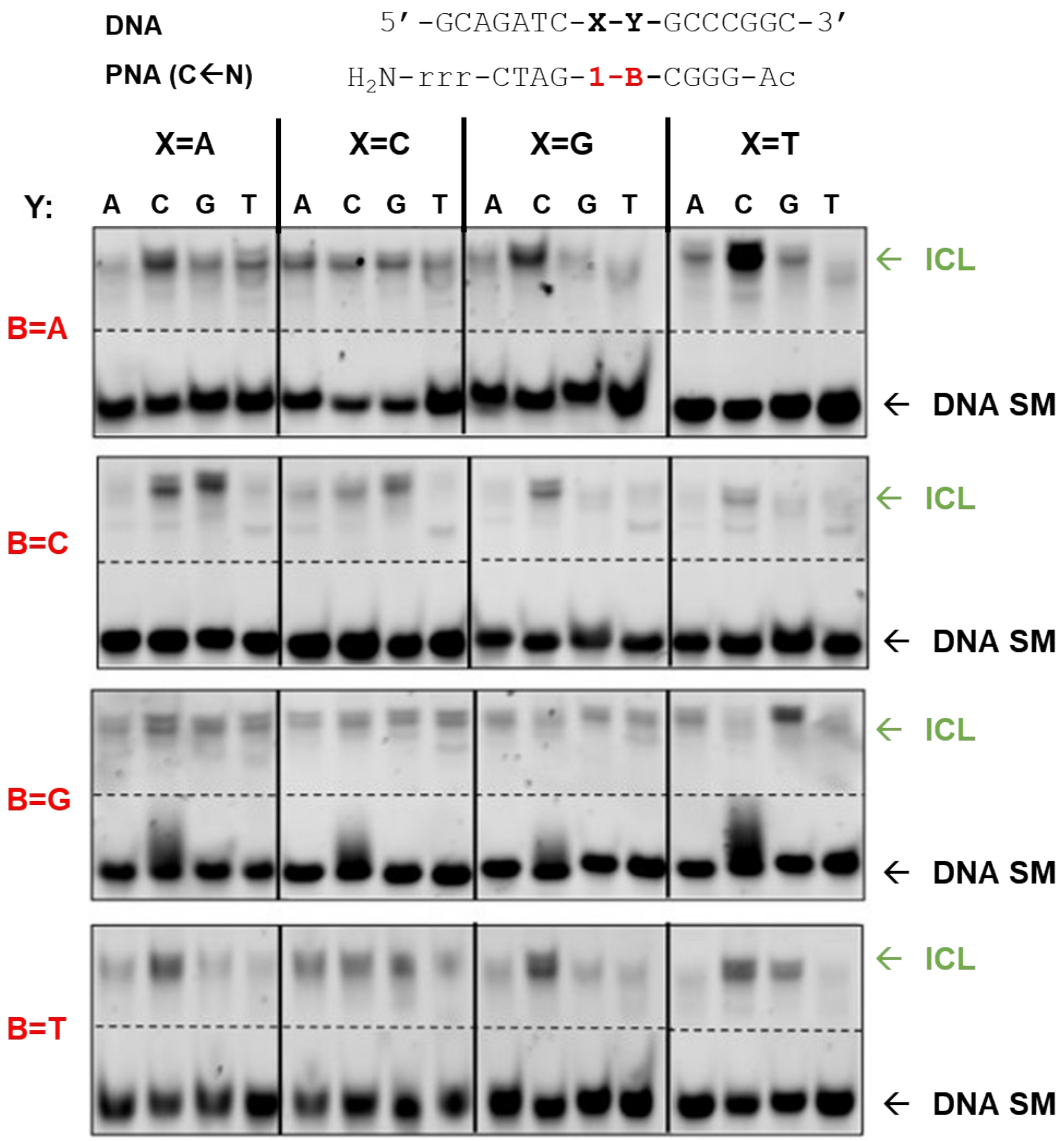

Figure S4. PAGE analysis of the ICL reactions with **PNA-1B**, in presence of DNA-1-XY. In each line, indicating a different B permutation, DNA starting material (SM) and ICL product, are indicated with a black and green arrow, respectively.

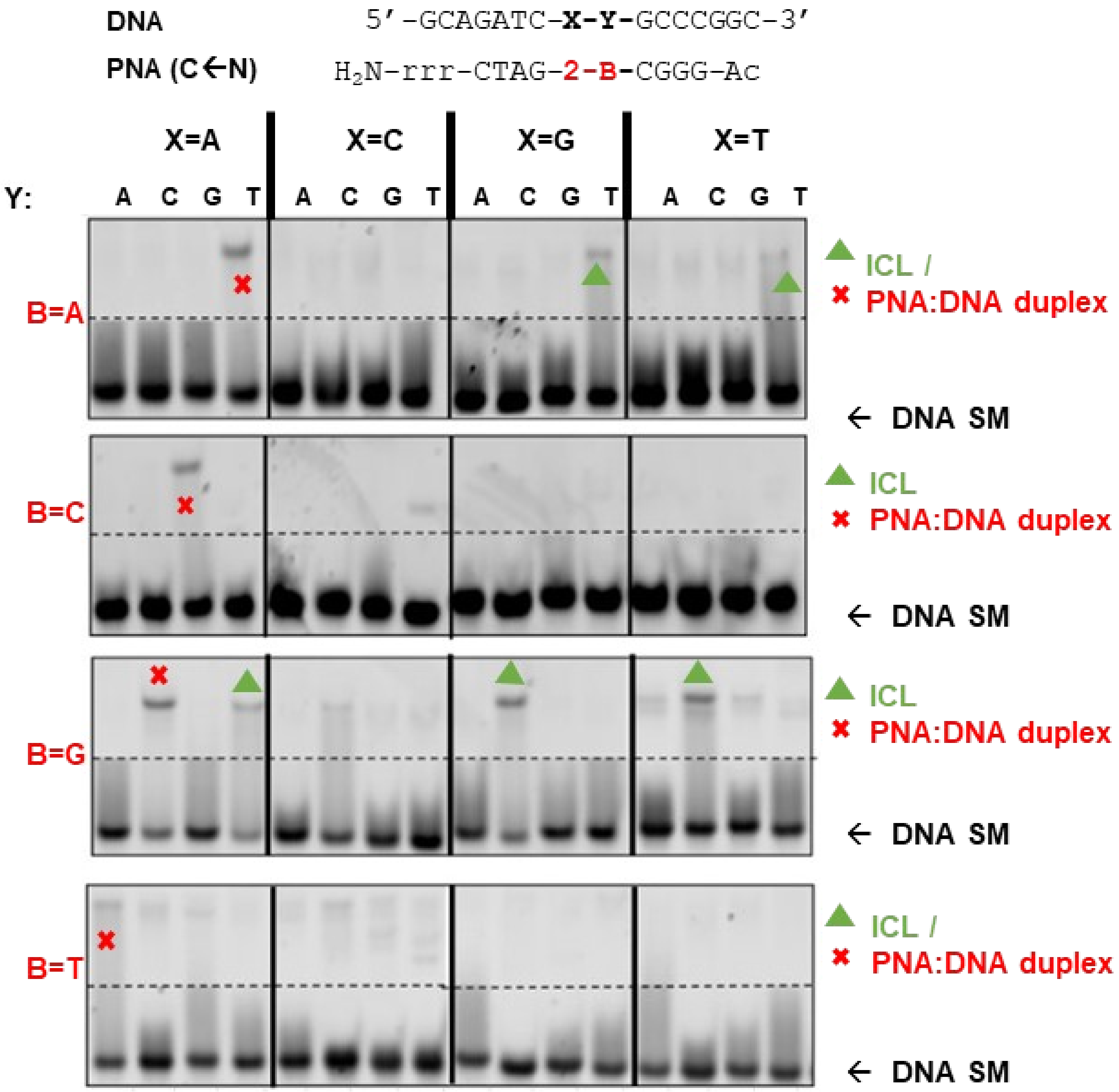

Figure S5. PAGE analysis of the ICL reactions with **PNA-2B**, in presence of DNA-1-XY. In each line, indicating a different B permutation, DNA starting material (SM) and ICL product, are indicated with a black arrow and green symbol, respectively. In some cases, the upper band indicates the formation of a stable PNA:DNA duplex (red **x**).

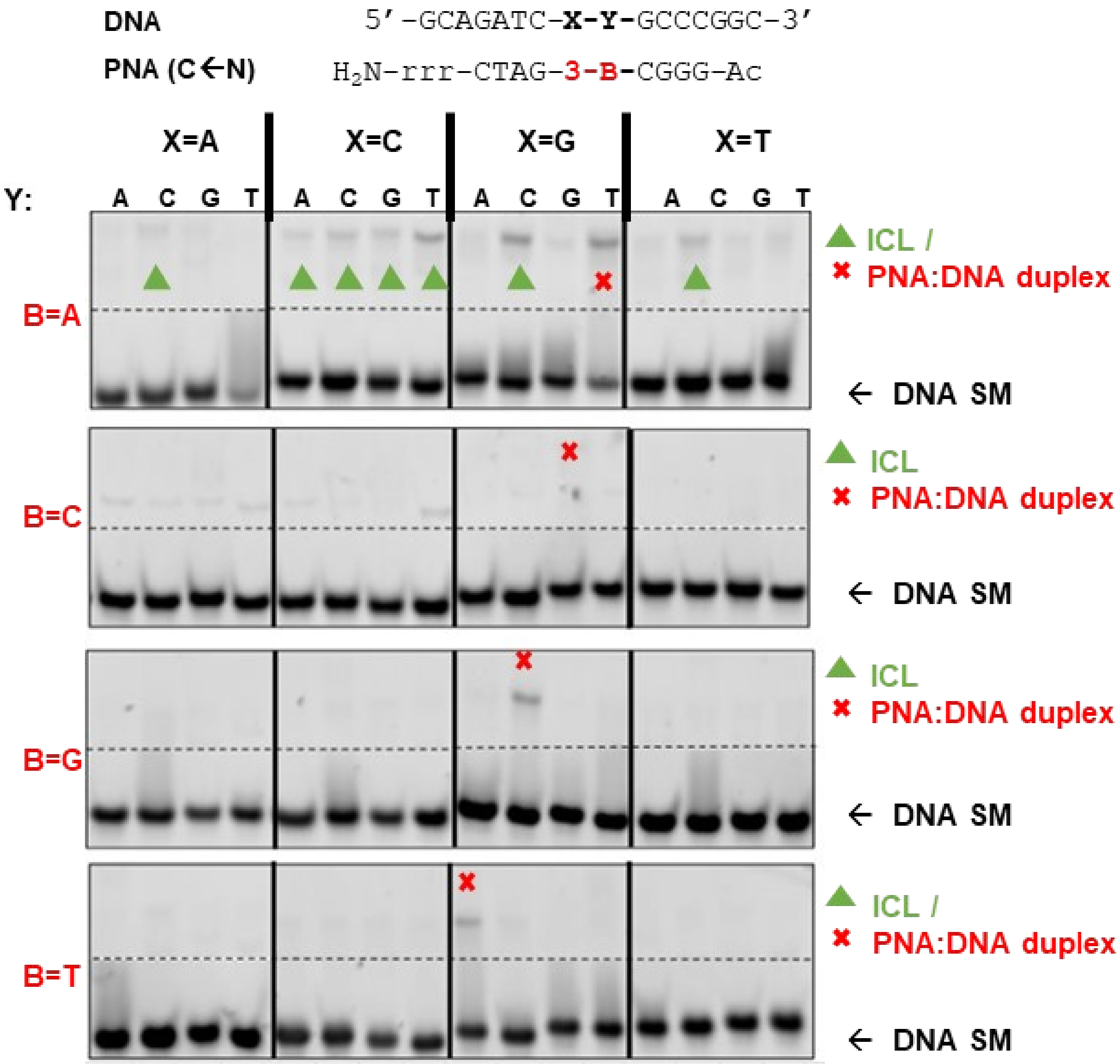

Figure S6. PAGE analysis of the ICL reactions with **PNA-3B**, in presence of DNA-1-XY. In each line, indicating a different B permutation, DNA starting material (SM) and ICL product, are indicated with a black arrow and green symbol, respectively. In some cases, the upper band indicates the formation of a stable PNA:DNA duplex (red **x**).

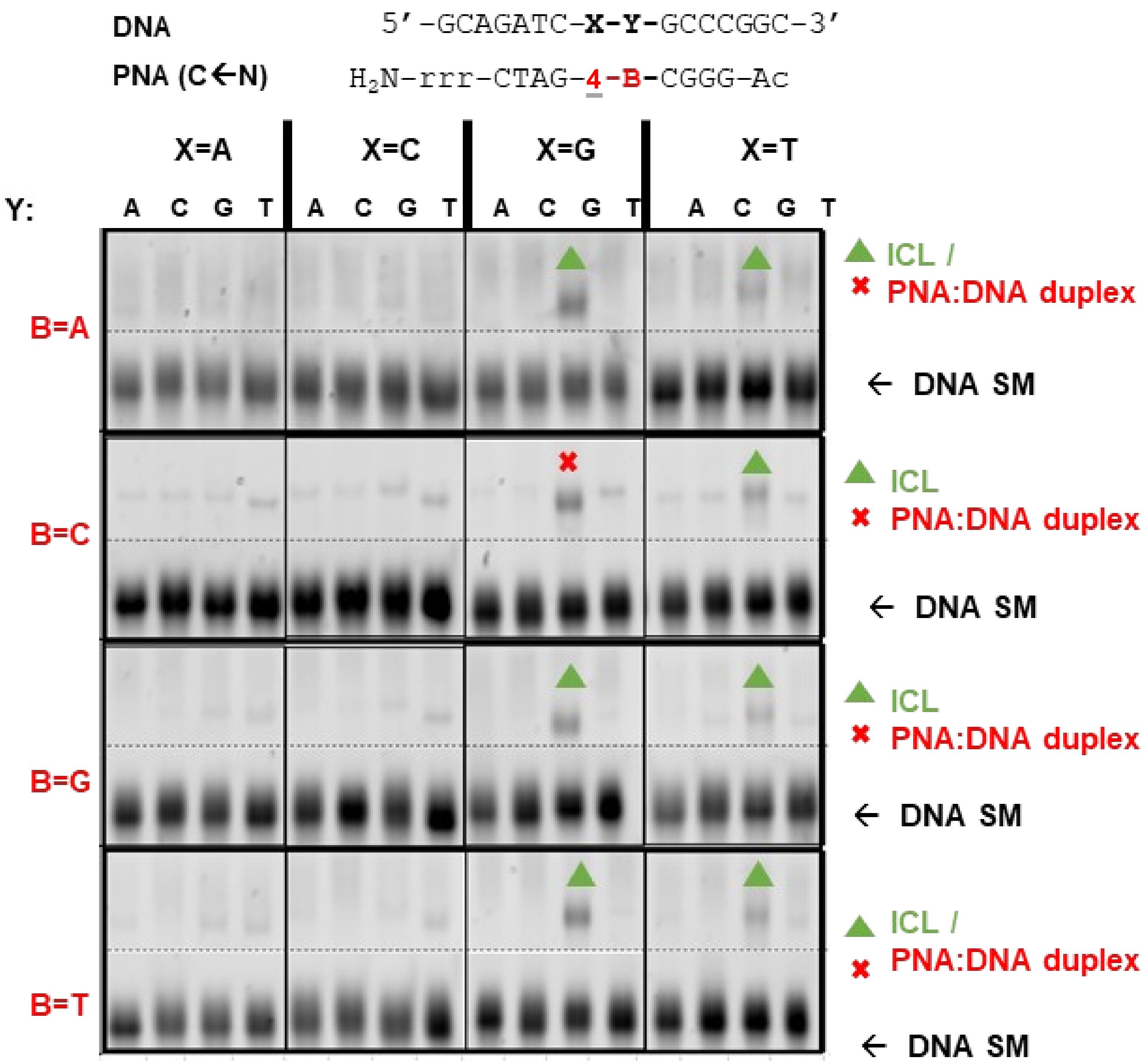

Figure S7. PAGE analysis of the ICL reactions with **PNA-4B**, in presence of DNA-1-XY. In each line, indicating a different B permutation, DNA starting material (SM) and ICL product, are indicated with a black arrow and green symbol, respectively. In some cases, the upper band indicates the formation of a stable PNA:DNA duplex (red **x**).

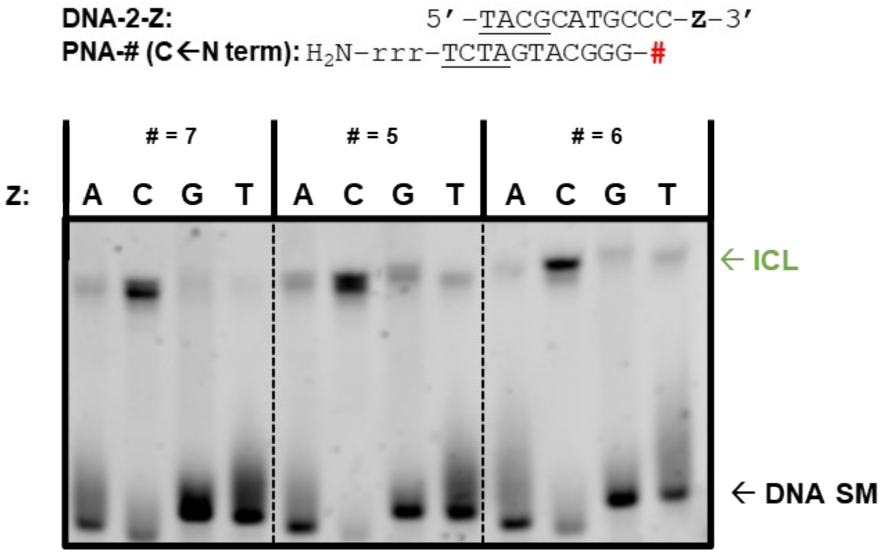

Figure S8. PAGE analysis of the ICL reactions of **PNA-5**, **PNA-6**, and **PNA-7** in presence of **DNA-2-Z** probes. The specific nucleobase placed at the 3'-end is indicated as lane header. DNA starting material (SM) and ICL product, are indicated with black and green arrows, respectively.

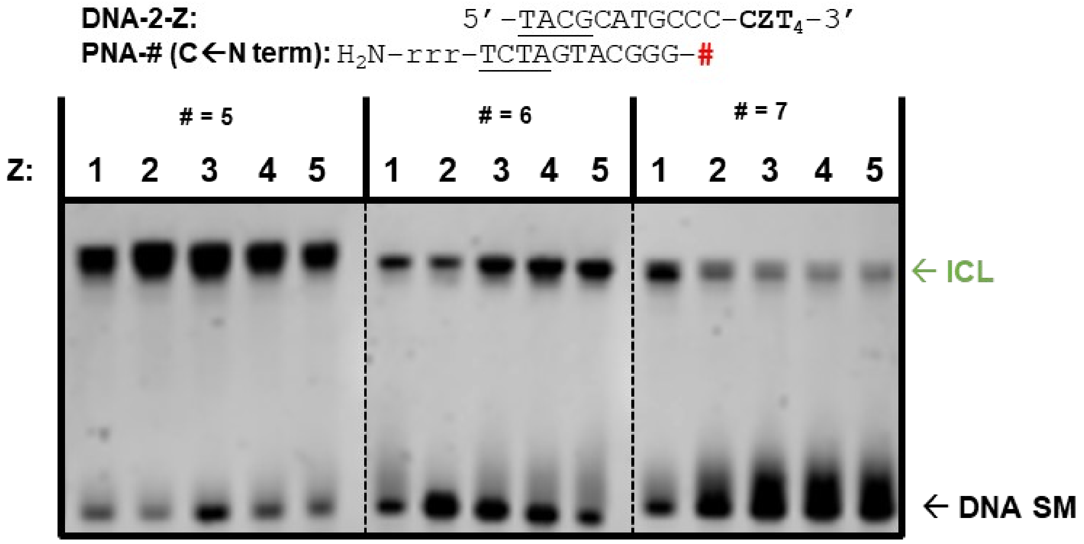

Figure S9. PAGE analysis of the ICL reactions with **PNA-5**, **PNA-6**, and **PNA-7** in presence of **DNA-2-CZT$_4$** (C-scan experiment) probes. Cytosine position in the T-tail is indicated as lane header. DNA starting material (SM) and ICL product, are indicated with black and green arrows, respectively.

5. Representative MALDI spectra

For each ICL experiment, 30 µL of crosslinked sample were purified by HPLC-UV (HPLC-3), freeze-dried and resuspended in 10 µL of mQ water. 0.3 µL of sample were spotted on the plate and after evaporation of the solvent, 0.3 µL of matrix (2,5-DHB, 100 mg/mL in H_2O: MeCN + 0.1% TFA) were subsequently added.

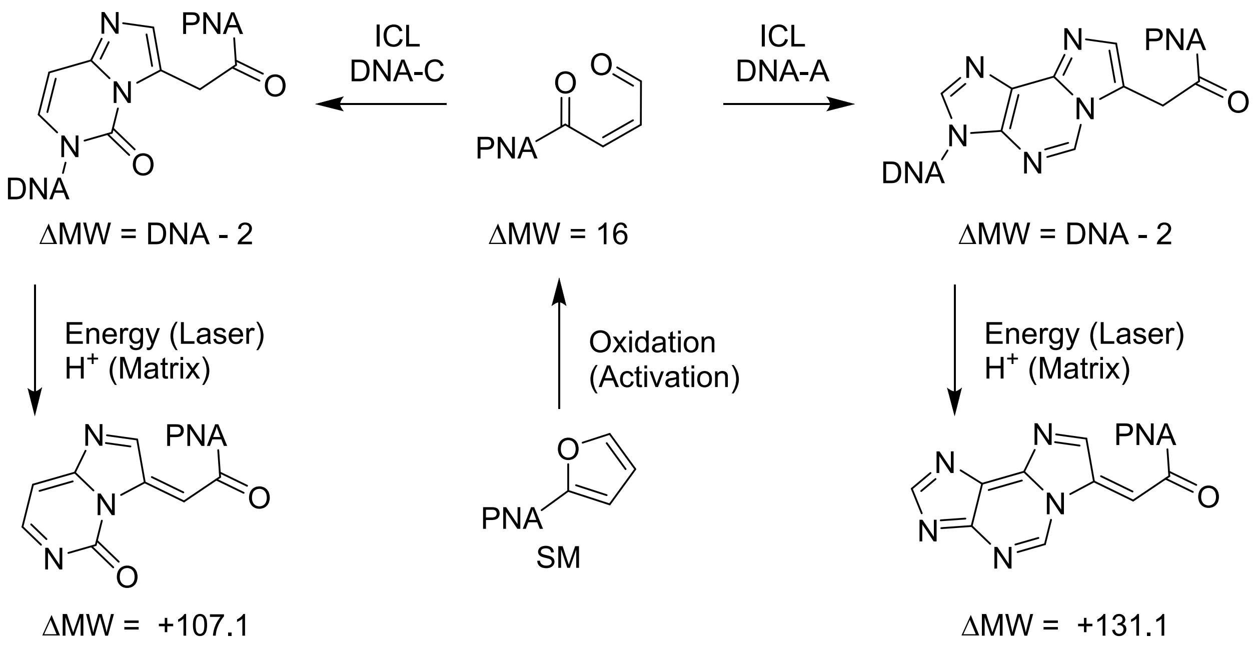

Figure S10. Proposed MALDI in-source fragmentation as previously reported[4]

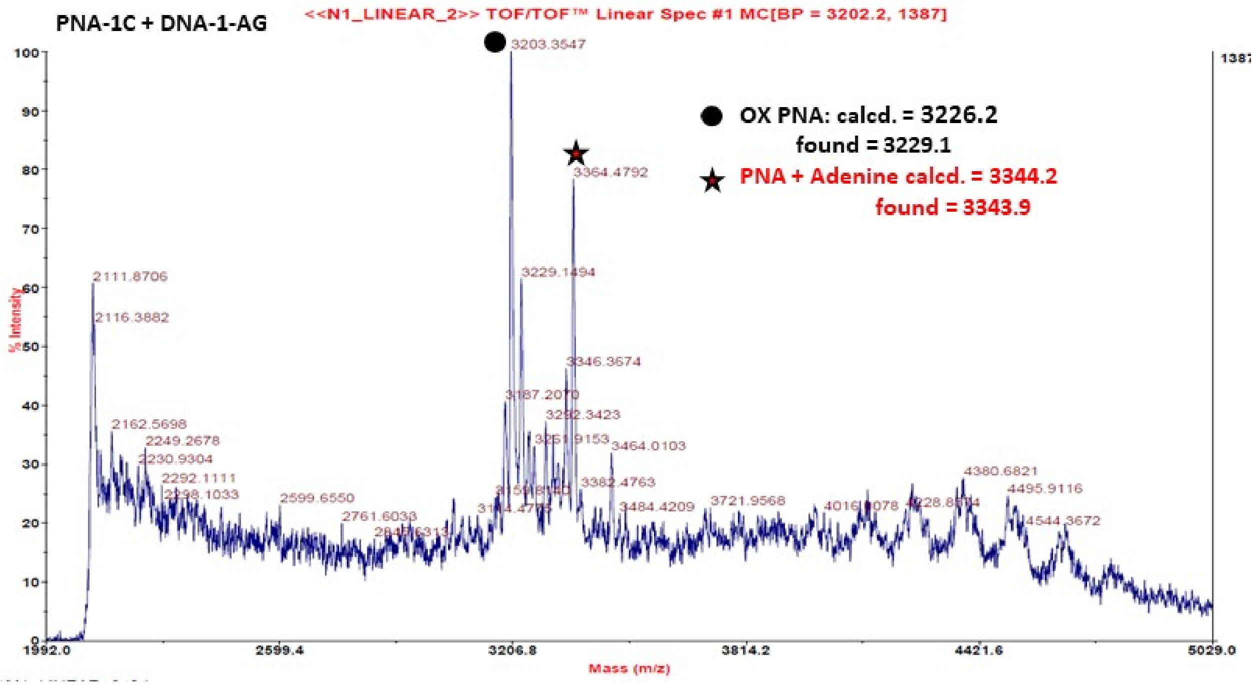

Figure S11. MALDI characterization of the reaction between **PNA-1C** and DNA-1-AG.

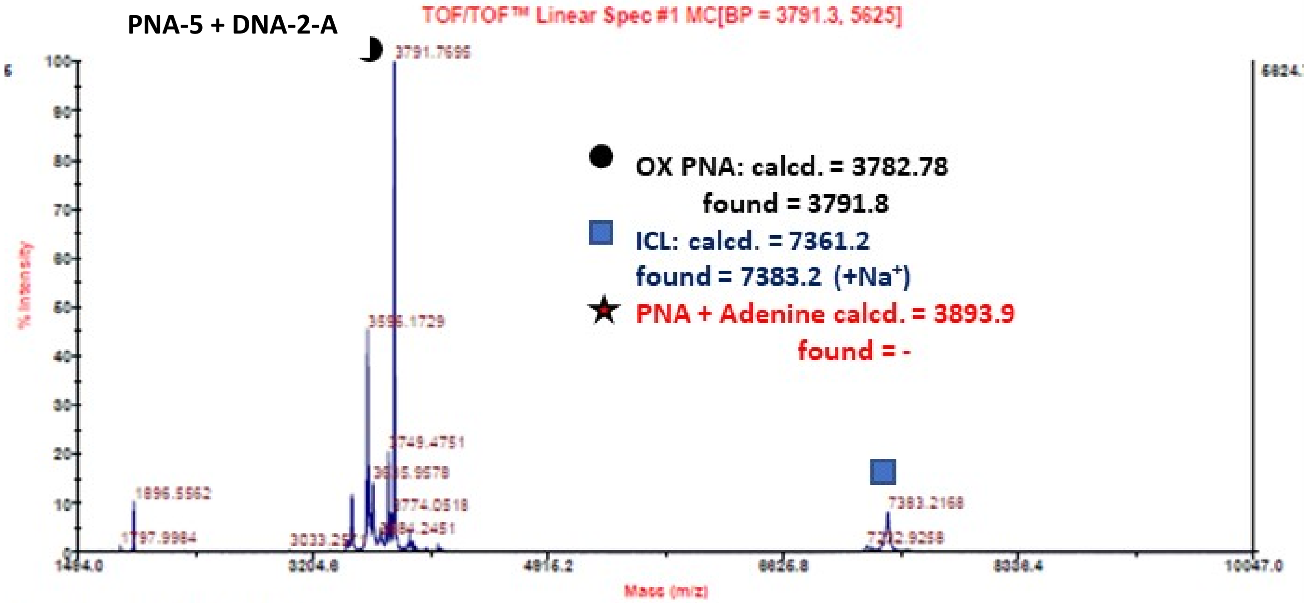

Figure S12. MALDI characterization of the reaction between **PNA-5** and DNA-2-A.

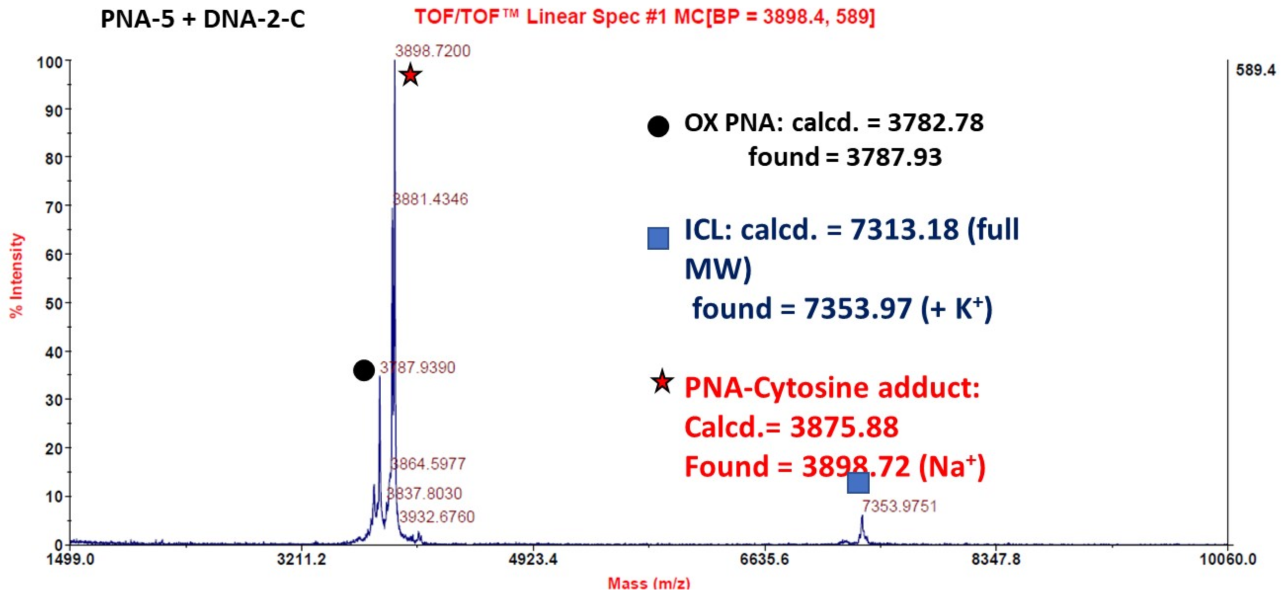

Figure S13. Maldi characterization of the reaction between **PNA-5** and DNA-2-C.

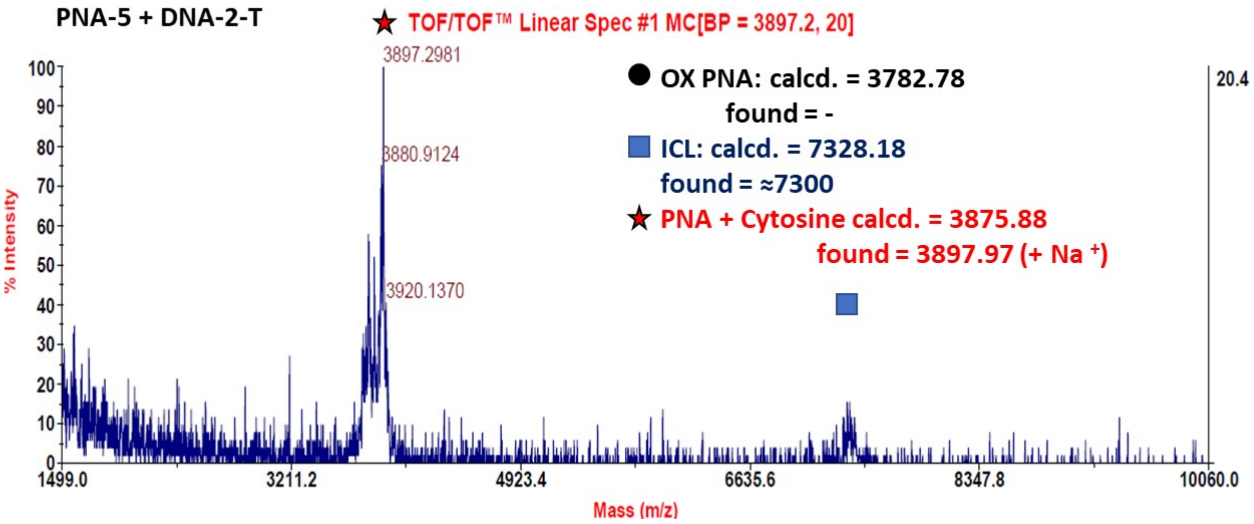

Figure S14 MALDI characterization of the reaction between **PNA-5** and DNA-2-T

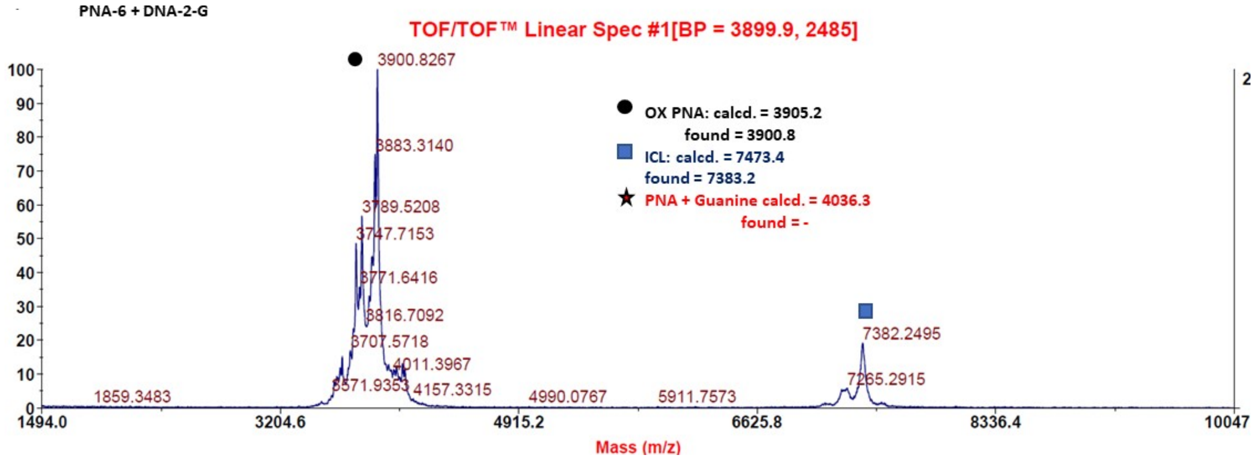

Figure S15 MALDI characterization of the reaction between **PNA-6** and DNA-2-G.

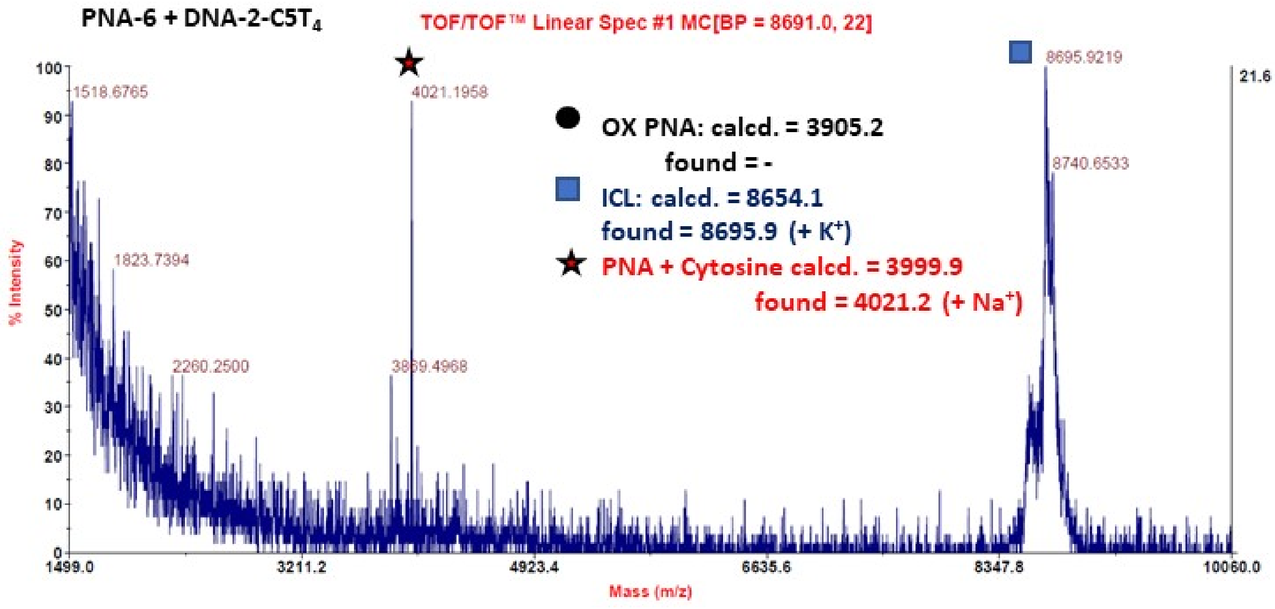

Figure S16 MALDI characterization of the reaction between **PNA-6** and DNA-2-C5T$_4$.

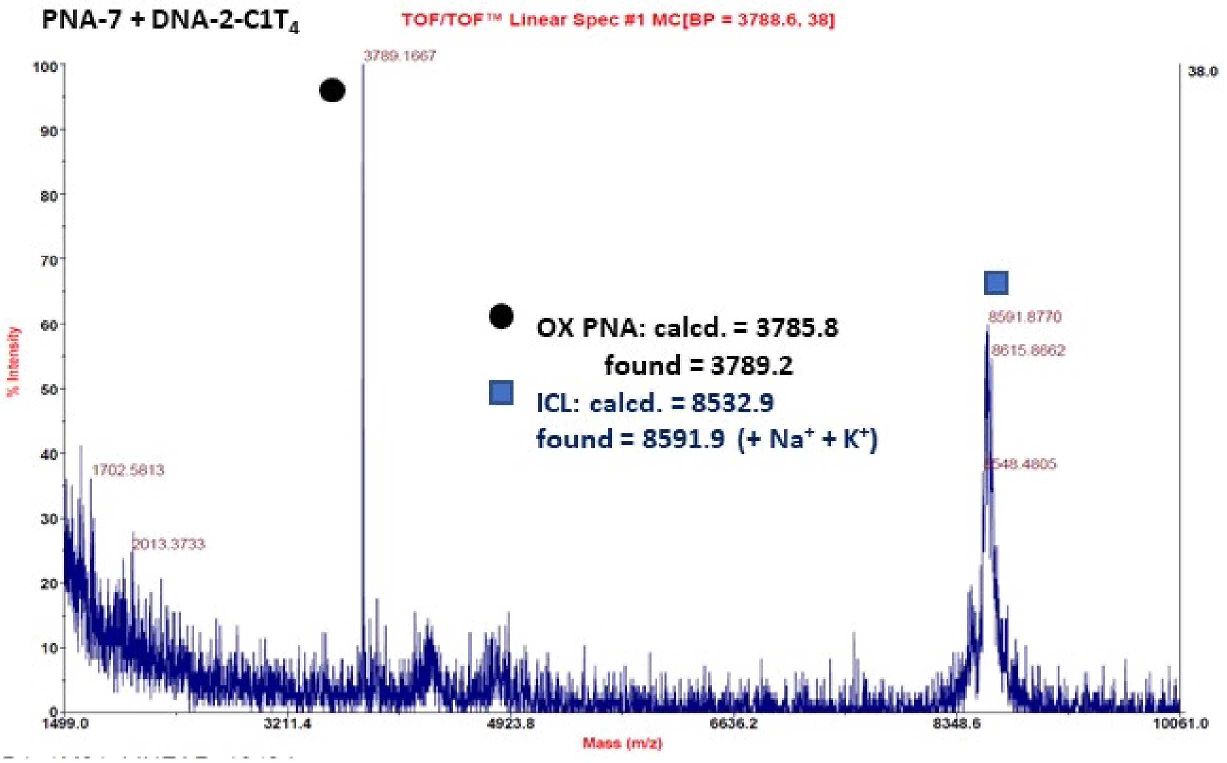

Figure S17 MALDI characterization of the reaction between **PNA-7** and DNA-2-C1T$_4$.

6. ICL reversibility

In a typical experiment, 100 µM working solution of PS was freshly prepared from a 1 mM stock solution. In a 1.5 mL Eppendorf tube, a 100 µL solution containing both PNA and DNA probes at 5 µM in PBS buffer (100 mM NaCl, 10 mM phosphates) pH 7.4 was prepared. This solution was allowed to equilibrate for 15 minutes at 25 °C before the addition of Methylene Blue at 5 µM concentration. The lamp (100 W halogen lamp LE.5210 Euromex EK-1 illuminator, equipped with Euromex LE.5214 dual arm light conductor) was placed on top of the Eppendorf tube for the entire duration of the experiment (20'). The intensity of the lamps was set before starting the irradiation, using a TES 1335 luxmeter equipped with a custom fitting for the lamp bulbs to maintain the light intensity constant and similar for each irradiated sample. After the irradiation, the sample was heated to 95°C for 1 hour, and intermediate aliquots were taken.

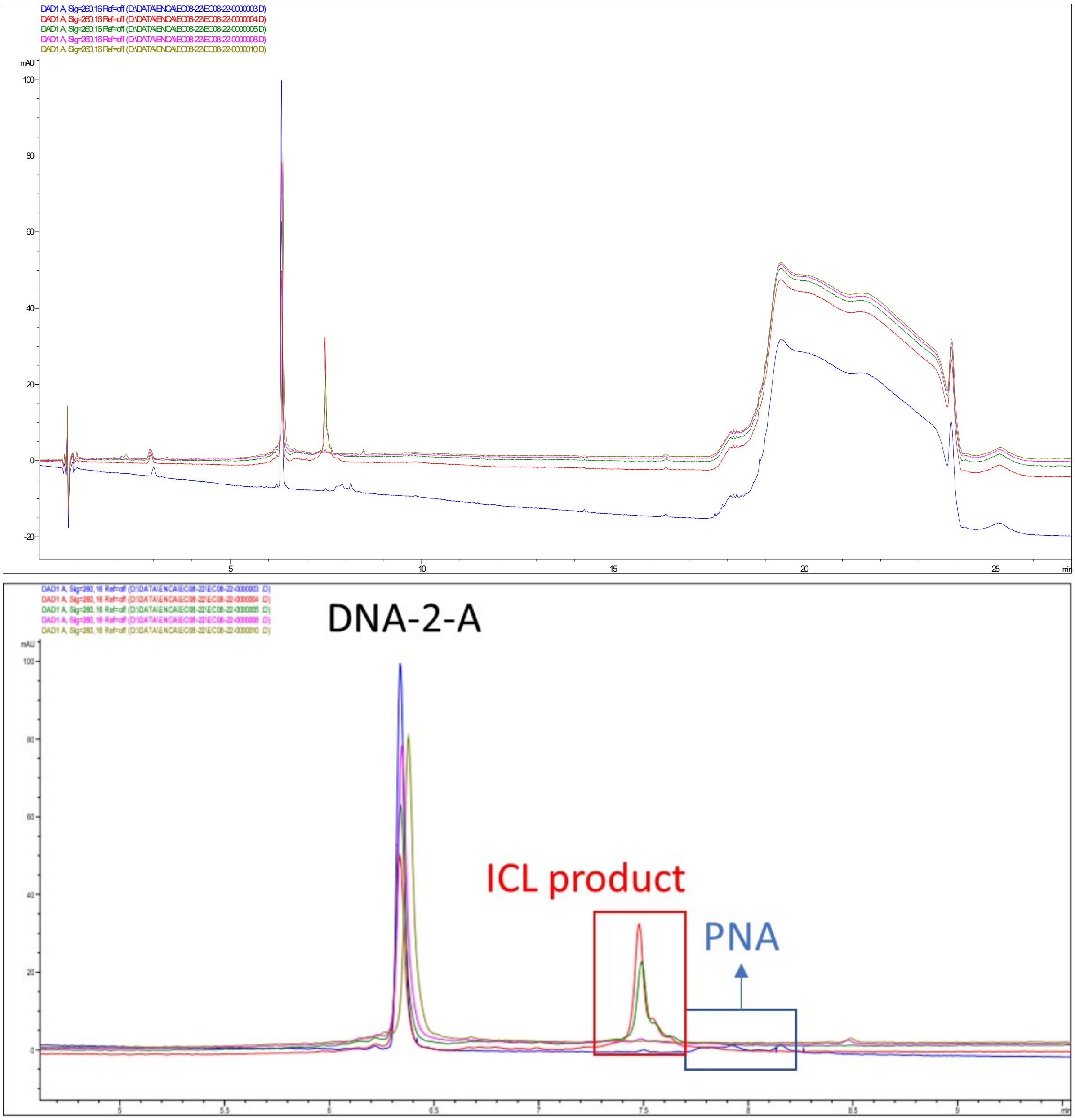

Figure S18. ICL reversibility between **PNA-5** and **DNA-2-A**. Full chromatogram (top) and zoom of the DNA peaks region (bottom).

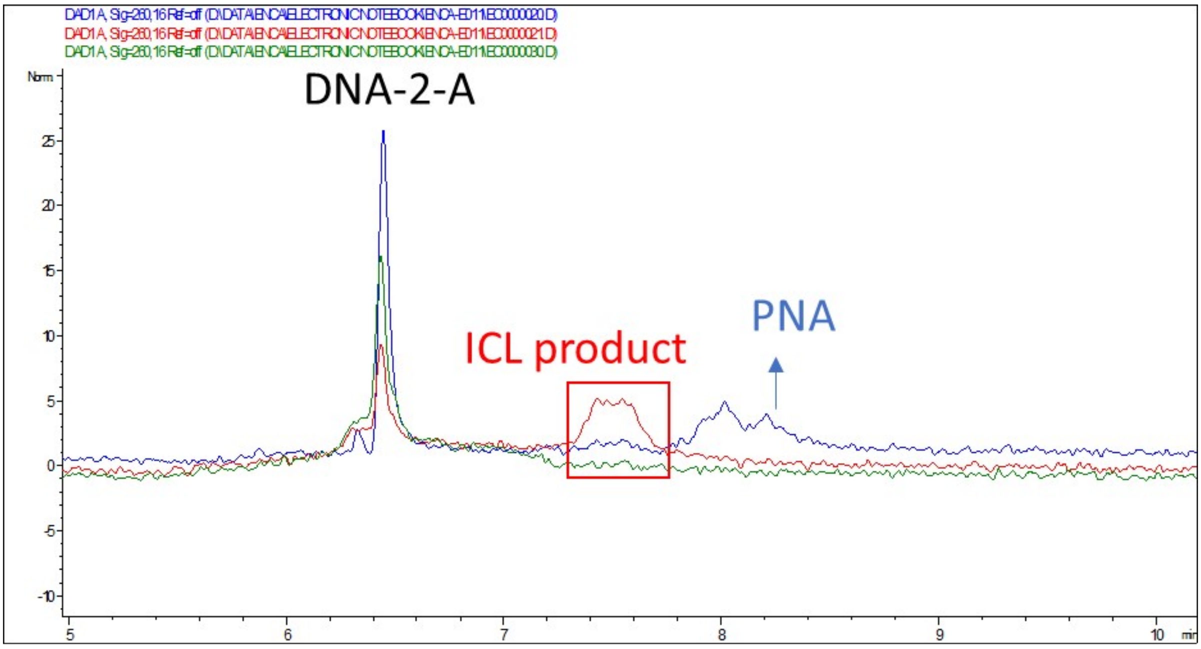

Figure S19. ICL reversibility between **PNA-5** and **RNA-A**. The blue trace indicates T=0, the red trace indicates the sample after ICL reaction. The green trace indicates the sample after treatment of 1h at 95°C.

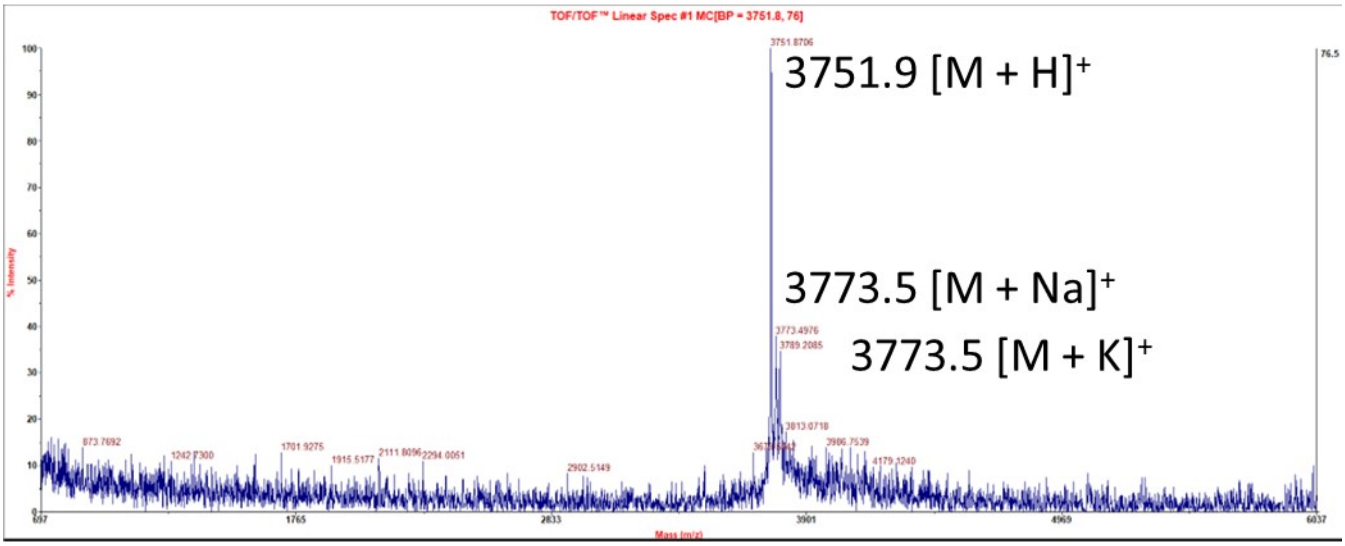

Figure S20. MALDI-TOF analysis of the purified RNA peak after ICL reversion upon heating the sample at 95°C for 1 hour.

7. Pull-down experiments

Prior to each experiment, Invitrogen ™ Dynabeads™ MyOne™ C1 magnetic beads (10 mg/mL) were vortexed for 30 seconds and transferred to a 1.5 mL Eppendorf tube. Following the manufacturer's protocol, beads were washed 3 times with 1x Binding and Washing buffer (Tris-HCl 5 mM, pH 7.5, 1 M NaCl, 0.5 mM EDTA) and resuspended in 30 µL of 2X Binding and Washing Buffer. 30 µL of crosslinked sample obtained using either methylene blue at 2 µM, or rhodamine B at 5 µM concentration (or hybridized PNA:RNA sample in absence of photosensitizer), at 5 µM strand concentration in cell lysate (SK-MEL-28, 55 million cells/mL, or MDA-MB-231, 4 million cells/mL), was mixed with an equal amount of resuspended beads (5 mg/mL). The beads were equilibrated with the sample for 30', after which they were washed 5 times with 1 mL of Binding and Washing buffer and resuspended in 30 µL of milli-Q water. The release of the RNA from the probes was achieved by incubating the beads at 95°C for 1 hour in an Eppendorf Thermomixer. The obtained solution was analysed by HPLC-UV.

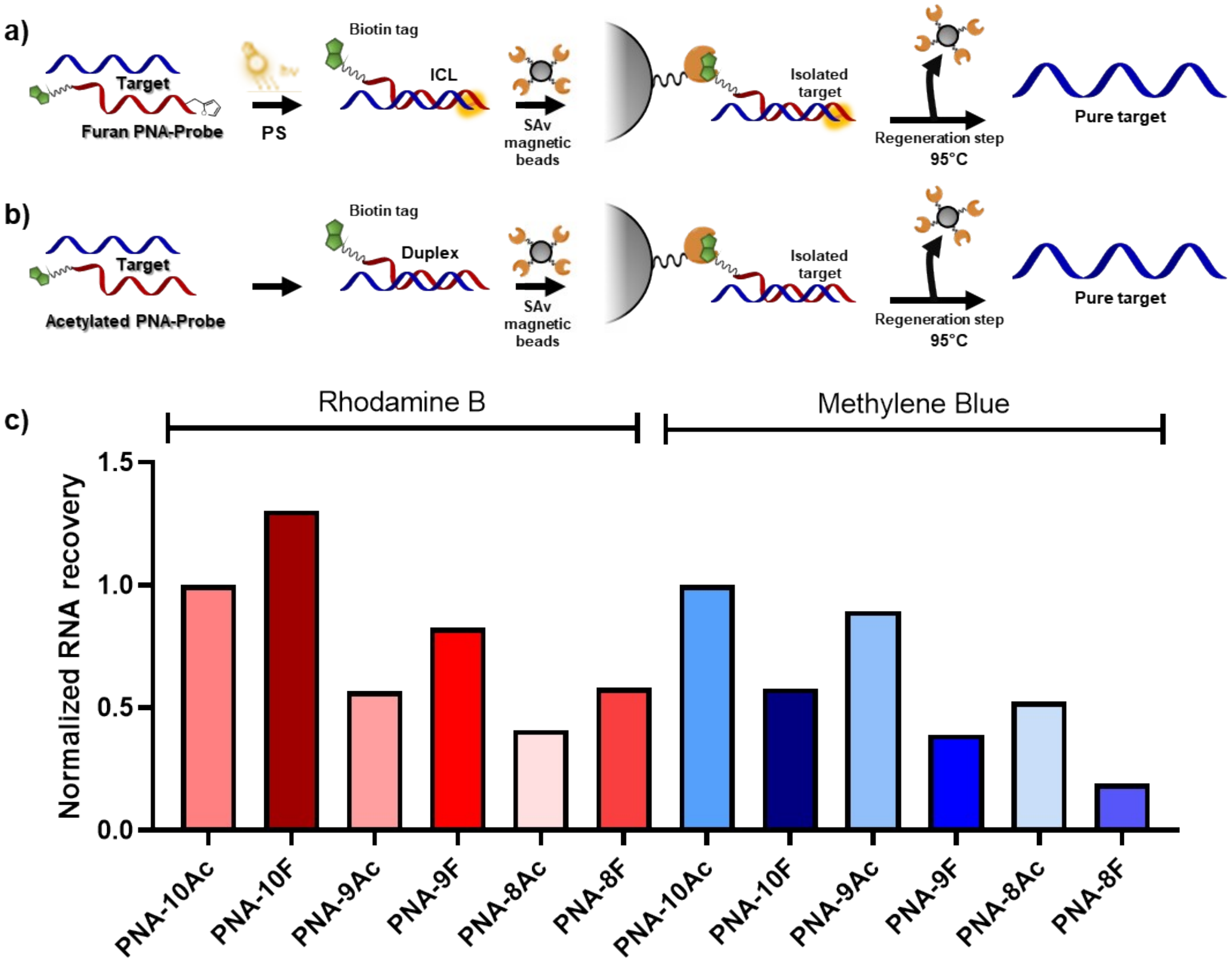

Figure S21. Pull-down experiment performed in this study. Comparison between the Pull-down with Furan-containing PNA probes **PNA-8F**, **PNA-9F**, **PNA-10F** (A) and with the acetylated probes **PNA-8Ac**, **PNA-9Ac**, **PNA-10Ac** (B). C) Comparison between the recovery of **RNA SAMMSON** performed after crosslinking with rhodamine B, 5 µM (red bars) and methylene blue 2 µM (blue bars). Experiment performed at 5 µM strand concentration, in PBS pH 7.4 supplemented with cell lysate (SK-MEL-28, 55 million cells/mL) Each dataset is normalized with respect to the **PNA-10Ac** experiment performed.

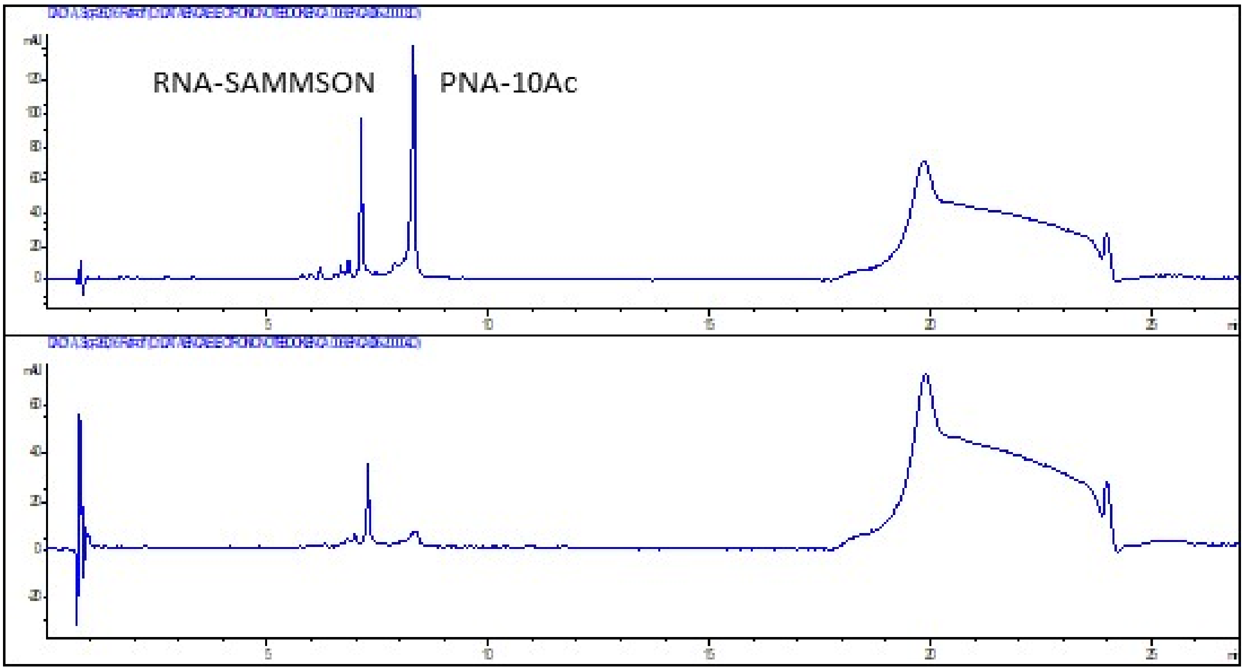

Figure S22. Pull down experiment of **RNA-SAMMSON** spiked in cell-lysate (SK-MEL-28, 55 million cells/mL), performed with **PNA-10Ac**. Top: starting solution; bottom: solution after pull down (incubation, washing and release by heating for 1h at 95°C).

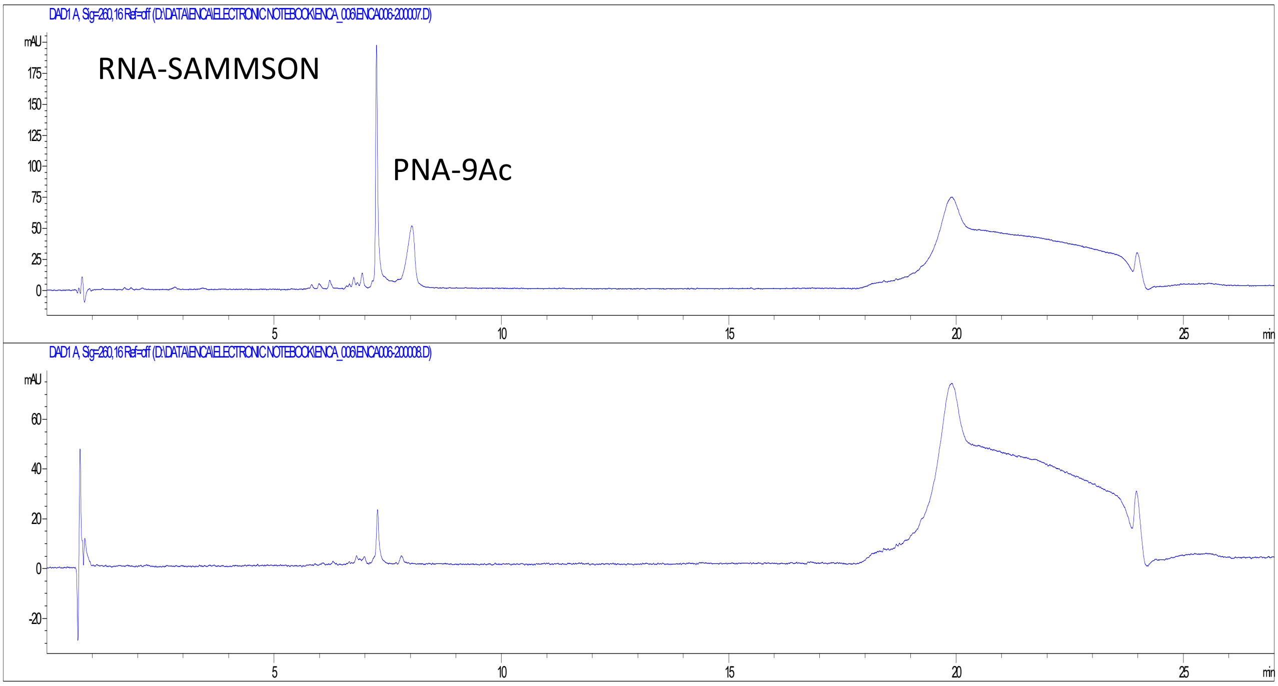

Figure S23. Pull down experiment of **RNA-SAMMSON** spiked in cell-lysate (SK-MEL-28, 55 million cells/mL), performed with **PNA-9Ac**. Top: starting solution; bottom: solution after pull down (incubation, washing and release by heating for 1h at 95°C).

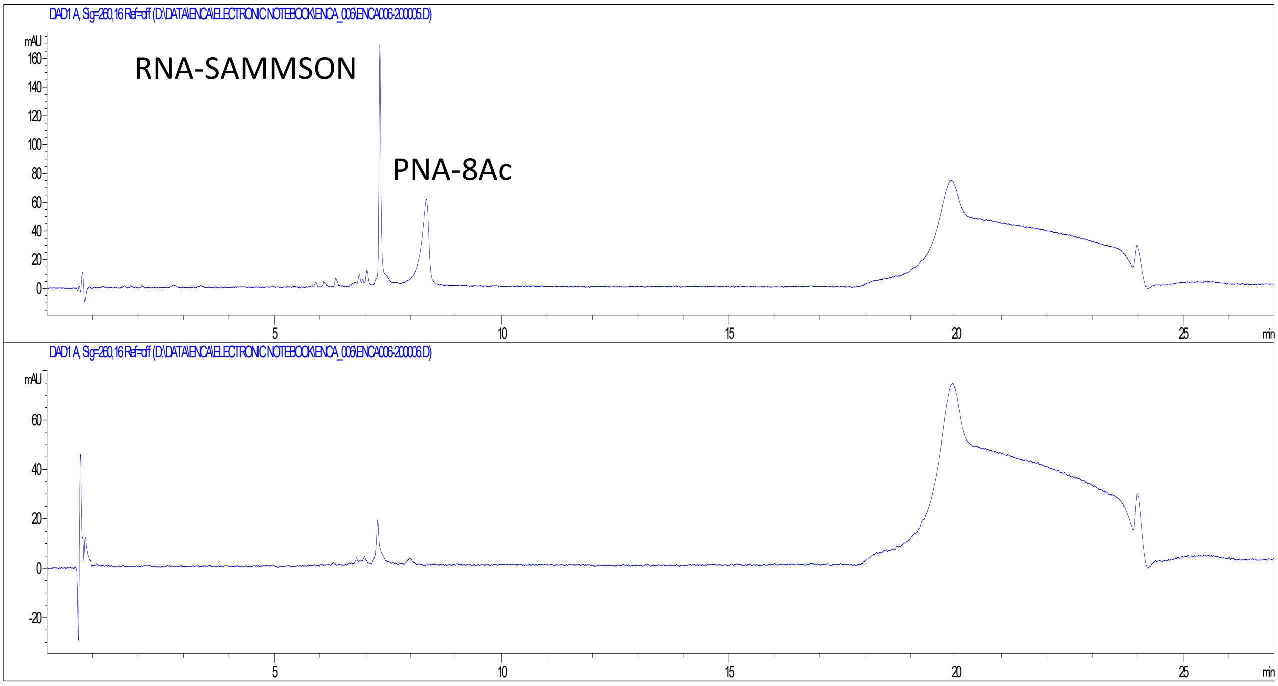

Figure S24. Pull down experiment of **RNA-SAMMSON** spiked in cell-lysate (SK-MEL-28, 55 million cells/mL), performed with **PNA-8Ac**. Top: starting solution; bottom: solution after pull down (incubation, washing and release by heating for 1h at 95°C).

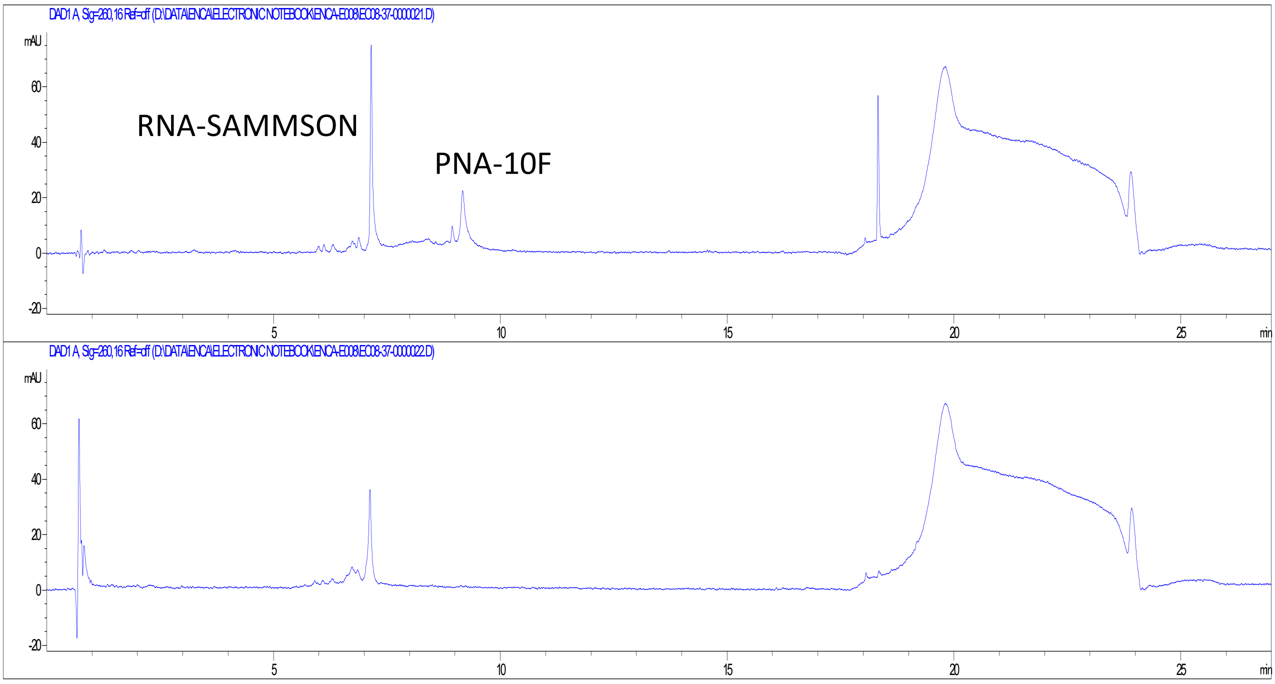

Figure S25. Pull down experiment of **RNA-SAMMSON** spiked in cell-lysate (SK-MEL-28, 55 million cells/mL), performed with **PNA-10F**. Top: starting solution; bottom: solution after pull down (incubation, washing and release by heating for 1h at 95°C).

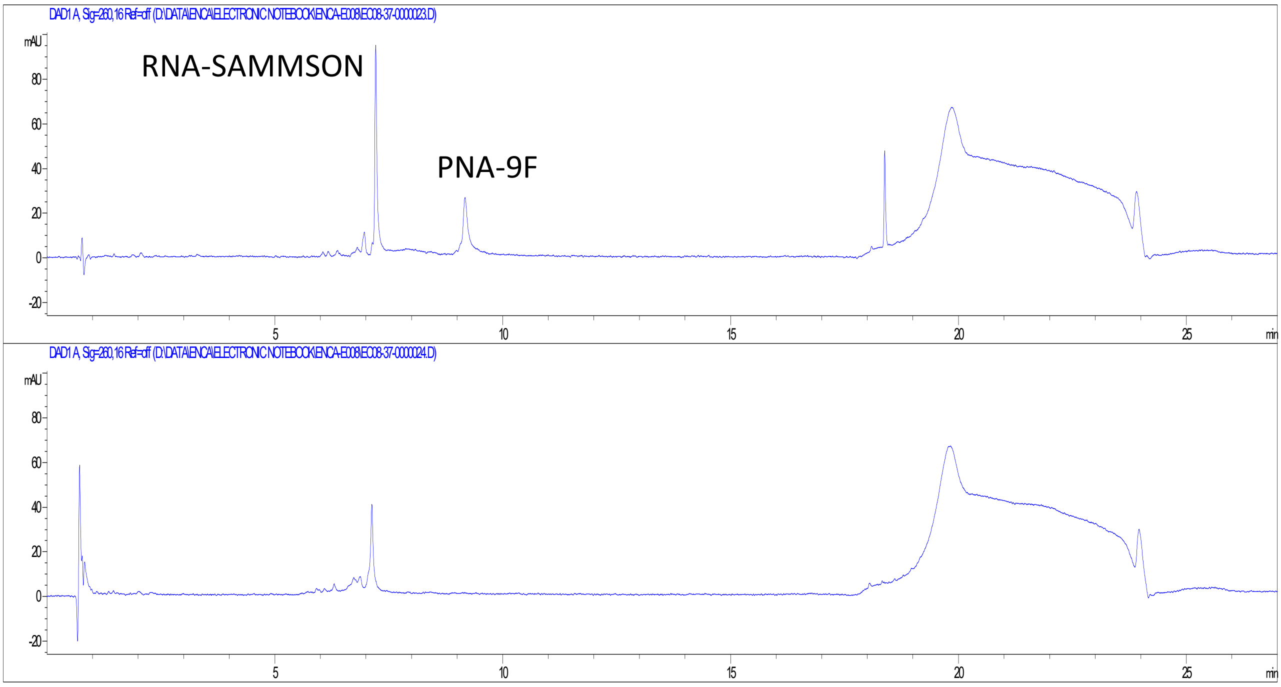

Figure S26. Pull down experiment of **RNA-SAMMSON** spiked in cell-lysate (SK-MEL-28, 55 million cells/mL), performed with **PNA-9F**. Top: starting solution; bottom: solution after pull down (incubation, washing and release by heating for 1h at 95°C).

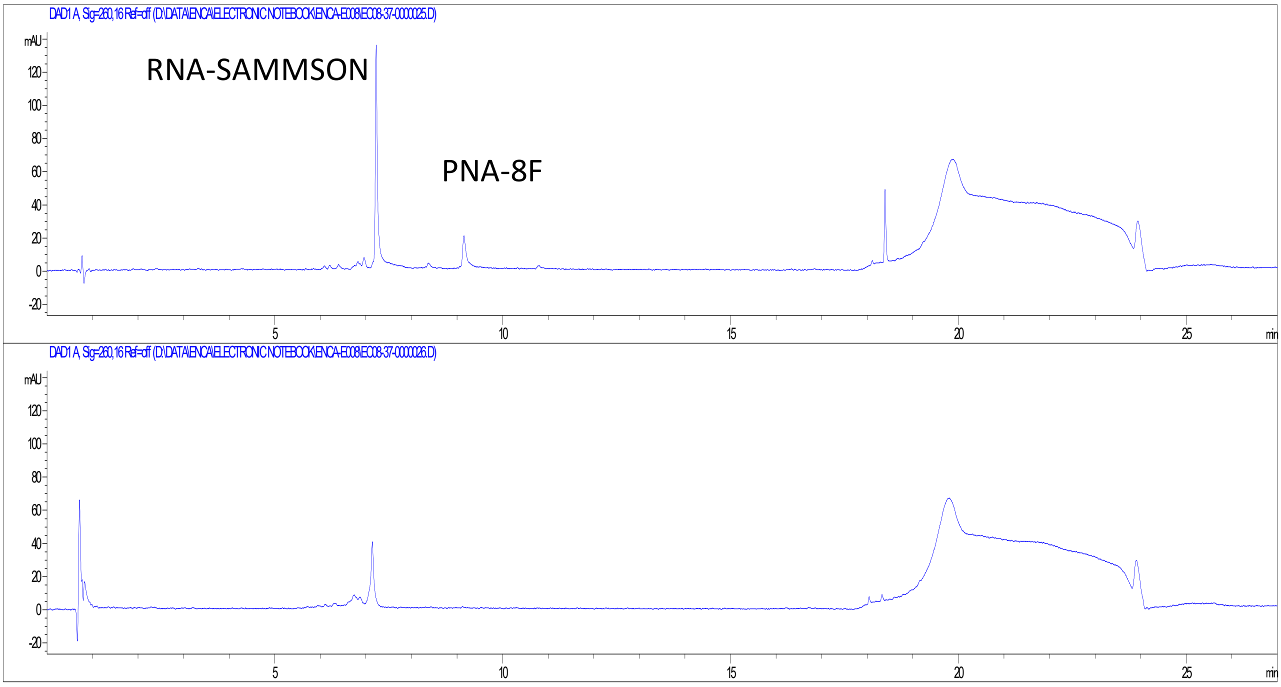

Figure S27. Pull down experiment of **RNA-SAMMSON** spiked in cell-lysate (SK-MEL-28, 55 million cells/mL), performed with **PNA-8F**. Top: starting solution; bottom: solution after pull down (incubation, washing and release by heating for 1h at 95°C).

8. Supporting References

1. Cadoni, E., Rosa-Gastaldo, D., Manicardi, A., Mancin, F., and Madder, A. (2020). Exploiting Double Exchange Diels-Alder Cycloadditions for Immobilization of Peptide Nucleic Acids on Gold Nanoparticles. Front. Chem. *8*.
2. Ausín, C., Ortega, J.-A., Robles, J., Grandas, A., and Pedroso, E. (2002). Synthesis of Amino- and Guanidino-G-Clamp PNA Monomers. Org. Lett. *4*, 4073–4075.
3. Elskens, J., Manicardi, A., Costi, V., Madder, A., and Corradini, R. (2017). Synthesis and Improved Cross-Linking Properties of C5-Modified Furan Bearing PNAs. Molecules *22*, 2010.
4. Manicardi, A., Gyssels, E., Corradini, R., and Madder, A. (2016). Furan-PNA: a mildly inducible irreversible interstrand crosslinking system targeting single and double stranded DNA. Chem. Commun. *52*, 6930–6933.

9. NMR spectra

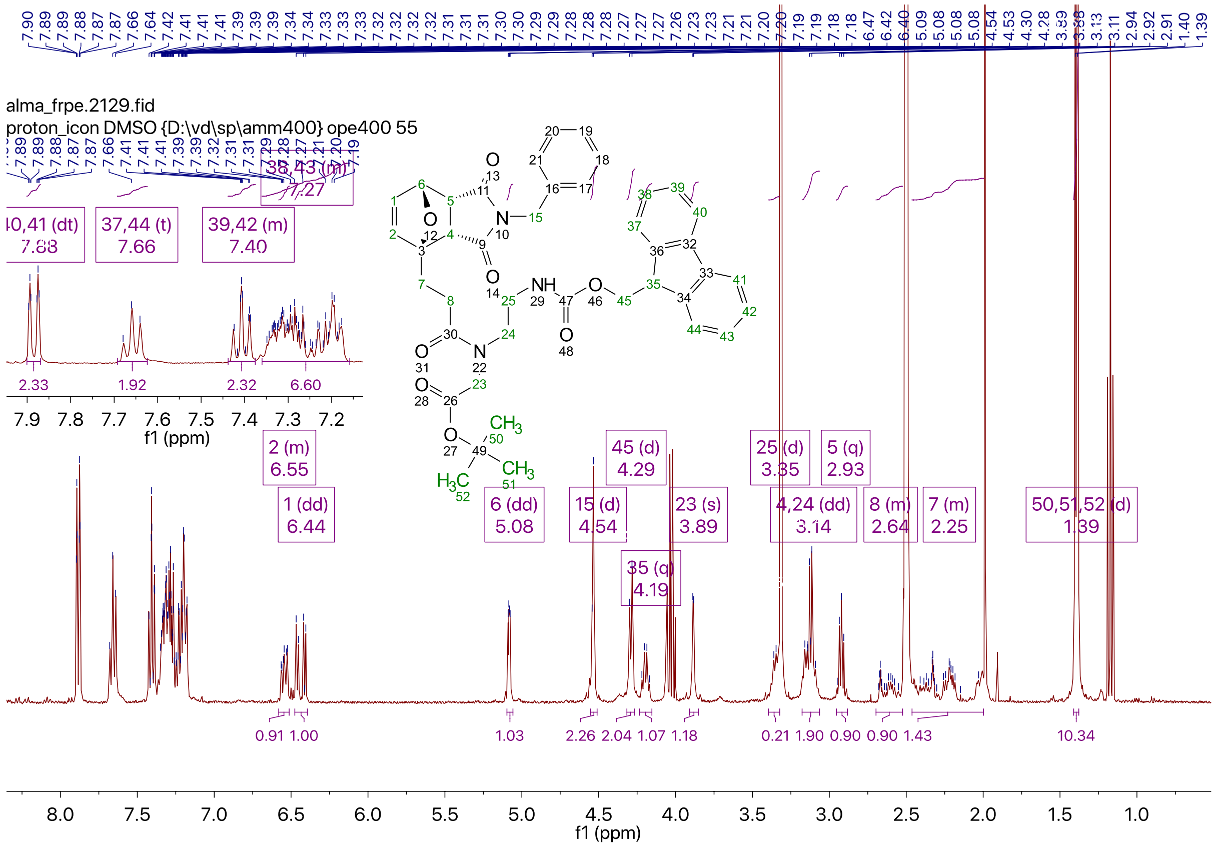

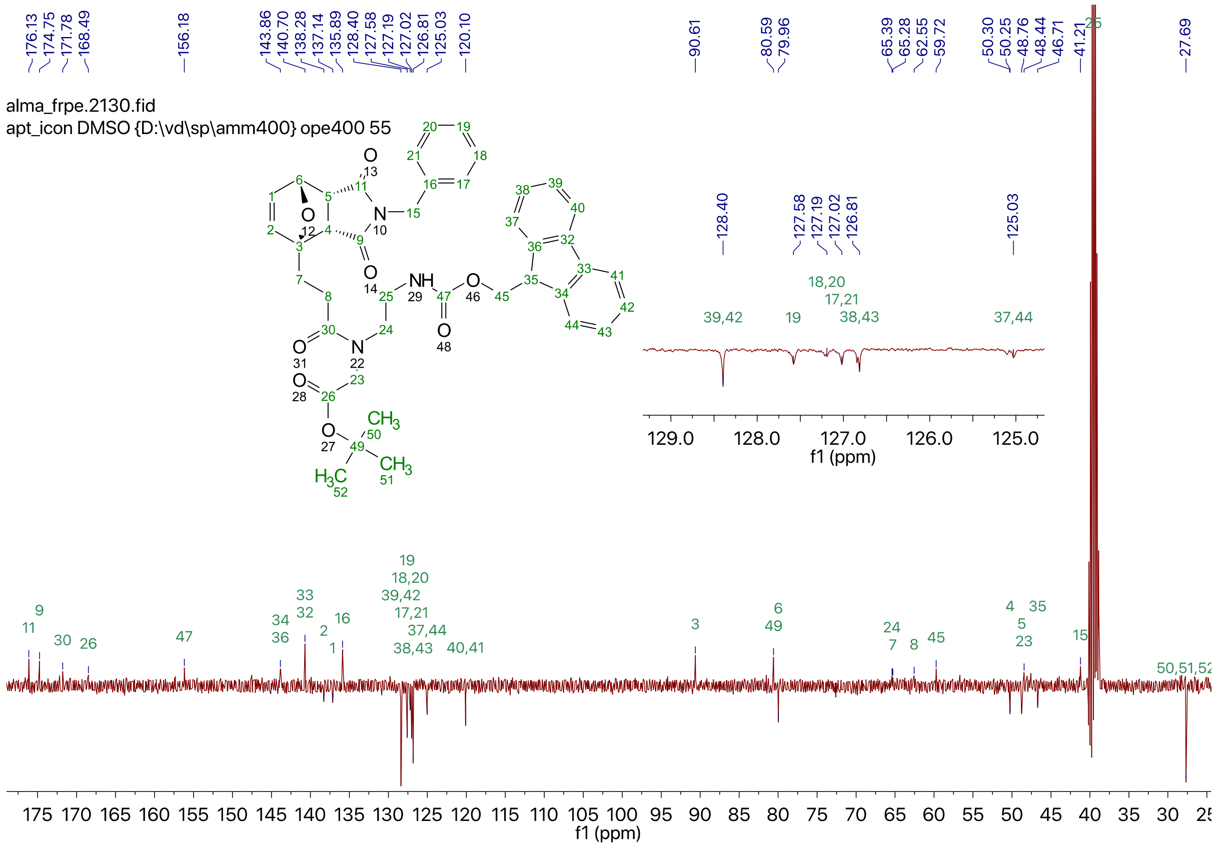

Figure S28: ^{1}H-NMR and ^{13}C-NMR (APT) spectra of **8**.

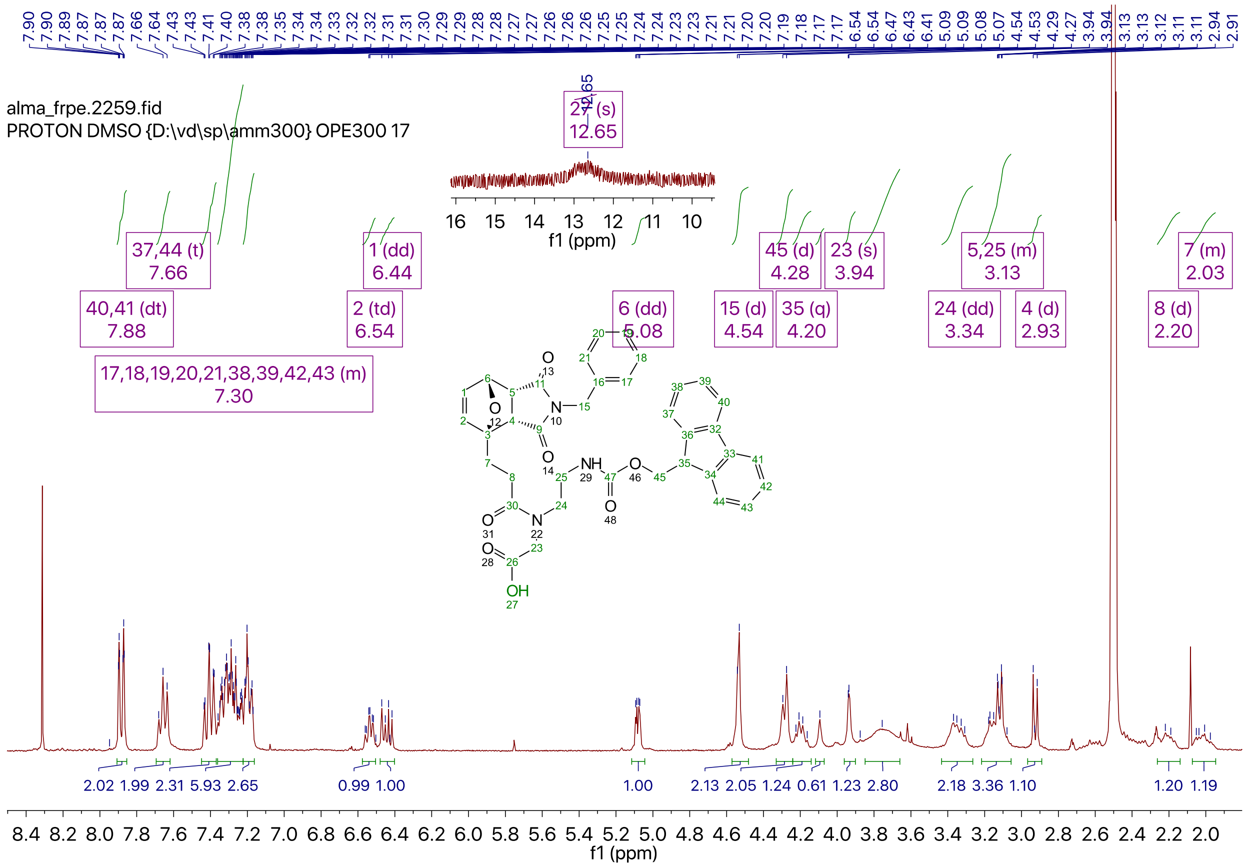

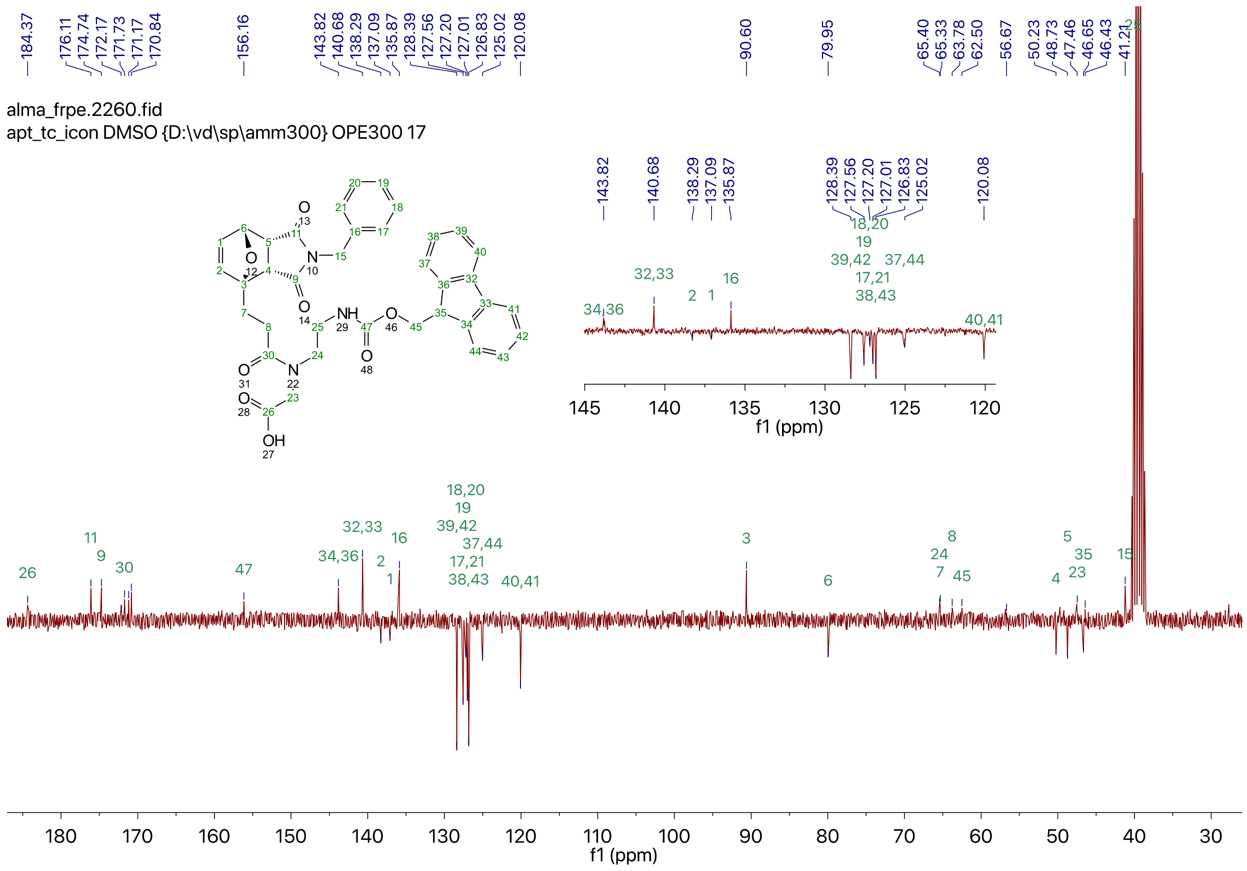

Figure S29: ^{1}H-NMR and ^{13}C-NMR (APT) spectra of **M1**.

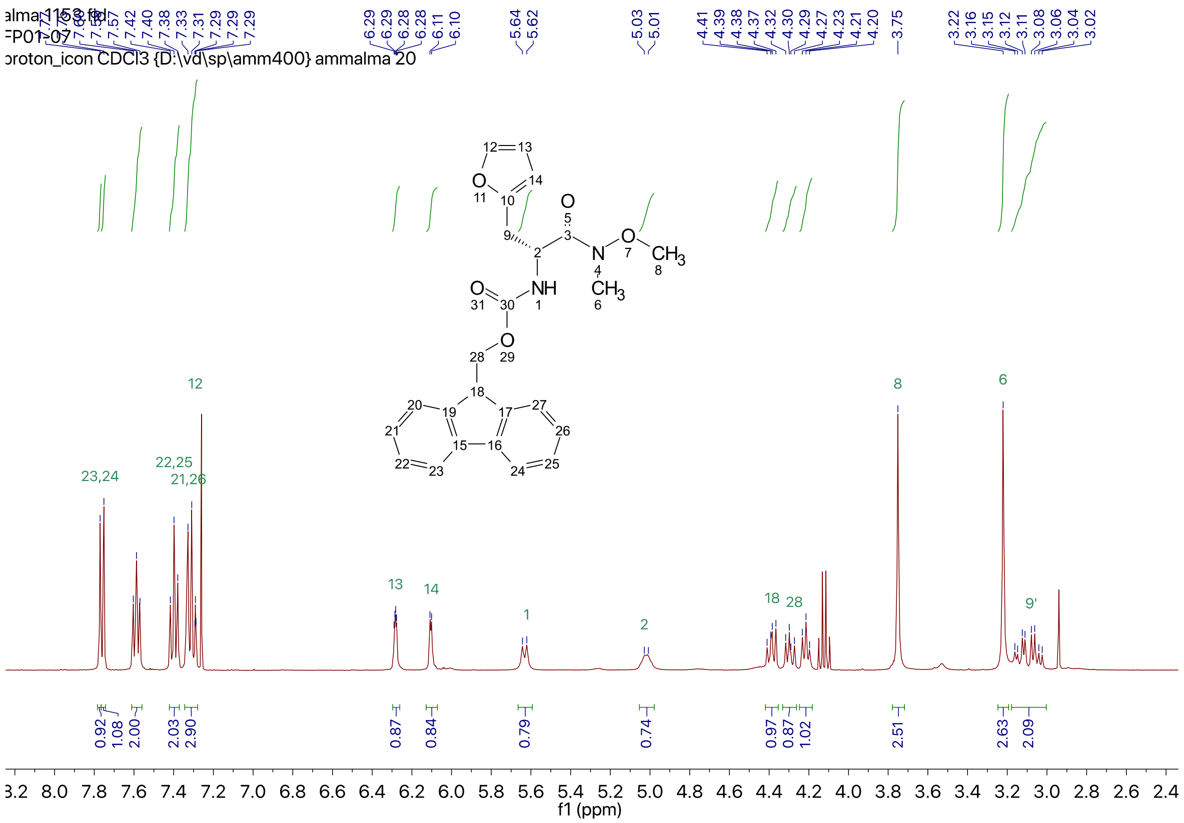

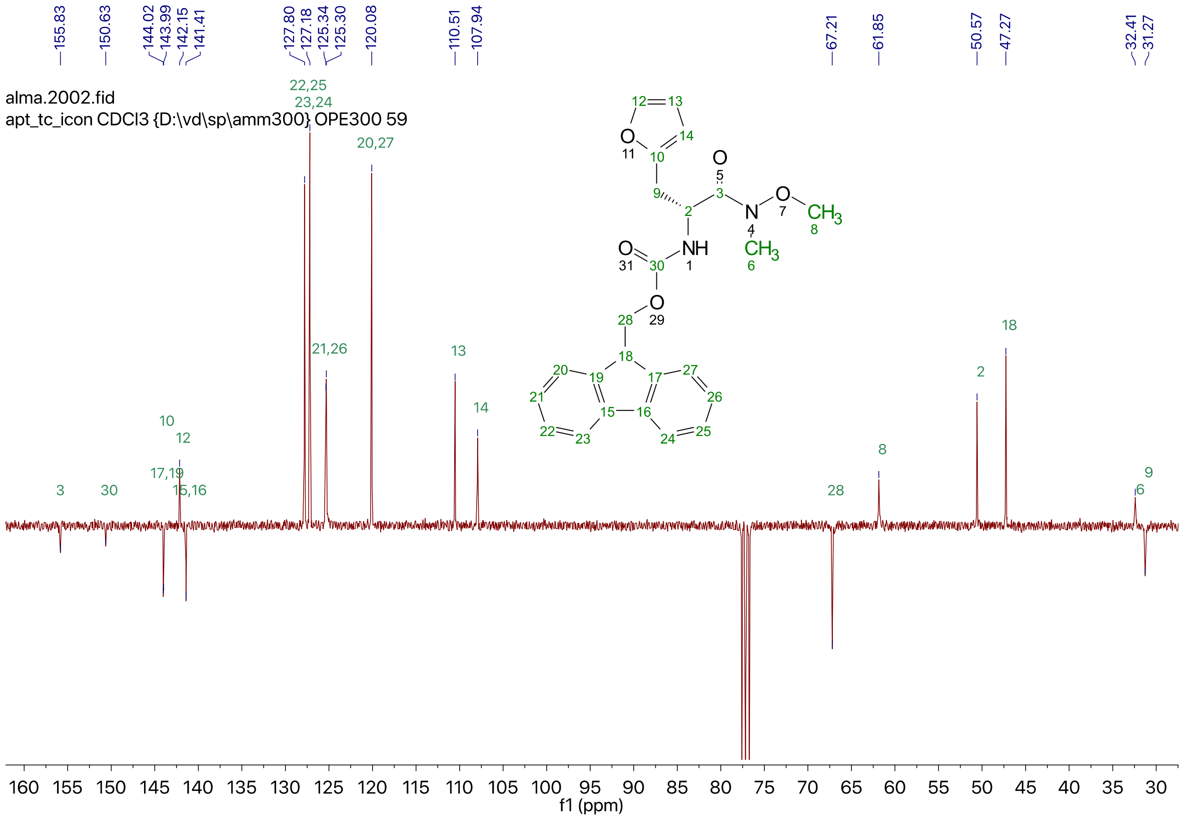

Figure S30: ^{1}H-NMR and ^{13}C-NMR (APT) spectra of **10**.

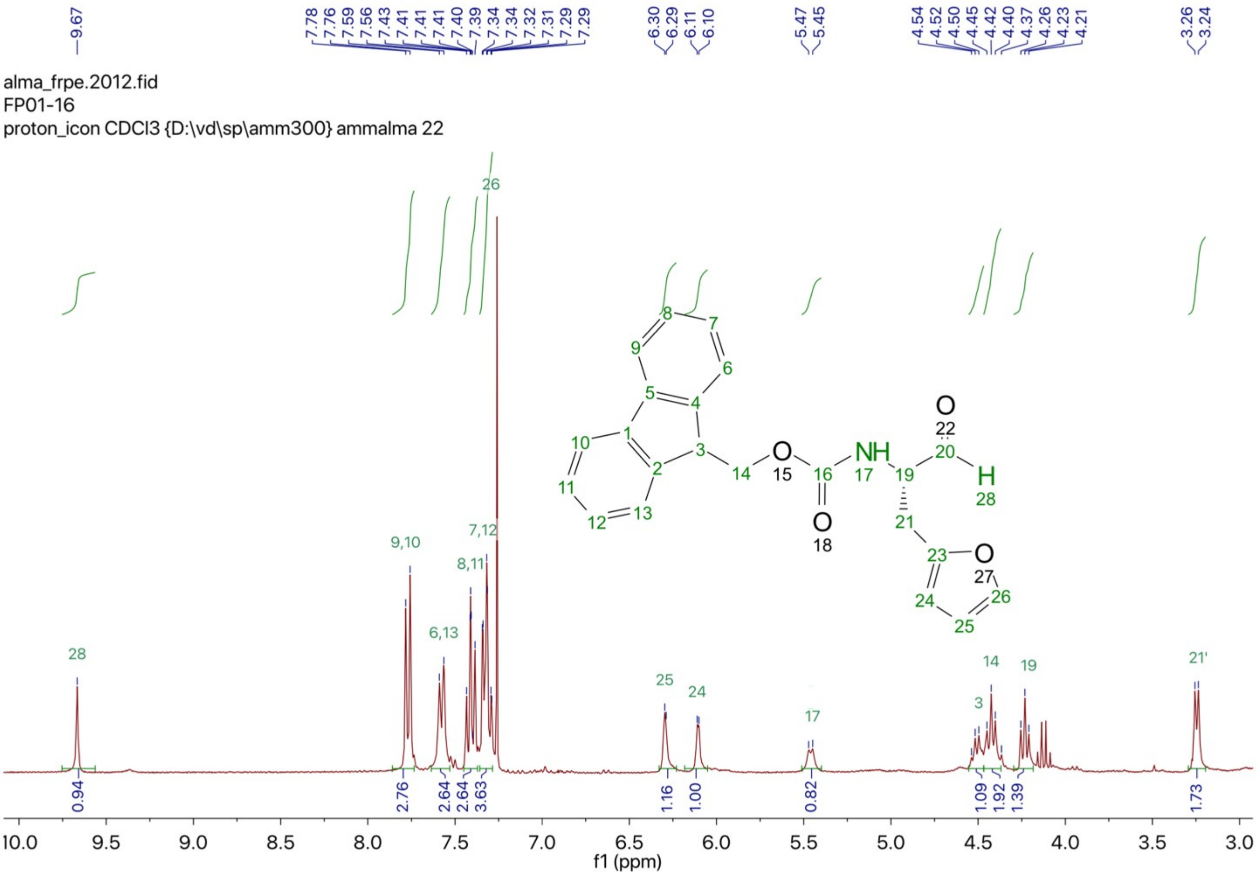

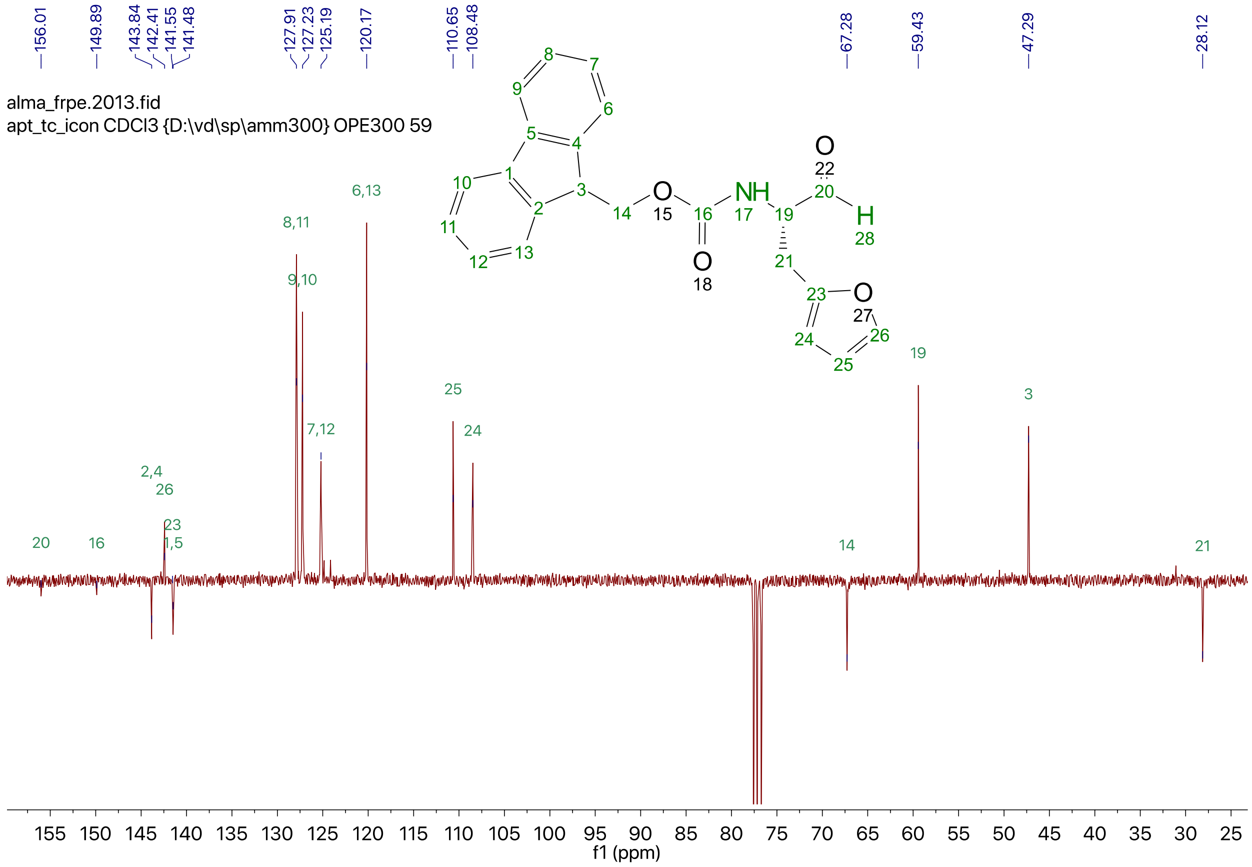

Figure S31: ^{1}H-NMR and ^{13}C-NMR (APT) spectra of **11**.

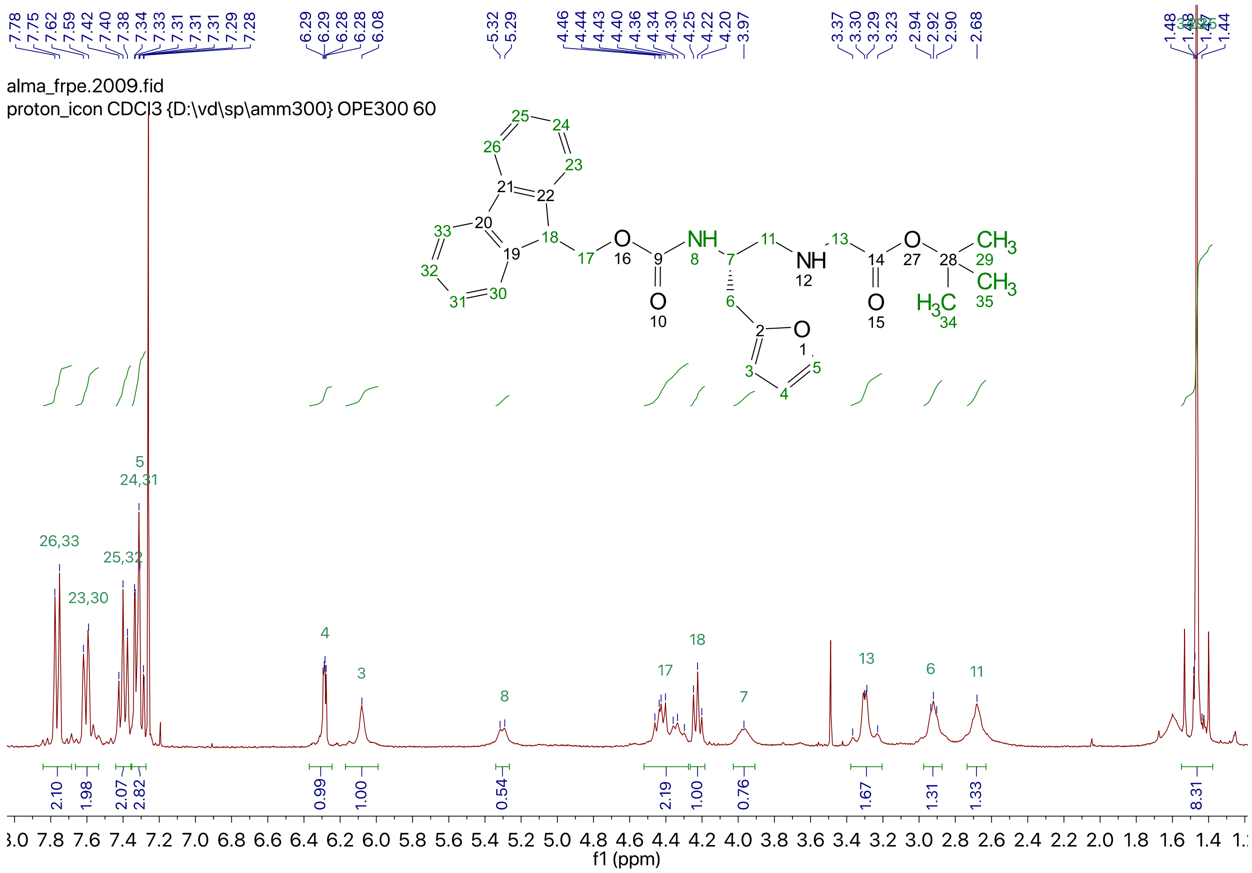

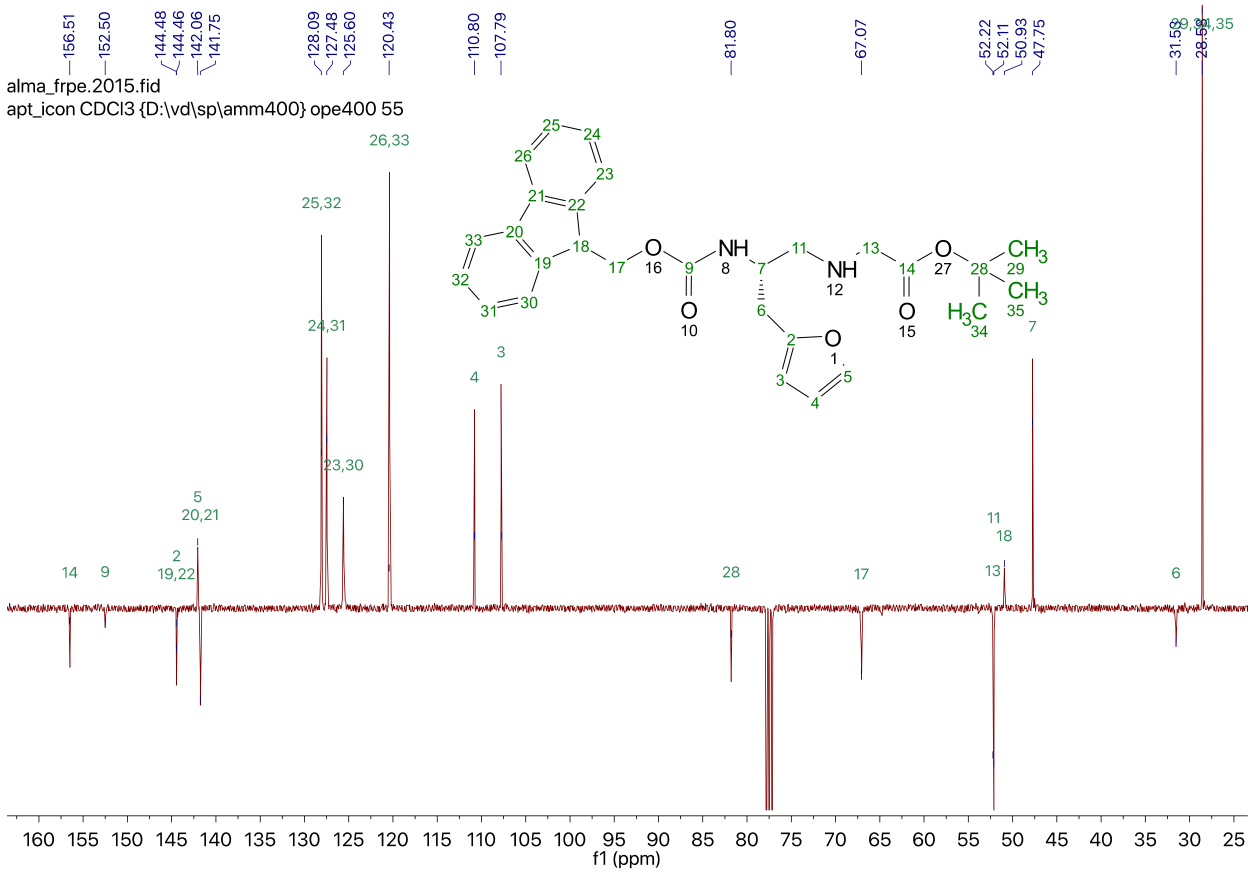

Figure S32: ^{1}H-NMR and ^{13}C-NMR (APT) spectra of **12**.

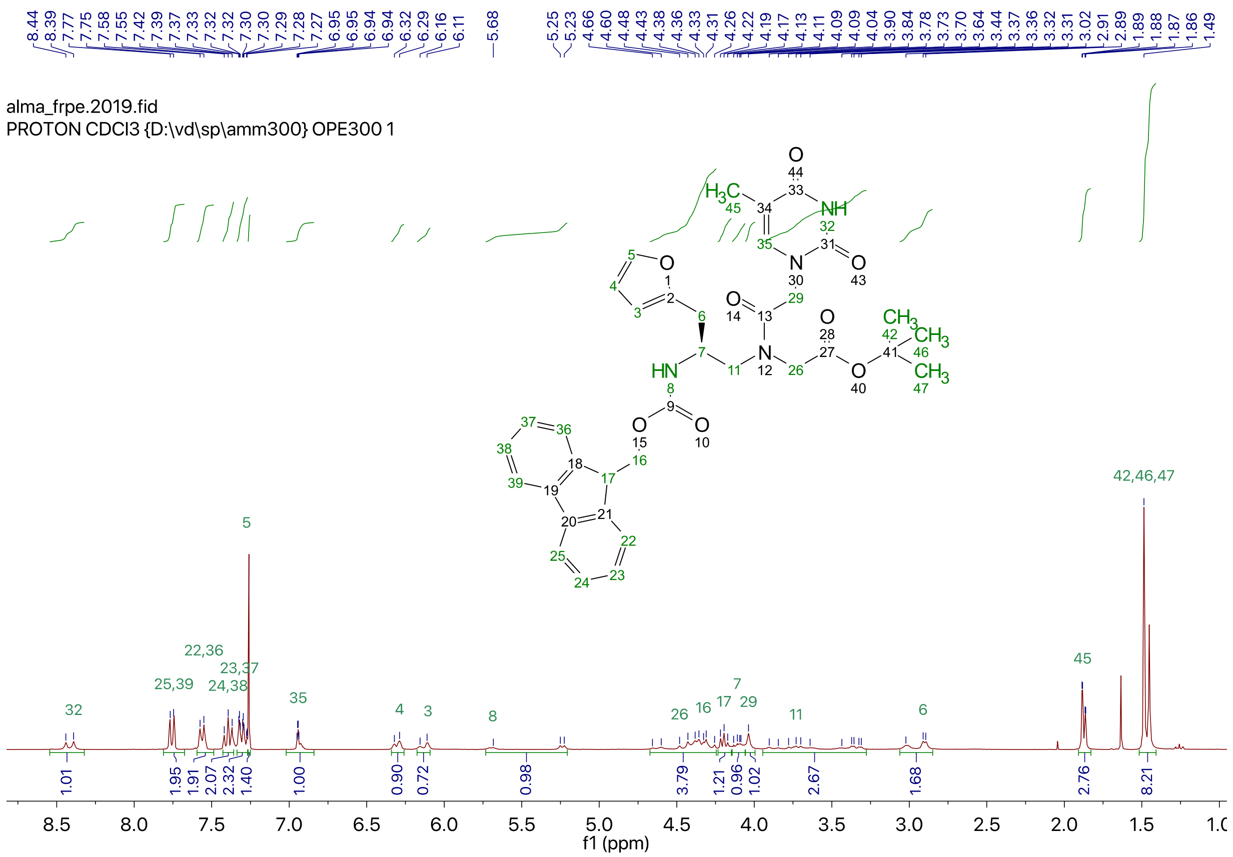

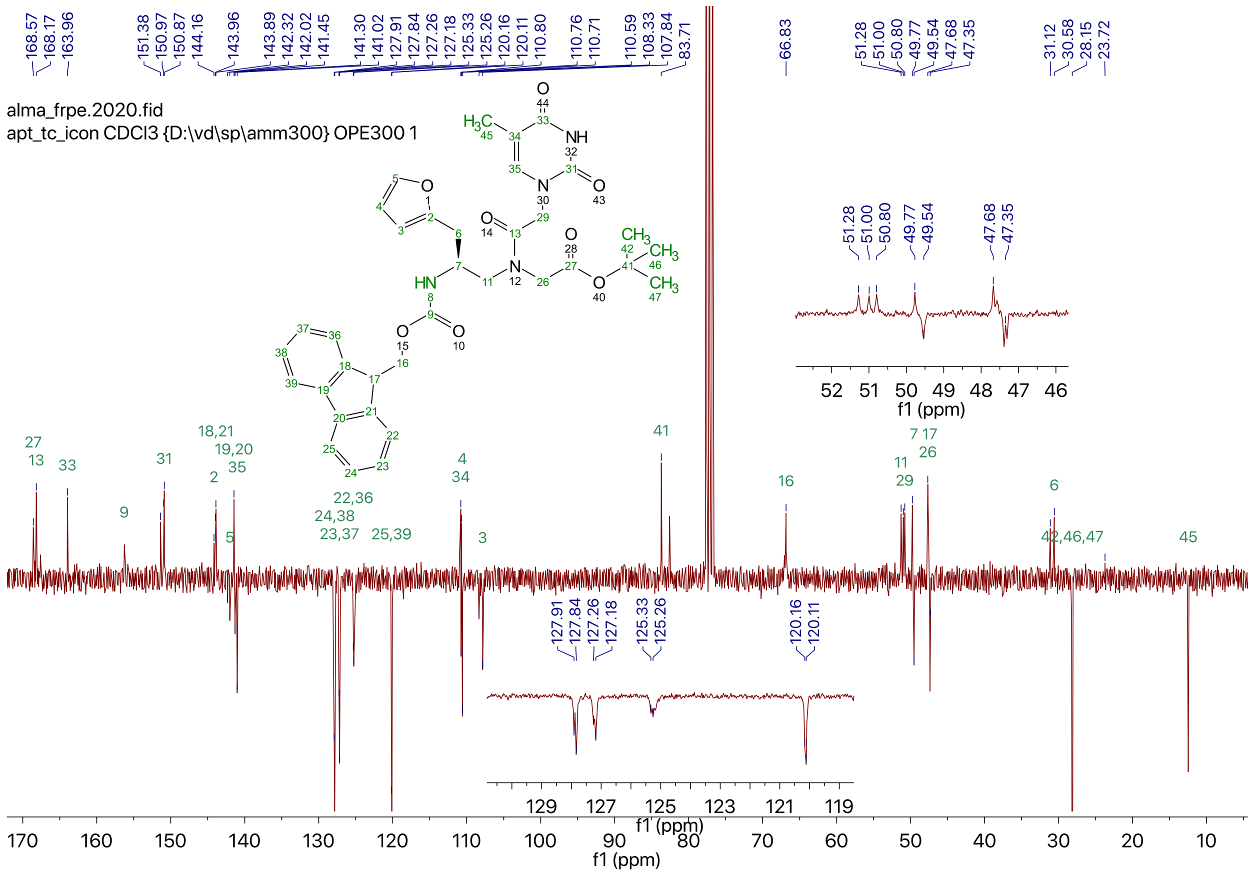

Figure S33: ^{1}H-NMR and ^{13}C-NMR (APT) spectra of **13**.

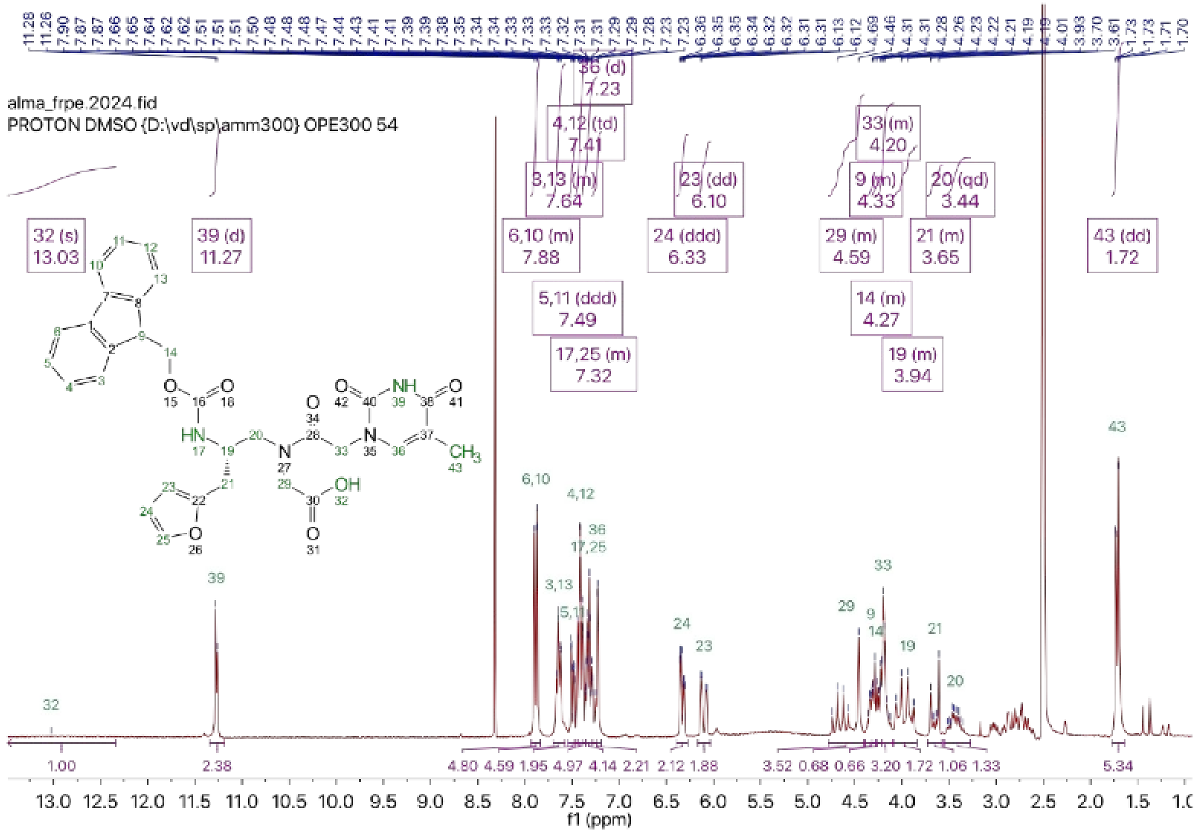

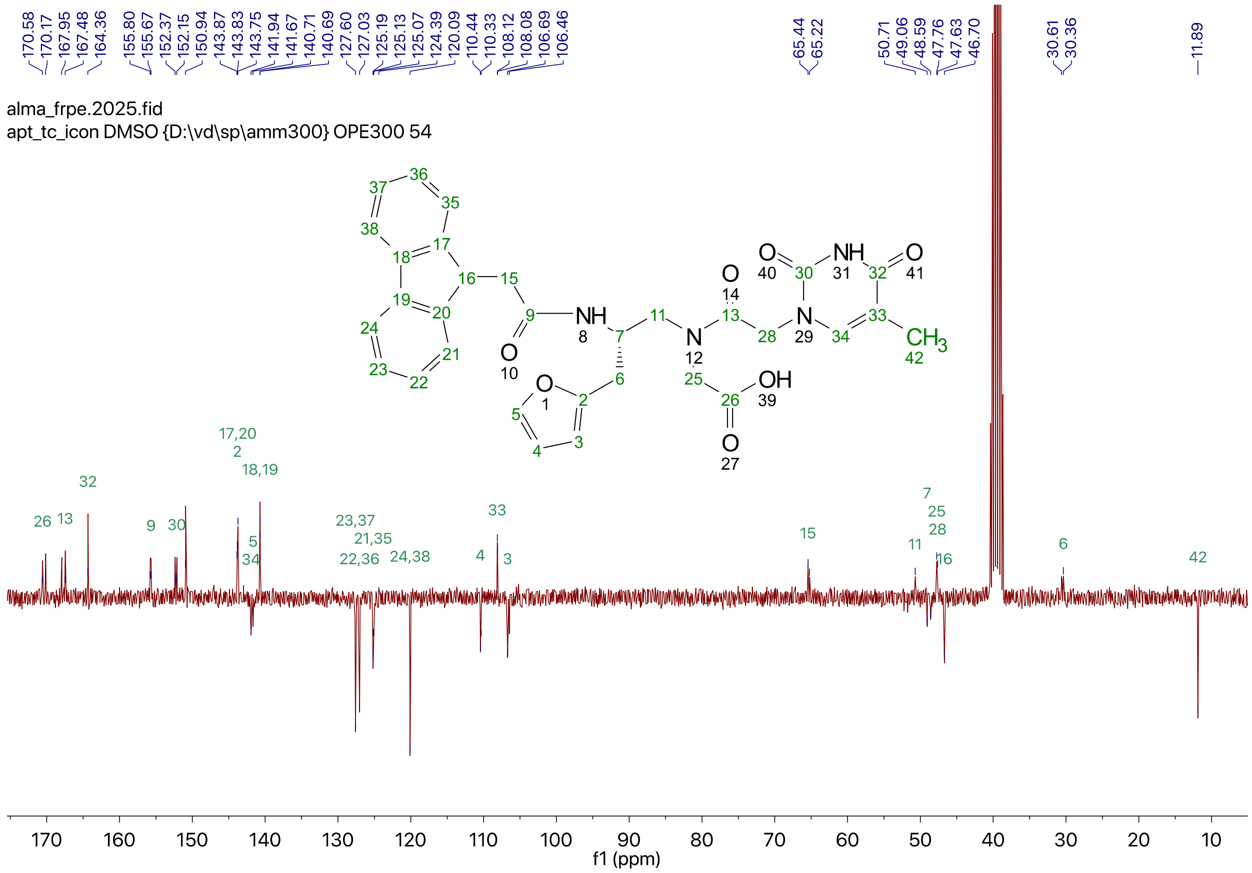

Figure S34: ^{1}H-NMR and ^{13}C-NMR (APT) spectra of **M2**.

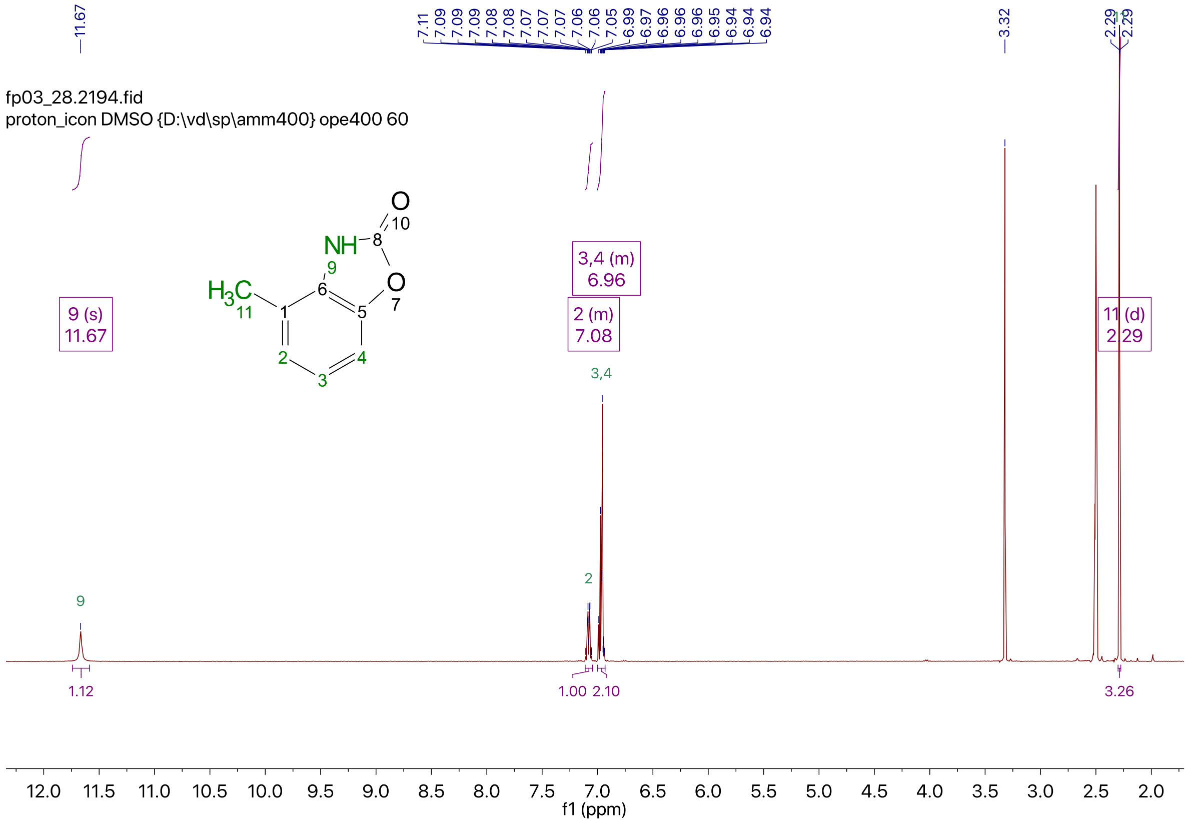

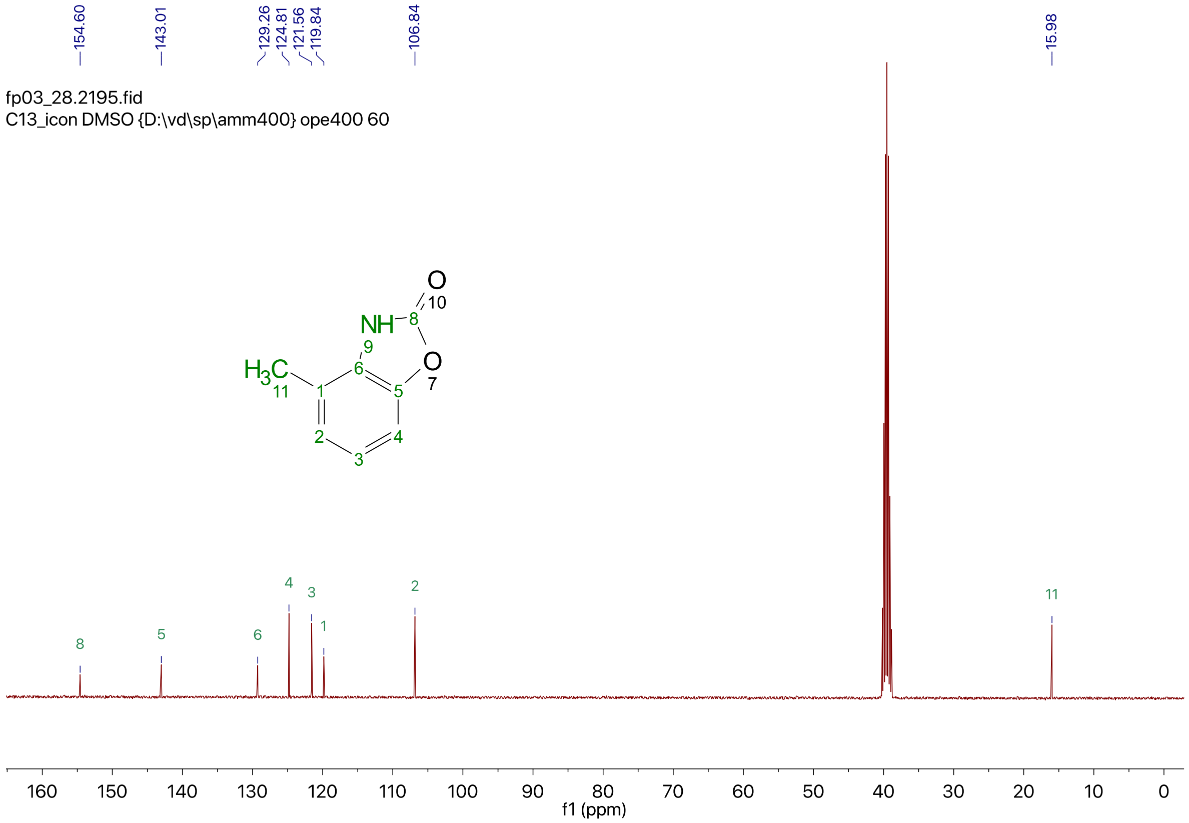

Figure S35: ^{1}H-NMR and ^{13}C-NMR spectra of **15**.

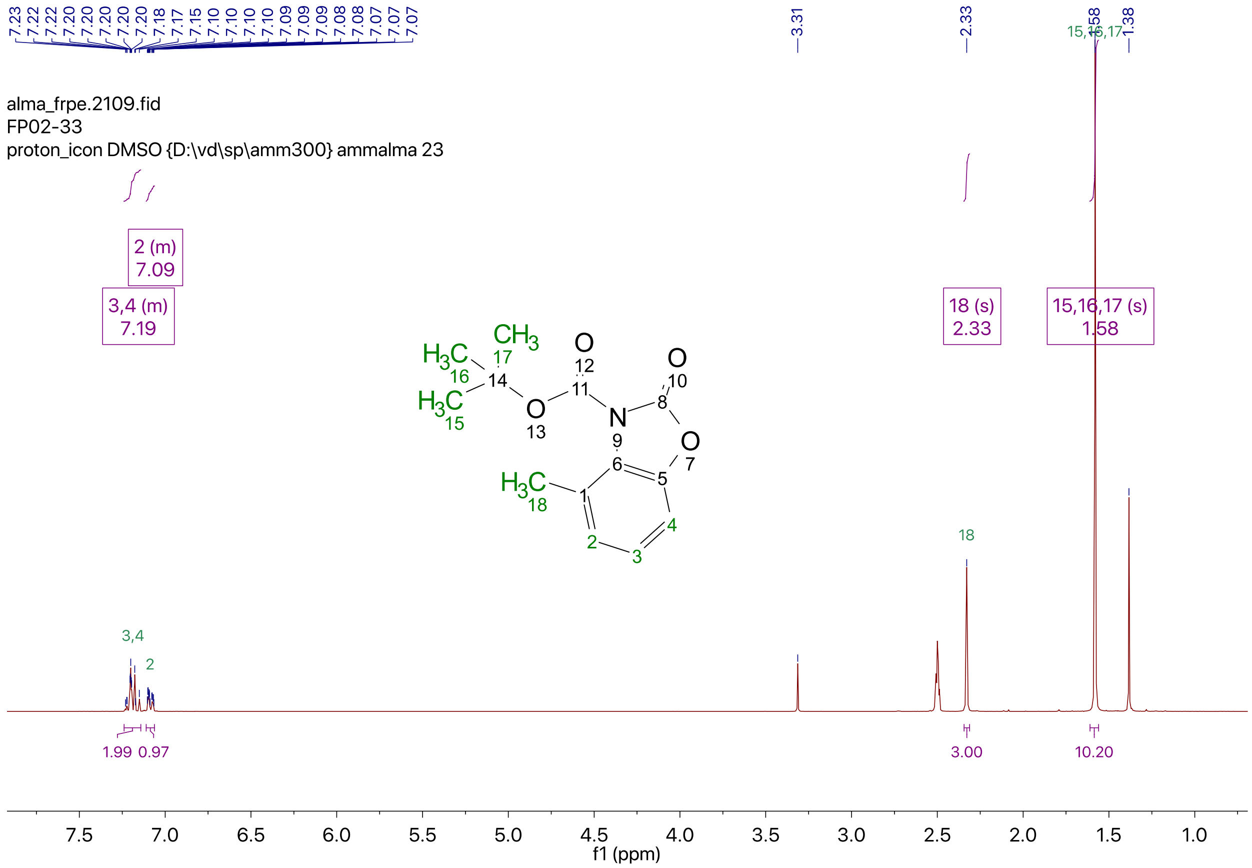

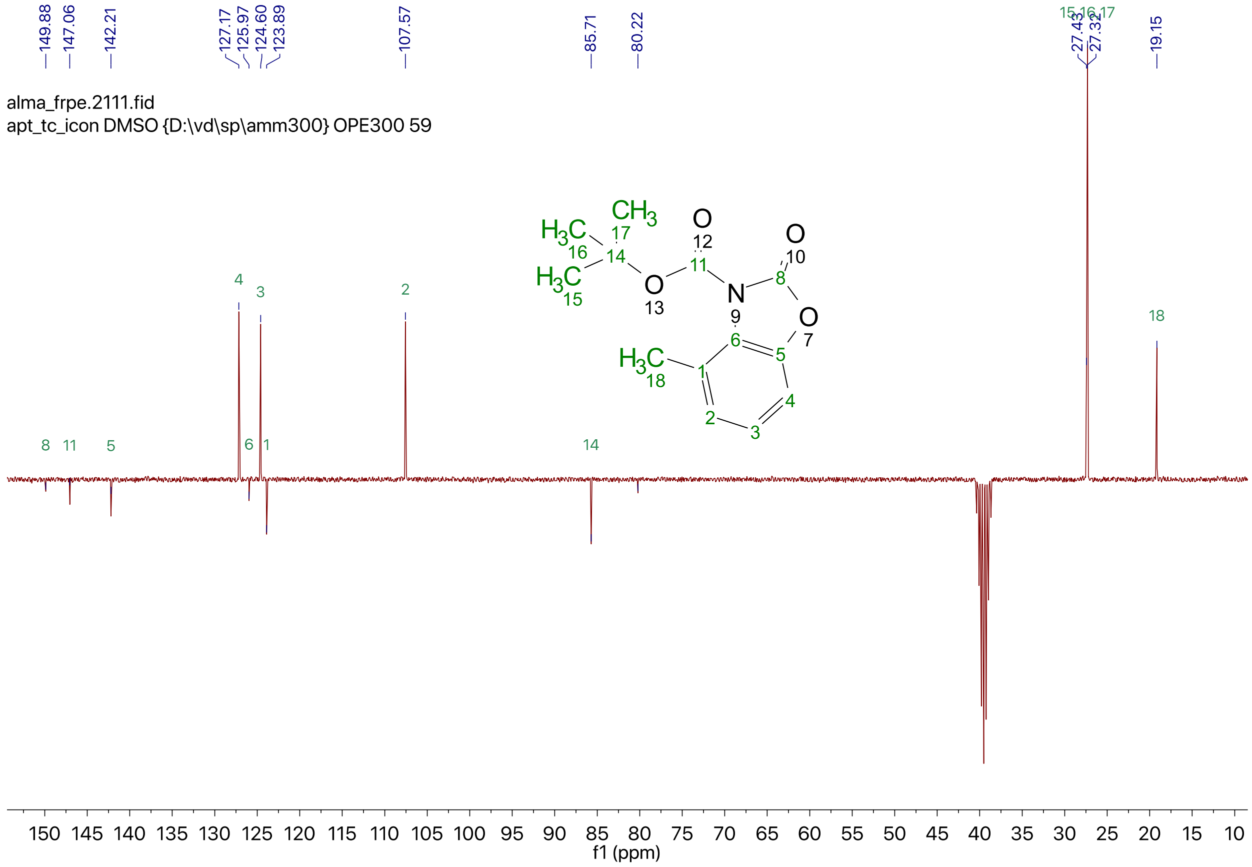

Figure S36: ^{1}H-NMR and ^{13}C-NMR (APT) spectra of **16**.

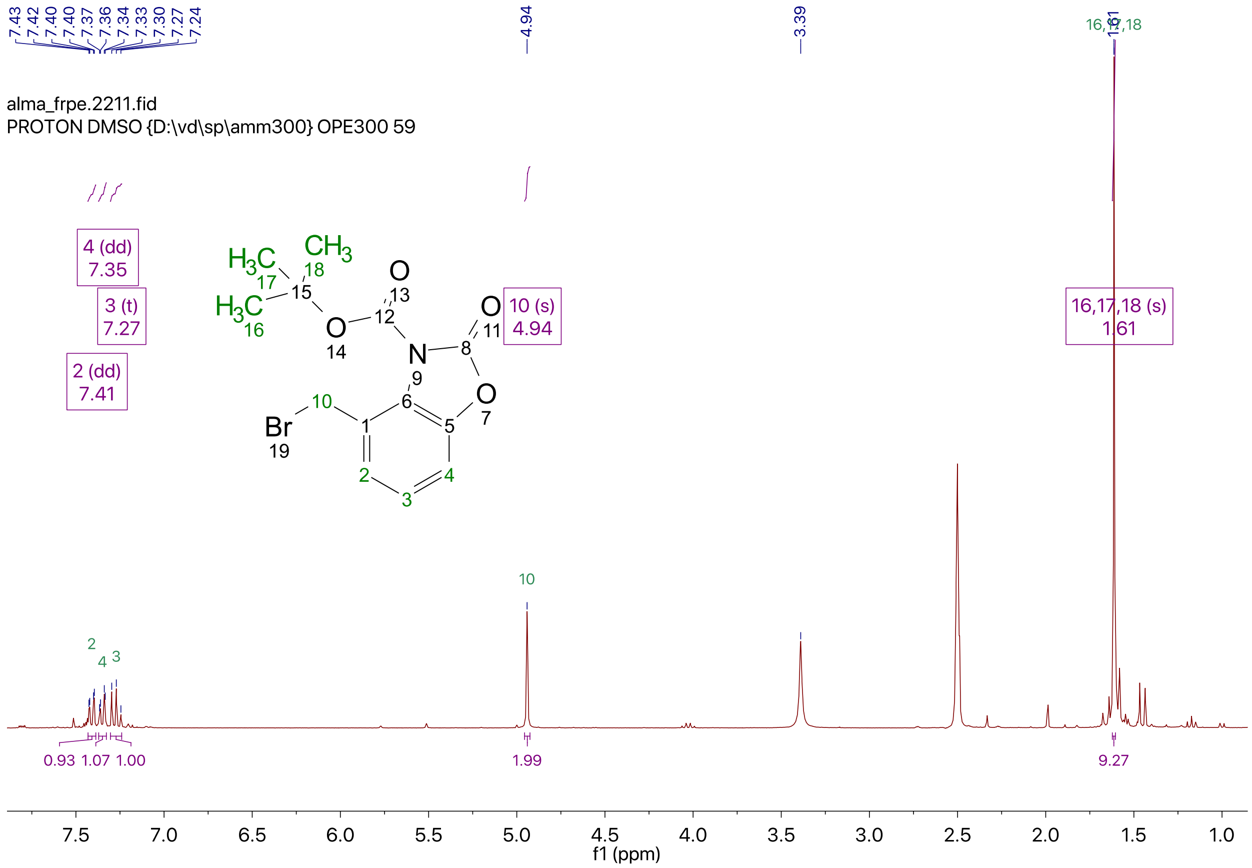

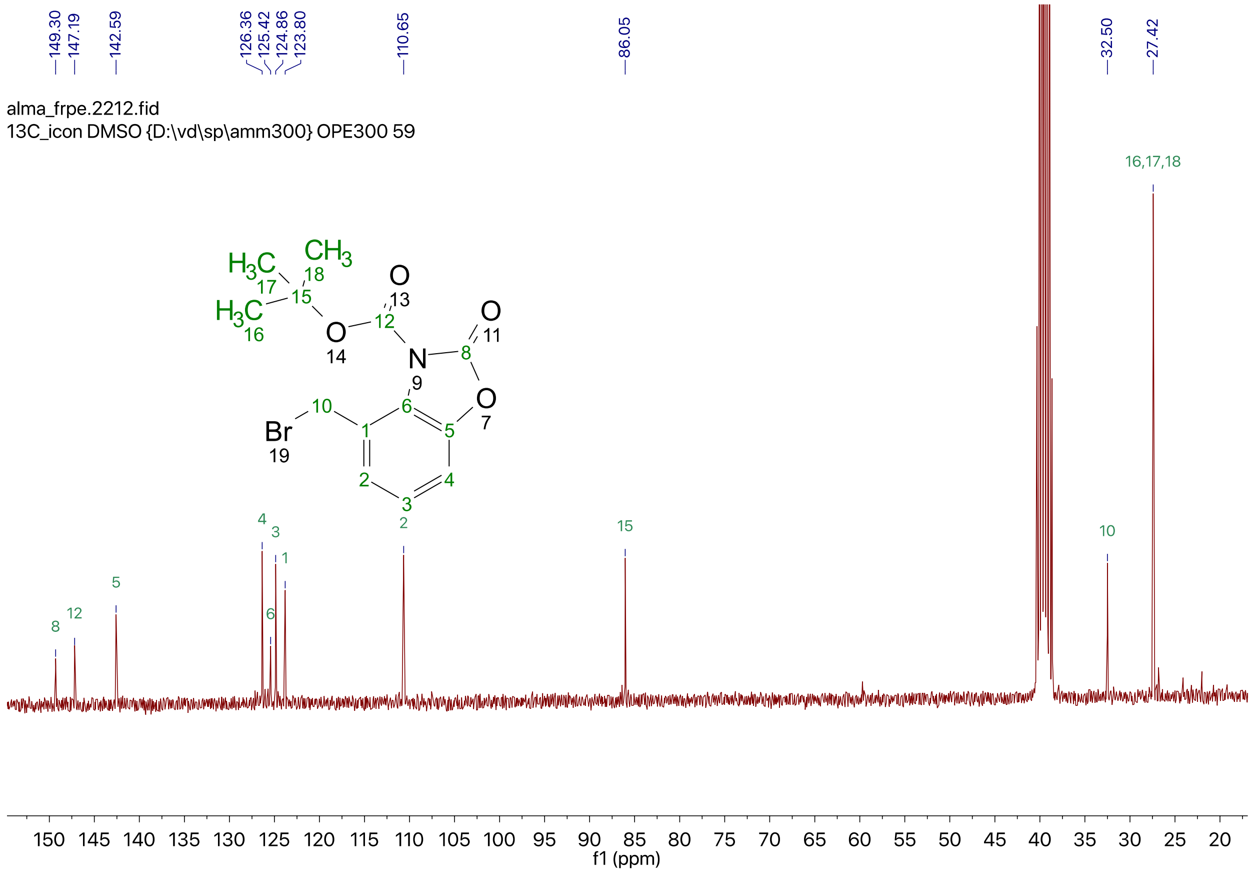

Figure S37: ^{1}H-NMR and ^{13}C-NMR spectra of **17**.

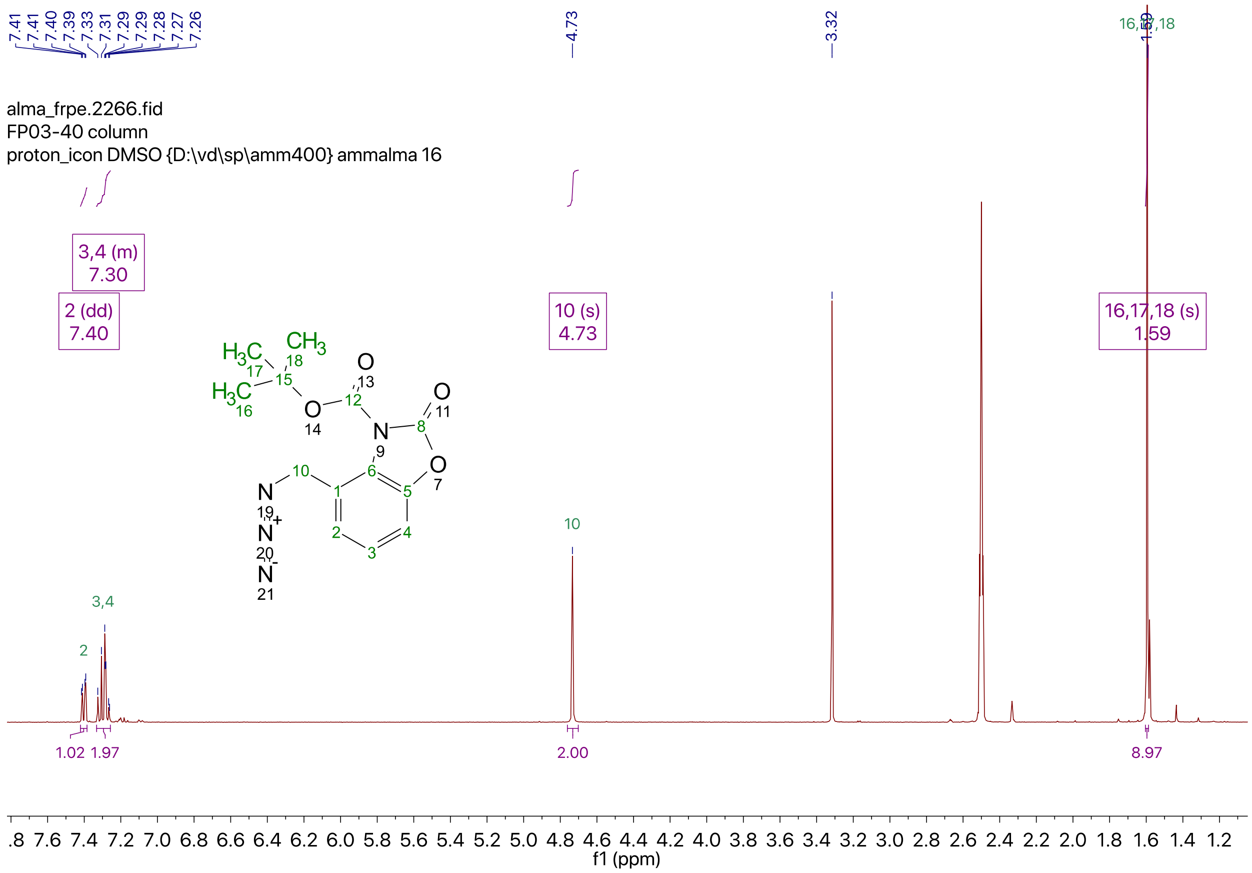

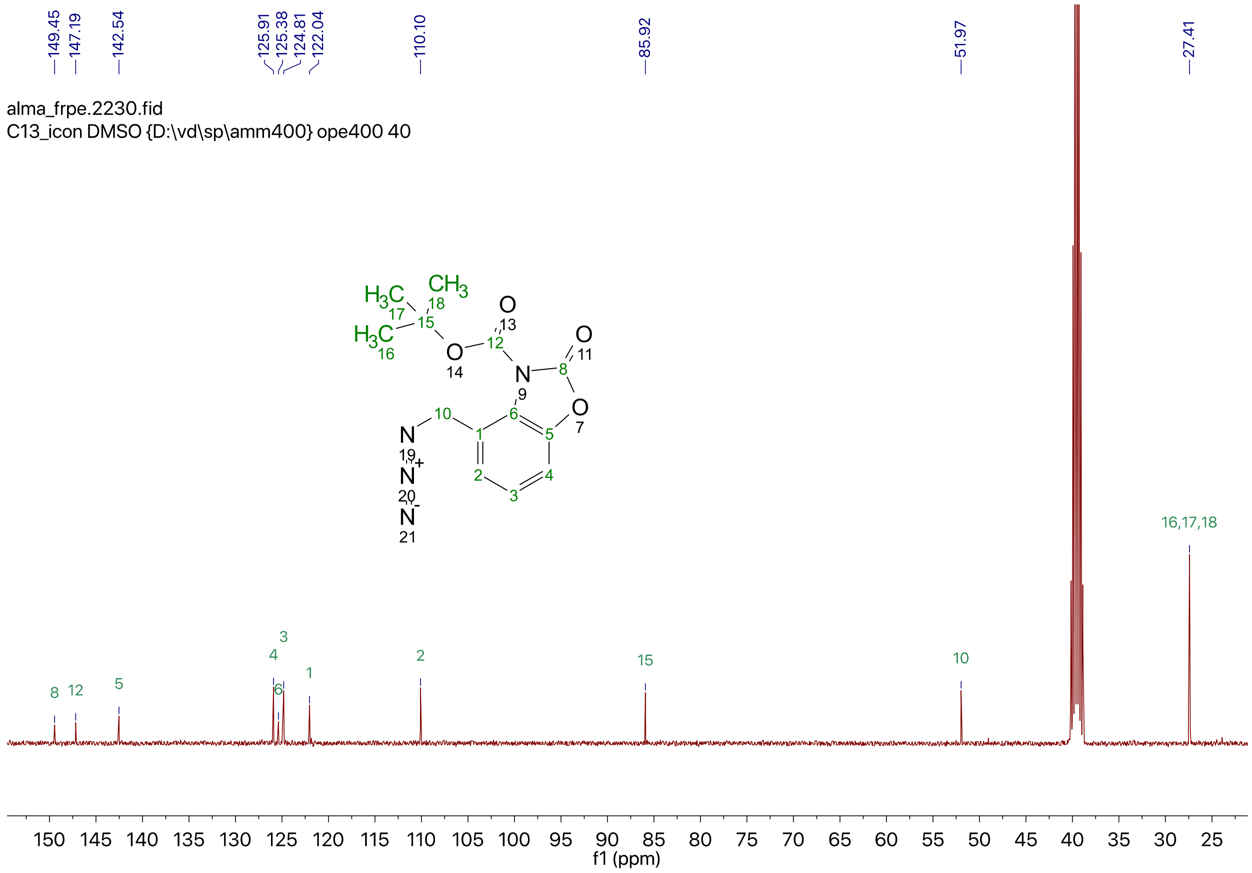

Figure S38: ^{1}H-NMR and ^{13}C-NMR spectra of **18**.

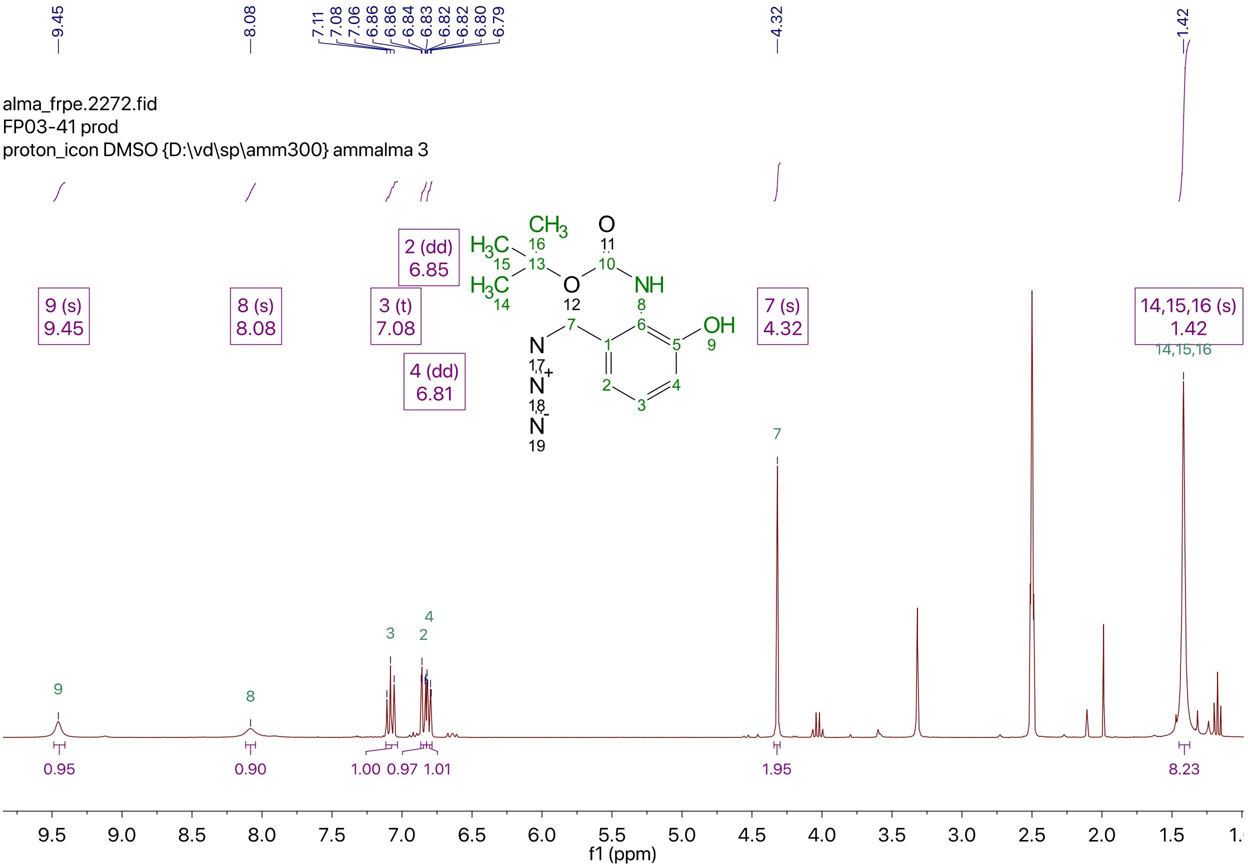

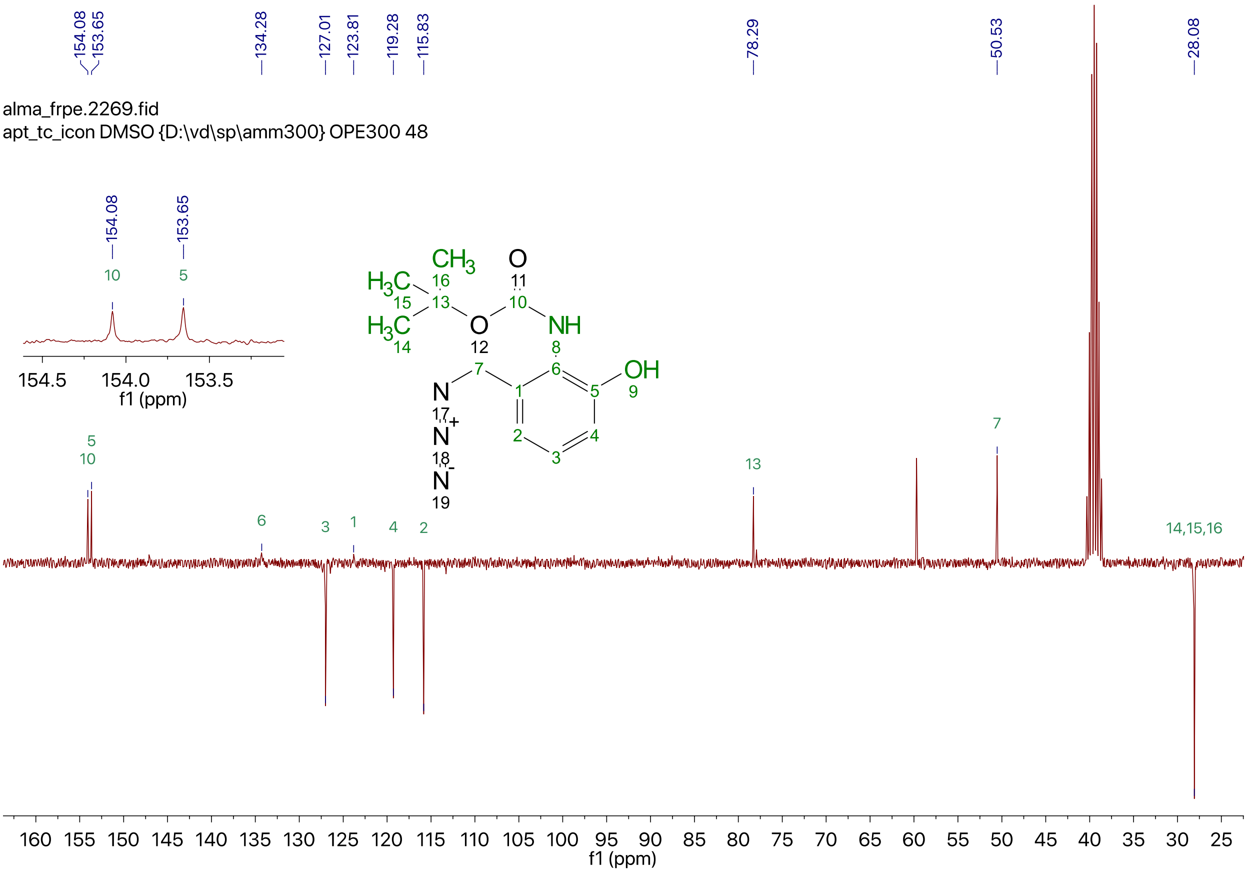

Figure S39: ^{1}H-NMR and ^{13}C-NMR (APT) spectra of **19**.

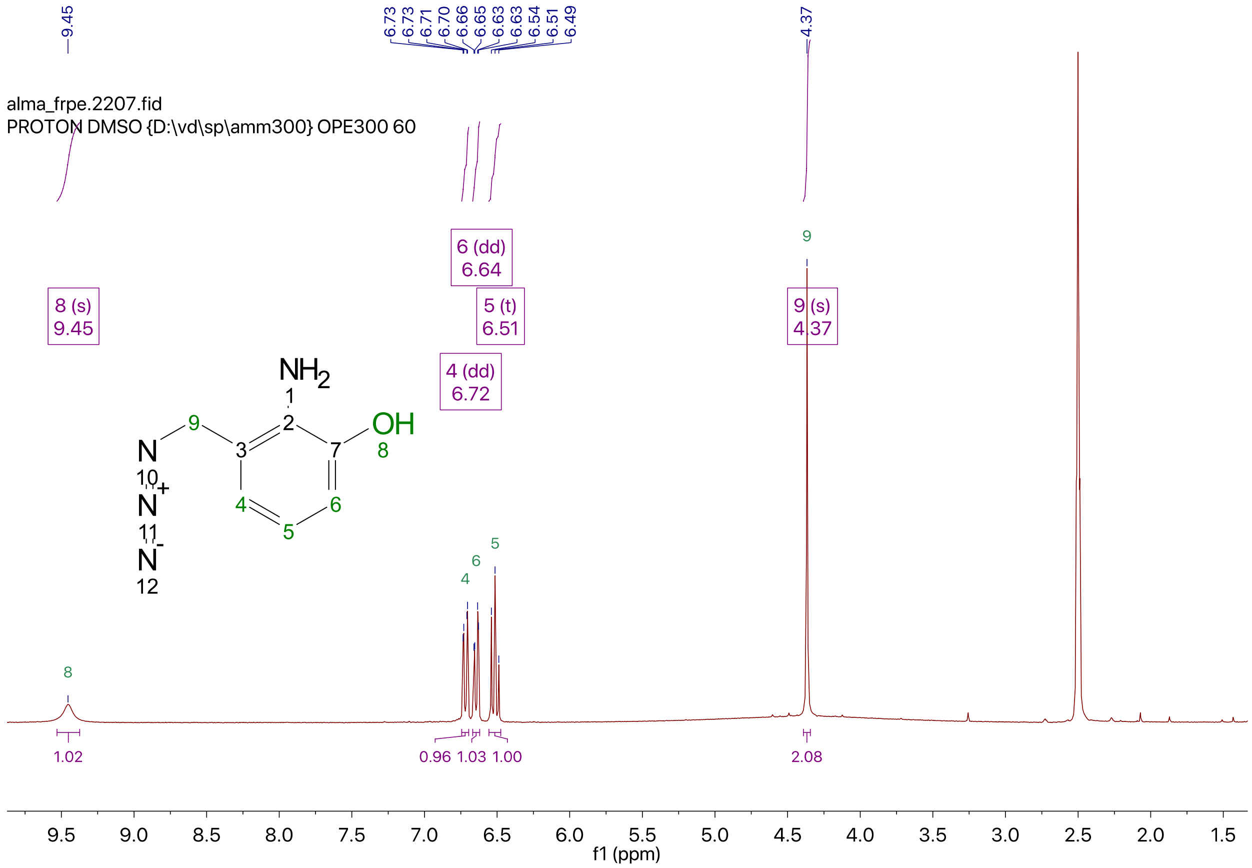

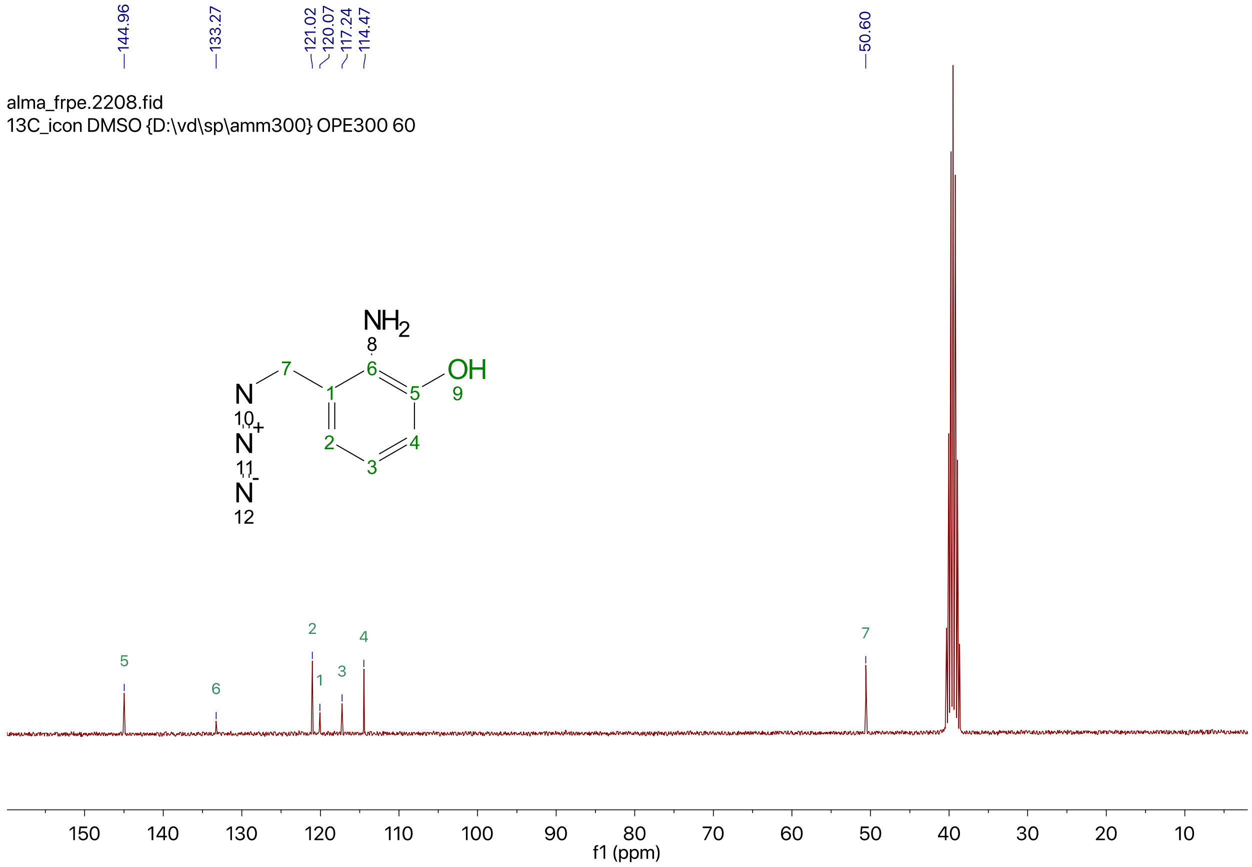

Figure S40: ^{1}H-NMR and ^{13}C-NMR spectra of **20**.

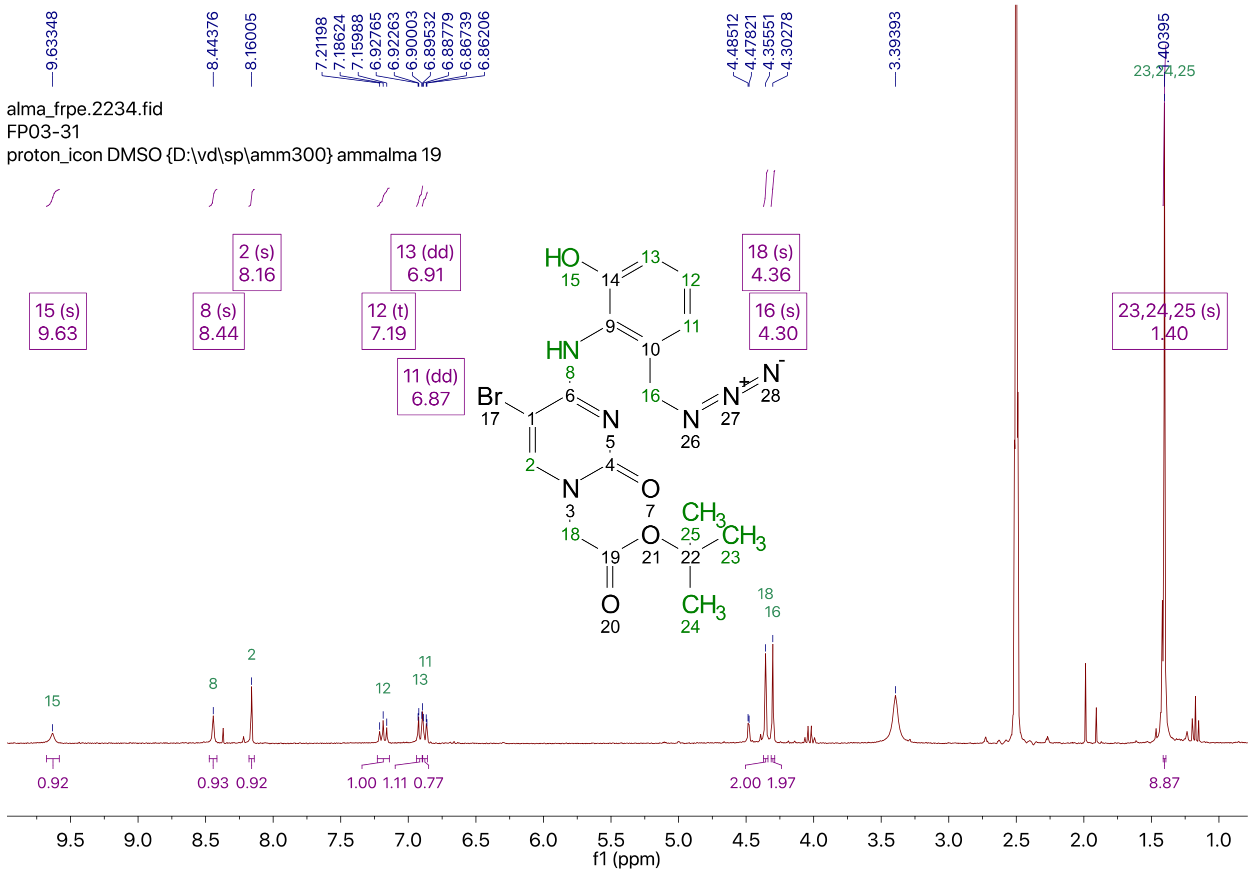

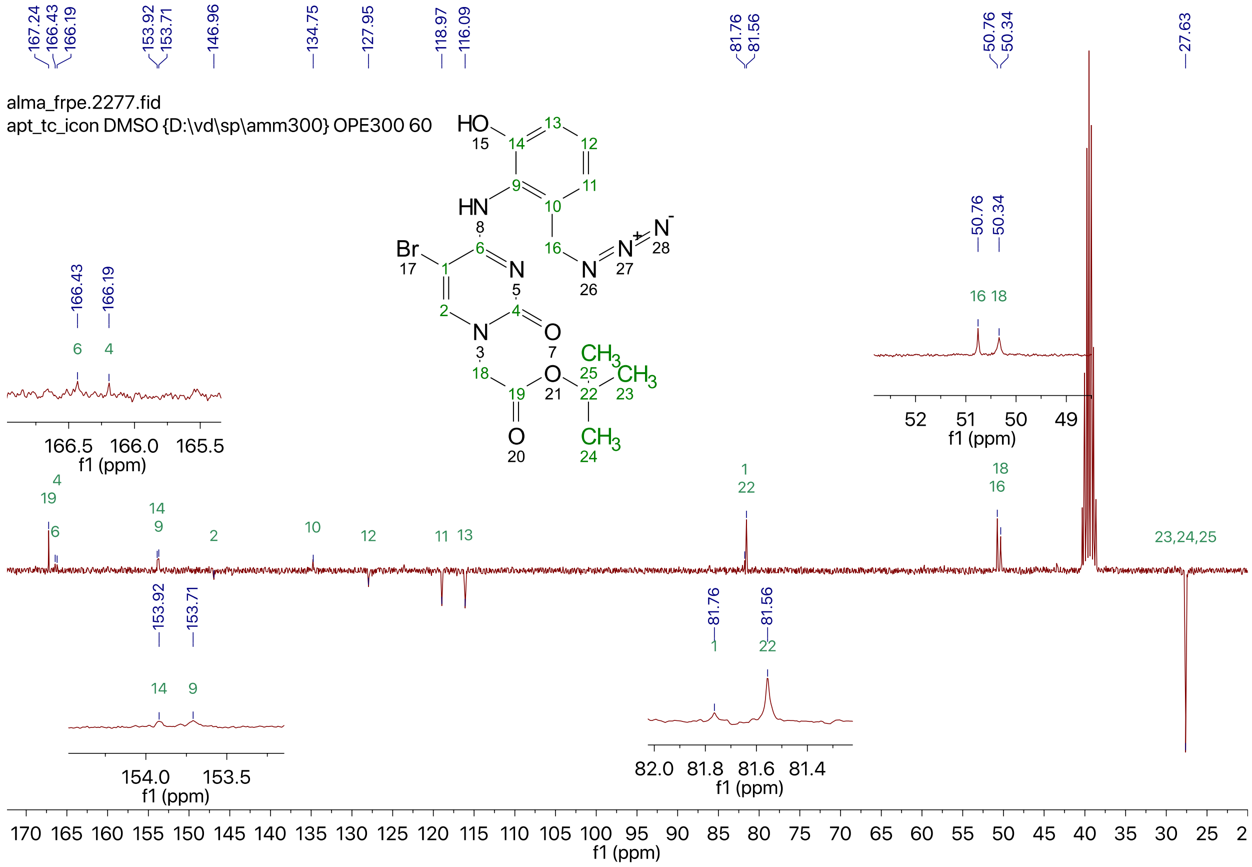

Figure S41: ^{1}H-NMR and ^{13}C-NMR (APT) spectra of **21**.

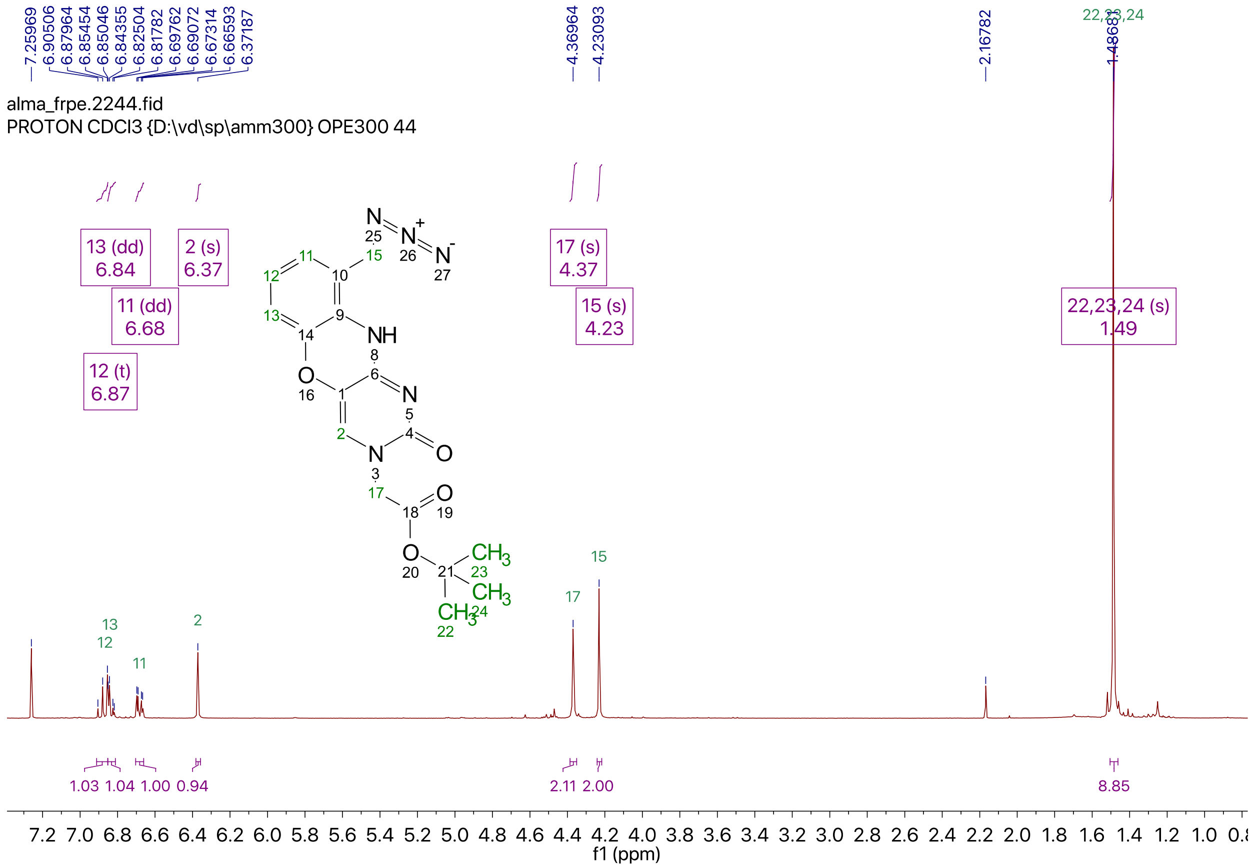

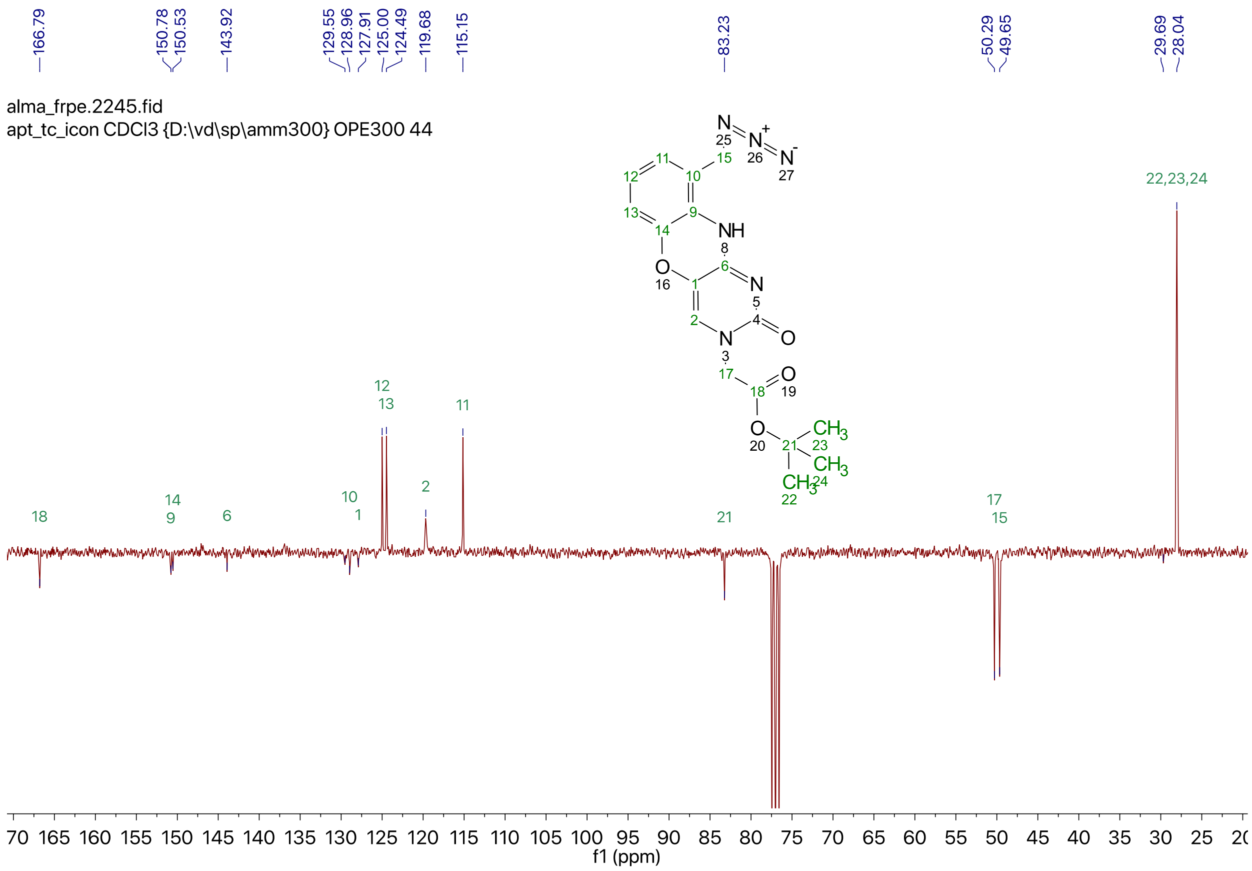

Figure S42: ^{1}H-NMR and ^{13}C-NMR (APT) spectra of **22**.

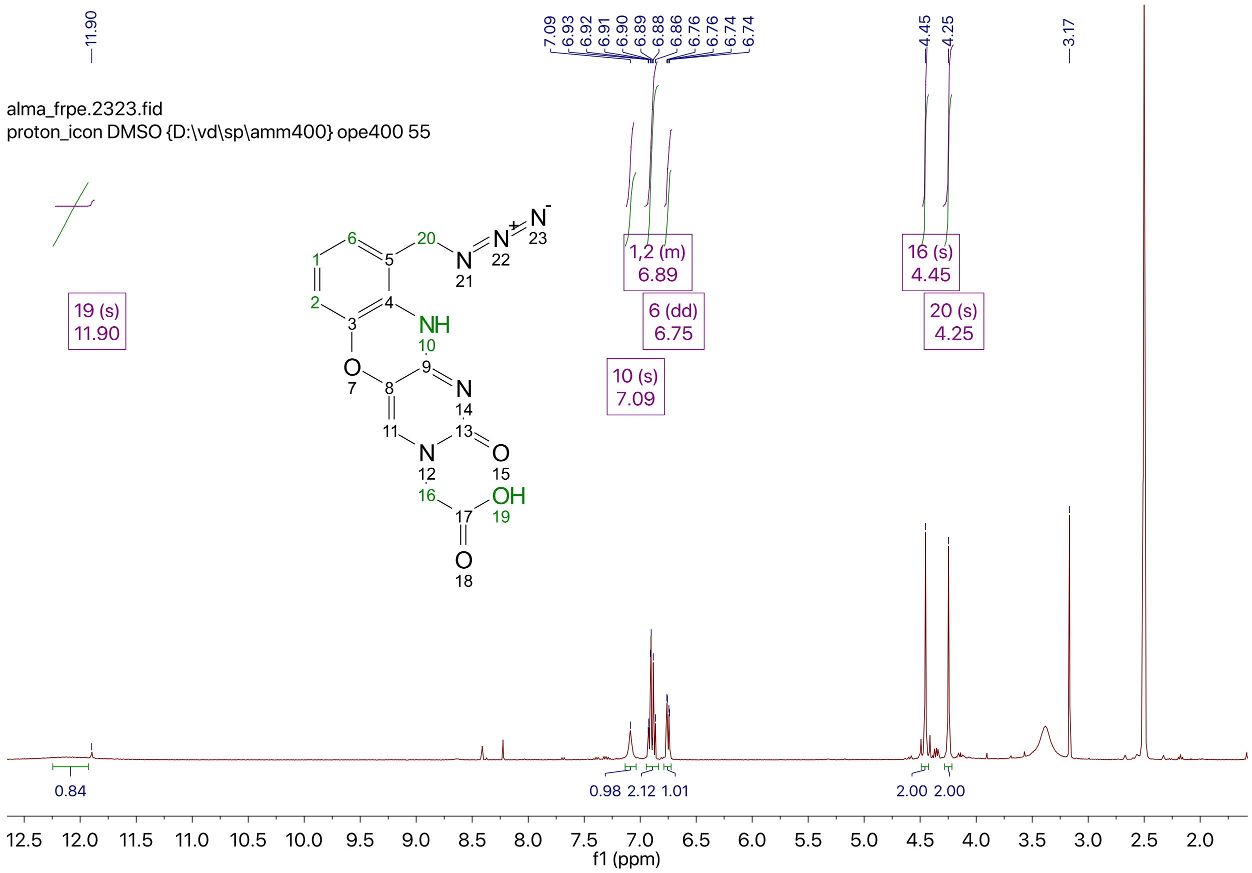

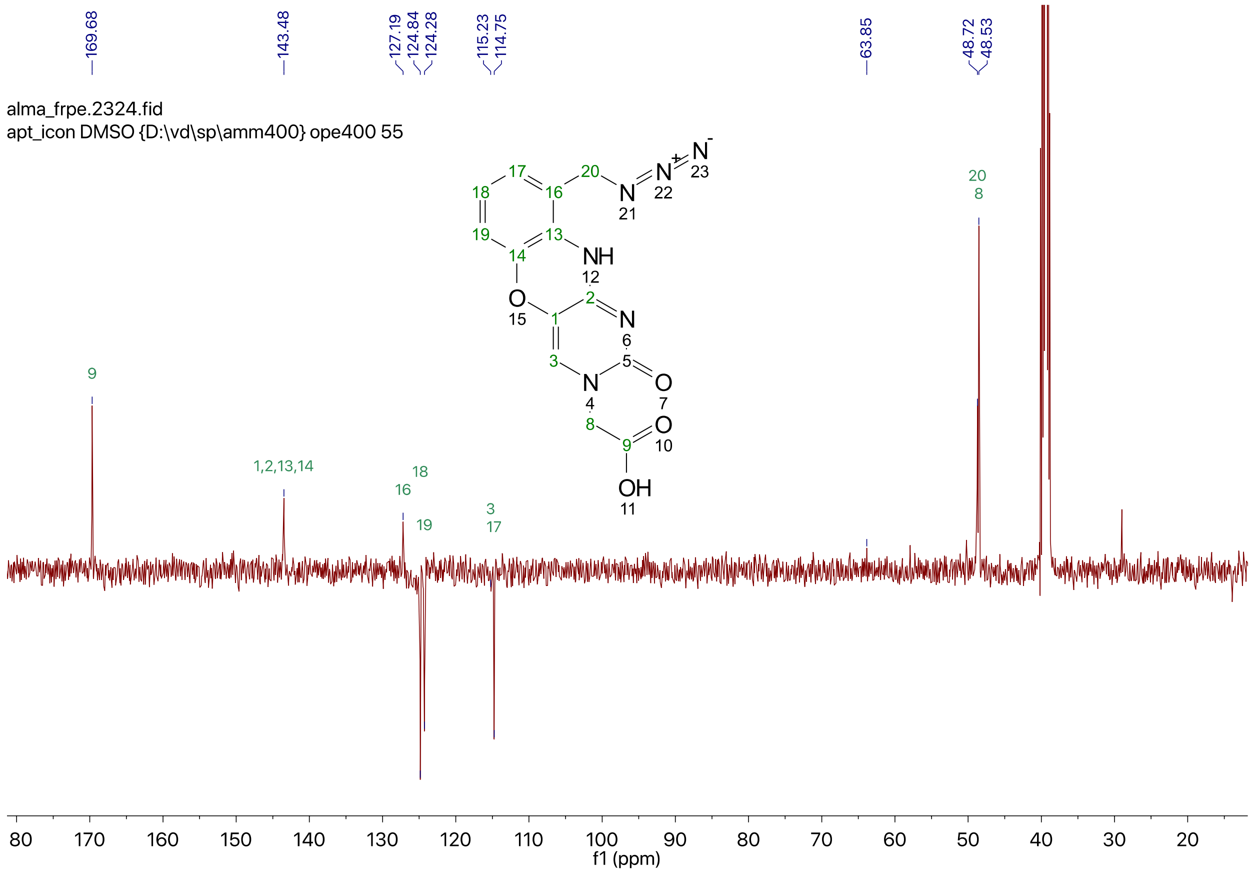

Figure S43: ^{1}H-NMR and ^{13}C-NMR (APT) spectra of **23**.

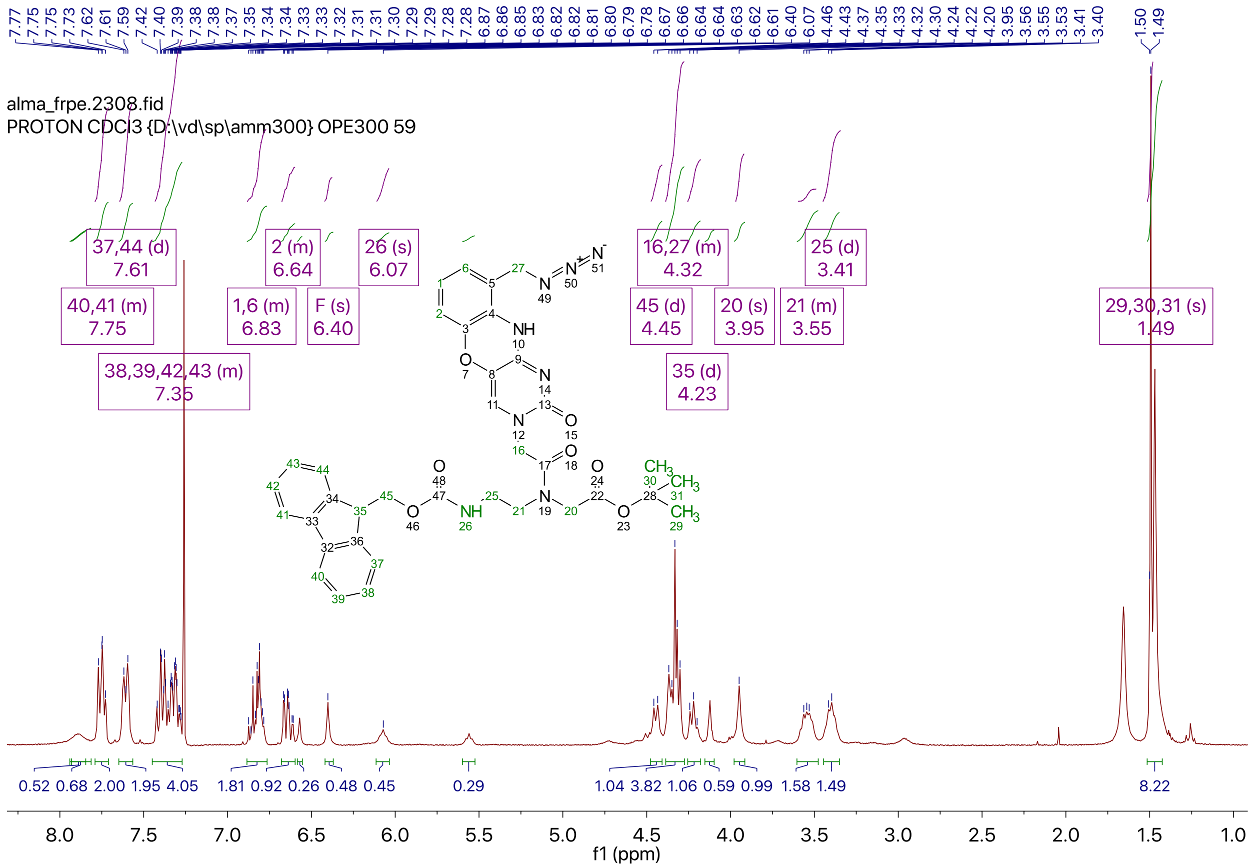

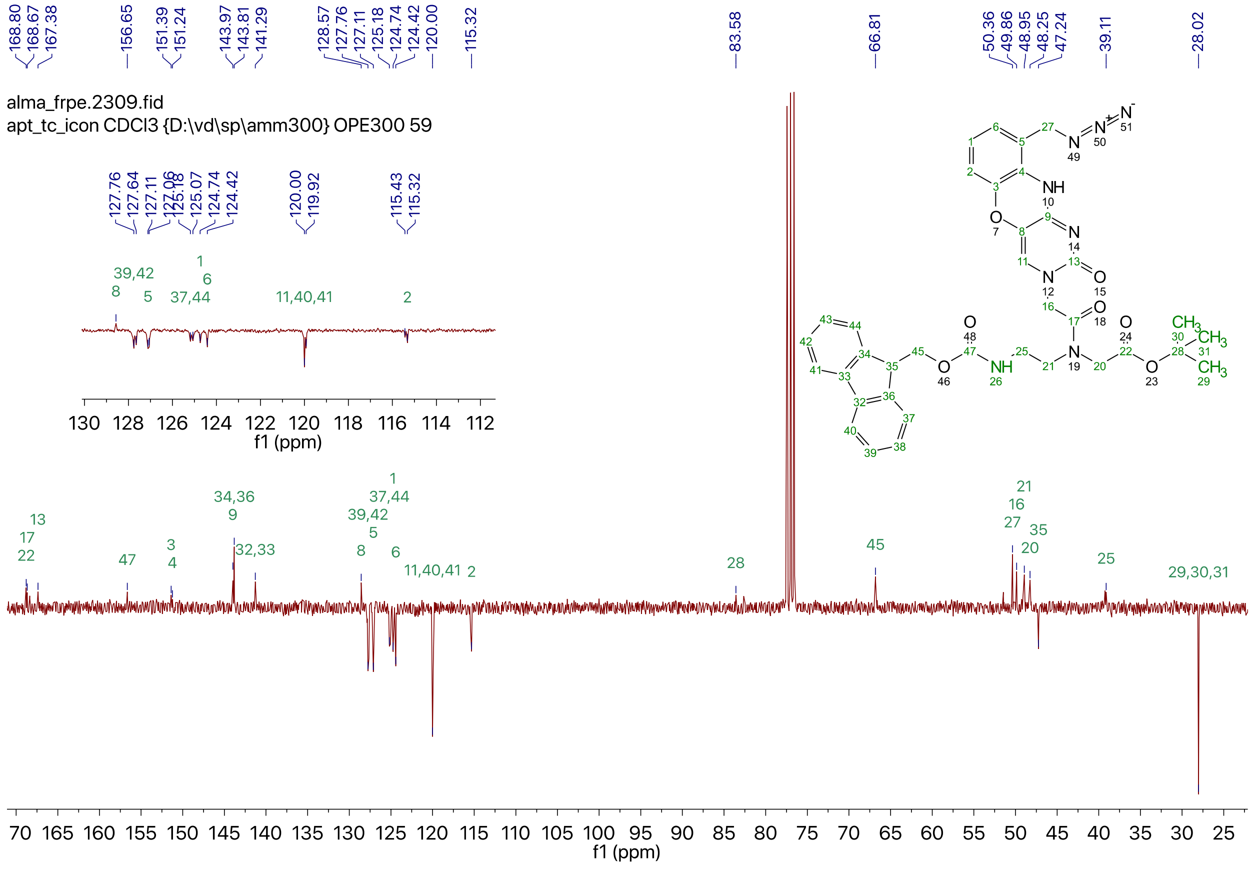

Figure S44: ^{1}H-NMR and ^{13}C-NMR (APT) spectra of **24**.

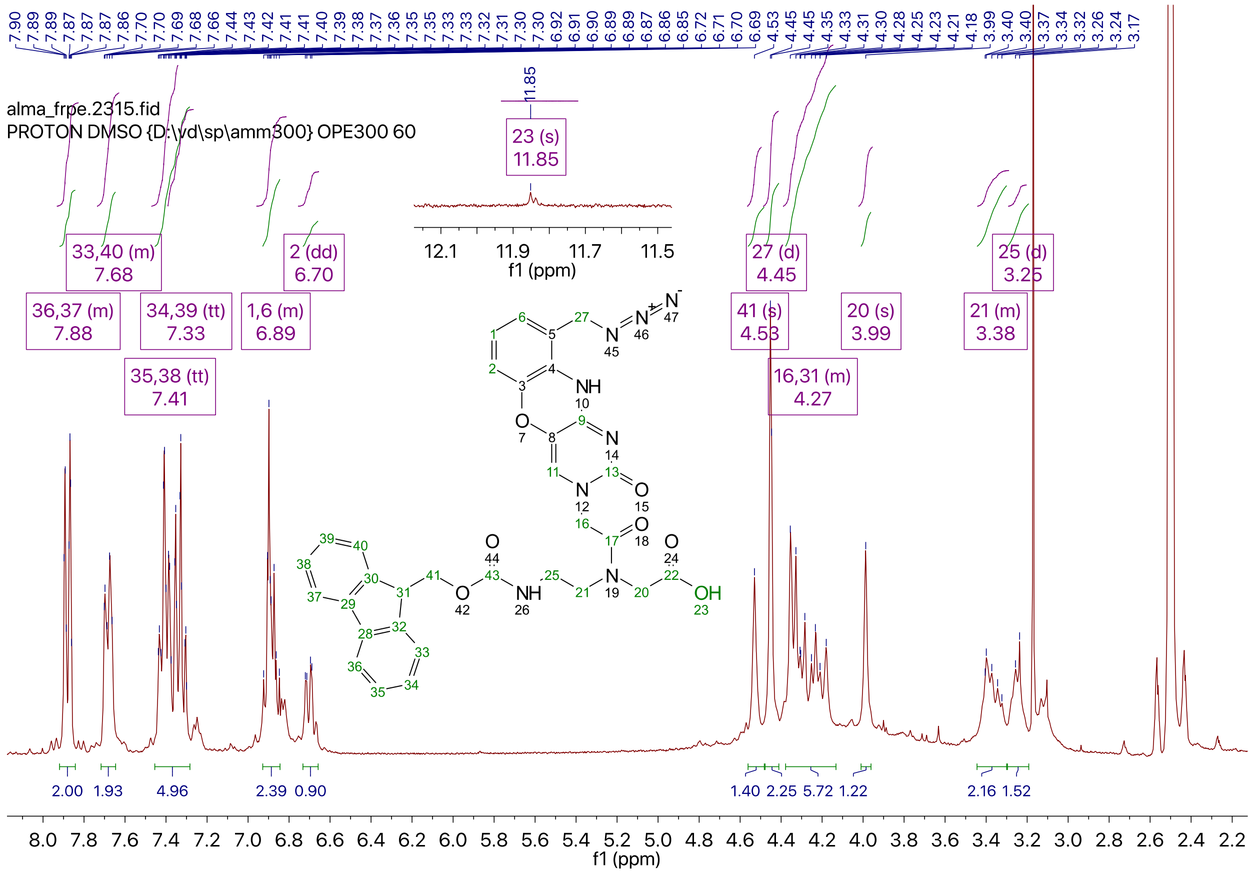

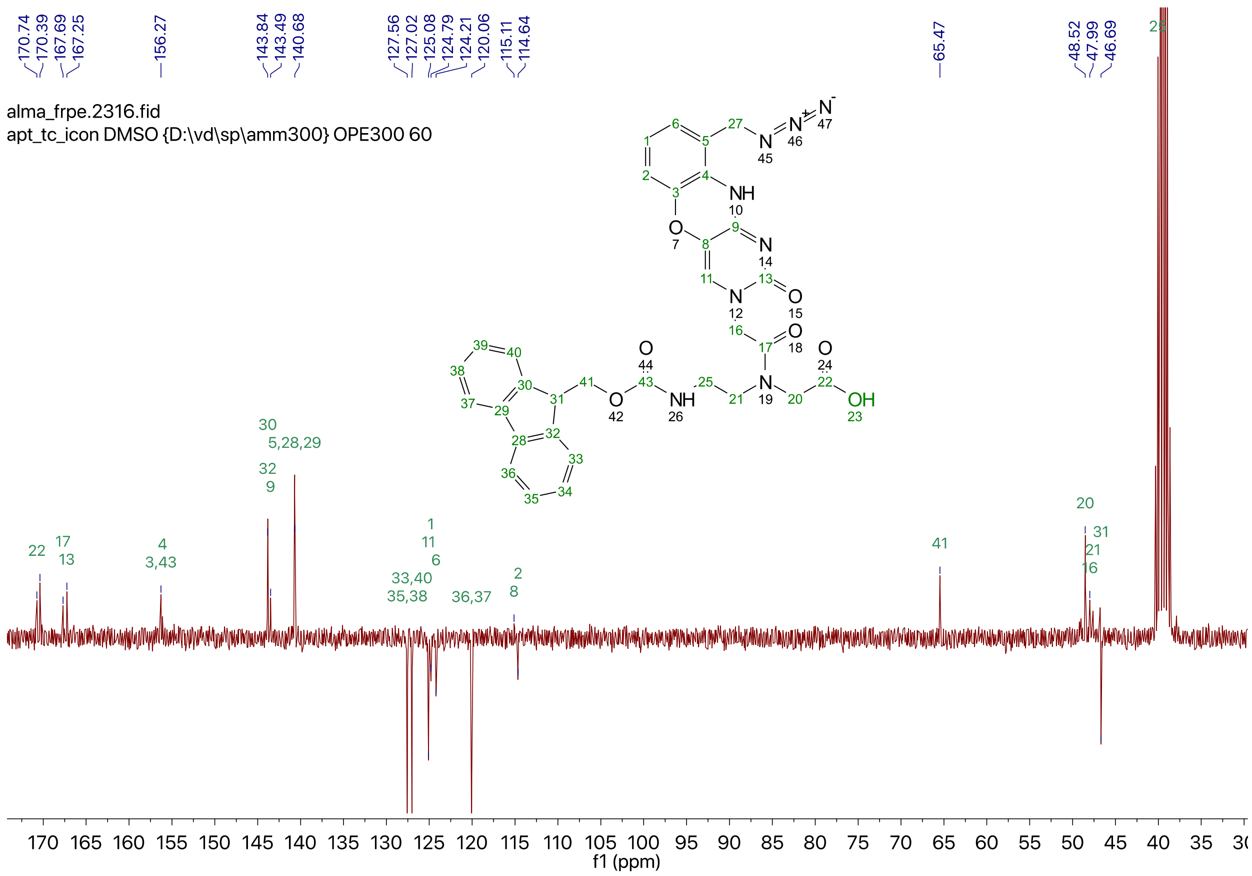

Figure S45: ^{1}H-NMR and ^{13}C-NMR (APT) spectra of **M3**.

10.HPLC-MS chromatograms of pure PNAs

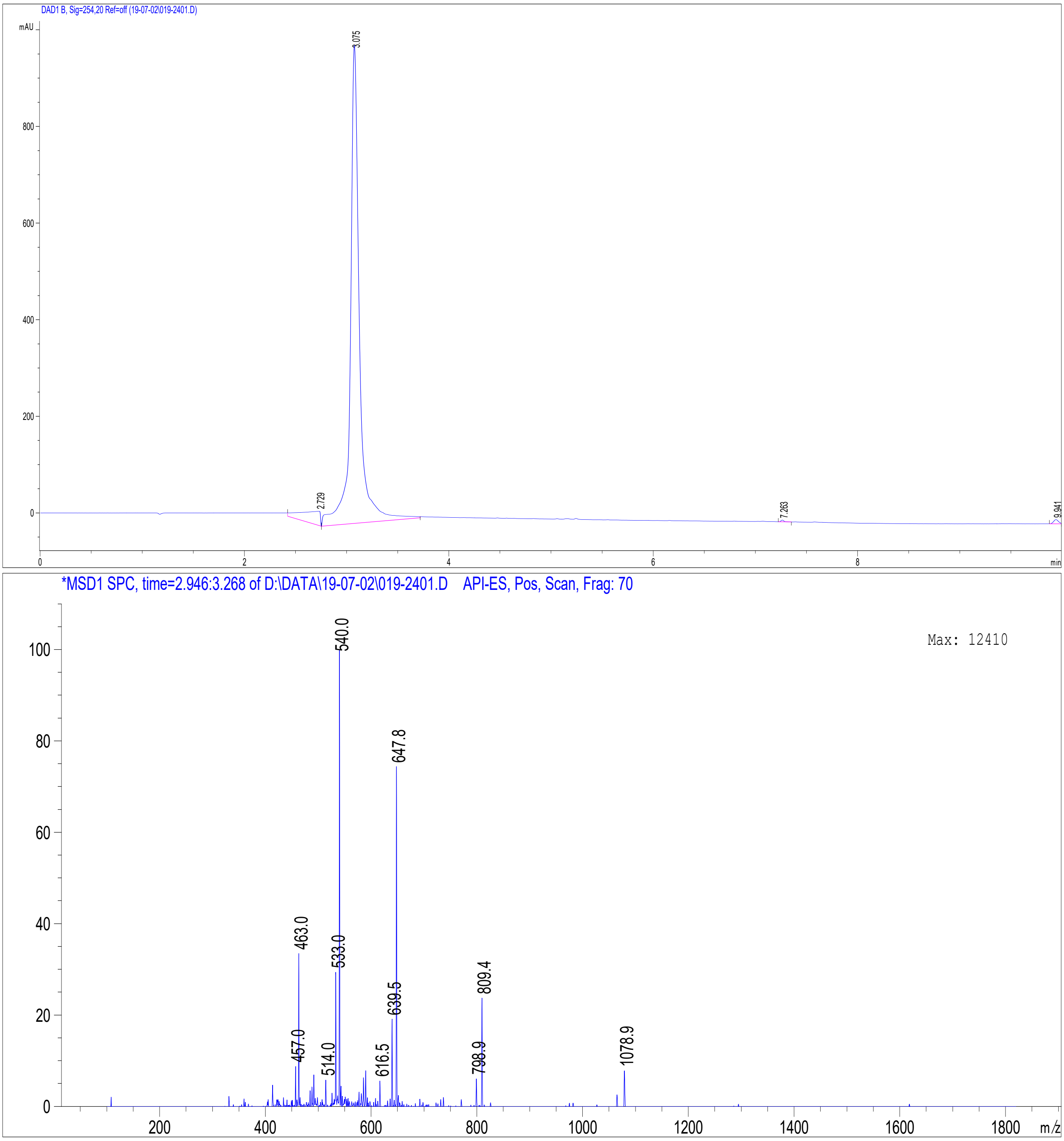

Figure S46: HPLC-MS chromatogram (HPLC1) of purified **PNA-1A**. HPLC-UV trace at 254 nm (top) and MS spectrum of the corresponding peak (bottom). Calcd MW: 3234.3, m/z found: 1078.9 $[M+3H]^{3+}$, 809.4 $[M+4H]^{4+}$, 647.8 $[M+5H]^{5+}$, 540.0 $[M+6H]^{6+}$, 463.0 $[M+7H]^{7+}$.

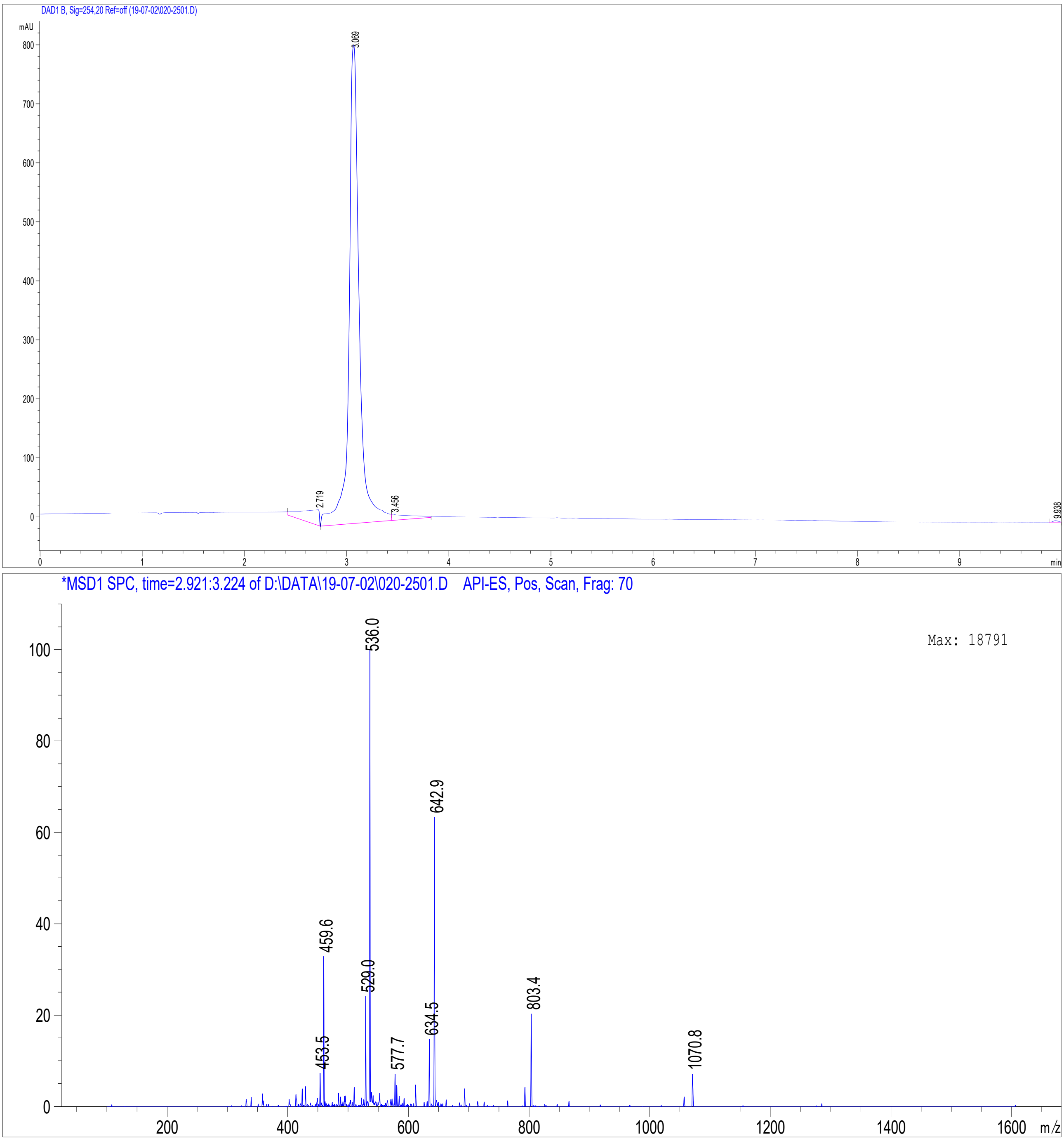

Figure S47: HPLC-MS chromatogram (HPLC1) of purified **PNA-1C**. HPLC-UV trace at 254 nm (top) and MS spectrum of the corresponding peak (bottom). Calcd MW: 3210.2, m/z found: 1070.8 $[M+3H]^{3+}$, 803.4 $[M+4H]^{4+}$, 642.9 $[M+5H]^{5+}$, 536.0 $[M+6H]^{6+}$, 459.6 $[M+7H]^{7+}$.

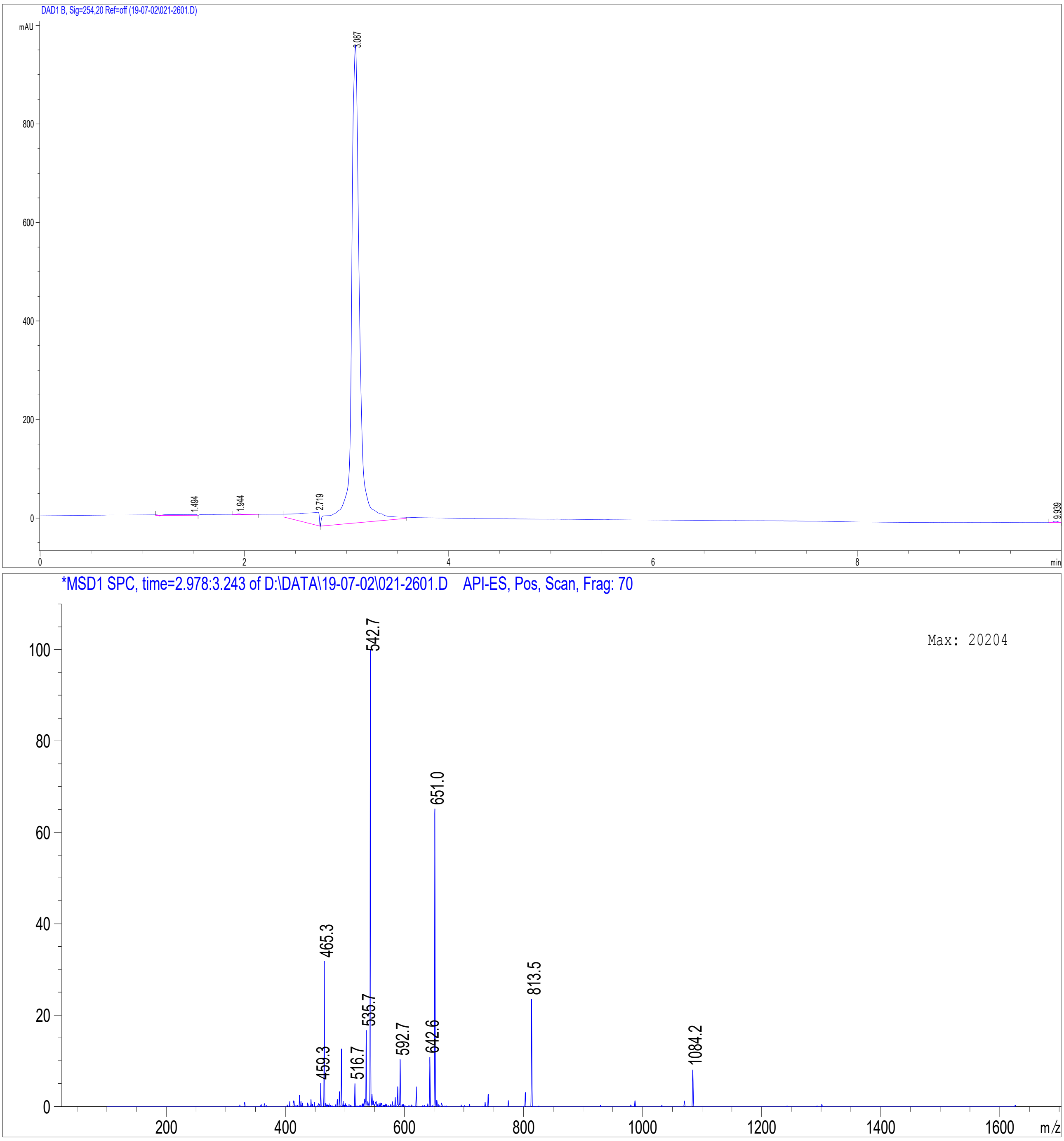

Figure S48: HPLC-MS chromatogram (HPLC1) of purified **PNA-1G**. HPLC-UV trace at 254 nm (top) and MS spectrum of the corresponding peak (bottom). Calcd MW: 3249.9, m/z found: 1084.2 $[M+3H]^{3+}$, 813.5 $[M+4H]^{4+}$, 651.0 $[M+5H]^{5+}$, 542.7 $[M+6H]^{6+}$, 465.3 $[M+7H]^{7+}$.

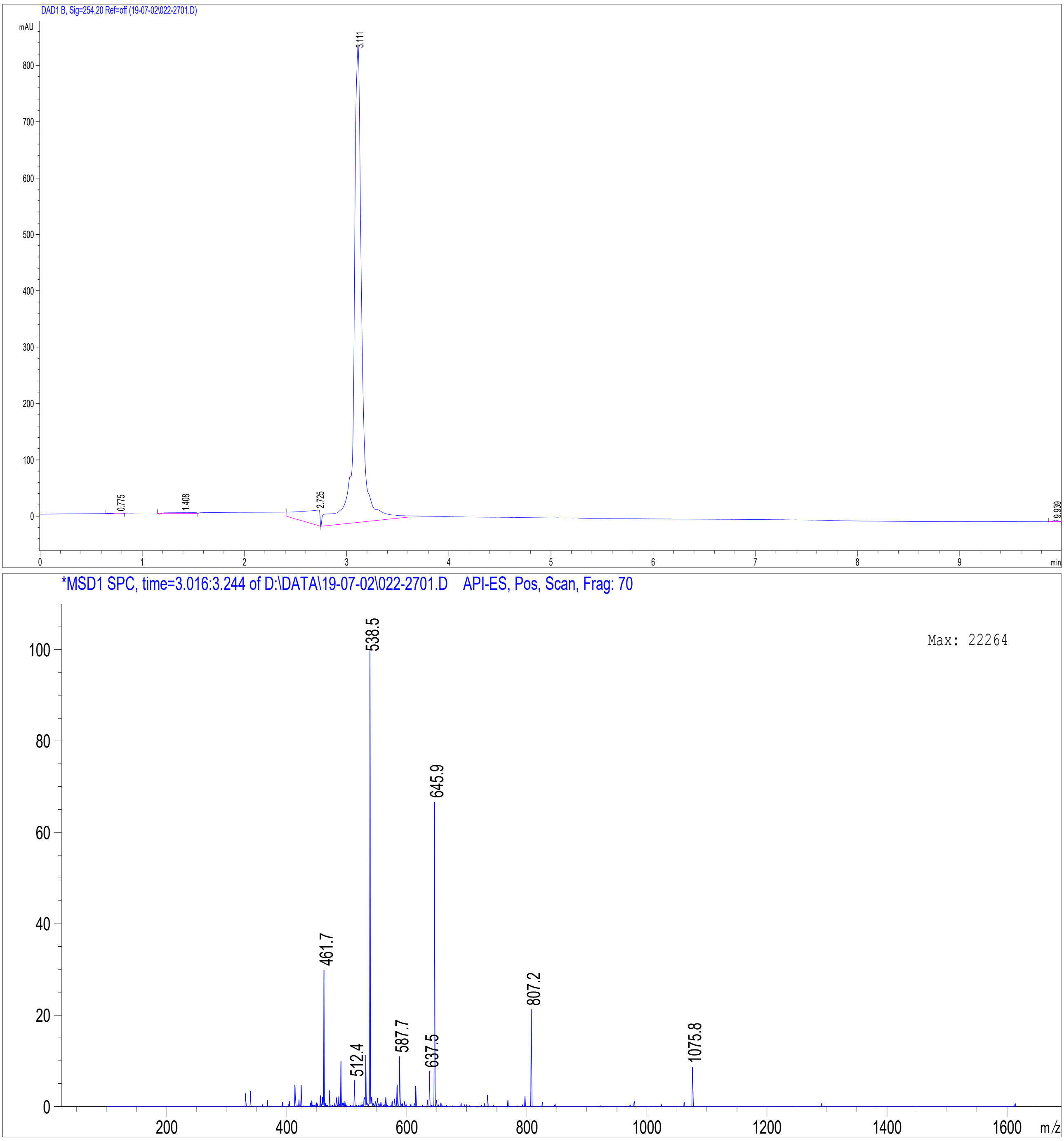

Figure S49: HPLC-MS chromatogram (HPLC1) of purified **PNA-1T**. HPLC-UV trace at 254 nm (top) and MS spectrum of the corresponding peak (bottom). Calcd MW: 3224.9, m/z found: 1075.8 $[M+3H]^{3+}$, 807.2 $[M+4H]^{4+}$, 645.9 $[M+5H]^{5+}$, 538.5 $[M+6H]^{6+}$, 461.7 $[M+7H]^{7+}$.

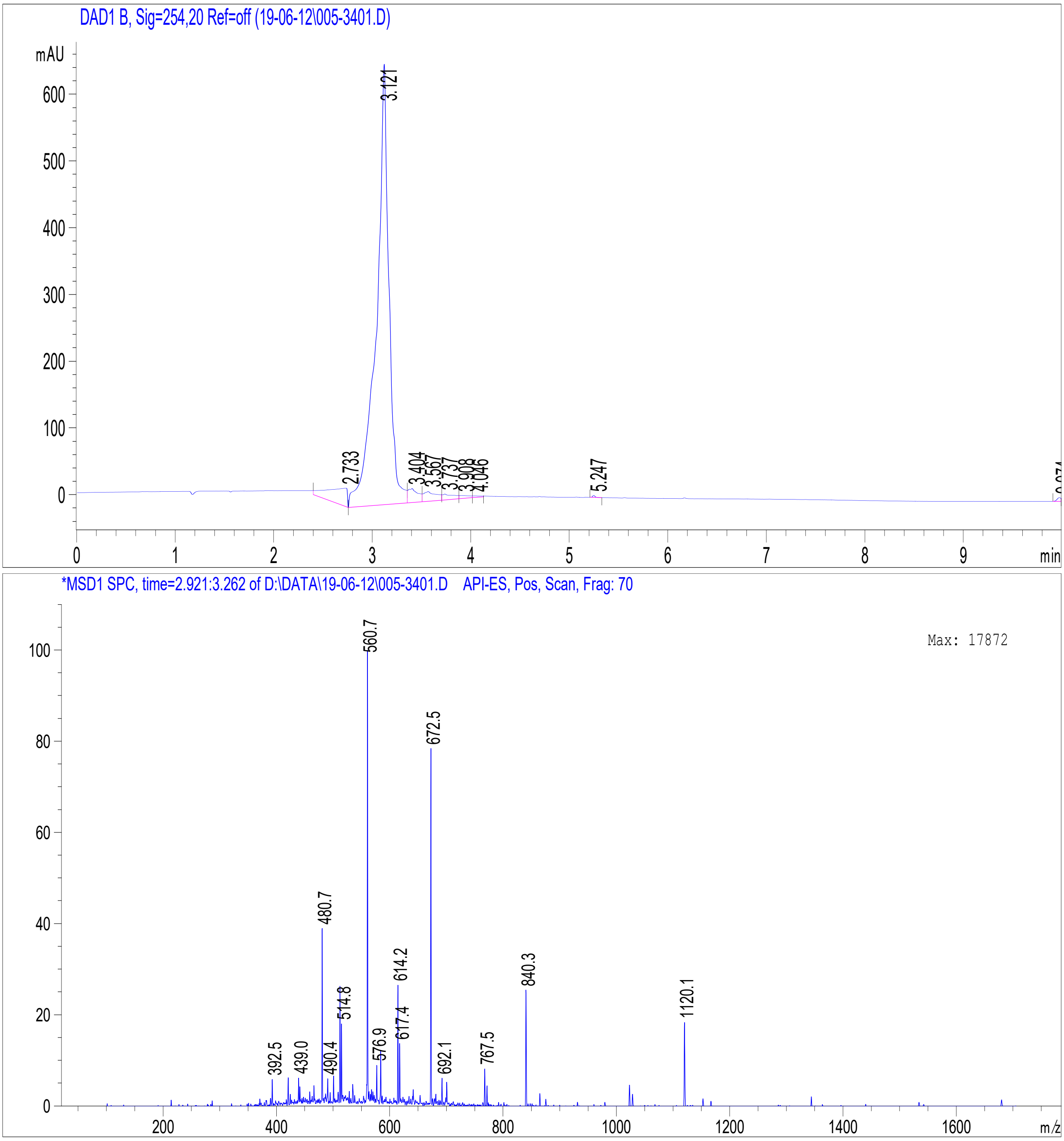

Figure S50: HPLC-MS chromatogram (HPLC1) of purified **PNA-2A**. HPLC-UV trace at 254 nm (top) and MS spectrum of the corresponding peak (bottom). Calcd MW: 3358.4, m/z found: 1120.1 $[M+3H]^{3+}$, 840.3 $[M+4H]^{4+}$, 672.5 $[M+5H]^{5+}$, 560.7 $[M+6H]^{6+}$, 480.7 $[M+7H]^{7+}$.

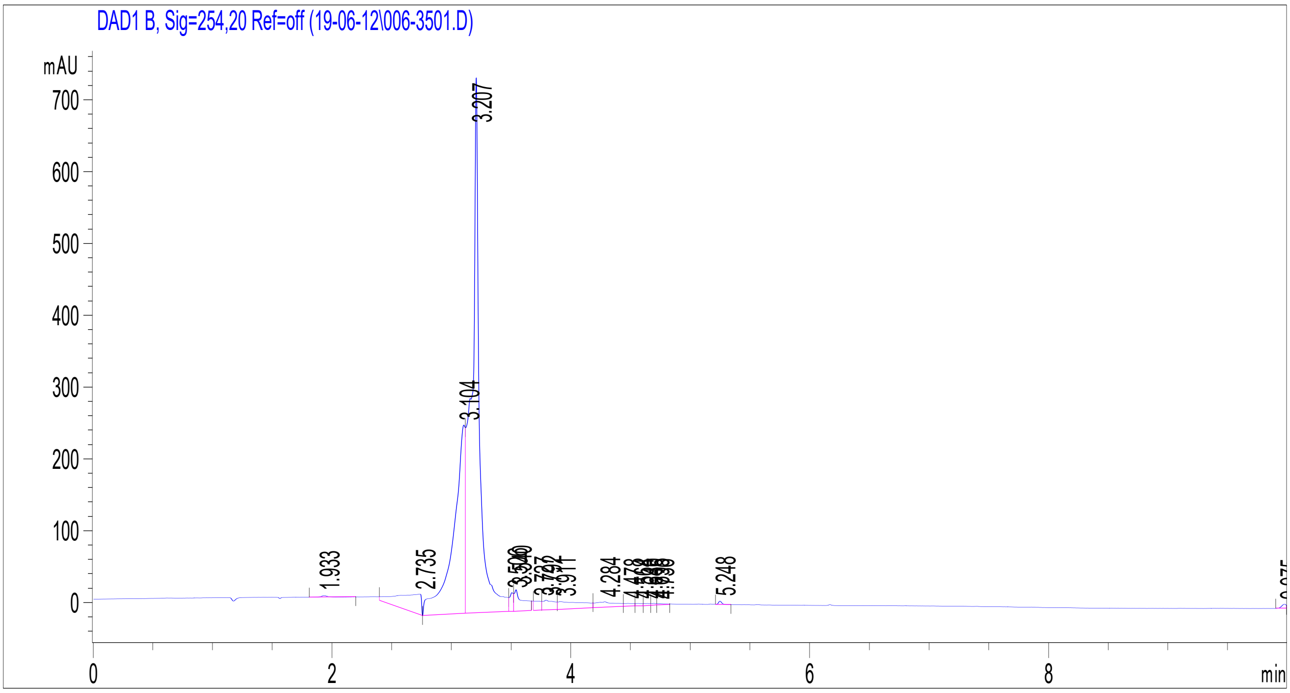

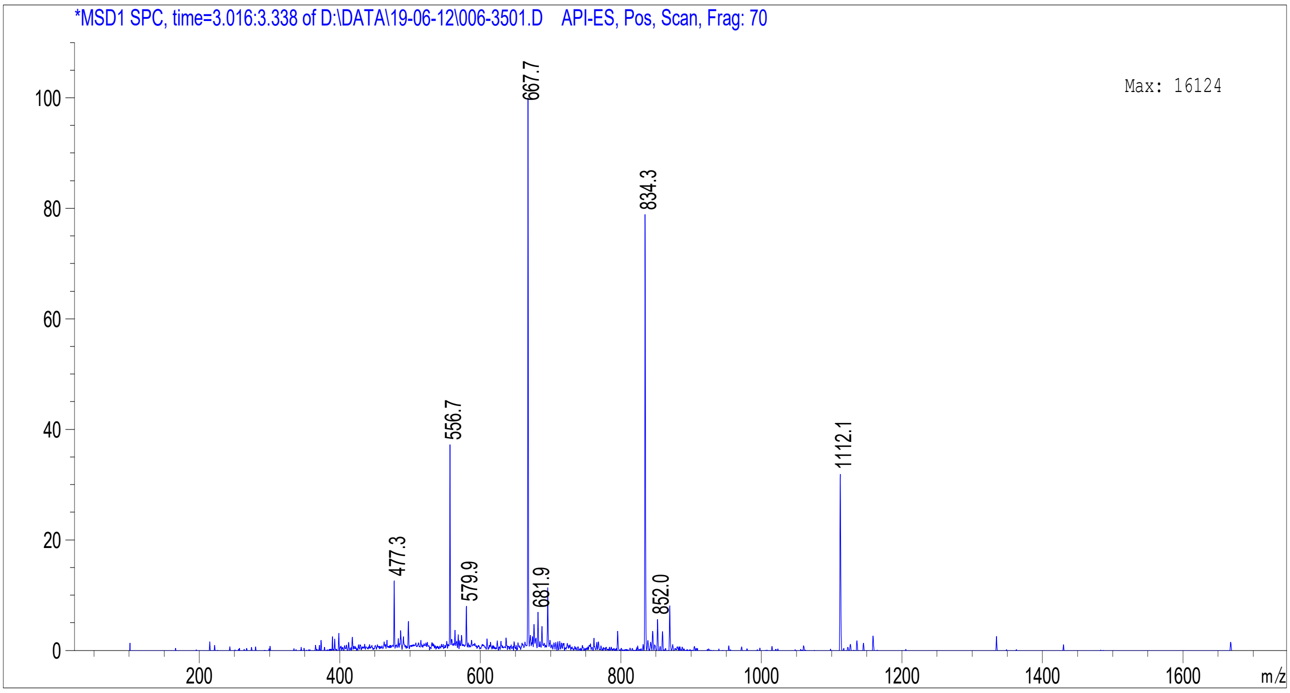

Figure S51: HPLC-MS chromatogram (HPLC1) of purified **PNA-2C**. HPLC-UV trace at 254 nm (top) and MS spectrum of the corresponding peak (bottom). Calcd MW: 3374.4, m/z found: 1125.4 $[M+3H]^{3+}$, 844.3 $[M+4H]^{4}$+, 675.6 $[M+5H]^{5+}$, 563.3 $[M+6H]^{6+}$, 483.0 $[M+7H]^{7+}$.

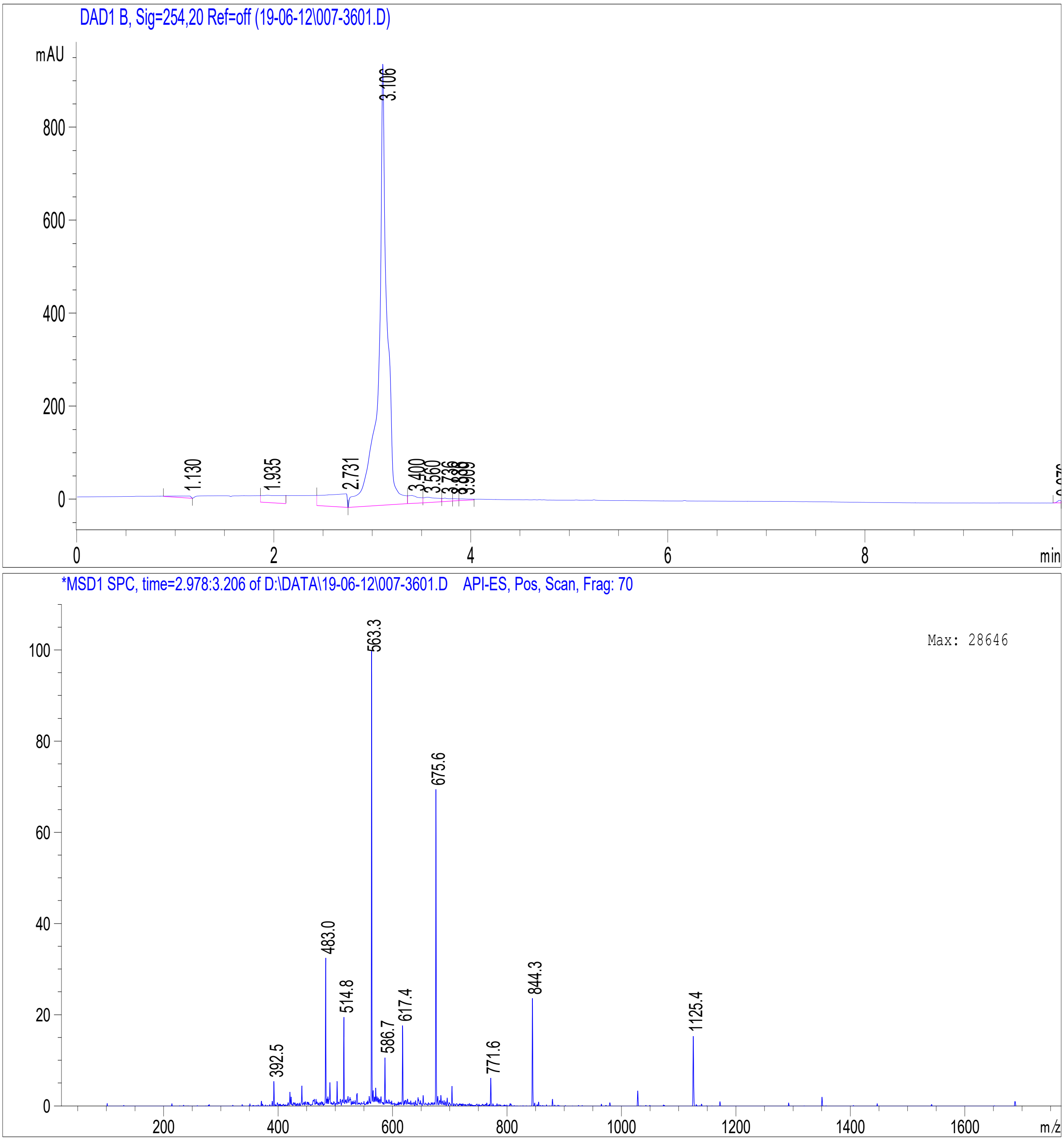

Figure S52: HPLC-MS chromatogram (HPLC1) of purified **PNA-2G**. HPLC-UV trace at 254 nm (top) and MS spectrum of the corresponding peak (bottom). Calcd MW: 3374.4, m/z found: 1125.4 $[M+3H]^{3+}$, 844.3 $[M+4H]^{4+}$, 675.6 $[M+5H]^{5+}$, 563.3 $[M+6H]^{6+}$, 483.0 $[M+7H]^{7+}$.

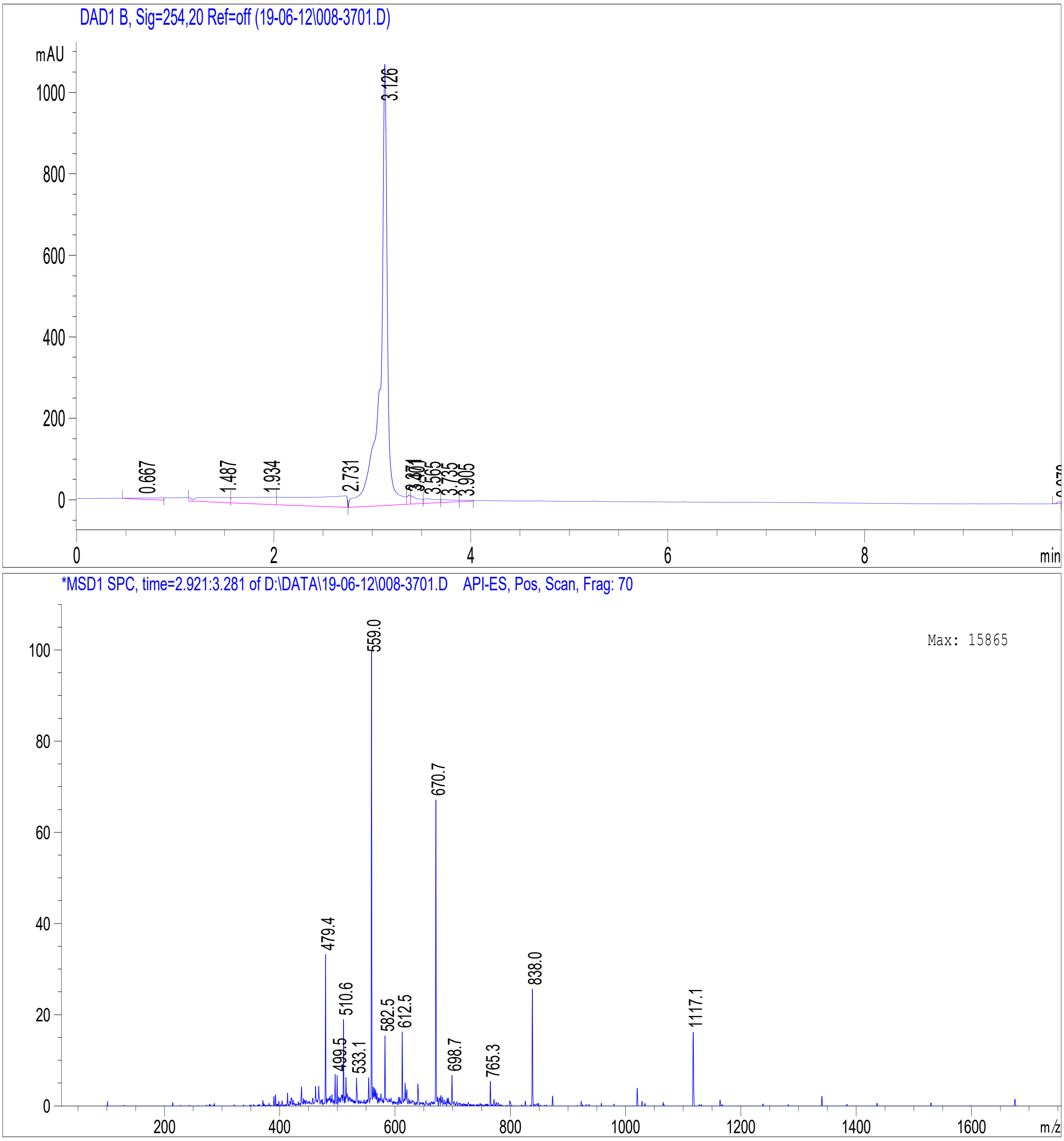

Figure S53: HPLC-MS chromatogram (HPLC1) of purified **PNA-2T**. HPLC-UV trace at 254 nm (top) and MS spectrum of the corresponding peak (bottom). Calcd MW: 3349.3, m/z found: 1117.1 $[M+3H]^{3+}$, 838.0 $[M+4H]^{4+}$, 670.7; $[M+5H]^{5+}$, 559.0 $[M+6H]^{6+}$, 479.4 $[M+7H]^{7+}$.

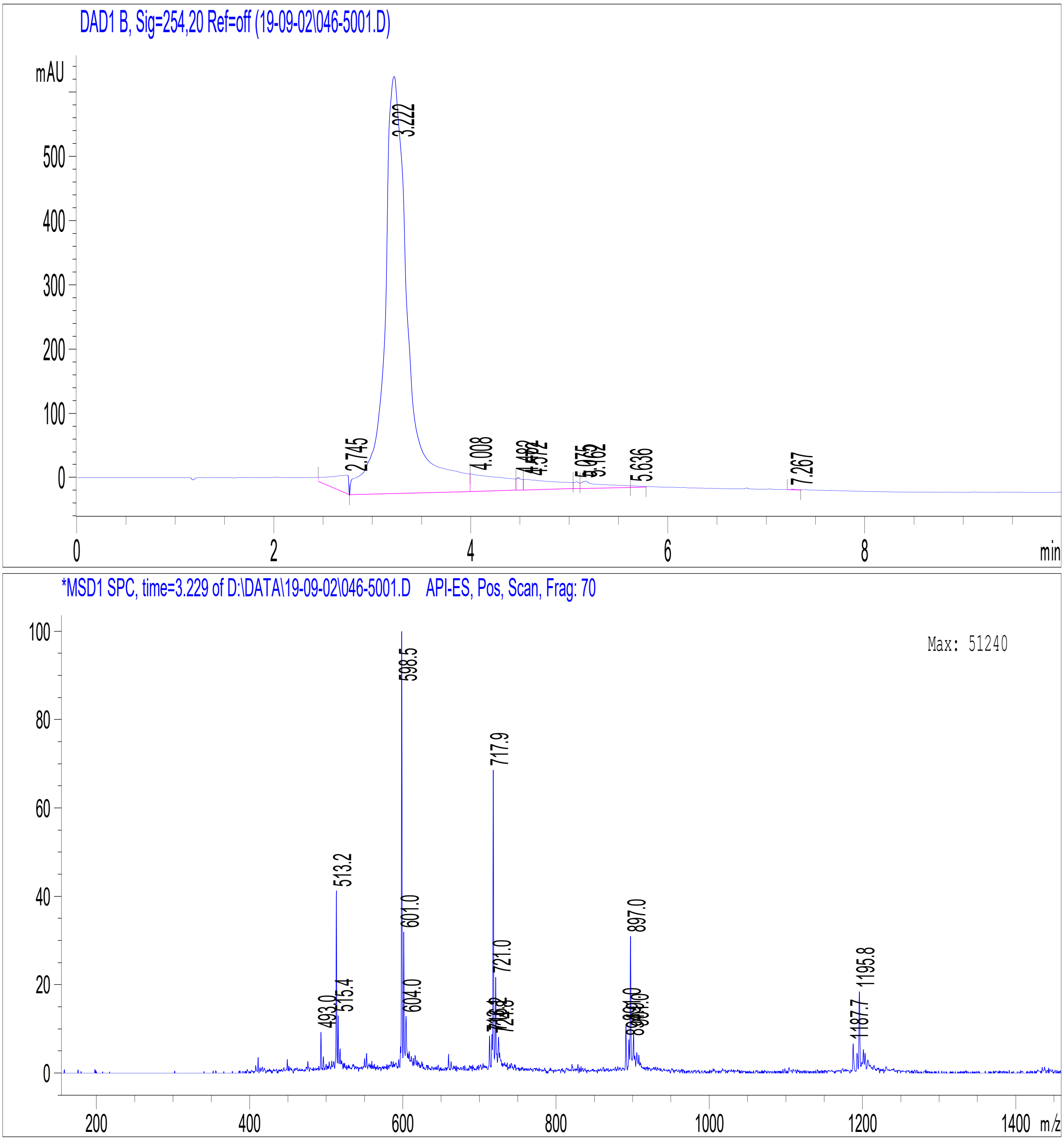

Figure S54: HPLC-MS chromatogram (HPLC1) of purified **PNA-3A**. HPLC-UV trace at 254 nm (top) and MS spectrum of the corresponding peak (bottom). Calcd MW: 3585.6, m/z found: 1195.8 $[M+3H]^{3+}$, 897.0 $[M+4H]^{4}$+, 717.9 $[M+5H]^{5+}$, 598.5 $[M+6H]^{6+}$, 513.2 $[M+7H]^{7+}$.

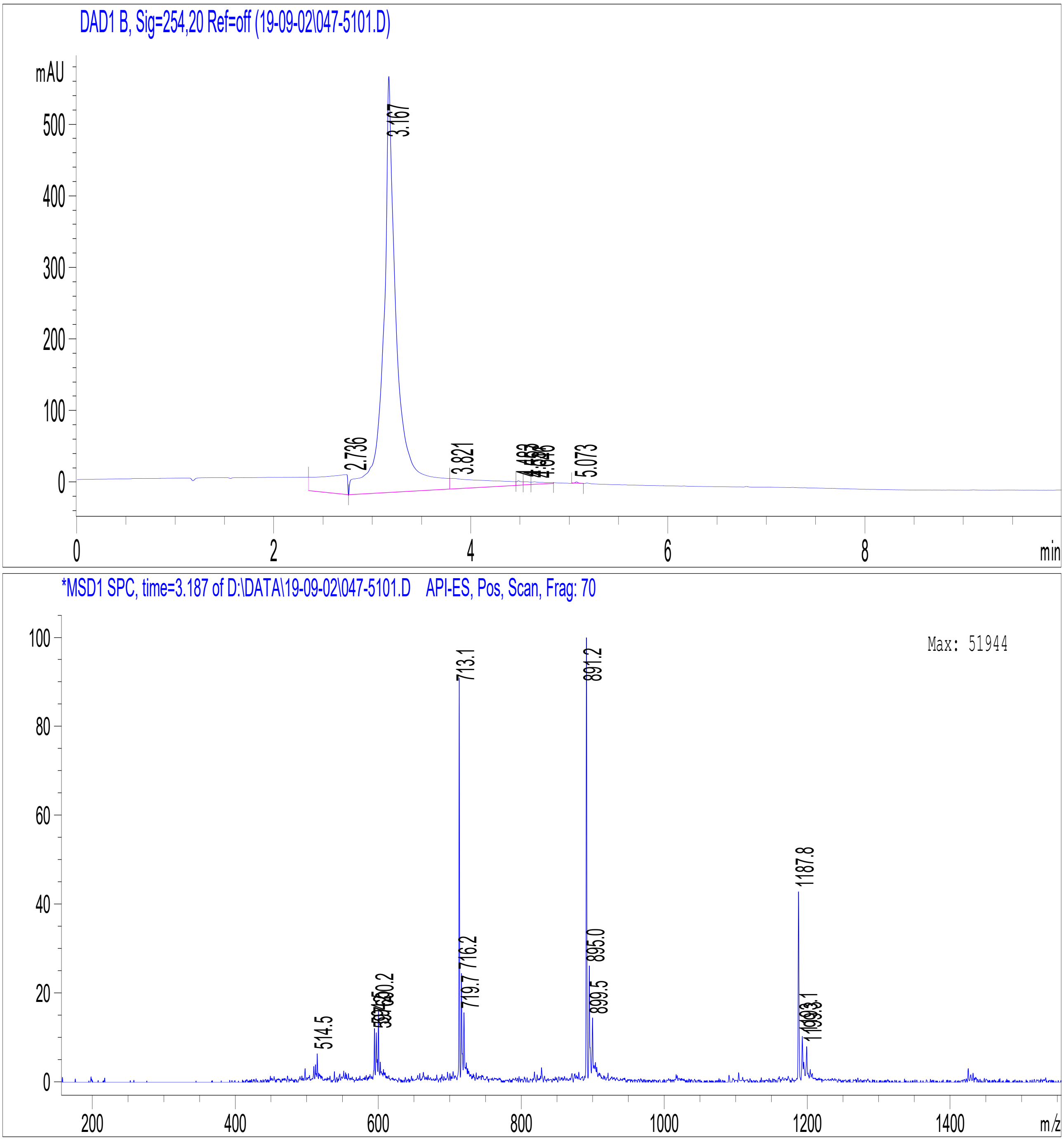

Figure S55: HPLC-MS chromatogram (HPLC1) of purified **PNA-3C**. HPLC-UV trace at 254 nm (top) and MS spectrum of the corresponding peak (bottom). Calcd MW: 3561.6, m/z found: 1187.8 $[M+3H]^{3+}$, 891.2 $[M+4H]^{4+}$, 713.1 $[M+5H]^{5+}$, 594.5 $[M+6H]^{6+}$.

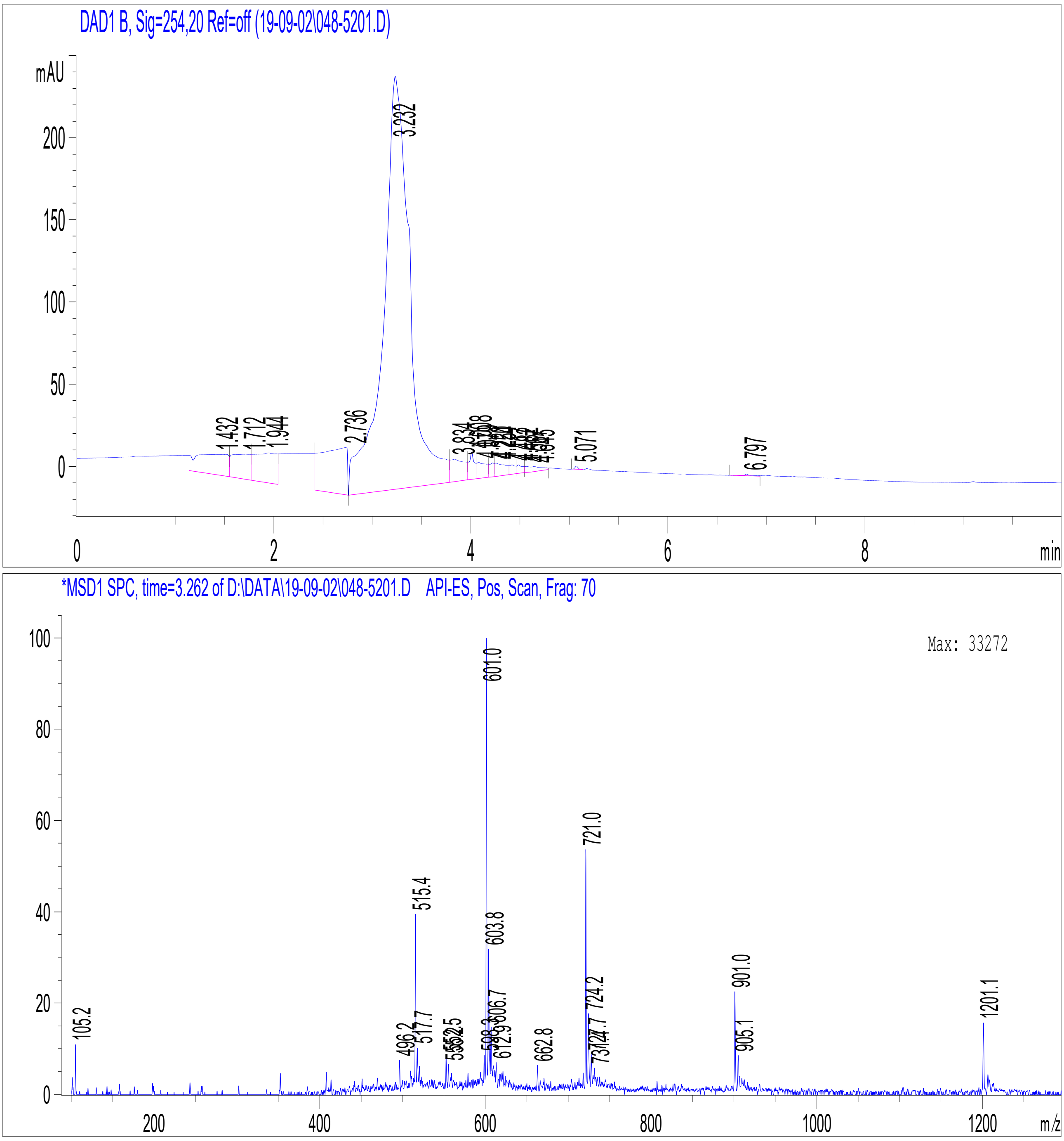

Figure S56: HPLC-MS chromatogram (HPLC1) of purified **PNA-3G**. HPLC-UV trace at 254 nm (top) and MS spectrum of the corresponding peak (bottom). Calcd MW: 3601.6, m/z found: 1201.1 $[M+3H]^{3+}$, 901.0 $[M+4H]^{4+}$, 721.0 $[M+5H]^{5+}$, 601.0 $[M+6H]^{6+}$, 515.5 $[M+7H]^{7+}$.

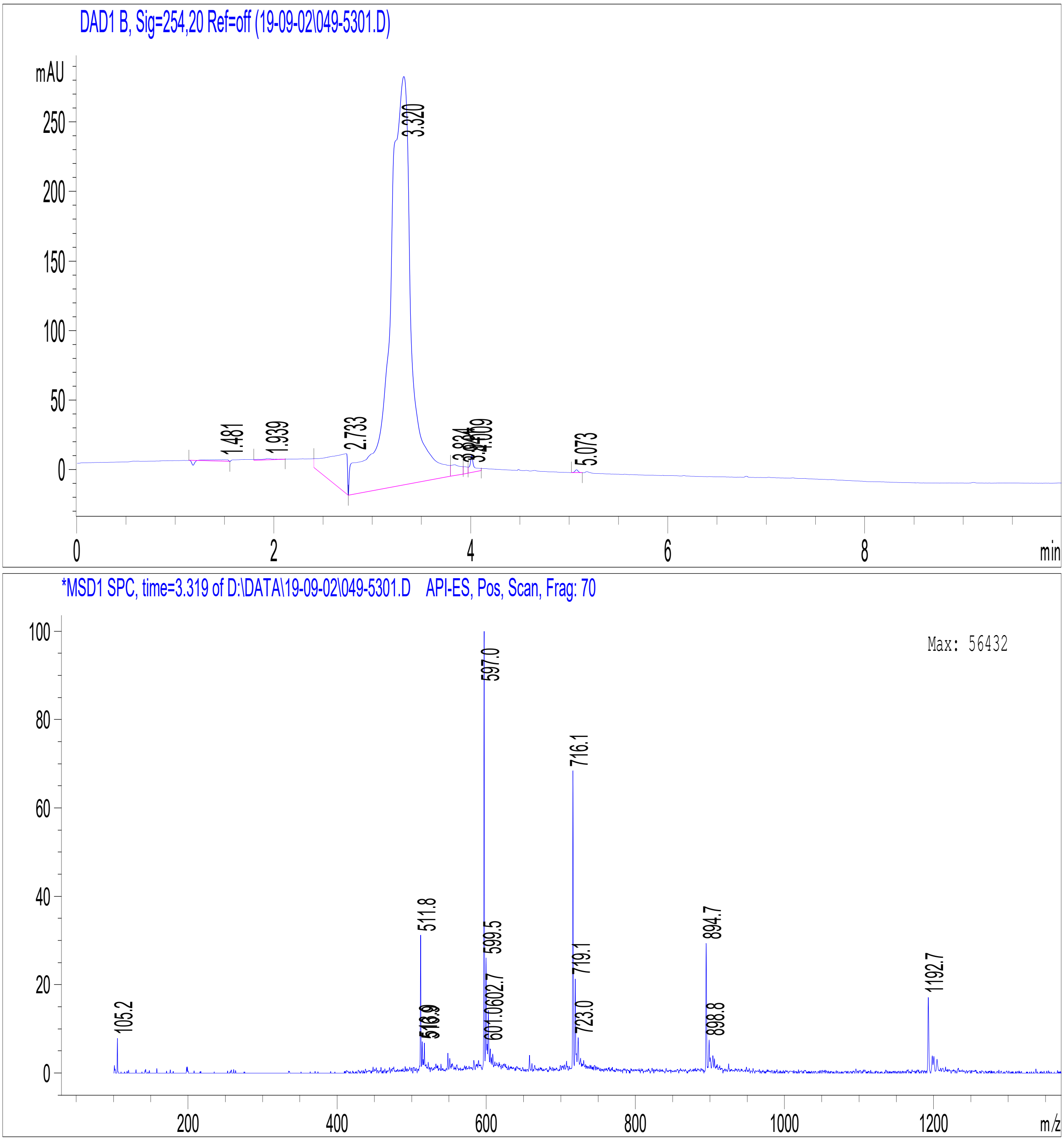

Figure S57: HPLC-MS chromatogram (HPLC1) of purified **PNA-3T**. HPLC-UV trace at 254 nm (top) and MS spectrum of the corresponding peak (bottom). Calcd MW: 3576.5, m/z found: 1192.7 $[M+3H]^{3+}$, 894.7 $[M+4H]^{4+}$, 716.1 $[M+5H]^{5+}$, 597.0 $[M+6H]^{6+}$, 511.8 $[M+7H]^{7+}$.

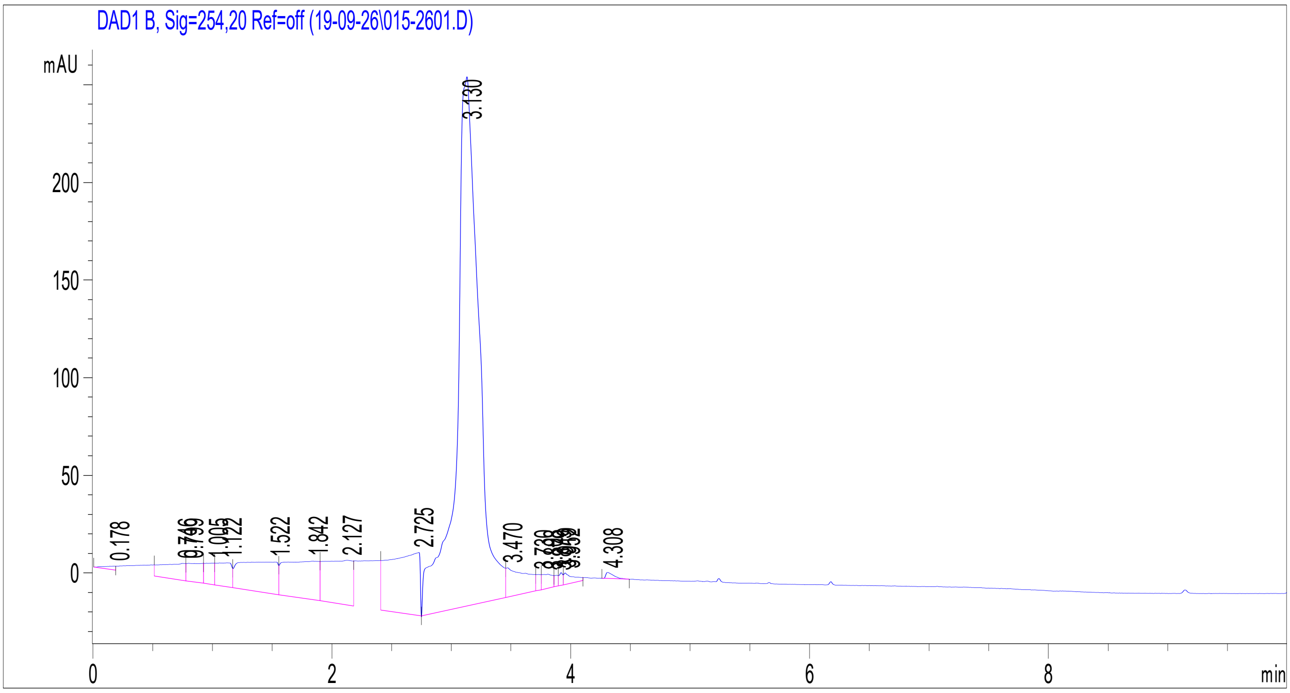

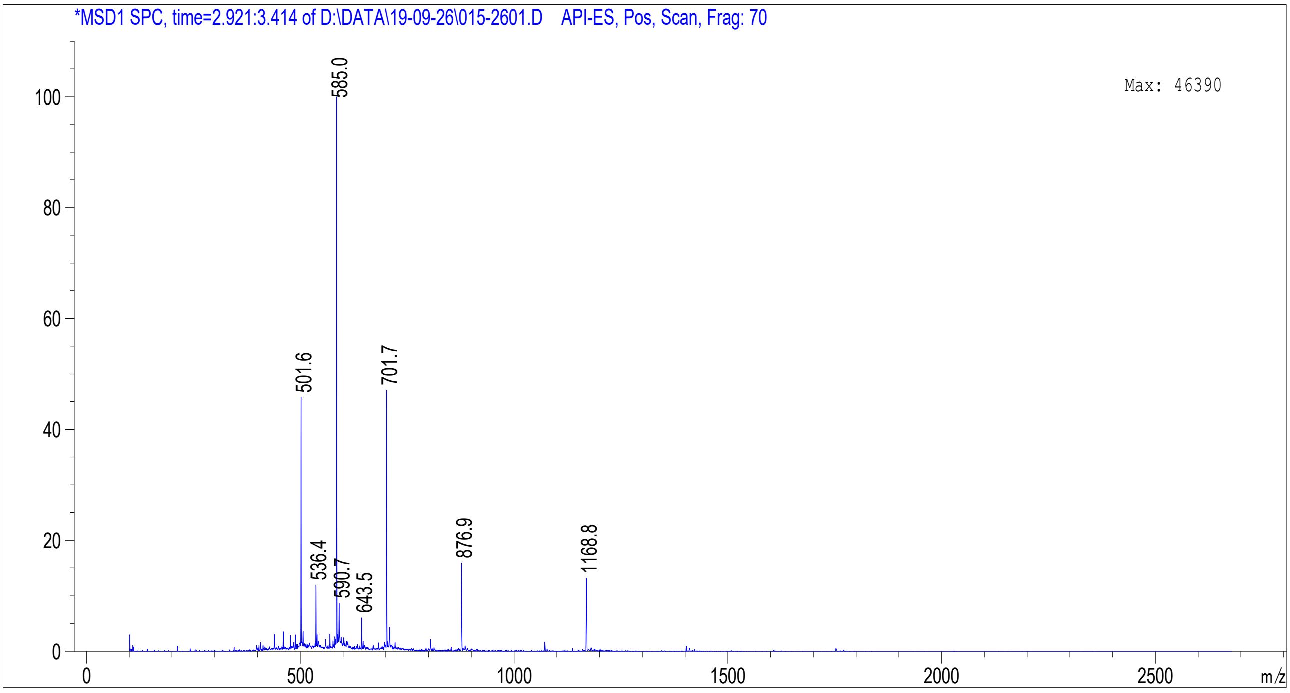

Figure S58: HPLC-MS chromatogram (HPLC1) of purified **PNA-4A**. HPLC-UV trace at 254 nm (top) and MS spectrum of the corresponding peak (bottom). Calcd MW: 3502.5, m/z found: 1168.8 $[M+3H]^{3+}$, 876.9 $[M+4H]^{4+}$, 701.7 $[M+5H]^{5+}$, 585.0 $[M+6H]^{6+}$, 501.6 $[M+7H]^{7+}$.

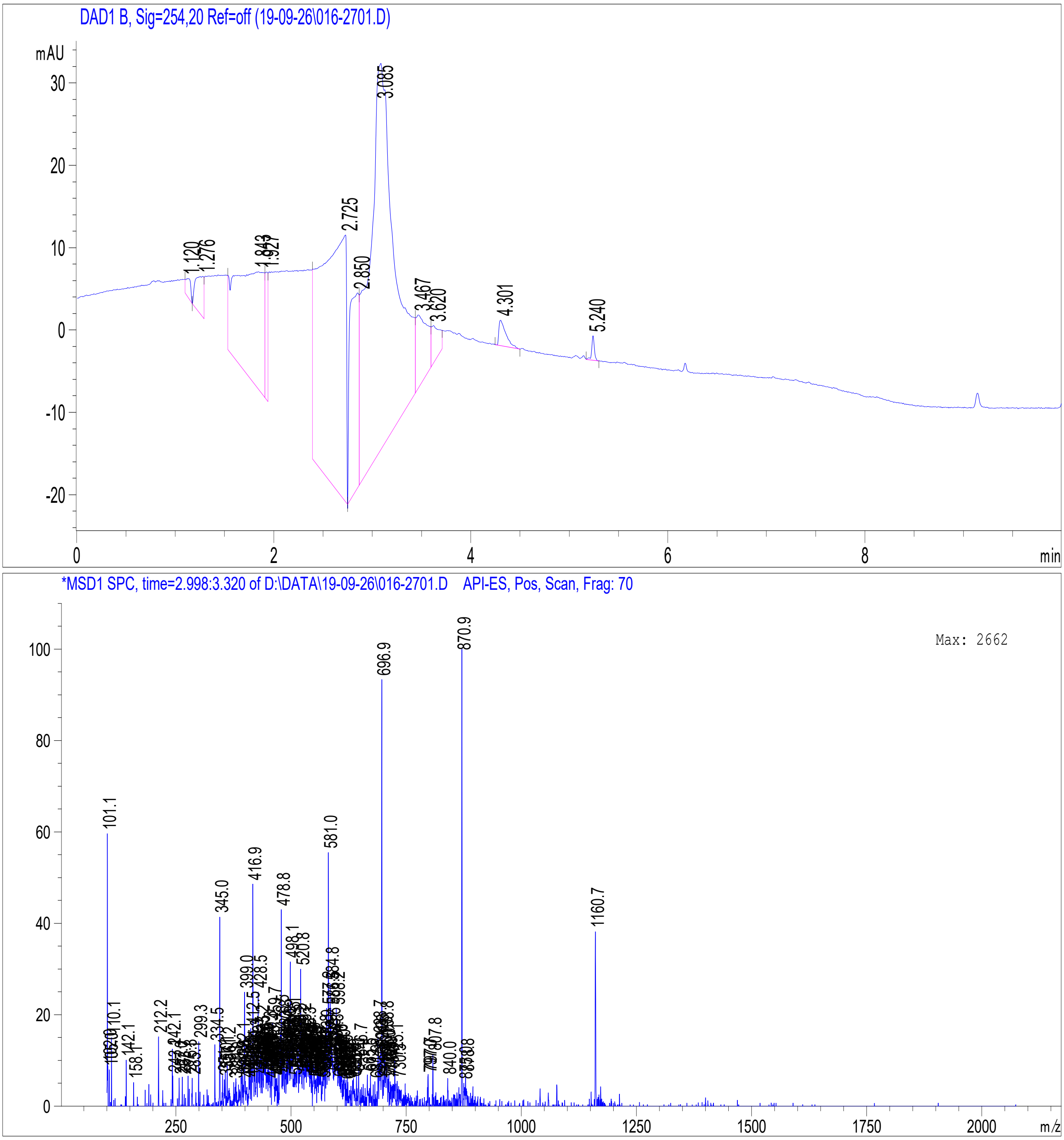

Figure S59: HPLC-MS chromatogram (HPLC1) of purified **PNA-4C**. HPLC-UV trace at 254 nm (top) and MS spectrum of the corresponding peak (bottom). Calcd MW: 3478.5, m/z found: 1160.7 $[M+3H]^{3+}$, 870.9 $[M+4H]^{4+}$, 696.9 $[M+5H]^{5+}$, 581.0 $[M+6H]^{6+}$.

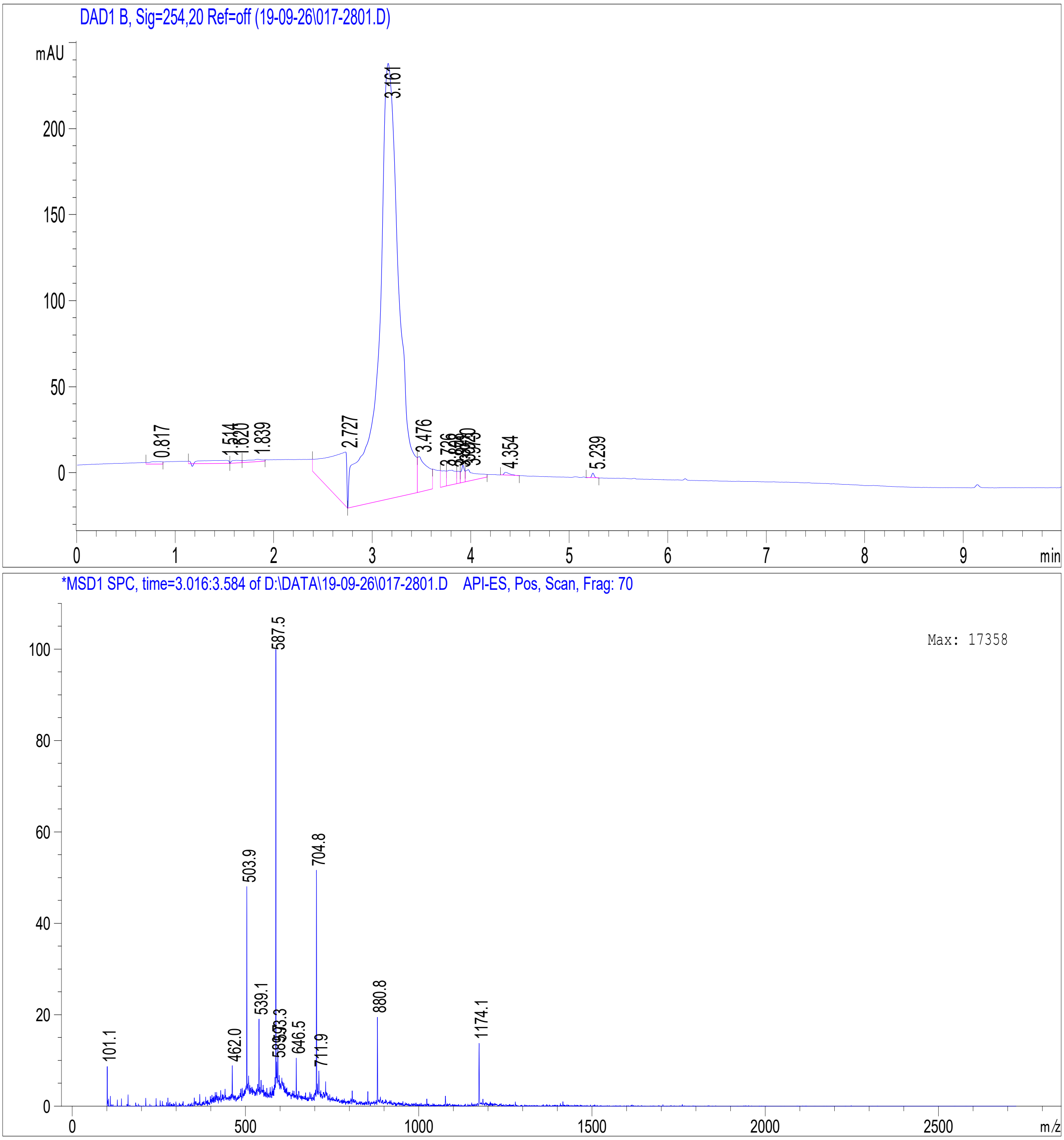

Figure S60: HPLC-MS chromatogram (HPLC1) of purified **PNA-4G**. HPLC-UV trace at 254 nm (top) and MS spectrum of the corresponding peak (bottom). Calcd MW: 3518.5, m/z found: 1174.1 $[M+3H]^{3+}$, 880.8 $[M+4H]^{4+}$, 704.8 $[M+5H]^{5+}$, 587.5 $[M+6H]^{6+}$, 503.9 $[M+7H]^{7+}$.

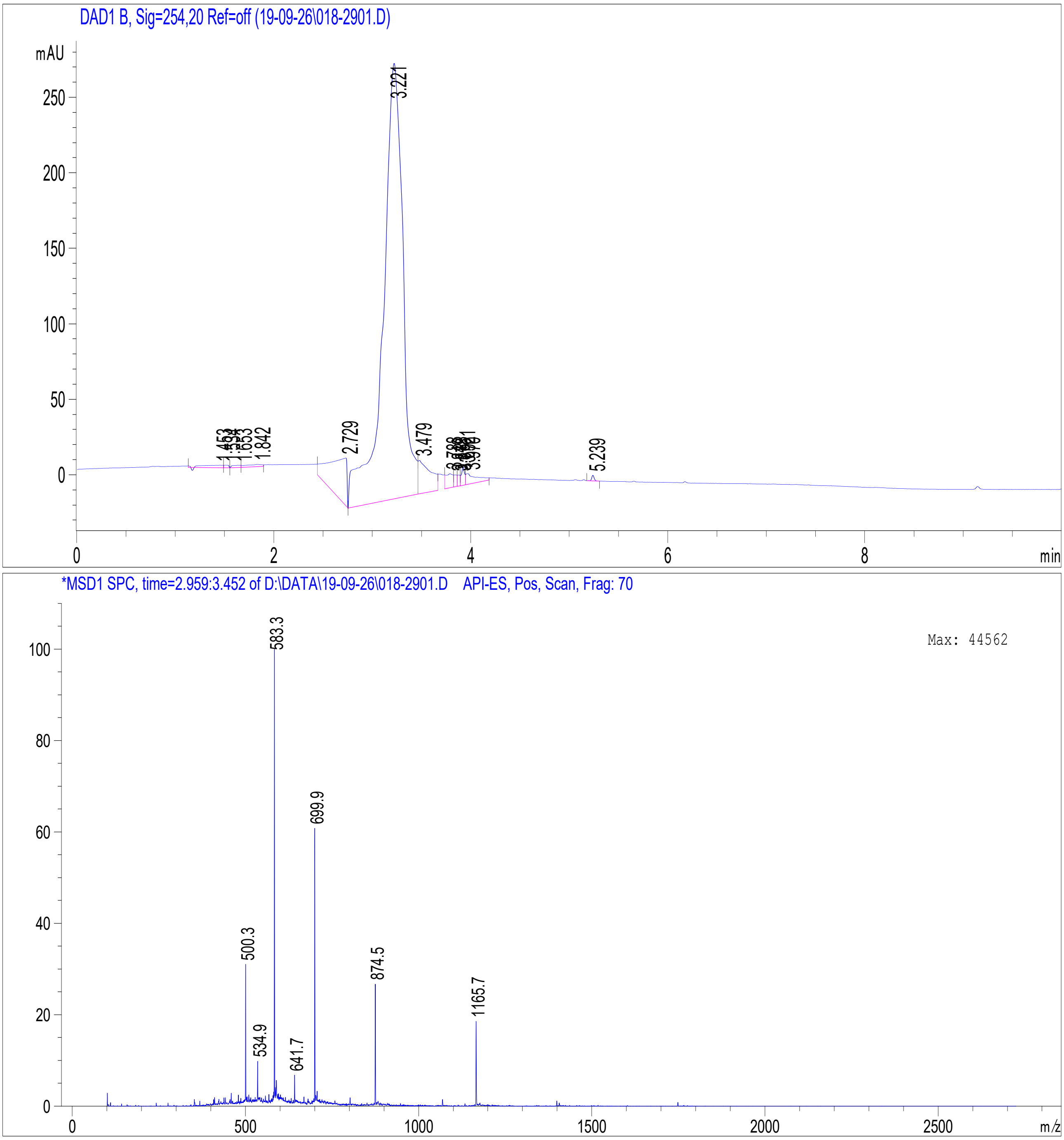

Figure S61: HPLC-MS chromatogram (HPLC1) of purified **PNA-4T**. HPLC-UV trace at 254 nm (top) and MS spectrum of the corresponding peak (bottom). Calcd MW: 3493.5, m/z found: 1165.7 $[M+3H]^{3+}$, 874.5 $[M+4H]^{4+}$, 699.9 $[M+5H]^{5+}$, 583.3 $[M+6H]^{6+}$, 500.3 $[M+7H]^{7+}$.

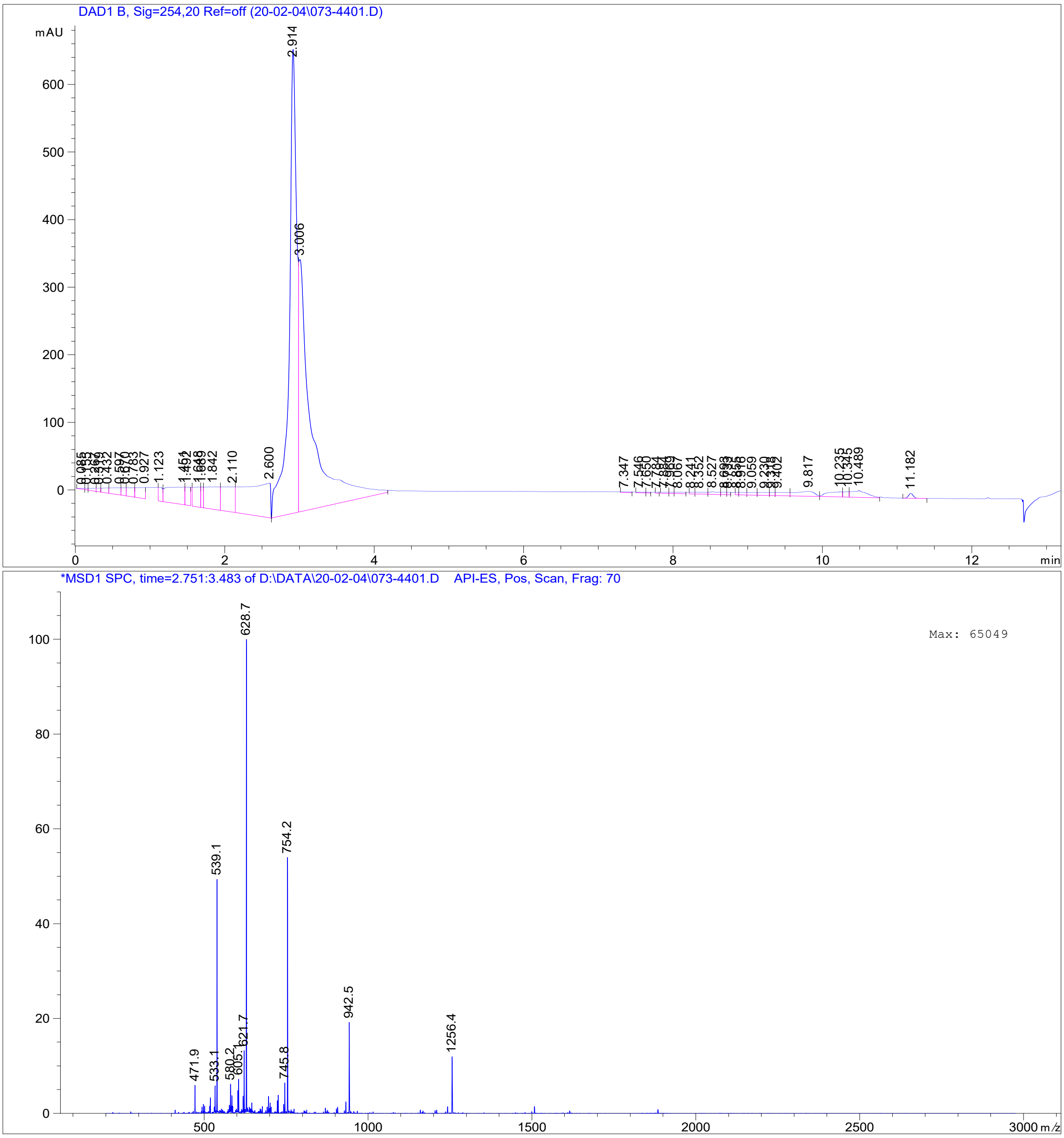

Figure S62: HPLC-MS chromatogram (HPLC1) of purified **PNA-5**. HPLC-UV trace at 254 nm (top) and MS spectrum of the corresponding peak (bottom). Calcd MW 3766.2, m/z found: 1256.4 $[M+3H]^{3+}$, 942.5 $[M+4H]^{4+}$, 754.2 $[M+5H]^{5+}$, 628.7 $[M+6H]^{6+}$, 539.1 $[M+7H]^{7+}$.

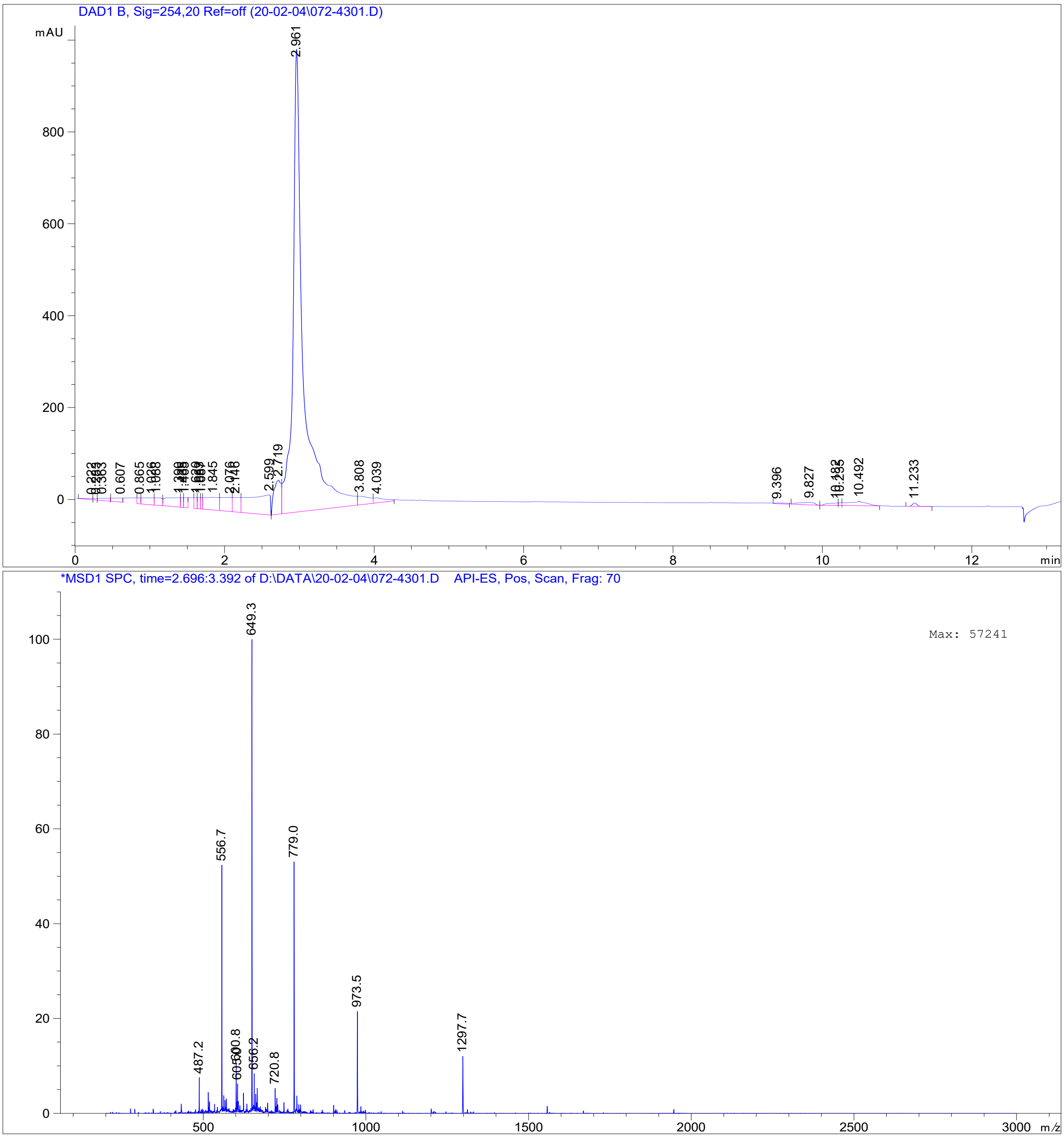

Figure S63: HPLC-MS chromatogram (HPLC1) of purified **PNA-6**. HPLC-UV trace at 254 nm (top) and MS spectrum of the corresponding peak (bottom). Calcd MW: 3889.2, m/z found: 1297.7[M+3H]$^{3+}$, 973.5[M+4H]$^{4+}$, 779.0 [M+5H]$^{5+}$, 649.3[M+6H]$^{6+}$, 556.7 [M+7H]$^{7+}$.

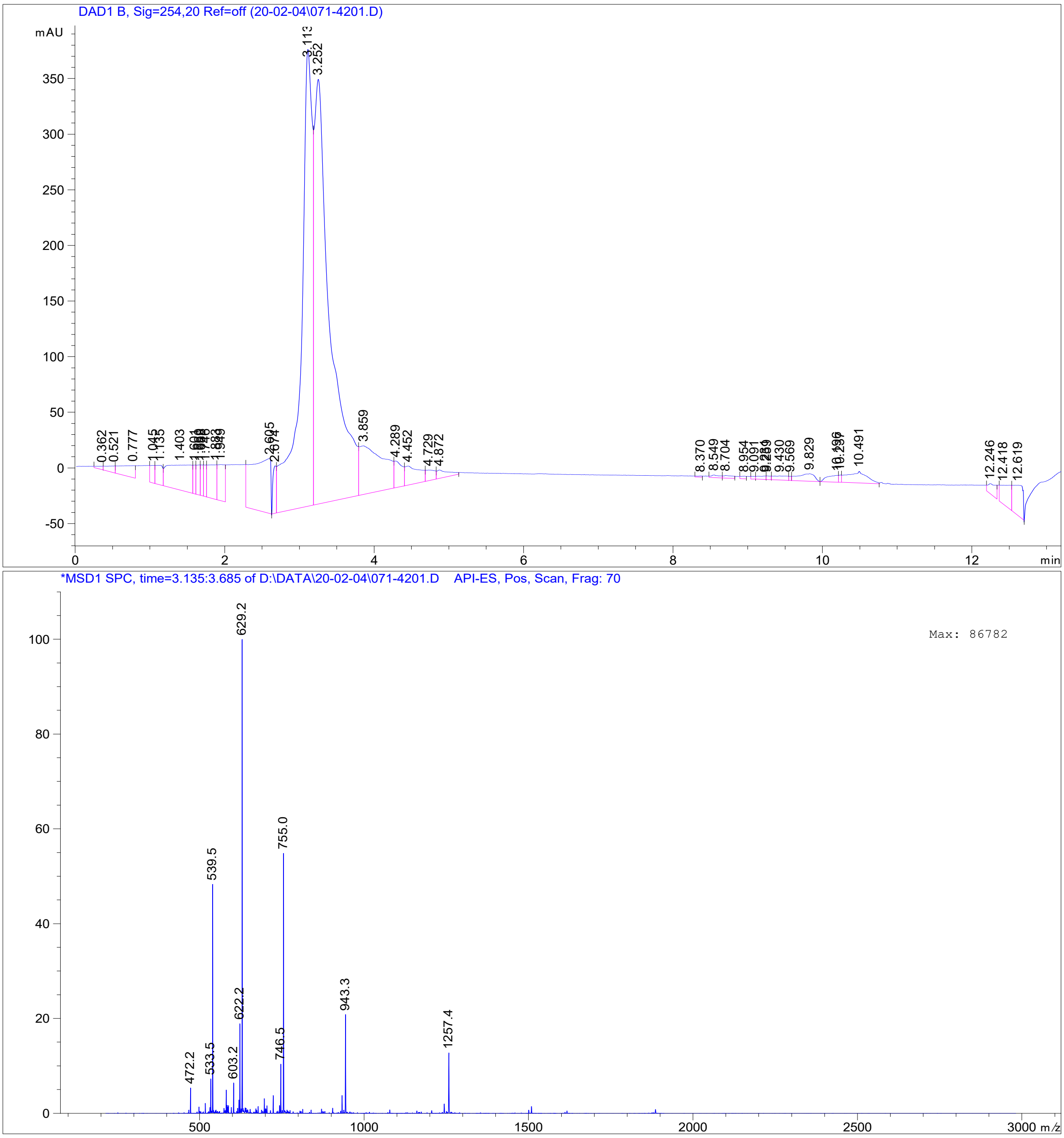

Figure S64: HPLC-MS chromatogram (HPLC1) of purified **PNA-7.** HPLC-UV trace at 254 nm (top) and MS spectrum of the corresponding peak (bottom). Calcd MW: 3768.2, m/z found: 1257.4 $[M+3H]^{3+}$, 943.3 $[M+4H]^{4+}$, 755.0 $[M+5H]^{5+}$, 629.2 $[M+6H]^{6+}$, 539.5 $[M+7H]^{7+}$.

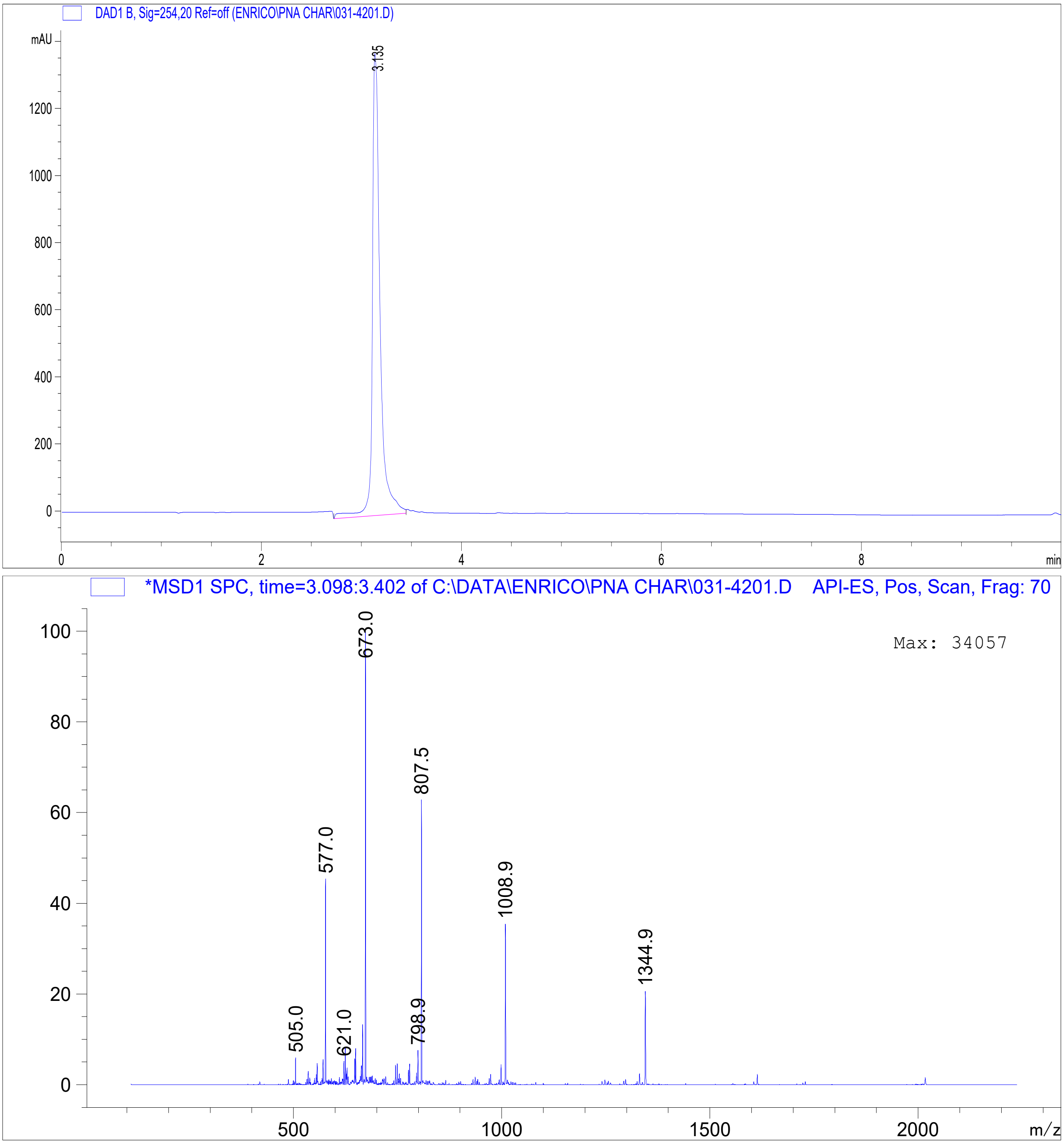

Figure S65: HPLC-MS chromatogram (HPLC1) of purified **PNA-5T**. HPLC-UV trace at 254 nm (top) and MS spectrum of the corresponding peak (bottom). Calcd MW: 4033.0. m/z found: 1344.9 $[M+3H]^{3+}$, 1008.9 $[M+4H]^{4+}$, 807.5 $[M+5H]^{5+}$, 673.0 $[M+6H]^{6+}$, 577.0 $[M+7H]^{7+}$.

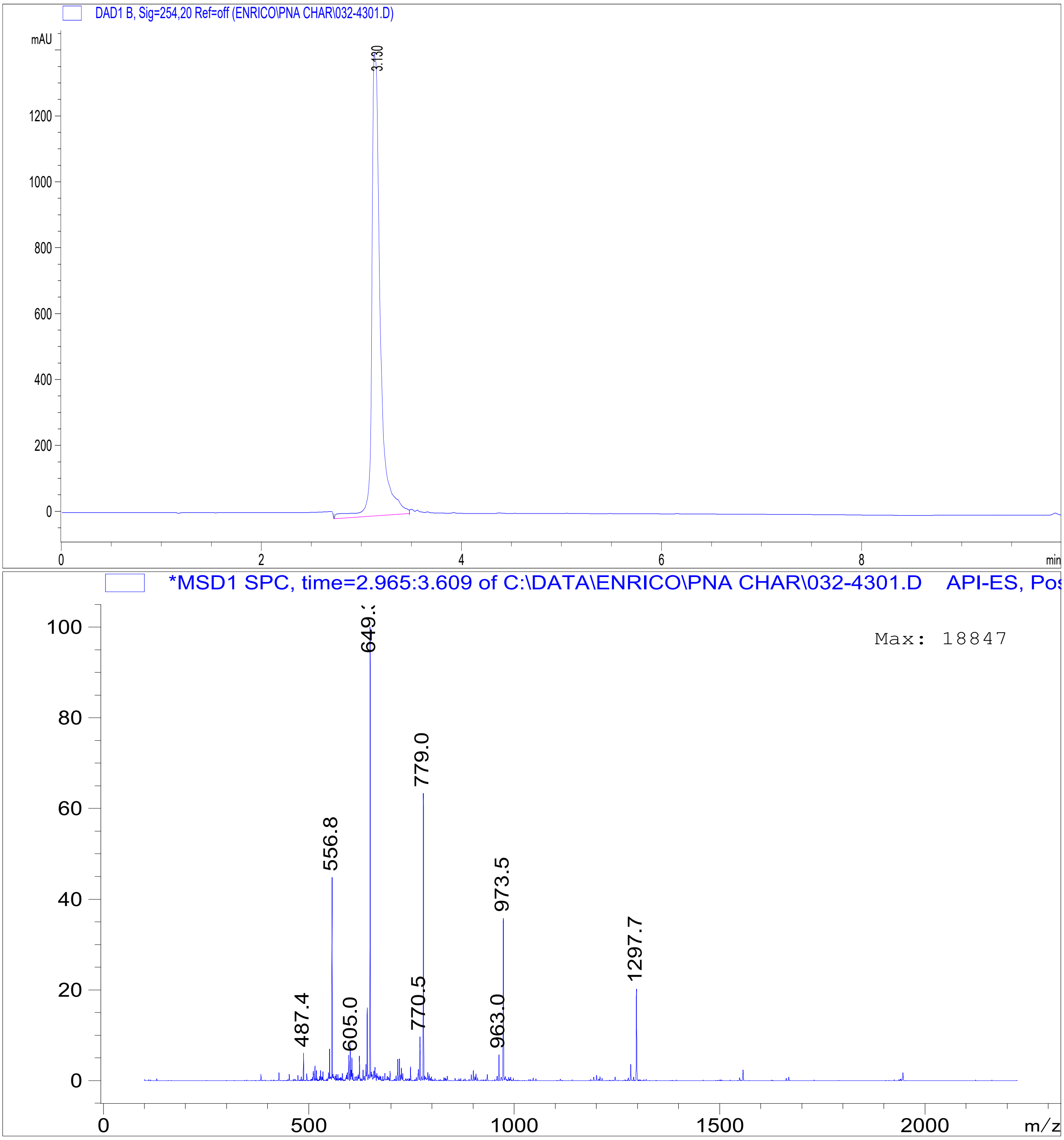

Figure S66: HPLC-MS chromatogram (HPLC1) of purified **PNA-7T**. HPLC-UV trace at 254 nm (top) and MS spectrum of the corresponding peak (bottom). Calcd MW: 3890.9, m/z found: 1297.7 $[M+3H]^{3+}$, 973.5 $[M+4H]^{4+}$, 779.0$[M+5H]^{5+}$, 649.3$[M+6H]^{6+}$, 556.8$[M+7H]^{7+}$.

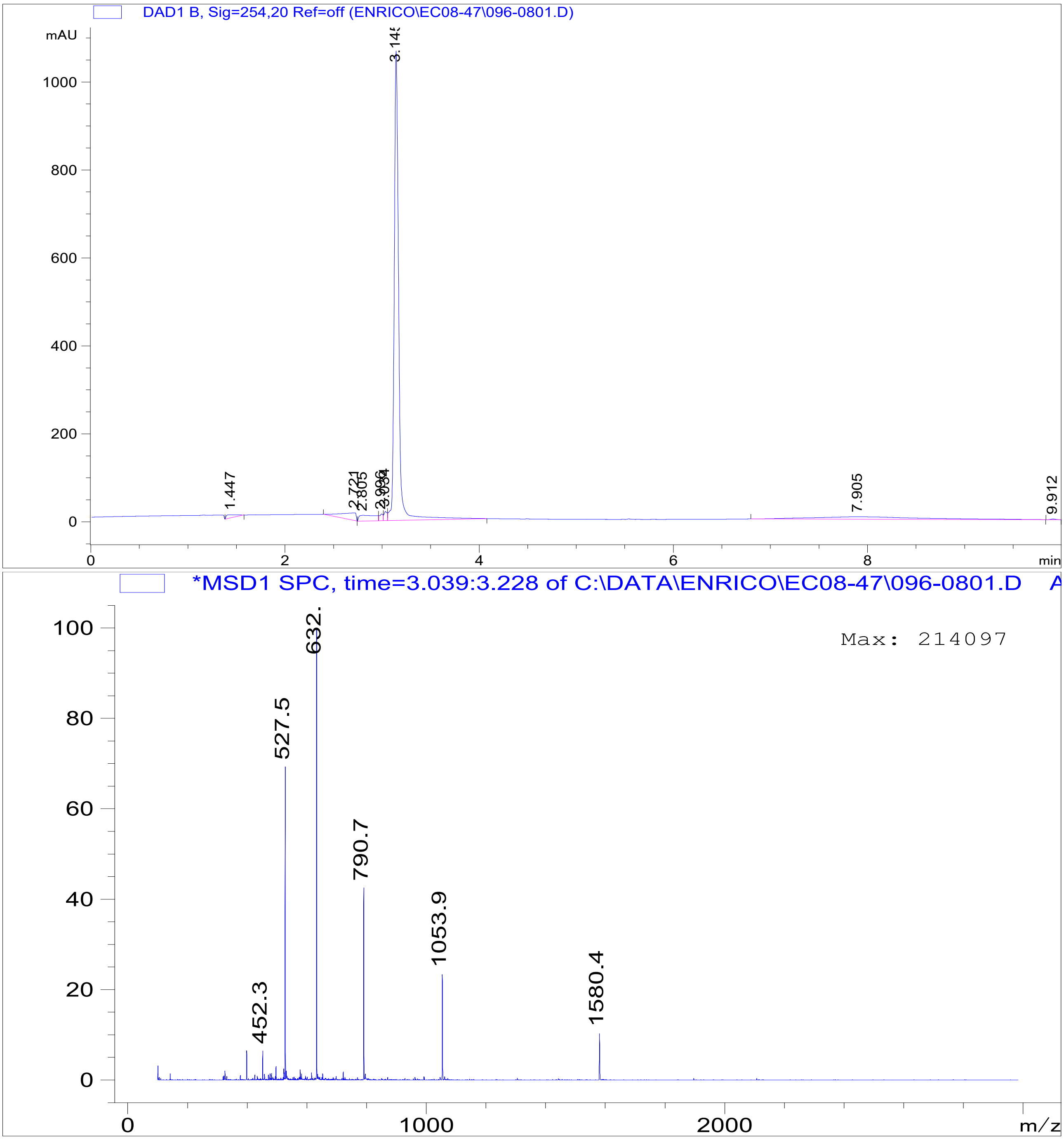

Figure S67: HPLC-MS chromatogram (HPLC1) of purified **PNA-8Ac**. HPLC-UV trace at 254 nm (top) and MS spectrum of the corresponding peak (bottom). Calcd MW: 3160.3, m/z found: 1257.4 $[M+3H]^{3+}$, 943.3 $[M+4H]^{4+}$, 755.0 $[M+5H]^{5+}$, 629.2 $[M+6H]^{6+}$, 539.5 $[M+7H]^{7+}$.

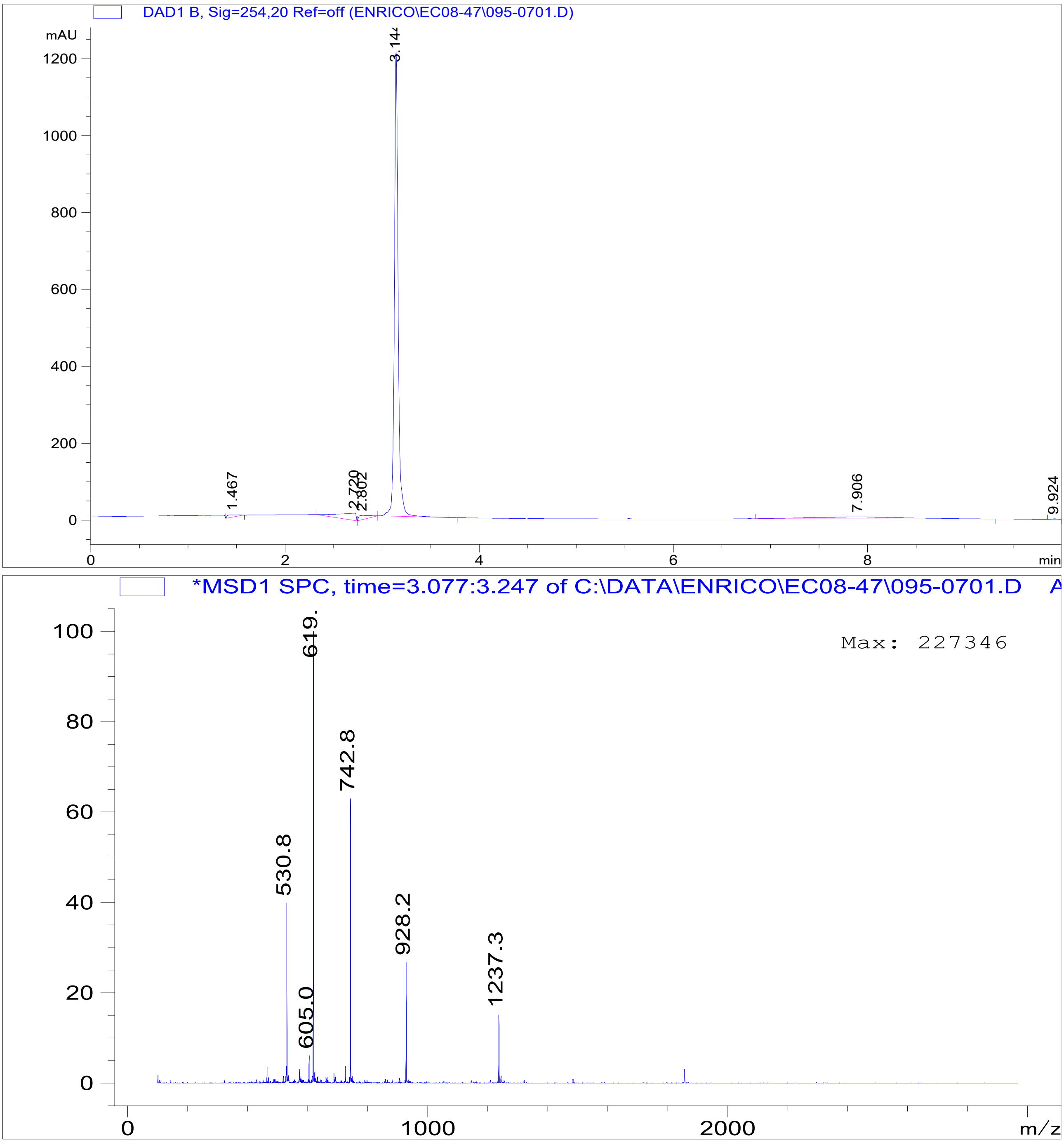

Figure S68: HPLC-MS chromatogram (HPLC1) of purified **PNA-9Ac**. HPLC-UV trace at 254 nm (top) and MS spectrum of the corresponding peak (bottom). Calcd MW: 3710.8, m/z found: 1237.3 $[M+3H]^{3+}$, 928.2 $[M+4H]^{4+}$, 742.8 $[M+5H]^{5+}$, 619.2 $[M+6H]^{6+}$, 530.8 $[M+7H]^{7+}$.

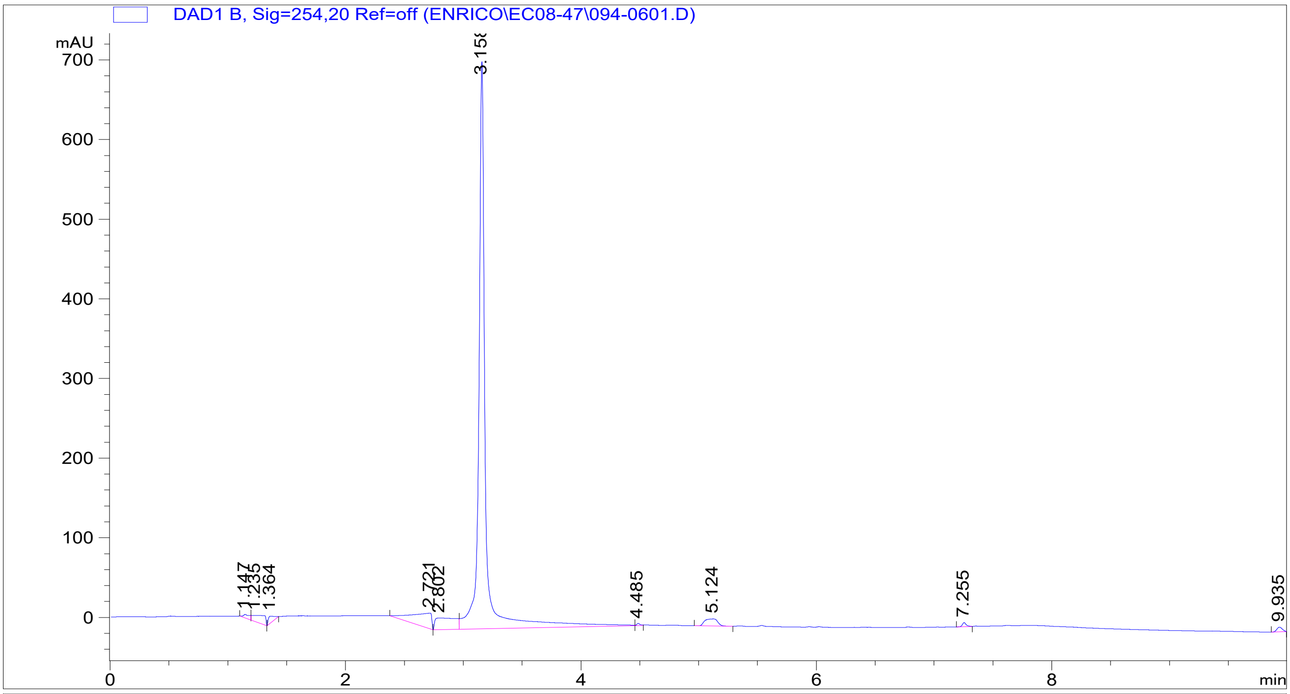

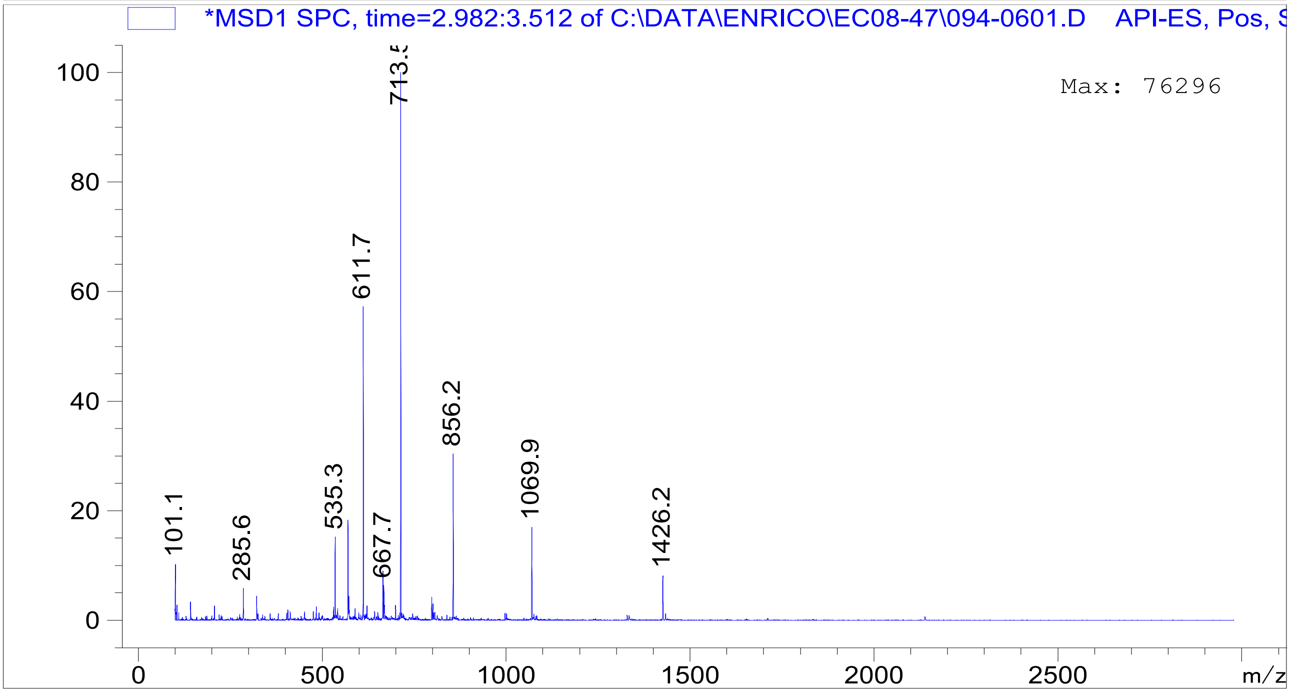

Figure S69: HPLC-MS chromatogram (HPLC1) of purified **PNA-10Ac**. HPLC-UV trace at 254 nm (top) and MS spectrum of the corresponding peak (bottom). Calcd MW: 4275.5, m/z found: 1426.2 $[M+3H]^{3+}$, 1069.9 $[M+4H]^{4+}$, 856.2 $[M+5H]^{5+}$, 713.5 $[M+6H]^{6+}$, 611.7 $[M+7H]^{7+}$.

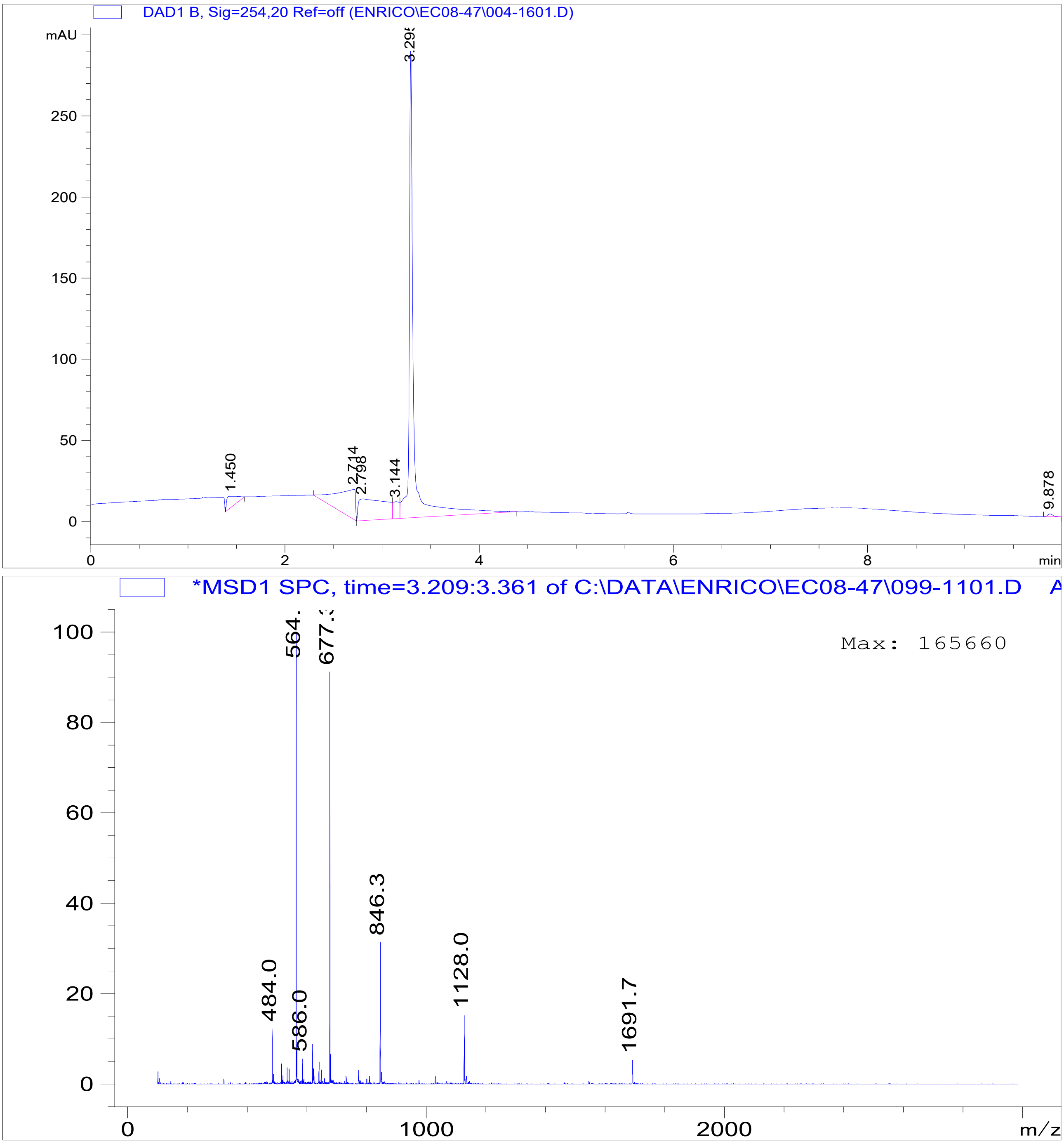

Figure S70: HPLC-MS chromatogram (HPLC1) of purified **PNA-8F**. HPLC-UV trace at 254 nm (top) and MS spectrum of the corresponding peak (bottom). Calcd MW: 3382.5, m/z found: 1691.7 $[M+2H]^{2+}$, 1128.0 $[M+3H]^{3+}$, 846.3 $[M+4H]^{4+}$, 677.3 $[M+5H]^{5+}$, 564.5 $[M+6H]^{6+}$.

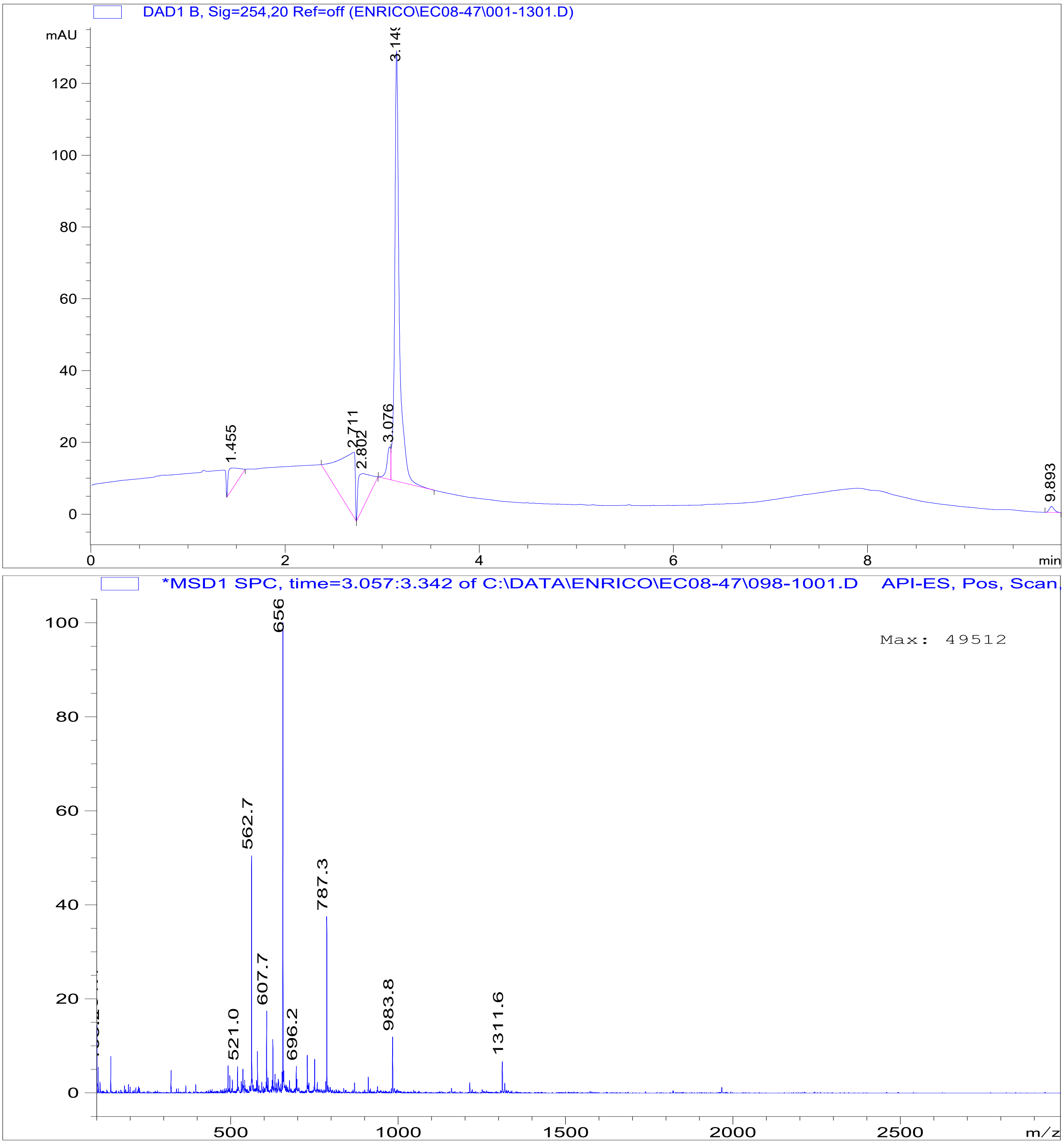

Figure S71: HPLC-MS chromatogram (HPLC1) of purified **PNA-9F**. HPLC-UV trace at 254 nm (top) and MS spectrum of the corresponding peak (bottom). Calcd MW: 3933.0. m/z found: 1331.6 $[M+3H]^{3+}$, 983.3 $[M+4H]^{4+}$, 787.3 $[M+5H]^{5+}$, 656.3$[M+6H]^{6+}$, 562.7 $[M+7H]^{7+}$.

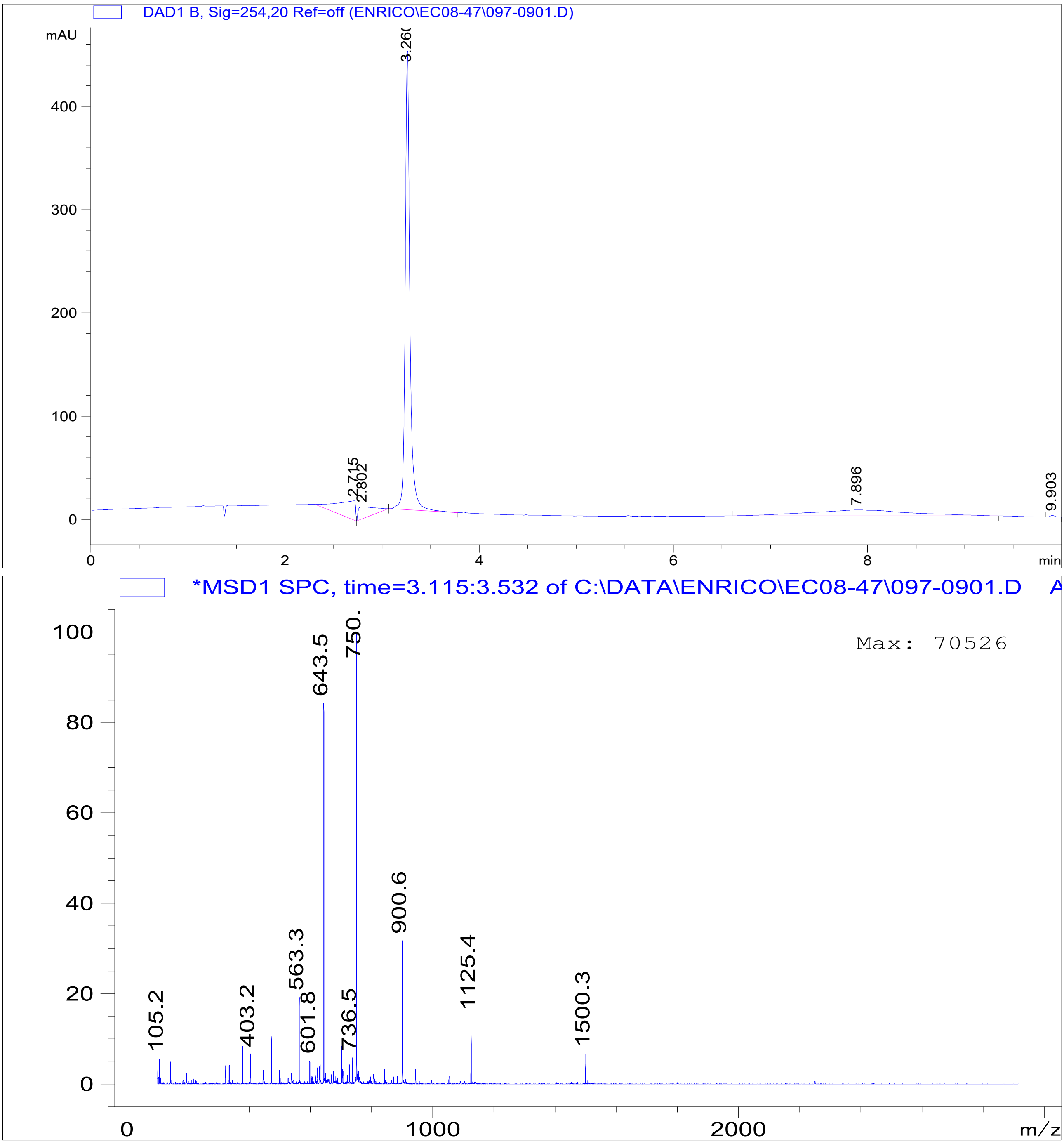

Figure S72: HPLC-MS chromatogram (HPLC1) of purified **PNA-10F**. HPLC-UV trace at 254 nm (top) and MS spectrum of the corresponding peak (bottom). Calcd MW: 4499.6. m/z found: 1500.3[M+3H]$^{3+}$, 1125.4 [M+4H]$^{4+}$, 900.6 [M+5H]$^{5+}$, 750.6 [M+6H]$^{6+}$, 643.5 [M+7H]$^{7+}$.

11.Representative HPLC traces of crosslink experiments

In view of the large amount of repetitive data presented in this study, we included here a representative selection of ICL experiments. Additional information and raw data are available upon request.

In a typical ICL experiment, the chromatograms at 0 eq. NBS (or 0' irradiation in presence of a PS), containing DNA or RNA starting material and PNA probe (blue trace) are superimposed with the chromatogram obtained upon addition of 4 eq. of NBS or after irradiation in presence of a PS (red trace). Typically, the PNA probe is not clearly visible, due to the HPLC condition optimized for DNA detection (not ideal for peptide detection). Upon ICL reaction, the peak corresponding to the starting material decreases, leading to the formation of ICL product at higher retention times (**Figure S59**).

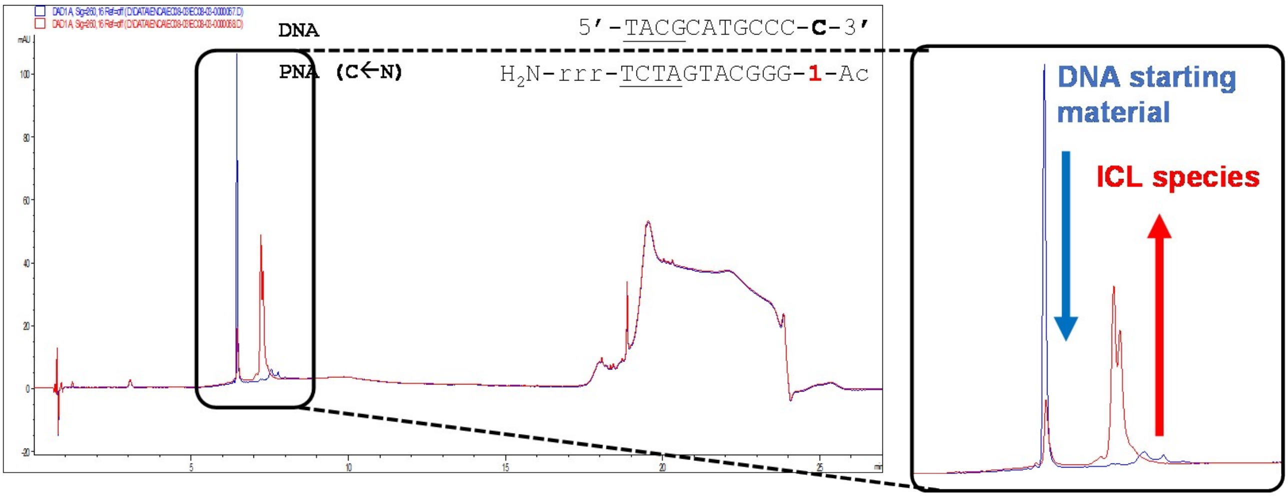

FigureS73. Representative chromatogram showing the reaction between DNA-2-C and PNA-5, at 0' (blue trace) and 20' irradiation time (red trace), in presence of MB.

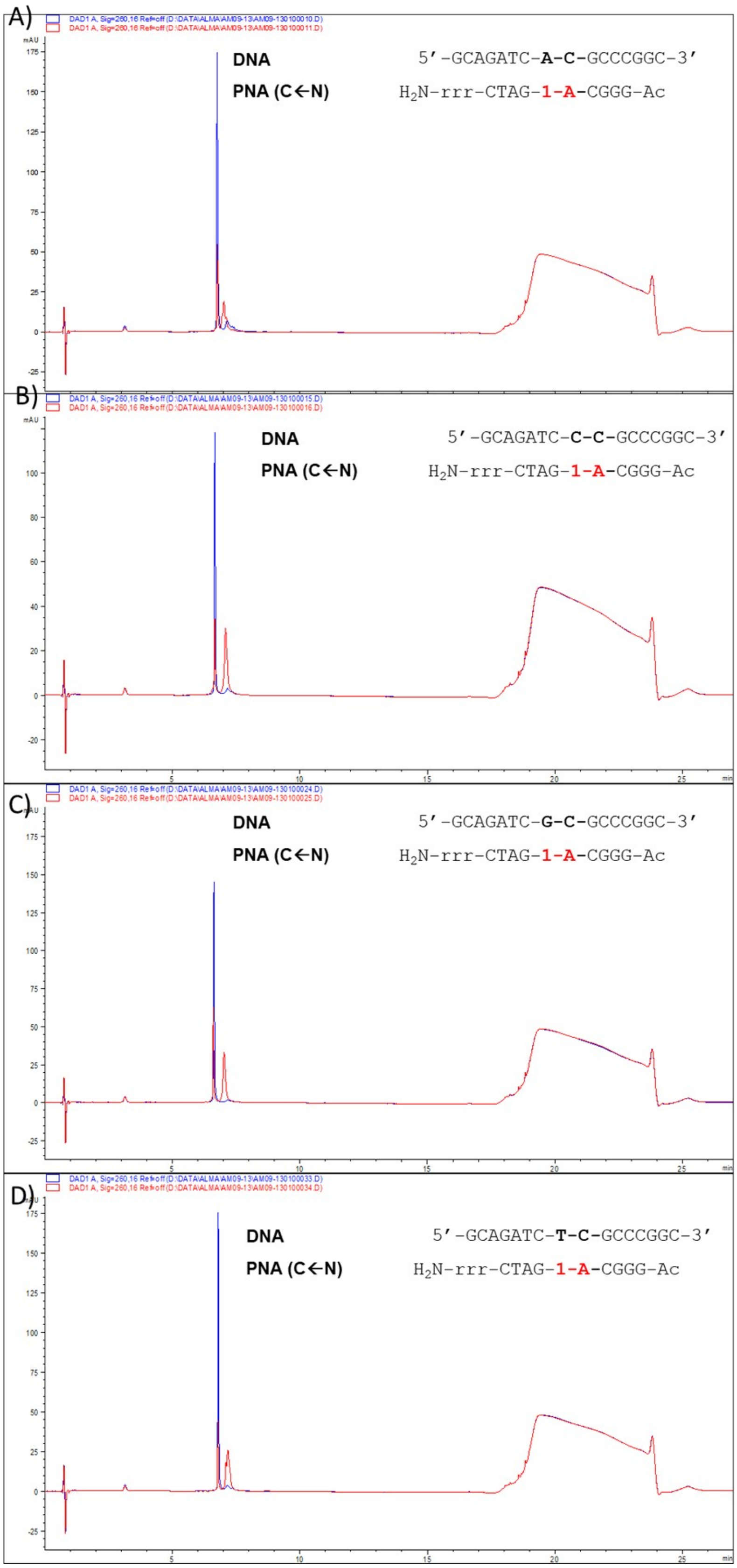

Figure S74: HPLC3 traces of crosslink experiment performed in presence of **PNA-1A** in presence of **DNA-1-XY** at 0 eq (blue trace) and 4 eq NBS (red trace)**.** (A) **PNA-1A + DNA-1-AC;** (B) **PNA-1A + DNA-1-CC;** (C) **PNA-1A + DNA-1-GC;** (D) **PNA-1A + DNA-1-TC.**

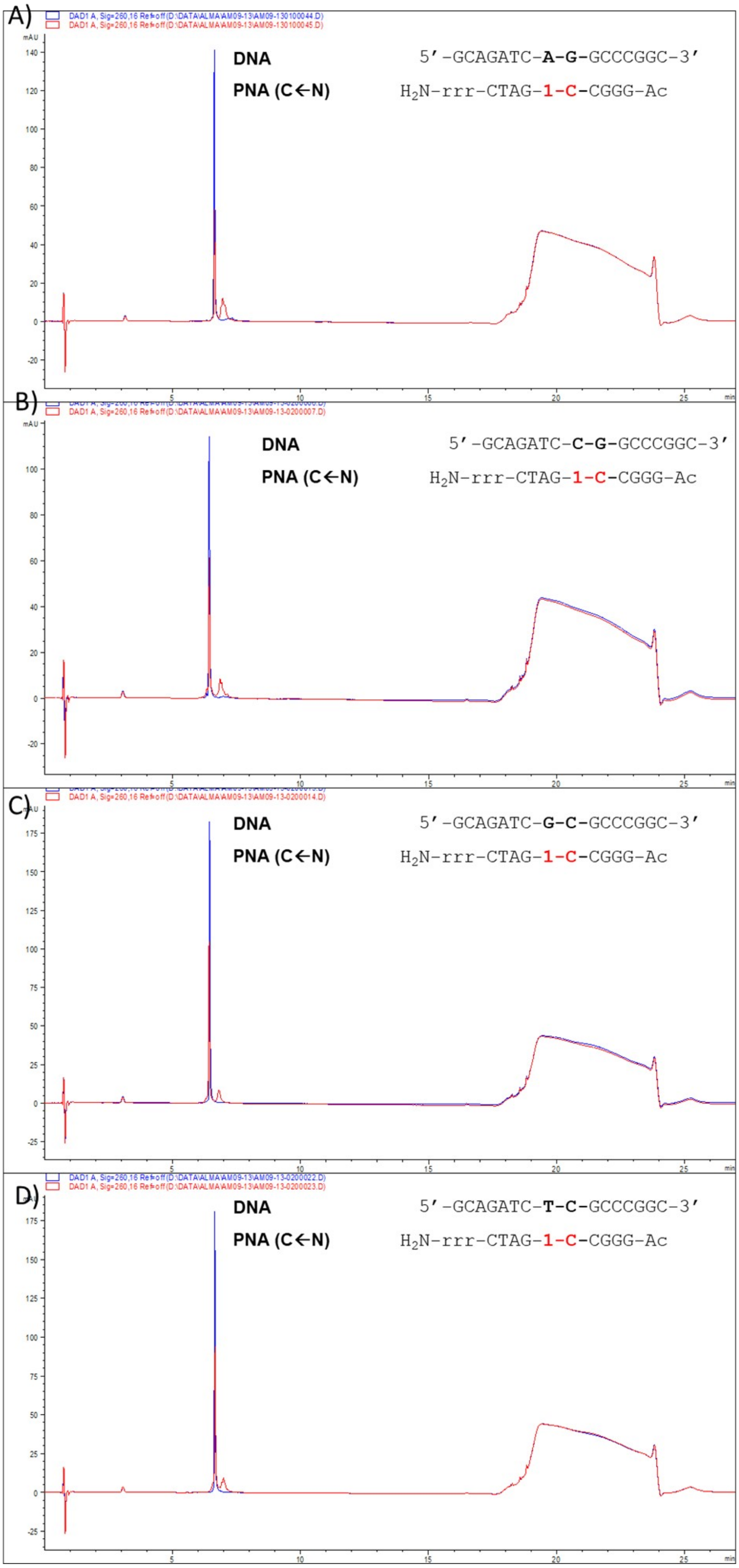

Figure S75: HPLC3 traces of crosslink experiment performed in presence of **PNA-1C** in presence of **DNA-1-XY** at 0 eq (blue trace) and 4 eq NBS (red trace)**.** (A) **PNA-1C + DNA-1-AG;** (B) **PNA-1C + DNA-1-CG;** (C) **PNA-1C + DNA-1-GC;** (D) **PNA-1C + DNA-1-TC.**

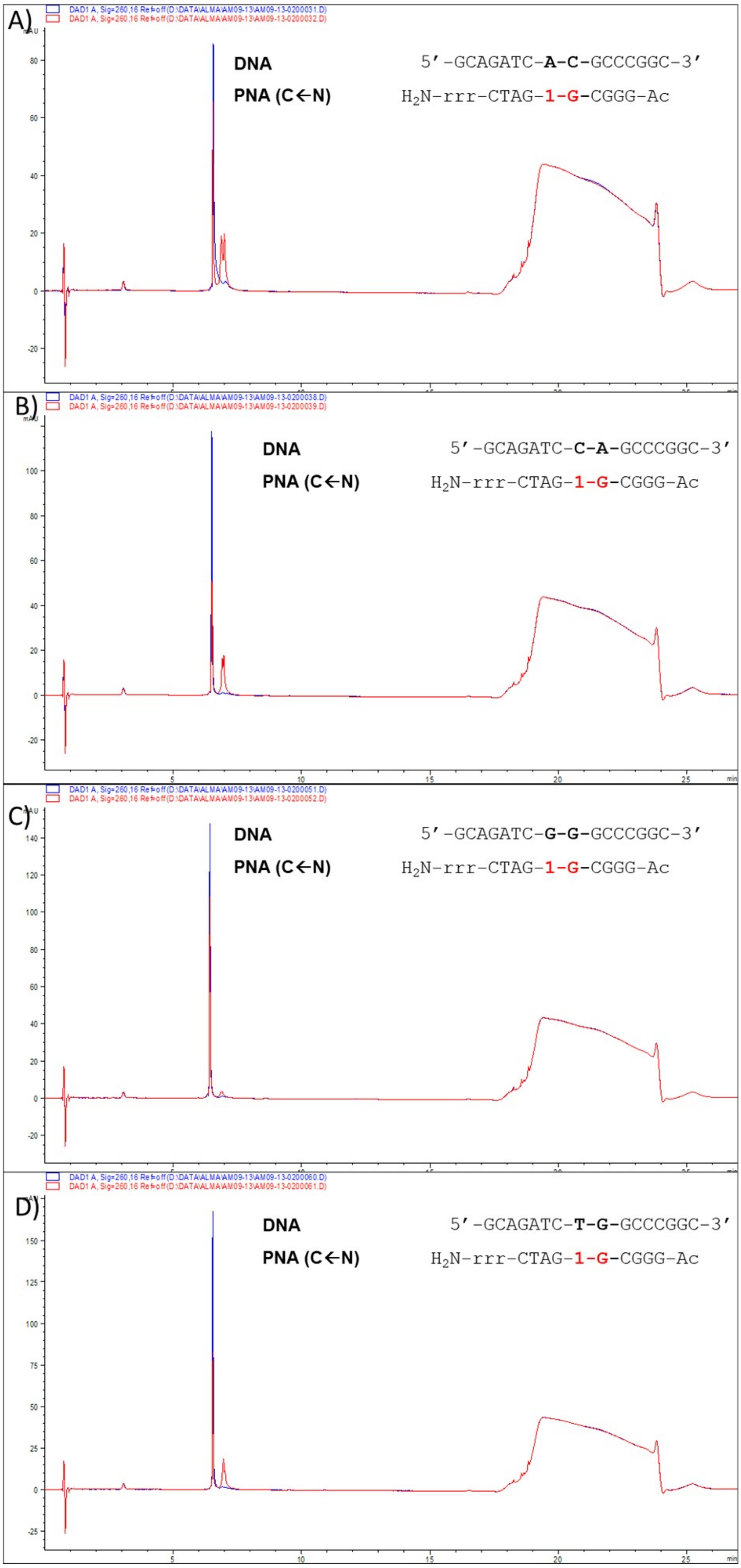

Figure S76: HPLC3 traces of crosslink experiment performed in presence of **PNA-1G** in presence of **DNA-1-XY** at 0 eq (blue trace) and 4 eq NBS (red trace). (A) **PNA-1G + DNA-1-AC;** (B) **PNA-1G + DNA-1-CA;** (C) **PNA-1G + DNA-1-GG;** (D) **PNA-1G + DNA-1-TG.**

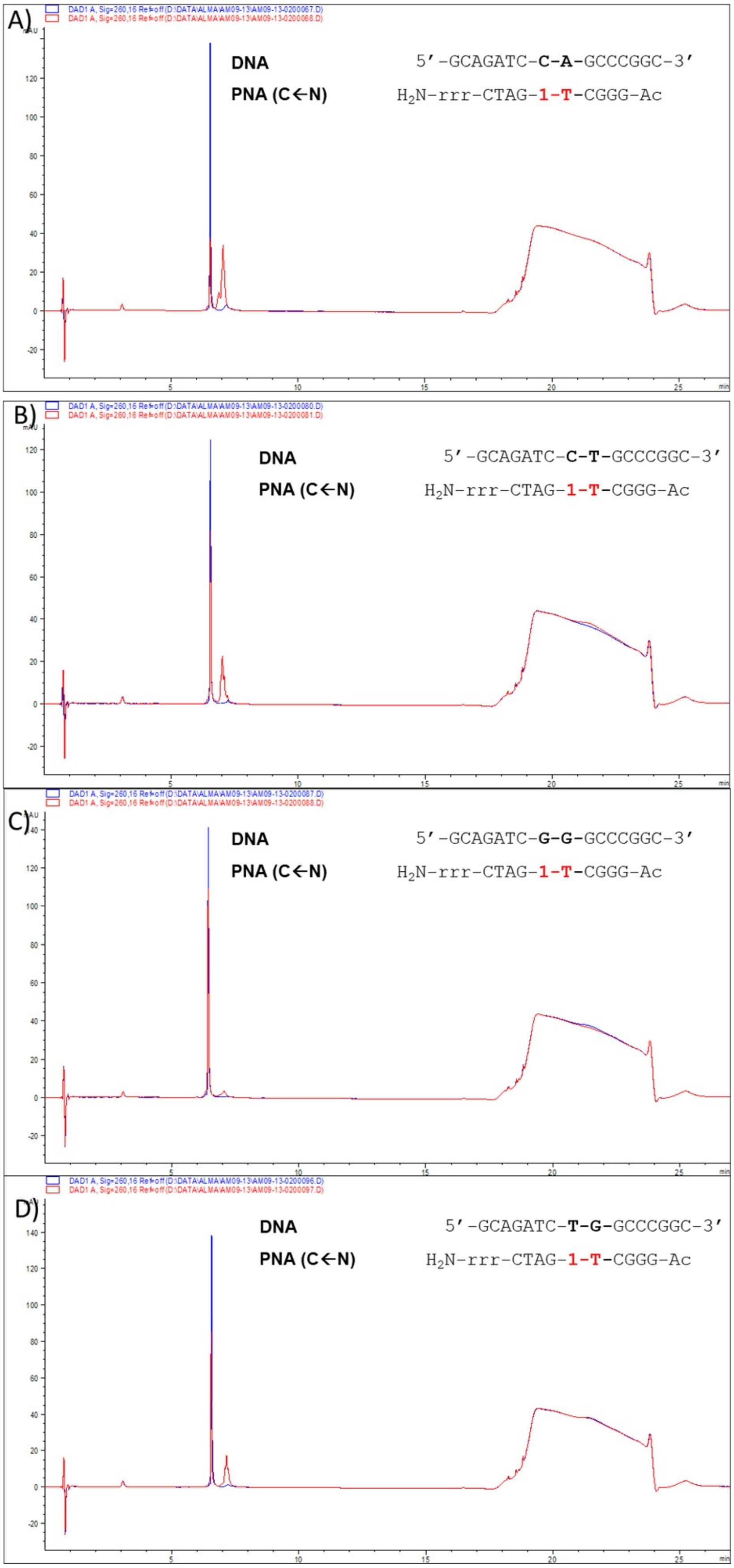

Figure S77: HPLC3 traces of crosslink experiment performed in presence of **PNA-1T** in presence of **DNA-1-XY** at 0 eq (blue trace) and 4 eq NBS (red trace)**.** (A) **PNA-1T + DNA-1-CA;** (B) **PNA-1T + DNA-1-CT;** (C) **PNA-1T + DNA-1-GG;** (D) **PNA-1T + DNA-1-TG.**

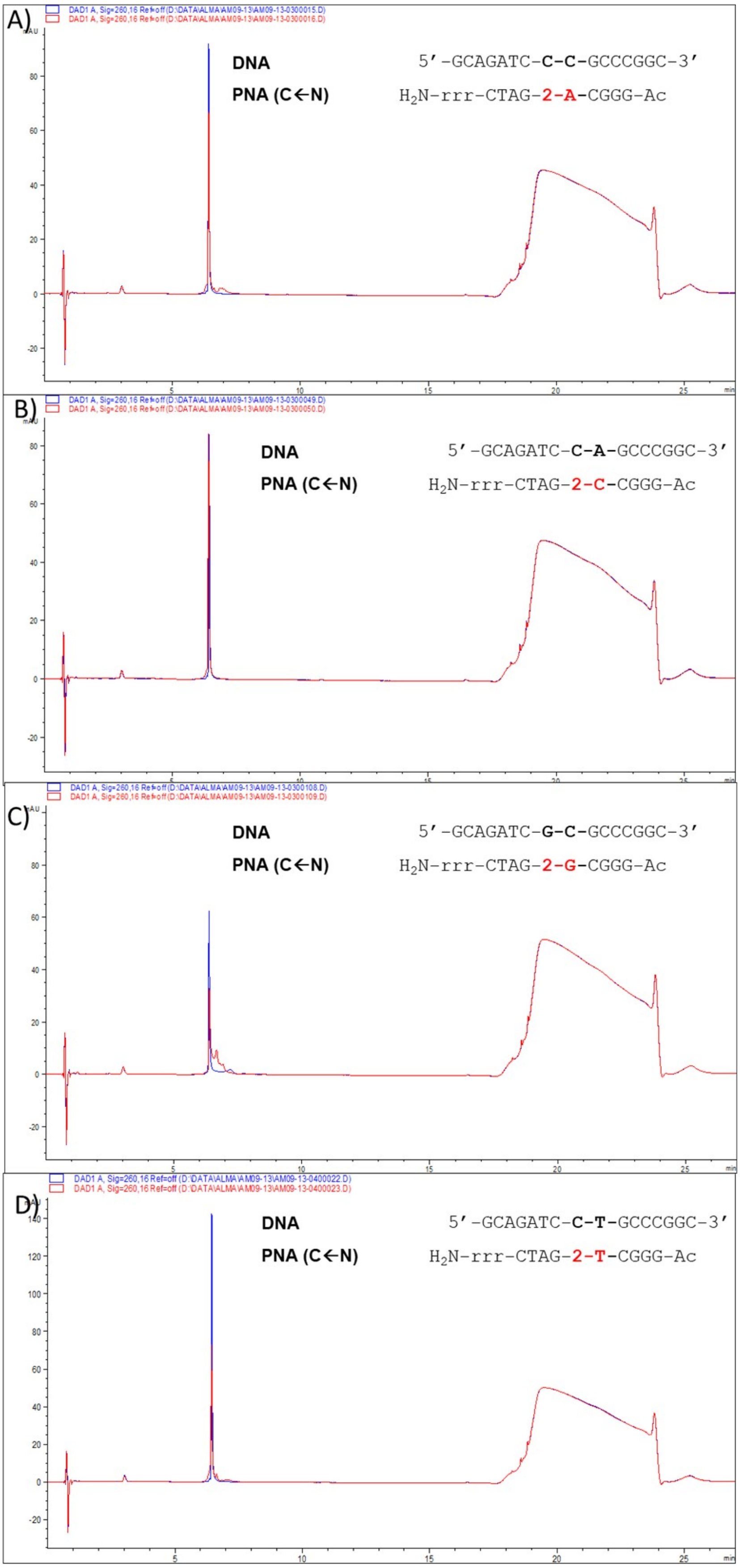

Figure S78: HPLC3 traces of crosslink experiment performed in presence of **PNA-2B** in presence of **DNA-1-XY** at 0 eq (blue trace) and 4 eq NBS (red trace). (A) **PNA-2A + DNA-1-CC;** (B) **PNA-2C + DNA-1-CA;** (C) **PNA-2G + DNA-1-GC;** (D) **PNA-2T + DNA-1-CT.**

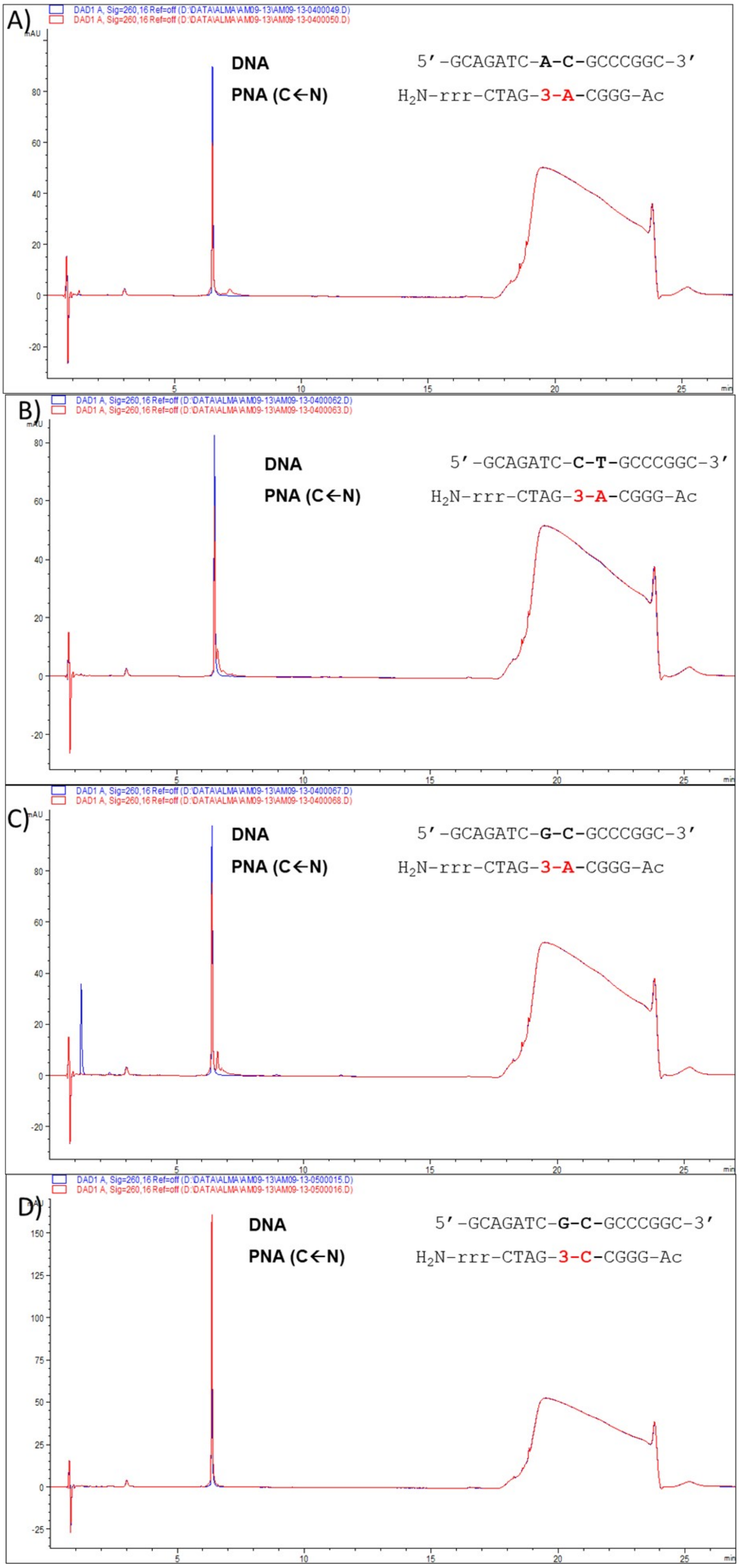

Figure S79: HPLC3 traces of crosslink experiment performed in presence of **PNA-3B** in presence of **DNA-1-XY** at 0 eq (blue trace) and 4 eq NBS (red trace)**.** (A) **PNA-3A + DNA-1-AC;** (B) **PNA-3A + DNA-1-CT;** (C) **PNA-3A + DNA-1-GC;** (D) **PNA-3C + DNA-1-GC.**

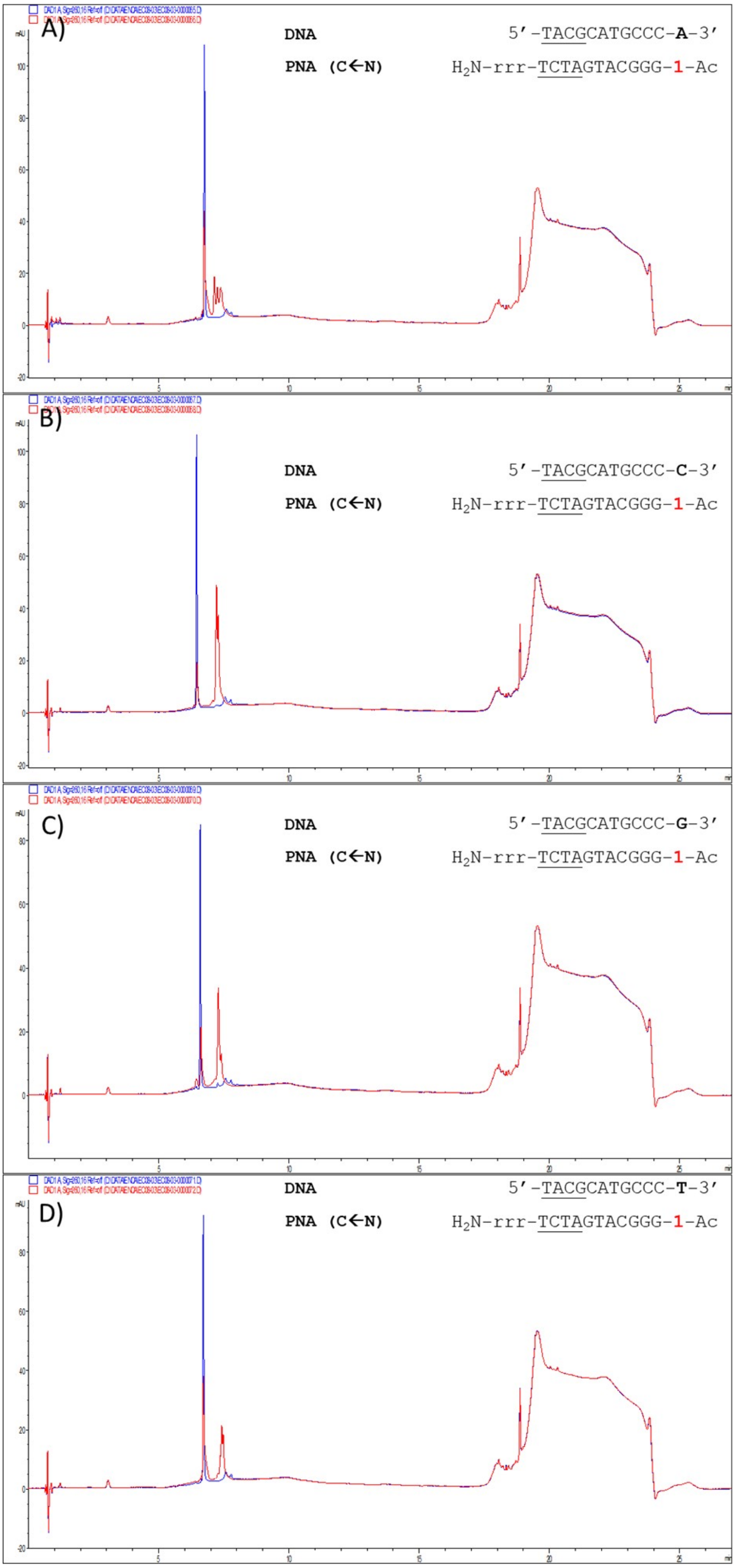

Figure S80: HPLC3 traces of crosslink experiment performed in presence of **PNA-5** in presence of **DNA-2-Z** at 0 (blue trace) and 20’ irradiation, at 2 μM MB concentration (red trace). (A) + **DNA-2-A;** (B) + **DNA-2-C;** (C) + **DNA-2-G;** (D) + **DNA-2-T.**

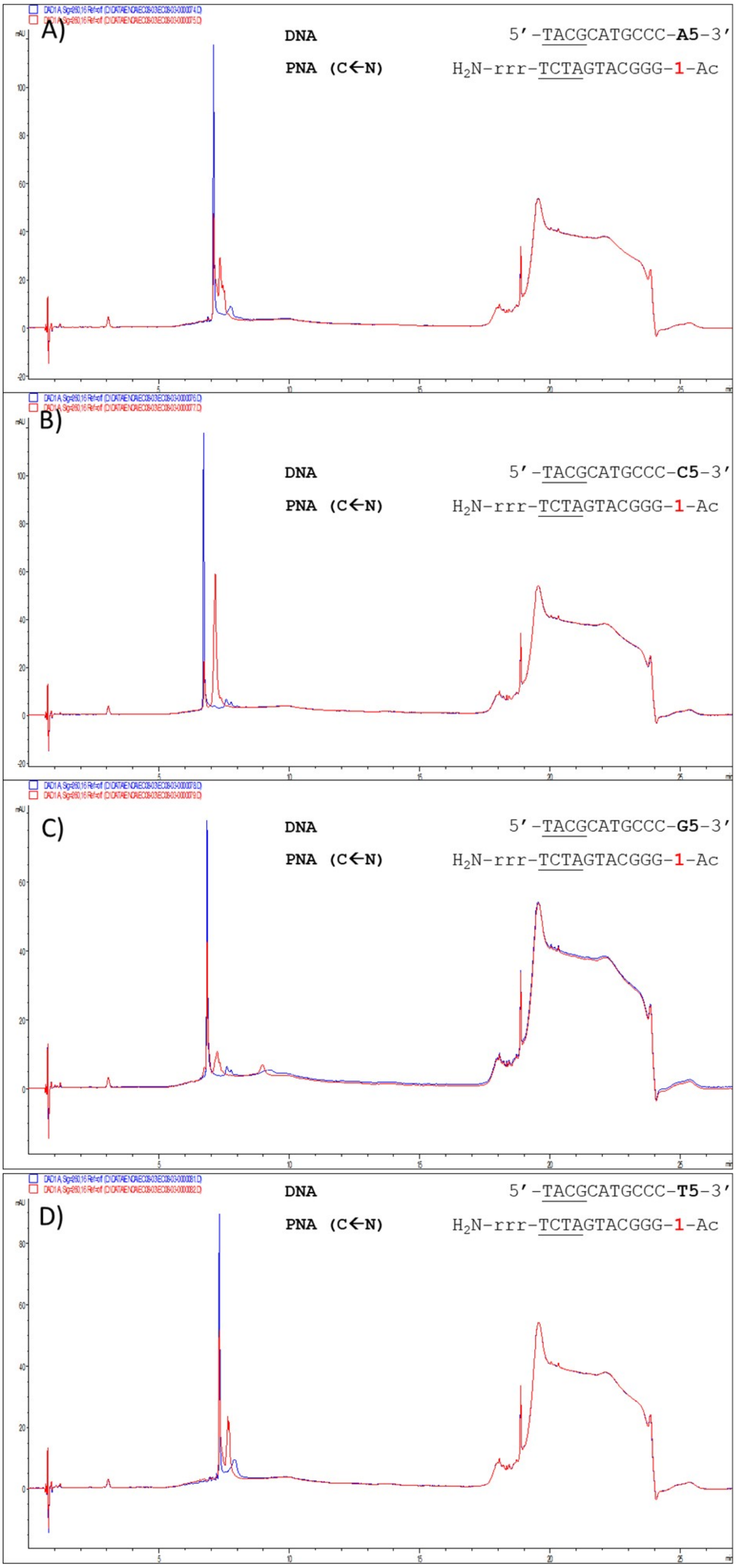

Figure S81: HPLC3 traces of crosslink experiment performed in presence of **PNA-5** in presence of **DNA-2-Z** at 0 (blue trace) and 20’ irradiation, at 2 μM MB concentration (red trace)**.** (A) + **DNA-2-A5;** (B) + **DNA-2-C5;** (C) + **DNA-2-G5;** (D) + **DNA-2-T5.**

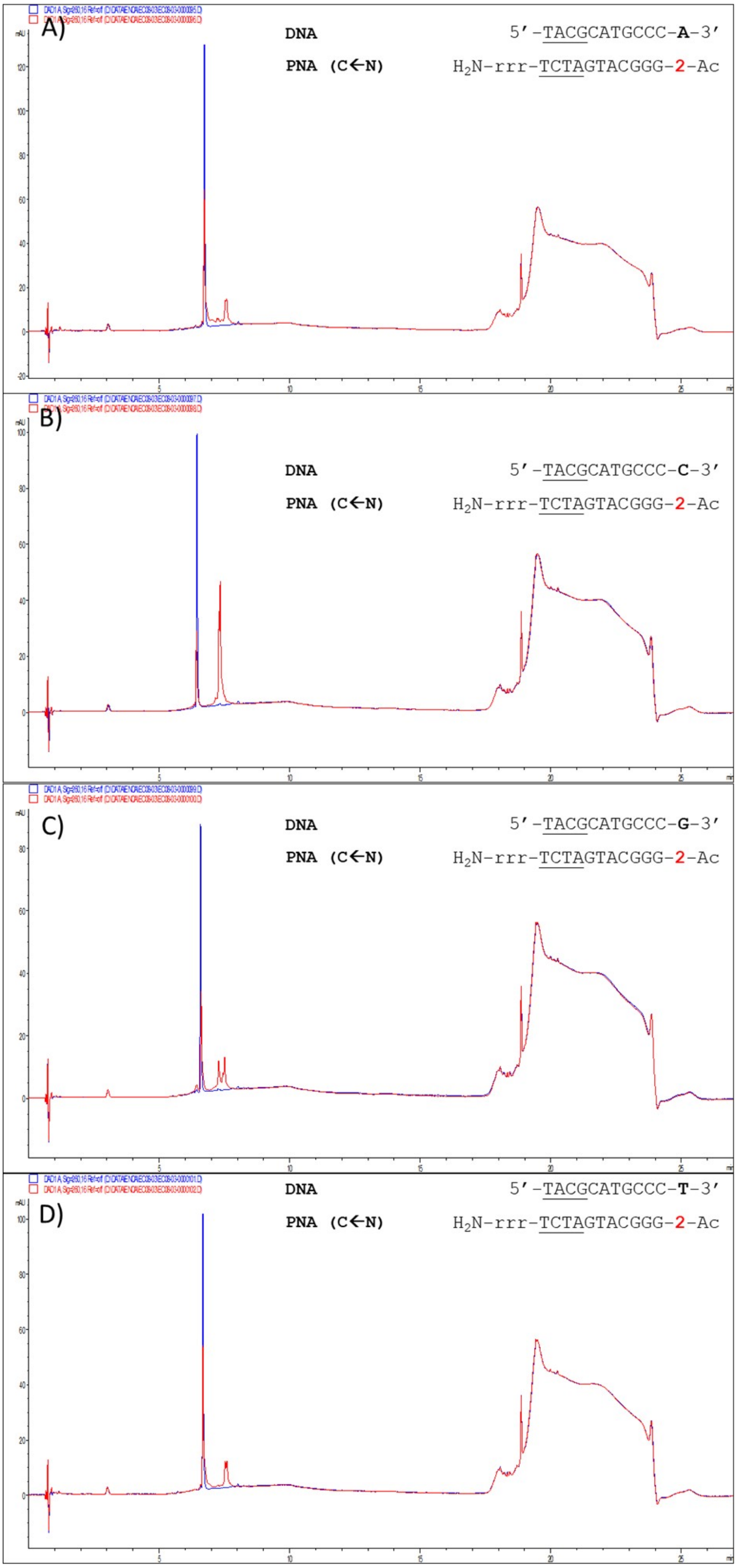

Figure S82: HPLC3 traces of crosslink experiment performed in presence of **PNA-6** in presence of **DNA-2-Z** at 0 (blue trace) and 20’ irradiation, at 2 μM MB concentration (red trace). (A) + **DNA-2-A;** (B) + **DNA-2-C;** (C) + **DNA-2-G;** (D) + **DNA-2-T.**

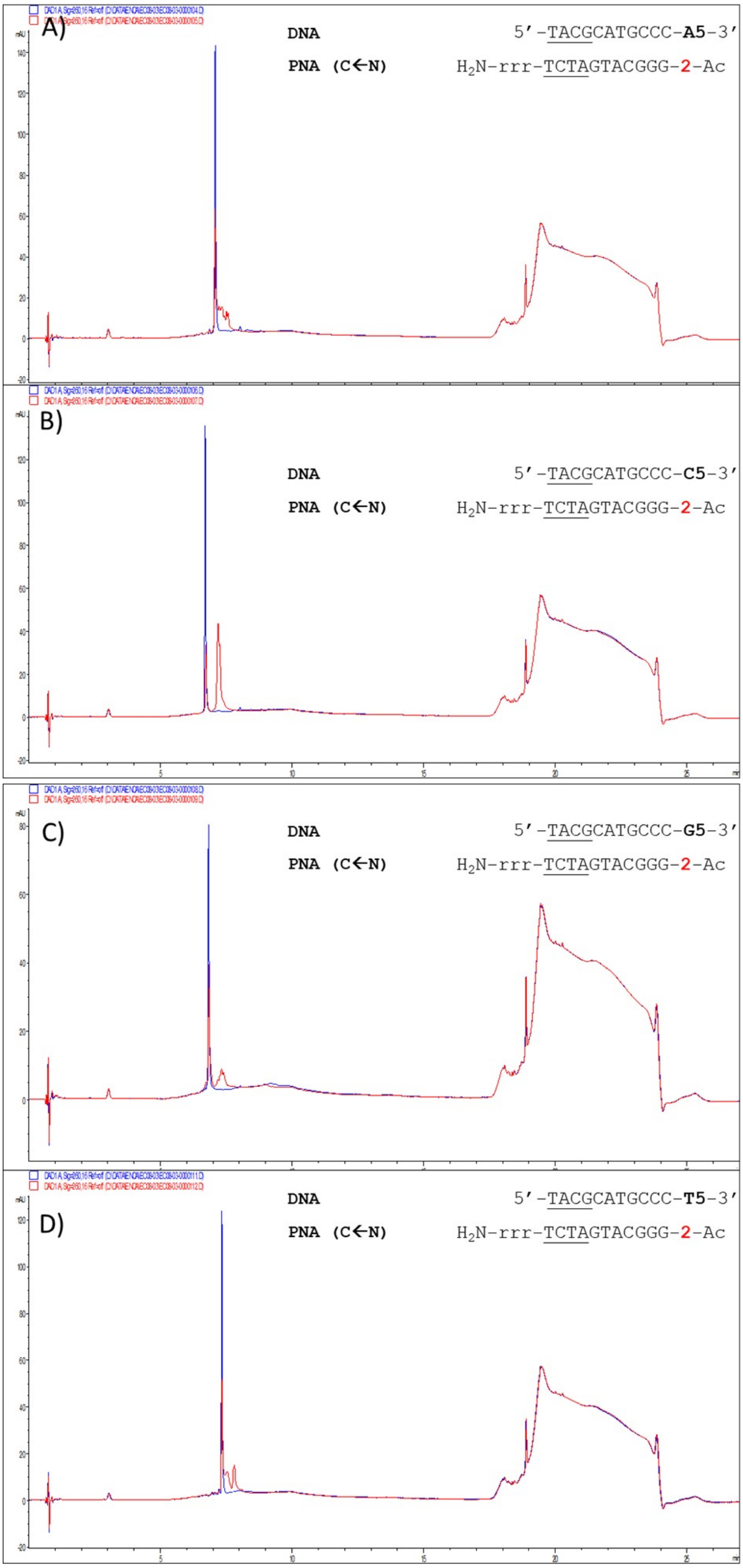

Figure S83: HPLC3 traces of crosslink experiment performed in presence of **PNA-6** in presence of **DNA-2-Z** at 0 (blue trace) and 20’ irradiation, at 2 µM MB concentration (red trace)**.** (A) + **DNA-2-A5;** (B) + **DNA-2-C5;** (C) + **DNA-2-G5;** (D) + **DNA-2-T5.**

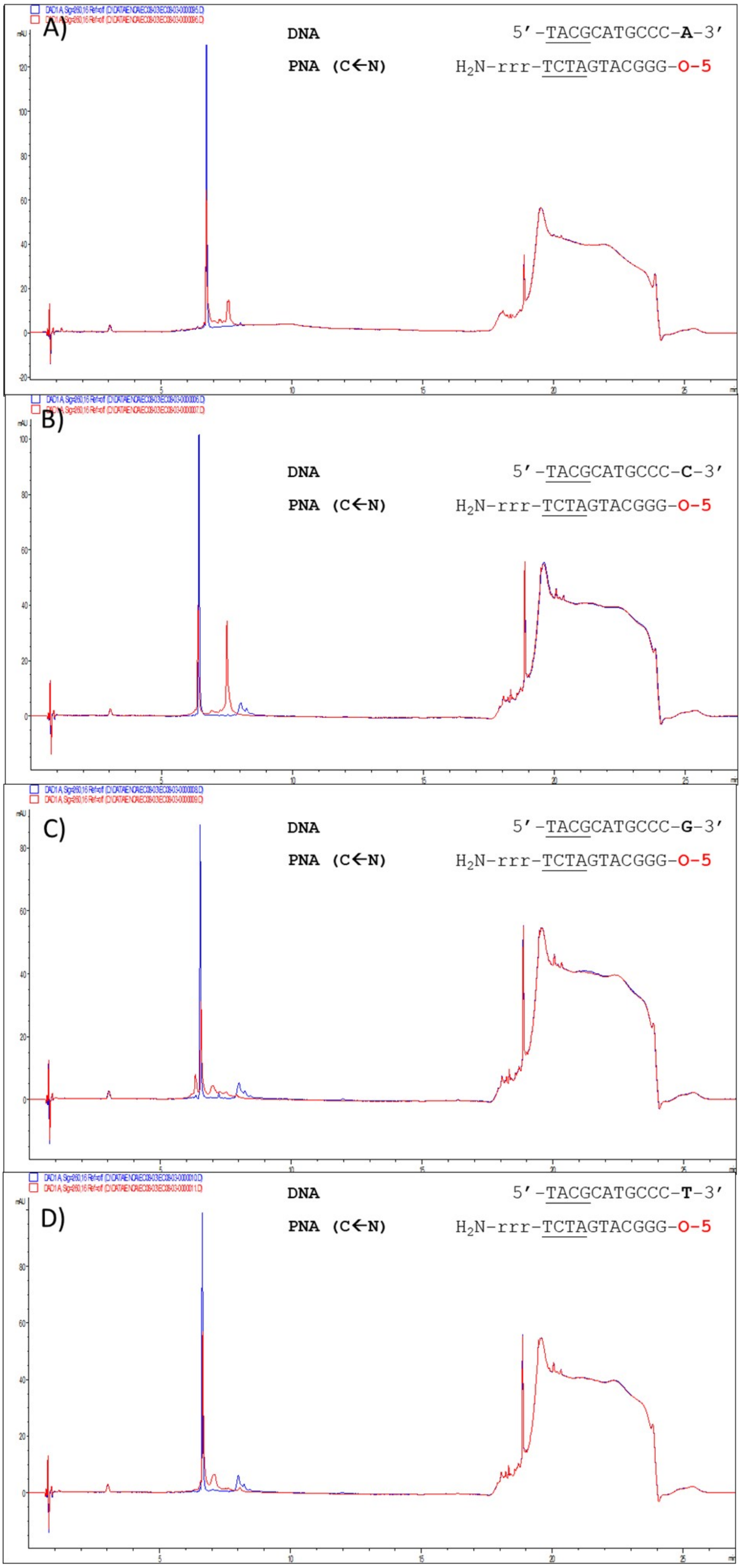

Figure S84: HPLC3 traces of crosslink experiment performed in presence of **PNA-7** in presence of **DNA-2-Z** at 0 (blue trace) and 20’ irradiation, at 2 µM MB concentration (red trace)**.** (A) + **DNA-2-A;** (B) + **DNA-2-C;** (C) + **DNA-2-G;** (D) + **DNA-2-T.**

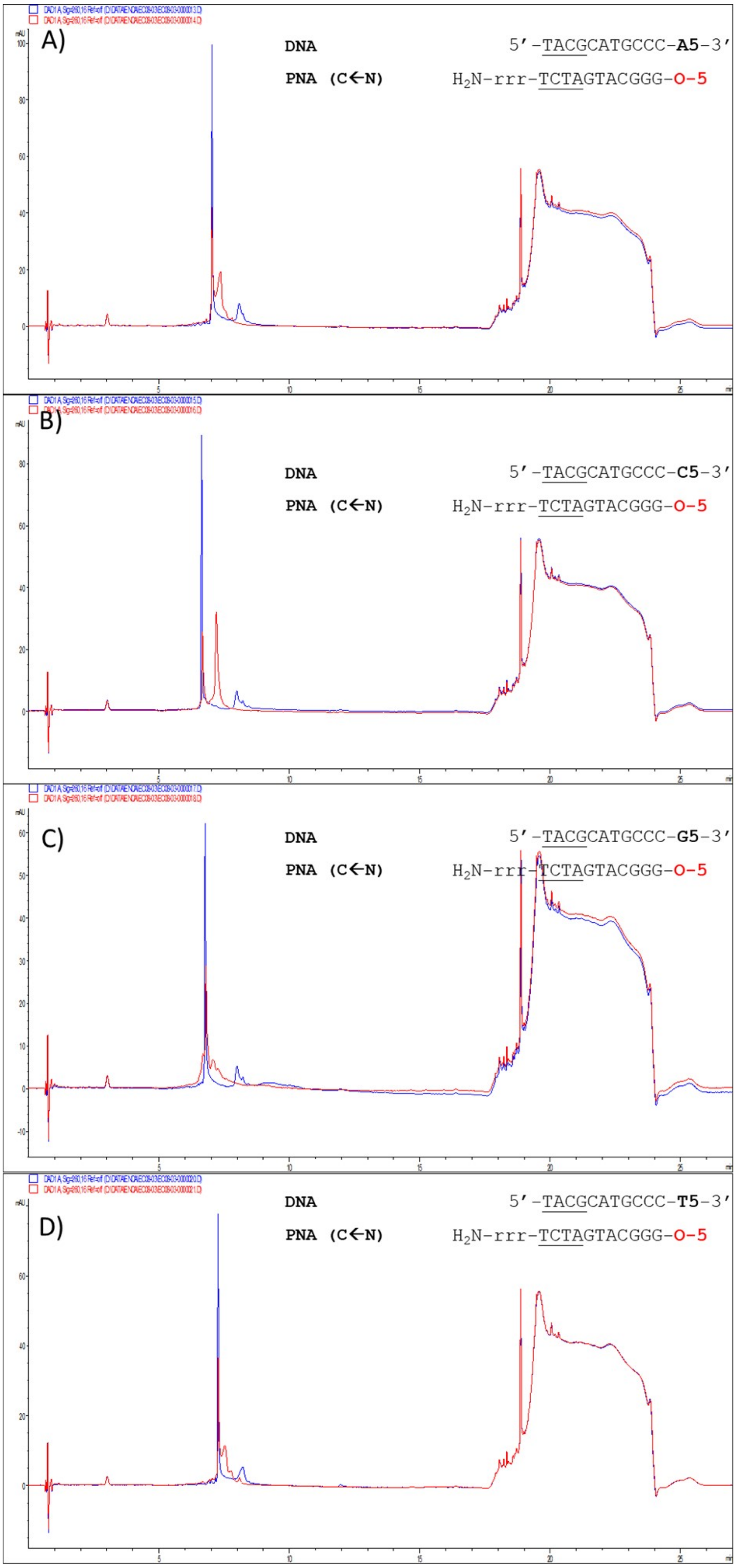

Figure S85: HPLC3 traces of crosslink experiment performed in presence of **PNA-7** in presence of **DNA-2-Z** at 0 (blue trace) and 20’ irradiation, at 2 µM MB concentration (red trace)**.** (A) + **DNA-2-A5;** (B) + **DNA-2-C5;** (C) + **DNA-2-G5;** (D) + **DNA-2-T5.**
